# Transcriptome analysis of six tissues obtained post‐mortem from sepsis patients

**DOI:** 10.1111/jcmm.17938

**Published:** 2023-09-20

**Authors:** Fabiano Pinheiro da Silva, André Nicolau Aquime Gonçalves, Amaro Nunes Duarte‐Neto, Thomaz Lüscher Dias, Hermes Vieira Barbeiro, Cristiane Naffah Souza Breda, Leandro Carvalho Dantas Breda, Niels Olsen Saraiva Câmara, Helder I. Nakaya

**Affiliations:** ^1^ Laboratório de Emergências Clínicas, Faculdade de Medicina Universidade de São Paulo São Paulo Brazil; ^2^ Faculdade de Ciências Farmacêuticas Universidade de São Paulo São Paulo Brazil; ^3^ Departamento de Patologia, Faculdade de Medicina Universidade de São Paulo São Paulo Brazil; ^4^ Departamento de Imunologia Instituto de Ciências Biomédicas, Universidade de São Paulo São Paulo Brazil; ^5^ Hospital Israelita Albert Einstein São Paulo Brazil

**Keywords:** cell response, immunity, sepsis, transcriptomics

## Abstract

Septic shock is a life‐threatening clinical condition characterized by a robust immune inflammatory response to disseminated infection. Little is known about its impact on the transcriptome of distinct human tissues. To address this, we performed RNA sequencing of samples from the prefrontal cortex, hippocampus, heart, lung, kidney and colon of seven individuals who succumbed to sepsis and seven uninfected controls. We identified that the lungs and colon were the most affected organs. While gene activation dominated, strong inhibitory signals were also detected, particularly in the lungs. We found that septic shock is an extremely heterogeneous disease, not only when different individuals are investigated, but also when comparing different tissues of the same patient. However, several pathways, such as respiratory electron transport and other metabolic functions, revealed distinctive alterations, providing evidence that tissue specificity is a hallmark of sepsis. Strikingly, we found evident signals of accelerated ageing in our sepsis population.

## 1 INTRODUCTION

Sepsis is a complex disease, which affects different organs in a heterogeneous manner and through different cellular mechanisms. The lung is the most common primary site of infection in sepsis and the organ that most frequently fails during this disease. Acute lung injury can be triggered by direct lung infection or infection at other sites and is characterized by inflammation, hypoxemia and noncardiac pulmonary oedema.^1^ Acute kidney injury (AKI) is also common in sepsis, being associated with extremely high mortality rates. Kidney damage during sepsis is attributed to renal hypoperfusion and secondary acute tubular necrosis, but the post‐mortem findings in humans and animal models are mild and do not explain the functional failure.^2^ Transient cardiac dysfunction is another common finding in sepsis and is characterized by impaired contractility, global dysfunction and diminished response to fluid resuscitation and catecholamines.^3^

Sepsis also impacts other human organs and tissues. For example, the disease alters many cognitive functions associated with the prefrontal cortex, such as attention, verbal fluency and executive functions.^4^ The hippocampus plays a major role in learning and memory, and pronounced hippocampal atrophy is a common finding in sepsis survivors.^5^ Finally, there is significant evidence that disruption of the intestinal epithelial barrier and modifications of the gut microbiome have negative effects on sepsis outcomes.^6^

Several studies have already investigated the gene expression profile of sepsis patients, through the analysis of whole blood cells or isolated leukocytes. Pioneering transcriptomic studies performed in blood samples revealed persistent repression of genes involved in adaptive immunity, and the massive activation of those involved in innate immunity from the onset of the disease,^7^, ^8^, ^9^, ^10^ proposing a new paradigm that contradicts the previous hypothesis that septic shock is a biphasic disease.

Sepsis is a lethal syndrome with heterogeneous clinical and molecular manifestations, making it difficult to investigate. Since most human studies are confined to sampling the peripheral blood, we believe they do not fully reflect what is happening in other tissues. We believe that high‐throughput transcriptomic studies that employ autopsy samples are crucial for a more comprehensive investigation into this disease. Here, we performed RNA‐seq experiments of 6 autopsy tissues (prefrontal cortex, hippocampus, heart, lung, kidney and colon) from 14 individuals, 7 of whom succumbed to septic shock.

## 2 METHODS

### 2.1 Selection of patients and sample collection and preparation

This study was approved by the Ethics Committee of our hospital (protocol #10248419.7.0000.0068), located in Sao Paulo, Brazil. Written informed consent was provided by a responsible family member, agreeing to the inclusion of the patient in this study. Seven patients who died of septic shock and seven critically ill patients who died from non‐infectious disorders underwent autopsies. All autopsies were performed less than 24 h after death. Sepsis was defined in accordance with the Sepsis‐3 criteria.^11^ Patients under 18 years old, cancer patients, those with autoimmune disorders or viral infections, and pregnant women were excluded from the study (Table S1).

All autopsies were performed by Dr Amaro Nunes Duarte‐Neto, following Letulle's technique.^12^ Tissue samples measuring 1 cm^3^ were collected from the prefrontal cortex, hippocampus, left ventricle, lung, kidney and ascending colon. The samples were then stored at −70°C for further analysis.

### 2.2 RNA extraction and sequencing

Total RNA was extracted from the tissue samples using RNeasy and AllPrep Micro Kit (Qiagen), in accordance with the manufacturer's protocol. The RNA quality of all samples showed a higher parameter measured by TapeStation 4200 (Agilent Technologies).

cDNA libraries were prepared for the 84 samples, starting with 500 pg of total RNA, using the QUANTSeq 3'mRNA‐Seq Library Prep Kit FWD HT for Illumina (Lexogen), in accordance with the manufacturer's protocol. The libraries were quantified by Qubit dsDNA High Sensitivity Assay Kit (Life Technologies Corporation) and the median sizes were determined by TapeStation 4200 (Agilent Technologies), using the High Sensitivity D1000 ScreenTape assay, to form an equimolar pool. Sequencing was performed as a 75‐bp single‐read, single‐index run on a NextSeq 500 next‐generation sequencer (Illumina) with High Output kit.

## 3 RESULTS

We performed RNA‐seq on frozen autopsy samples of six distinct tissues (prefrontal cortex, hippocampus, heart, lung, kidney and colon) obtained from seven patients who died of septic shock and seven critically ill patients who died of non‐infectious diseases. The lungs were the primary site of infection in four of our sepsis patients. The other primary sources of infection were meningitis, soft‐tissue infection and duodenal perforation. All seven patients with non‐infectious disorders died of cardiogenic or hypovolemic shock (Table S1).

**FIGURE 1:**
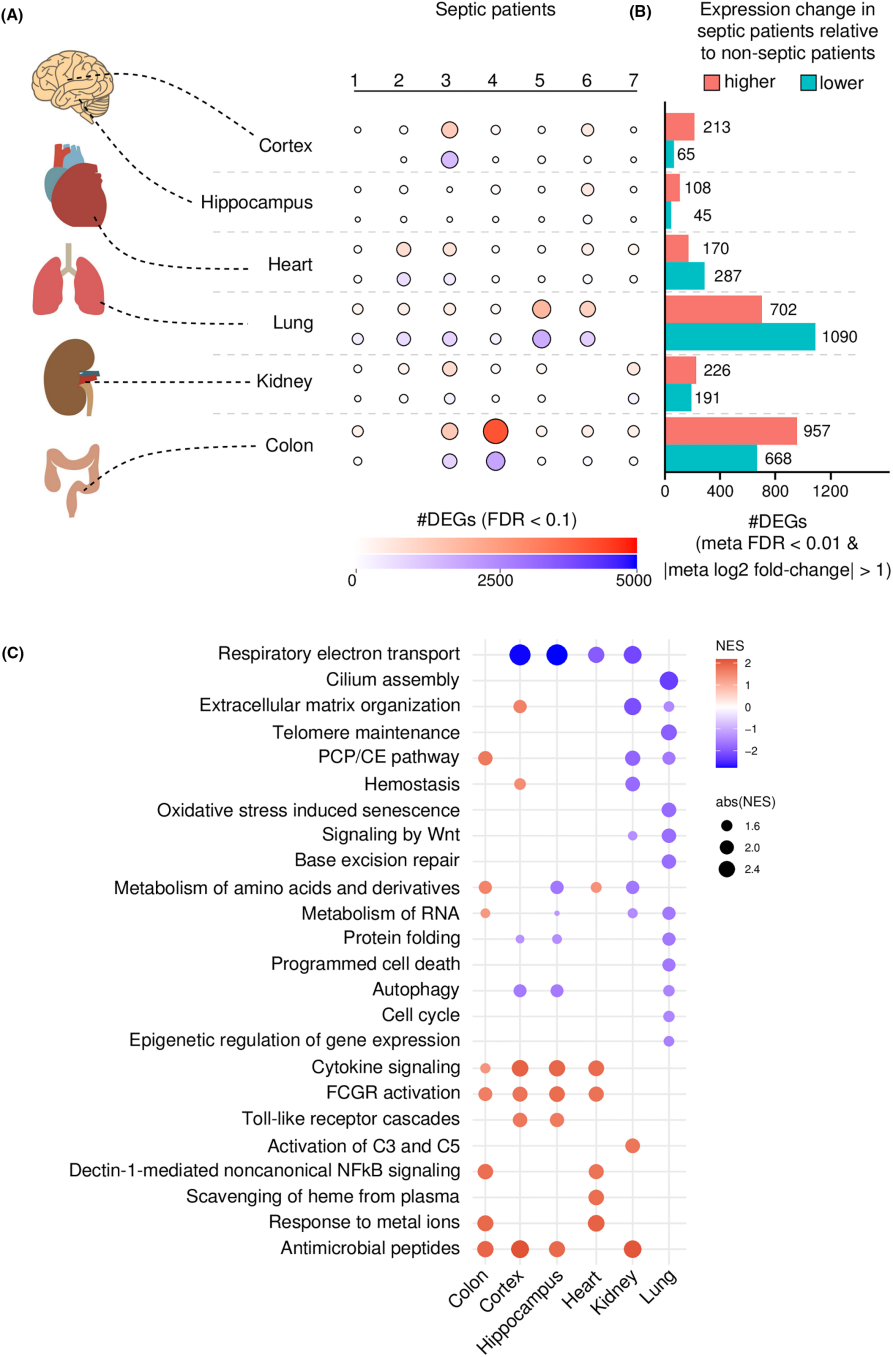
(A) Total number of differentially expressed genes (DEGs) detected per tissue in each of the sepsis patients. (B) Total numbers of upregulated (higher) and downregulated (lower) DEGs in each tissue of the sepsis patients, in comparison with the controls. (C) Normalized enrichment score (NES) of 24 key pathways identified in our study.

To analyse the whole transcriptome in these six organs, we compared each septic shock patient to the group of seven uninfected control individuals (Figure 1A). We believe that this approach has the potential to provide valuable insights into the molecular mechanisms underlying sepsis. By comparing the transcriptomics of a single patient to a group of controls, we can identify changes at the individual level that may be missed when comparing groups of patients. While patient #3 exhibited profound transcriptional alterations in the brain and colon, in patients #5 and #6 the lungs were the most affected organ (Figure 1A). The source of infection did not appear to be the main factor determining these differences because patient #6, for example, died of purulent meningitis (Table S1). This observation provides evidence of the great heterogeneity of our sepsis patients, upon comparing different patients at the transcriptional level.

We also performed differential expression analysis between all seven septic shock patients and seven control individuals (Figure 1B). This analysis revealed that the lungs and colon were the most affected organs, with 1792 and 1625 differentially expressed genes (DEGs) between the two groups, respectively. In the prefrontal cortex, hippocampus and kidney, a total of 278, 153 and 417 DEGs were detected, respectively (Figure 1B). While upregulation dominated in the prefrontal cortex, hippocampus and ascending colon, the total number of downregulated genes surpassed the number of upregulated genes in the lungs and heart (Figure 1B).

Enrichment analysis using the Reactome database detected more than 370 pathways significantly affected in one or more tissues in the sepsis patients (Table S3). Among these pathways, 24 were selected by curation for detailed analysis and are discussed in detail below (Figure 1C).

Some pathways were detected in several tissues, while other pathways were specific to one or two tissues. Examining the pathways detected in most tissues, we could see that the pattern of activation varied depending on the tissue investigated. Indeed, we even found pathways that were upregulated in some tissues and downregulated in others, confirming the heterogeneity of the disease when different tissues are compared in the same patient and providing evidence of the complexity of the mechanisms involved.

As expected, many pathways related to immune recognition and defence against infection were identified by our enrichment analysis (Figure 1C). The ‘Toll‐like receptor cascades’, ‘cytokine signalling’, ‘activation of C3 and C5’, ‘FcγR activation’, ‘antimicrobial peptides’ and ‘dectin‐1 mediated non‐canonical NF‐κB signalling’ pathways were strongly activated in our sepsis patients, at times in several tissues.

**FIGURE 2:**
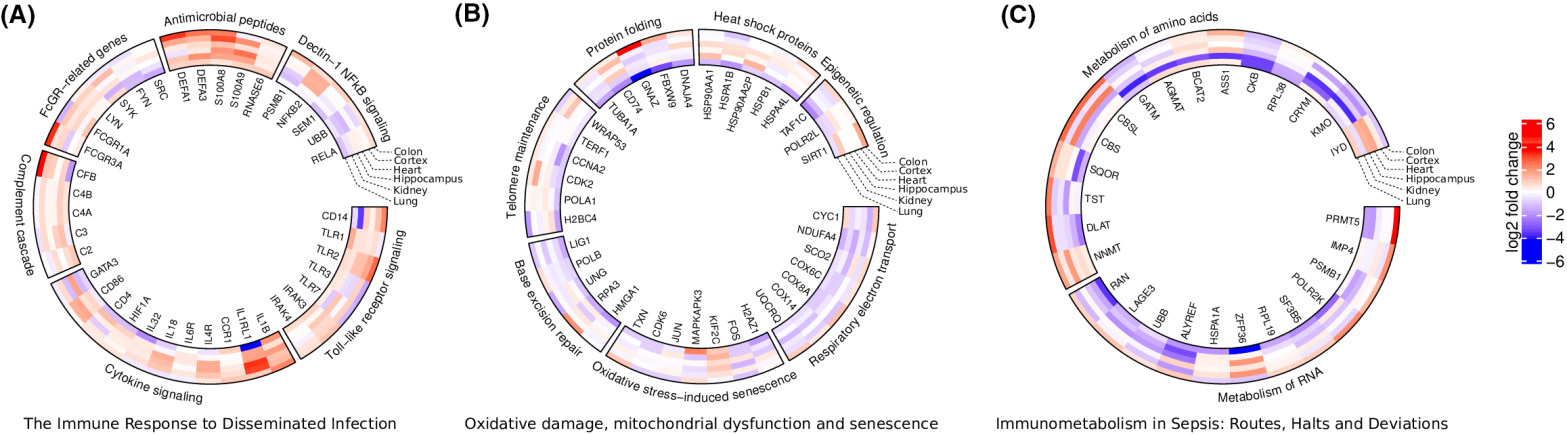
Circos plots demonstrating the pattern of activation or inhibition of various differentially expressed genes (DEGs) identified in our tissue samples. (A) Immune response to disseminated infection. (B) Oxidative damage, mitochondrial dysfunction and senescence. (C) RNA and amino acid metabolism.

Interestingly, Toll‐like receptor signalling was detected in sepsis, specifically in the brain (prefrontal cortex and hippocampus), via the upregulation of genes such as CD14, TLR1, TLR2, TLR3, TLR7, IRAK3 and IRAK4 (Figure 2A). In contrast, the upregulation of cytokine signalling was identified in most organs, except in the kidneys and lungs. Genes related to this pathway detected in our study include IL1B, IL1RL1, CCR1, IL4R, IL6R, IL18, IL32, HIF1A, CD4, CD86 and GATA3 (Figure 2A). Our results thus indicate that massive activation of both innate and adaptive immunity occurred, at least in some organs, at the end of life in our sepsis patients.

Activation of the complement cascade (components C3 and C5) was detected in the kidneys in our sepsis patients, but could not be detected in any other tissue. Genes related to this pathway detected in our study include C2, C3, C4A, C4B and CFB (Figure 2A). The upregulation of FcγR‐related genes, such as FCGR3A, FCGR1A, LYN, SYK, FYN and SRC, was detected in the prefrontal cortex, hippocampus, heart and colon of our sepsis patients, but not in the kidneys or lungs (Figure 2A).

Antimicrobial peptides are well‐conserved components of the innate immune system that have direct bactericidal properties, while also being able to regulate many host defence mechanisms.^13^ This pathway was strongly upregulated in the prefrontal cortex, hippocampus, kidneys and colon of our sepsis patients. Examples of genes related to this pathway that were differentially expressed in our sepsis samples include DEFA1, DEFA3, S100A8, S100A9 and RNASE6 (Figure 2A).

Non‐canonical NF‐κB signalling mediated by the upregulation of Dectin‐1 was detected in the heart and colon in our sepsis patients. Dectin‐1 is a membrane receptor that recognizes β‐glucans, inducing phagocytosis, the production of ROS and cytokine secretion. Dectin‐1 also boosts the responses of specific Toll‐like receptors (TLRs) and activates the NLRP3 inflammasome. Dectin‐1 activates both canonical and non‐canonical NF‐κB activation. Non‐canonical NF‐κB signalling induces more sustained NF‐κB activation and is mediated by Raf‐1, instead of Syk.^14^ Genes that participate in this pathway and were differentially expressed in our sepsis patients include PSMB1, NFKB2, SEM1, UBB and RELA (Figure 2A).

Another critically affected pathway in sepsis is ‘respiratory electron transport’. This pathway was strongly inhibited in the prefrontal cortex and hippocampus, while its inhibition was also detected at lower levels in the kidneys and heart. Genes that belong to this pathway include CYC1, NDUFA4, SCO2, COX6C, COX8A, COX14 and UQCRQ (Figure 2B).

The identification of the ‘oxidative stress‐induced senescence’, ‘base excision repair’, ‘telomere maintenance’, ‘protein folding’ and ‘epigenetic regulation of gene expression’ pathways in our sepsis patients should be mentioned here because all these pathways, together with ‘respiratory electron transport’, are strongly interconnected and play major roles in sepsis and ageing (Figure 2B).

The ‘oxidative stress‐induced senescence’, ‘base excision repair’ and ‘telomere maintenance’ pathways were detected exclusively in the lungs in our sepsis patients, where they were strongly inhibited. Genes associated with the ‘oxidative stress‐induced senescence’ pathway include H2AZ1, FOS, KIF2C, MAPKAPK3, JUN, CDK6 and TXN (Figure 2B). The ‘base excision repair’ pathway was identified in the form of genes such as HMGA1, RPA3, UNG, POLB and LIG1, while the ‘telomere maintenance’ pathway was detected via genes such as H2AZ1, H2BC4, POLA1, RPA3, CDK2, CCNA2, TERF1 and WRAP53 (Figure 2B).

The protein folding pathway was inhibited in the lungs, and to a lesser extent in the brain (cortex and hippocampus). This was detected in the form of genes such as TUBA1A, CD74, GNAZ, FBXW9 and DNAJA4. Several heat shock proteins also participate in this pathway, including HSP90AA1, HSPA1B, HSP90AA2P, HSPB1, HSPE3 and HSPA4L (Figure 2B).

Epigenetic regulation of gene expression was inhibited exclusively in the lungs, in the form of genes such as TAF1C, H2AZ1, H2BC4, POLR2L, SIRT1 and SIN3 (Figure 2B).

The ‘metabolism of RNA’ and ‘metabolism of amino acids and derivatives’ pathways were also selected for a more detailed analysis. This was because, according to our data, sepsis targets RNA and amino acid metabolism at many levels (Figure 2C).

While the ‘metabolism of RNA’ was upregulated in the colon in our sepsis patients, it was downregulated in the hippocampus, kidneys and lungs. Upregulated genes include PRMT5, IMP4, PSMB1, POLR2K, SF3B5 and RPL19, while downregulated ones include ZFP36, HSPA1A, ALYREF, UBB, LAGE3 and RAN (Figure 2C). Similarly, the ‘eukaryotic translation elongation’ and ‘eukaryotic translation initiation’ pathways were also upregulated in the colon and downregulated in the hippocampus, kidneys and lungs in our sepsis patients. In contrast, mRNA splicing and rRNA processing were only affected in the kidneys and lungs, where they were inhibited. Moreover, the ‘regulation of mRNA stability by proteins that bind AU‐rich elements’ pathway was upregulated in the heart and downregulated in the lungs in our sepsis patients. Finally, the ‘negative epigenetic regulation of rRNA expression’, ‘gene silencing by RNA’ and ‘transcriptional regulation by small RNAs’ pathways were also inhibited in our sepsis patients, exclusively in the lungs (Table S3). Sepsis, thus, deregulates many processes related to RNA functions and homeostasis.

The metabolism of amino acids and their derivatives was upregulated in the colon and heart in our sepsis patients, while being inhibited in the hippocampus and kidney. Upregulated genes include NNMT, DLAT, TST, SQOR, PSMB1, CBS and CBSL, while downregulated ones include GATM, AGMAT, BCAT2, ASS1, CKB, RPL38, CRYM, KMO and IYD (Figure 2C).

We detected inhibition of the ‘diseases of glycosylation’ and ‘diseases associated with O‐glycosylation of proteins’ pathways in the kidney of our sepsis patients, as well as alterations of the ‘asparagine N‐linked glycosylation’ pathway in the colon (upregulated) and brain (downregulated). Meanwhile, the ‘regulation of insulin‐like growth factor transport and uptake by insulin‐like growth factor binding proteins’ pathway was upregulated in the cortex and downregulated in the kidneys (Table S3).

Global metabolism of lipids was affected exclusively in the hippocampus and kidneys in our sepsis patients, where its downregulation could be detected. However, there were some exceptions to this for specific pathways. For example, the ‘eicosanoid ligand binding receptors’ pathway exhibited remarkable upregulation in the heart (Table S3).

**FIGURE 3:**
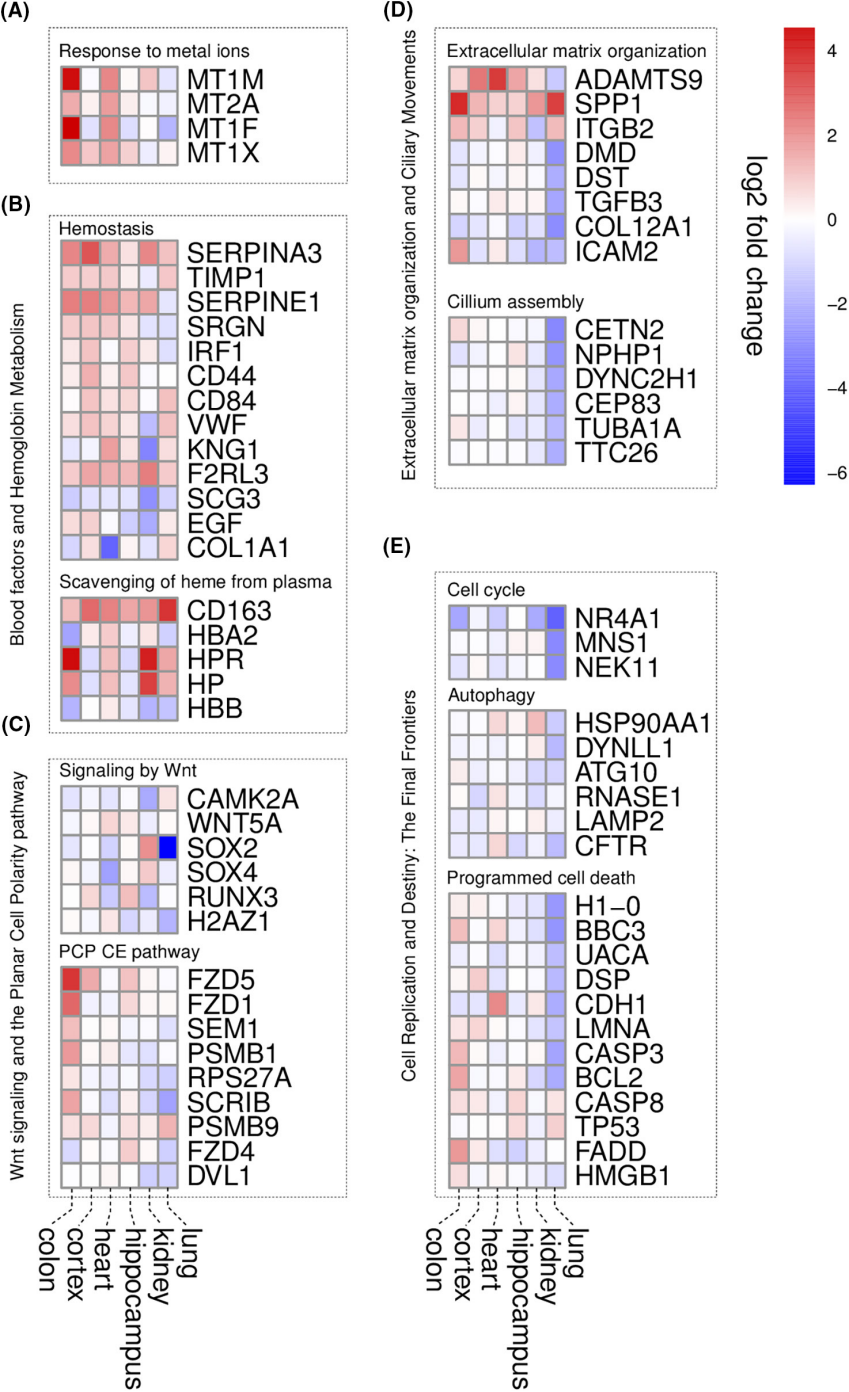
Heat maps showing the pattern of activation or inhibition of various differentially expressed genes (DEGs) identified in our tissue samples. (A) Response to metal ions (B) Haemostasis and scavenging of haem from plasma. (C) Signalling by Wnt and PCP CE pathway. (D) Extracellular matrix organization and cilium assembly. (E) Cell cycle, autophagy and programmed cell death.

The ‘response to metal ions’ pathway was significantly activated in the prefrontal cortex and heart in our sepsis population. This response was detected via the upregulation of genes encoding several metallothioneins, such as MT1M, MT2A, MT1F and MT1X (Figure 3A).

The ‘haemostasis’ pathway was activated in the prefrontal cortex and inhibited in the kidneys in our sepsis patients. Upregulated genes include SERPINA3, TIMP1, SERPINE1, SRGN, IRF1, CD44, CD84 and VWF, while downregulated ones include SERPINA3, KNG1, F2RL3, SERPINE1, SCG3, EGF and COL1A1 (Figure 3B).

The ‘scavenging of haem from plasma’ pathway was upregulated in the heart in our sepsis patients. Genes associated with this finding include CD163, HBA2, HPR, HP and HBB (Figure 3B).

The ‘signalling by Wnt’ pathway was inhibited in the kidneys and lungs in our sepsis patients. Genes associated with this are CAMK2A, WNT5A, SOX2, SOX4, RUNX3 and H2AZ1 (Figure 3C). Moreover, the ‘PCP CE pathway’ was activated in the colon and inhibited in the kidneys and lungs in our sepsis patients. Upregulated genes include FZD5, FZD1, SEM1, PSMB1 and CTLA, while downregulated ones include RPS27A, SCRIB, PSMB9, FZD4 and DVL1 (Figure 3C).

The ‘extracellular matrix organization’ pathway was found to be activated in the prefrontal cortex and inhibited in the kidneys and lungs in our sepsis patients. Upregulated genes in these patients include ADAMTS9, SPP1, TIMP1, SERPINE1, CD44 and ITGB2, while downregulated ones include SPP1, DMD, DST, DIMP1, TGFB3, COL12A1 and ICAM2 (Figure 3D).

The ‘cilium assembly’ pathway was strongly inhibited in the lungs in our sepsis patients. Genes associated with this include CETN2, NPHP1, DYNC2H1, CEP83, TUBA1A and TTC26 (Figure 3D).

The ‘cell cycle’ pathway was inhibited in the lungs of our sepsis patients. This kind of signalling was detected via the downregulation of genes such as SOX2, NR4A1, MNS1, NEK11, CETN2 and TUBA1A (Figure 3E).

The ‘autophagy’ pathway was inhibited in the brain (prefrontal cortex and hippocampus) and lungs in our sepsis patients. Genes found to be associated with this cellular response include HBB, TUBA1A, HSP90AA1, DYNLL1, ATG10, RNASE1, LAMP2 and CFTR (Figure 3E).

The ‘programmed cell death’ pathway was also significantly affected only in the lungs of our sepsis patients, which was found to be downregulated. Genes associated with this finding include H1‐0, BBC3, UACA, DSP, CDH1, LMNA, CASP3, BCL2, CASP8, TP53, FADD and HMGB1 (Figure 3E).

## 4 DISCUSSION

Our study is the first RNA sequencing (RNA‐seq) analysis conducted on autopsy samples from septic patients, investigating multiple tissues. Brusletto et al. performed a transcriptome study on five human organs, but they used DNA microarrays, which have lower sensitivity and dynamic range compared to RNA‐seq technology. Moreover, their study only involved five meningococcal septic shock patients and two uninfected controls (sudden deaths).^15^ In their research, they found significant upregulation of Toll‐like receptors, acute phase response, TREM1, IL6, HMGB1, p38, IL1, PPARα/RXRα and NFκB signalling pathways in the lungs. However, we could not detect upregulation of these pathways in our lung samples. For heart tissue, they observed upregulation of EIF2, TREM1, P13/AKT and HMGB1 signalling pathways, along with a significant downregulation of PPAR signalling. In our heart samples, we identified upregulation of cytokines and FcγR signalling and downregulation of respiratory electron transport pathways as the most notable results. In the kidney samples, they identified significant upregulation of several peptidases such as EIF2, C3, HP and SOD2, as well as SERPINE1, while we identified activation of antimicrobial peptides and complement signalling in the presence of inhibition of respiratory electron transport, haemostasis and extracellular matrix organization pathways. Notably, they also investigated two organs which play an important role during systemic inflammation and sepsis that have not been investigated in our study, the spleen and the liver. The lungs exhibited the most extensive changes in gene expression, while the spleen had the fewest regulated genes. They concluded that each organ manifests a distinct transcriptional signature in meningococcal septic shock.

Bustamante et al. compared RNA isolated from the parietal cortex grey matter of 12 subjects who died of sepsis and 12 who died of a non‐infectious critical illness, finding 176 differentially expressed genes (DEGs). The most differentially expressed genes were related to immunity, including damage‐associated molecular patterns (S100A8, S100A9 and members of the HSP family), markers of astrocyte activation (HSPB2, GBP2 and SERPINA3) and macrophage and microglial signatures (SOC3, CHI3L2 and CHI3L1). They did not investigate other organs or tissues.^16^

### 4.1 Oxidative damage, mitochondrial dysfunction and senescence

Many researchers have already investigated sepsis‐induced mitochondrial dysfunction.^17^, ^18^ Sepsis is accompanied by an increase in oxidative damage, which is caused by numerous factors, including the production of reactive oxygen species (ROS) by immune cells, increased xanthine oxidase activity, increased nitric oxide plasma levels and decreased antioxidant serum capacity. Proinflammatory mediators and oxidative stress impair the function of the enzymes of the respiratory chain and lead to structural damage.^19^

Cellular senescence, genomic instability, telomere attrition, loss of proteostasis, epigenetic alterations and mitochondrial dysfunction constitute six of the nine classical hallmarks of ageing,^20^ providing evidence that most mechanisms that characterize the ageing process have a role in the pathophysiology of septic shock.^21^ With the exception of ‘oxidative stress‐induced senescence’, the inhibition of these Reactome pathways disturbs cell homeostasis and leads to accelerated ageing.

It is surprising that the ‘oxidative stress‐induced senescence’ pathway is inhibited in the lungs in sepsis, since it is a cell response to stress. We expected this pathway to be upregulated, so this finding may be a sign of late sepsis and immunosuppression associated with extensive mitochondrial damage.

Evidence that sepsis induces epigenetic changes have already been reported.^22^ Epigenetic changes exert sustained effects on gene expression that can be beneficial to the organism but, in the end, can also lead to aberrant gene expression and genome instability.

There was striking downregulation of many pathways in the lungs in our sepsis patients, compared with the findings in other tissues. The lungs are the most common primary site of infection in sepsis and the organ that most frequently fails during this disease. According to our results, it is also the organ that experiences the most robust decline in cellular activity.

### 4.2 Immunometabolism in sepsis

Globally, in our sepsis patients, RNA and amino acid metabolism appears to be more affected than carbohydrate and lipid metabolism. However, this type of broad conclusion should be drawn with caution because further investigations are required.

Glycosylation, a very common post‐translational protein modification, was also affected. In fact, the covalent attachment of carbohydrates is the most common and diverse modification in proteins, altering their structures, functions and localization. A few studies have investigated the role of glycosylation in sepsis.^23^, ^24^ A recent study also performed an N‐glycoproteomic analysis in patients with bloodstream infections and detected several interesting N‐glycopeptides that merit deeper analyses.^25^

### 4.3 Metals in cell signalling: from transcription factors to enzymatic activity

Metallothioneins are a family of low‐molecular‐weight proteins bound to the membrane of the Golgi complex. They bind both physiological and xenobiotic heavy metals, regulating zinc and cooper levels within the body and protecting against metal toxicity and oxidative stress.^26^

Zinc and copper play crucial roles in various molecular processes. Zinc can bind to more than 300 enzymes (including SOD1) and more than 2000 transcription factors (including NF‐κB and HIF‐1α) with significant roles in fundamental cellular functions, such as DNA replication, DNA damage repair, apoptosis and immunity.^27^ This trace element is also crucial to ensuring that virtually all immune cells function appropriately. Zinc deficiency impairs both innate and adaptive immunity,^28^ and dysregulation of Zn homeostasis is significantly involved in the development of cardiovascular diseases.^29^ Moreover, in the brain, zinc deficiency has been associated with many neurodegenerative disorders, leading to learning and memory deficits.^30^

An analysis of serum from sepsis patients in an intensive care unit (ICU) revealed that serum zinc concentrations were reduced compared with those in a healthy control group or the normal physiological range. In fact, the redistribution of zinc from serum into the liver has been observed during sepsis and several studies have implied a correlation between zinc and sepsis outcome.^31^

The redistribution of zinc and its accumulation in the liver may have some protective effects for the host, restricting pathogens' access to essential transition metals. However, this process may also have some adverse effects. Possible symptoms of hypozincaemia include higher levels of proinflammatory cytokines and oxidative damage to proteins, lipids and DNA.^32^

Superoxide dismutase 1 (SOD1) is a zinc‐ and copper‐dependent enzyme. Its dysfunction causes excessive levels of oxygen and nitrogen reactive species (ROS and NRS, respectively), leading to decreased nitrogen oxide (NO) synthesis. NO is a key modulator of vasodilation and the reduction of its level plays an important role in the pathogenesis of many cardiovascular diseases.^33^

The matrix metalloproteinases (MMPs) also share a common Zn binding sequence motif. These metalloproteinases are tightly regulated and their expression is transcriptionally controlled by inflammatory cytokines and growth factors within the extracellular matrix, as well as hormones and cell–matrix interactions. MMPs play important roles in immunity, control of the vascular tone and many other cellular processes.^34^

The A disintegrin and metalloprotease (ADAM) family of proteases is another important family containing Zn binding sequences. ADAMs have been implicated in the shedding of six out of the seven known EGFR ligands (transforming growth factor alpha [TGFα], epidermal growth factor [EGF], HB‐EGF, betacellulin, epiregulin and amphiregulin).^35^, ^36^ EGFRs are essential for cardiac responses to physiological and pathological perturbations. The EGFR family appears to be critical in protecting cardiac cells from injury^37^ and its potential therapeutic application is under investigation.^38^

### 4.4 Blood factors and haemoglobin metabolism

Systemic inflammation invariably leads to endothelial dysfunction and activation of the coagulation system.^39^ Proinflammatory mediators can activate the coagulation system and downregulate crucial anticoagulant mechanisms. The initiation of coagulation and consequent thrombin generation are triggered by the expression of tissue factor on activated monocytes and endothelial cells. At the same time, endothelial‐associated anticoagulant pathways, in particular the protein C system, are impaired by proinflammatory cytokines. Furthermore, fibrin removal is severely obstructed by inactivation of the endogenous fibrinolytic system, mainly due to upregulation of plasminogen activator inhibitor type 1.^39^ Overall, this process leads to the deposition of microvascular clots, which may contribute to tissue ischaemia and organ dysfunction. In severe disease, the coagulation system becomes diffusely activated, with the consumption of multiple clotting factors, resulting in disseminated intravascular coagulation (DIC).^40^ Notably, in a vicious circle, components of the coagulation system trigger further inflammation through the release of proteases into circulation and the activation of platelets.^41^, ^42^

CD163 is a scavenger receptor for haptoglobin–haemoglobin complexes expressed by cells of the macrophage/monocyte lineage, which is cleaved to become soluble CD163 by inflammatory stimuli. In addition to the prevention of oxidative stress by haem,^43^ the removal of haemoglobin establishes an environment with a shortage of iron for bacterial growth, with critical implications in the pathogenesis of sepsis.^44^

### 4.5 Wnt signalling and the planar cell polarity pathway

In adults, canonical Wnt/β‐catenin signalling maintains a variety of stem cell pools in many organs and tissues.^45^ β‐Catenin and many of the Wnt effector proteins are ubiquitinated and their levels are tightly regulated to ensure precise coordination of development and tissue homeostasis.^46^ The Wnt/β‐catenin pathway also interferes with inflammasome signalling.^45^ Confirming this observation, inhibition of Wnt/β‐catenin signalling was reported to reduce inflammation and mitigate organ injury in experimental sepsis.^47^

The planar cell polarity (PCP) pathway is activated by the binding of Wnt to Frizzled proteins and its co‐receptor. Frizzled proteins (Fzd) are membrane receptors that participate in multiple signal transduction pathways. They bind secreted Wnt proteins and other ligands, and are essential for cell polarity, cell proliferation, tissue homeostasis, tissue repair and the formation of neural synapses.

### 4.6 Extracellular matrix organization and ciliary movements

Increasing evidence shows that complex host–pathogen interactions occur in the extracellular matrix. Indeed, a pathogen's binding or degradation of the extracellular matrix facilitates adhesion and penetration into the host.^48^, ^49^ The extracellular matrix is also a site where the host directs tissue‐specific responses to infection, modulating various cellular events.^50^

Microtubules act as a scaffold to determine cell shape, and provide a microstructure for cell organelles and vesicles to move on. When arranged inside cilia, they are used for locomotion.^51^ Multiple pathogenic microbes have evolved mechanisms to resist mucociliary clearance to colonize the airway. Common pathogenic bacteria such as *Streptococcus pneumoniae*, *Pseudomonas aeruginosa* and *Haemophilus influenzae* produce virulence factors that impair ciliary motion and coordination.^52^

### 4.7 Cell replication and fate

The presence of stress inhibits the cell cycle. If major cell damage has occurred and cannot be repaired, the cell will probably die via apoptosis, being eliminated by phagocytosis. Death by autophagy is different, in that the cell components are degraded and recycled at the intracellular level by the fusion of autophagosomes with lysosomes. Other forms of cell death include necrosis, pyroptosis and necroptosis.

The consequence of excessive cell death is a loss of function. For that reason, it has been asserted that extensive programmed cell death is a critical factor in the pathophysiology of multiple organ dysfunction syndrome (MODS), a common finding in prolonged septic shock.^53^ However, our analysis identified inhibition of the ‘autophagy’ and ‘programmed cell death’ pathways, instead of their activation, and this inhibition was only found in the sepsis‐affected lungs. Our findings thus do not corroborate that apoptosis is a common finding in sepsis.

There is significant crosstalk among the various forms of cell death. For example, the inhibition of caspases can switch cell death from apoptosis to necrosis.^54^ This appears to be the case in the lungs of our sepsis patients. Indeed, while the lungs are a large organ, with high functional capacity that can even receive external mechanical support to work, organs such as the heart, brain and even colon cannot resist extensive necrosis, explaining at least in part why necrosis would prevail there.

In conclusion, our findings confirm that sepsis interferes with various molecular processes, corroborating previous publications that suggest defects in many metabolic and immune pathways in this disease,^55^ occurring in concert with extensive metabolic reprogramming.^56^ According to our analysis, depending on the tissue investigated, certain innate immune pathways are preferentially activated in sepsis. This tissue selectivity is intriguing because most of these pathways are supposed to work in concert. Moreover, none of our selected pathways was identified in the lungs, suggesting that a certain degree of immunosuppression is compartmentalized there or an attempt to control exacerbated cell activation (Figure 1C). A total of 120 genes found in the analysis between sepsis and non‐sepsis patients, however, are shared among three or more tissues (Table S2). Many of these genes are crucial for immune regulation (encoding Fc receptors, complement components, antimicrobial peptides and cytokines, etc.) and haemoglobin metabolism (CD163, HP, HBA1 and HBA2). This demonstrates that these genes play fundamental roles in the pathophysiology of sepsis and do not depend on tissue‐specific regulation.

By analysing the molecular changes in the prefrontal cortex, hippocampus, heart, lung, kidney and colon of sepsis patients, we identified signatures that were shared or specific to some of these tissues. Our findings revealed that sepsis exhibits striking heterogeneity, even when comparing different tissues of the same patient. A certain degree of tissue specificity, however, could be detected. Indeed, important cellular mechanisms such as immune defence against infection, respiratory electron transport, haemoglobin scavenging, as well as several protein and RNA metabolic processes, were significantly affected in some tissues, but not in others. Characterization of the patterns of gene expression in various tissues thus has the potential to open up new avenues to obtain a better understanding of the molecular aspects of sepsis.

It is noteworthy that some data are in contrast with those obtained in animal models. This may be explained by the delay between the onset of sepsis and the occurrence of death, the consequences of long‐term intensive care provided to the patients, the use of irrelevant animal models and the intrinsic differences between humans and rodents.^57^

Our study has some limitations. We investigated a heterogeneous group of septic shock patients in terms of the source of infection, so variabilities observed between the seven patients could reflect this parameter. For example, genes up‐ or downregulated in the gut following a peritoneal infection could be different from those in a patient with pneumonia. The same hypothesis could be proposed when comparing the activated or suppressed genes in the brain of a patient with meningitis as compared to a patient dying of lung infection.^58^ Other sources of heterogeneity could be the nature of the infection, either Gram‐positive or Gram‐negative^59^, ^60^ and the length between sepsis diagnosis and the occurrence of death. A similar criticism can be made of the control group, since the causes of death also vary.

### 4.8 Transcriptome analysis

Quality control analysis was performed using FastQC software (www.bioinformatics.babraham.ac.uk/projects/fastqc/), showing a Phred value superior to 30 (data not shown).

Trimmomatic software was used to trim low‐quality reads and adapters.^61^ Raw reads were aligned to the hg38 reference through HISAT2 software.^62^ Quantification of the gene expression data was performed through the function featureCounts of the R package Rsubread and the counts were normalized according to log2CPM. Differential expression analysis was performed by the R package edgeR (FDR <0.1 was considered significant), comparing each male patient with sepsis with all male uninfected controls and the female patients with sepsis with all female uninfected controls.^63^ The results were combined using the R package MetaVolcano and enrichment analyses were performed with the Reactome database using the R package fgsea. The MetaVolcano DEG criteria were meta FDR <0.1 and absolute log2 fold‐change >1.

## AUTHOR CONTRIBUTIONS

**Fabiano Pinheiro da Silva:** Conceptualization (lead); data curation (equal); formal analysis (equal); funding acquisition (lead); investigation (equal); methodology (equal); project administration (equal); resources (equal); supervision (equal); writing – original draft (lead); writing – review and editing (equal). **André Nicolau Aquime Gonçalves:** Formal analysis (equal); investigation (equal); methodology (equal). **Amaro Nunes Duarte‐Neto:** Investigation (equal); methodology (equal). **Thomaz Luscher Dias:** Formal analysis (equal); methodology (equal). **Hermes Vieira Barbeiro:** Methodology (equal). **Cristiane Naffah Souza Breda:** Investigation (equal); methodology (equal). **Leandro Carvalho Dantas Breda:** Methodology (equal). **Niels Olsen Saraiva Camara:** Supervision (equal). **Helder I Nakaya:** Data curation (equal); formal analysis (equal); investigation (equal); methodology (equal); project administration (equal); resources (equal); supervision (equal); writing – review and editing (equal).

## CONFLICT OF INTEREST STATEMENT

The authors have no financial or ethical conflicts of interest.

## CODE AVAILABILITY

Additional information about the algorithms used will be shared upon reasonable request.

## Supporting information


Table S1.

Table S2.

Table S3.

## Data Availability

Raw and processed data were deposited at the Gene Expression Omnibus (accession number GSE237861).
