# Supplementary material for: Transcriptome analysis of six tissues obtained post‐mortem from sepsis patients
Source: J Cell Mol Med. 2023 Sep 20;27(20):3157–67. doi: 10.1111/jcmm.17938 (PMC10568675; doi:10.1111/jcmm.17938)
Supplement: Supplementary file 1 — Table S1. [file JCMM-27-3157-s002.docx]

| **ID** | **age (years)** | **sex** | **cause of death I** | **cause of death II** | **cause of death III** | **cause of death IV** |
| --- | --- | --- | --- | --- | --- | --- |
| **control 1** | **62** | **F** | **Hemorrhagic Shock** | **Aortic Dissection** | **Arterial Hypertension** |  |
| **control 2** | **52** | **F** | **Pulmonary Edema** | **Heart Failure** | **Arterial Hypertension** | **Kidney Failure** |
| **control 3** | **63** | **M** | **Hypovolemic Shock** | **Aortic Dissection** | **Atherosclerosis** |  |
| **control 4** | **48** | **M** | **Pulmonary Edema** | **Heart Attack** | **Atherosclerosis** |  |
| **control 5** | **71** | **M** | **Heart Failure** | **Kidney Failure** | **Atherosclerosis** |  |
| **control 6** | **78** | **M** | **Cardiogenic Shock** | **Tension Pneumothorax** | **Zenker’s Diverticulum Perforation** |  |
| **control 7** | **61** | **F** | **Pulmonary Edema** | **Heart Failure** | **Arterial Hypertension** | **Kidney Failure** |
| **sepsis 1** | **61** | **M** | **Septic Shock** | **Soft Tissue Infection** | **Supurative Orchitis** | **Ischemic Cardiomyopathy** |
| **sepsis 2** | **57** | **M** | **Septic Shock** | **Pneumonia** | **Diabetes Mellitus** |  |
| **sepsis 3** | **79** | **F** | **Septic Shock** | **Pneumonia** | **Pulmonar Emphysema** | **Chronic Cardiomyopathy** |
| **sepsis 4** | **67** | **M** | **Septic Shock** | **Lung Abscess** | **Pyomyositis** | **Atherosclerosis** |
| **sepsis 5** | **79** | **M** | **Septic Shock** | **Acute Abdomen** | **Perforated Duodenal Ulcer** | **Hypertrophic Cardiomyopathy** |
| **sepsis 6** | **56** | **M** | **Septic Shock** | **Purulent Meningitis** | **Cerebral Herniation** |  |
| **sepsis 7** | **69** | **M** | **Septic Shock** | **Pneumonia** | **Diabetes Mellitus** |  |
