# Supplementary material for: Transcriptome analysis of six tissues obtained post‐mortem from sepsis patients
Source: J Cell Mol Med. 2023 Sep 20;27(20):3157–67. doi: 10.1111/jcmm.17938 (PMC10568675; doi:10.1111/jcmm.17938)
Supplement: Supplementary file 2 — Table S2. [file JCMM-27-3157-s003.docx]

DEGs COLON

| gene_ID | metap | metafc | idx | fdr |
| --- | --- | --- | --- | --- |
| NACC1 | 1.12E-05 | 5.585679102 | 27.66110146 | 0.000152878 |
| DNAJB9 | 6.13E-05 | 5.478753919 | 23.0775438 | 0.000620109 |
| AC011446.1 | 1.12E-05 | 5.477558418 | 27.12567199 | 0.000152878 |
| PATZ1 | 9.70E-06 | 5.473692133 | 27.44063834 | 0.000135509 |
| AMZ2P1 | 2.67E-06 | 5.439025821 | 30.315998 | 4.56E-05 |
| KLHL22 | 1.88E-05 | 5.325220323 | 25.16632416 | 0.000233514 |
| ZNF282 | 1.51E-07 | 5.293778011 | 36.11121021 | 3.93E-06 |
| CCDC74BP1 | 5.47E-06 | 5.285677619 | 27.8113959 | 8.29E-05 |
| AC098484.3 | 2.41E-05 | 5.277271754 | 24.37390273 | 0.000286778 |
| VWA1 | 2.02E-18 | 5.242972805 | 92.77557609 | 9.00E-16 |
| EMC9 | 1.06E-08 | 5.224570261 | 41.65824244 | 4.04E-07 |
| PRDX4 | 0.000143574 | 5.199391082 | 19.98086451 | 0.001253104 |
| ARMCX2 | 4.42E-10 | 5.1718317 | 48.37822887 | 2.63E-08 |
| GADD45G | 7.71E-08 | 5.164891314 | 36.73865862 | 2.23E-06 |
| TIMM23 | 2.32E-13 | 5.156296725 | 65.14552166 | 3.79E-11 |
| MTLN | 2.75E-05 | 5.072338318 | 23.13140912 | 0.000320546 |
| SNAPC2 | 8.95E-09 | 5.069436253 | 40.79869546 | 3.49E-07 |
| TOLLIP | 0.000102985 | 5.069436253 | 20.21298062 | 0.000949137 |
| CLGN | 0.000198686 | 5.033341051 | 18.63258646 | 0.00164091 |
| NEK6 | 9.99E-07 | 5.026915831 | 30.16451399 | 2.03E-05 |
| SLC43A1 | 0.000548365 | 4.962369154 | 16.18194 | 0.003719159 |
| PDPN | 4.46E-09 | 4.912256392 | 41.02131586 | 1.91E-07 |
| F2R | 0.000757209 | 4.909877557 | 15.32266888 | 0.004829286 |
| NECTIN2 | 0.000548365 | 4.903443913 | 15.9897889 | 0.003719159 |
| GPAA1 | 1.34E-07 | 4.836244561 | 33.24553408 | 3.55E-06 |
| NR2C2AP | 5.77E-05 | 4.814969502 | 20.40913697 | 0.000588942 |
| AUH | 7.84E-05 | 4.814969502 | 19.77006946 | 0.000759945 |
| C1orf54 | 0.0002405 | 4.814969502 | 17.42482452 | 0.00191114 |
| DCTPP1 | 0.000995458 | 4.813406638 | 14.4497366 | 0.006031129 |
| AL049795.2 | 0.000803521 | 4.774405419 | 14.77679709 | 0.00505588 |
| AC137695.3 | 6.53E-08 | 4.77270457 | 34.29337101 | 1.92E-06 |
| PRMT5 | 4.76E-07 | 4.77270457 | 30.17433236 | 1.06E-05 |
| PRPS1 | 1.09E-05 | 4.77270457 | 23.68216978 | 0.000150052 |
| SECTM1 | 9.33E-07 | 4.771311292 | 28.77215996 | 1.92E-05 |
| GMPSP1 | 1.13E-06 | 4.766357034 | 28.34393017 | 2.22E-05 |
| CFB | 2.16E-15 | 4.763196898 | 69.85202776 | 5.27E-13 |
| CDK5 | 0.000292109 | 4.747493199 | 16.77979869 | 0.002239505 |
| AC004224.2 | 7.80E-08 | 4.72708832 | 33.60064758 | 2.25E-06 |
| AC008937.3 | 3.60E-07 | 4.72708832 | 30.45790775 | 8.36E-06 |
| TMEM11 | 5.01E-08 | 4.657007454 | 33.99625025 | 1.54E-06 |
| AC068724.2 | 0.000447877 | 4.616443279 | 15.45973466 | 0.00316158 |
| FPGS | 7.32E-05 | 4.599897444 | 19.02309279 | 0.000721835 |
| NDUFB4P12 | 0.000110235 | 4.556652084 | 18.0337751 | 0.001006816 |
| MT1F | 3.47E-08 | 4.546645991 | 33.91718747 | 1.11E-06 |
| WDR81 | 0.001105638 | 4.513261634 | 13.34294768 | 0.006529908 |
| BAD | 6.21E-05 | 4.51310158 | 18.98599877 | 0.00062603 |
| RPL22P12 | 0.000185817 | 4.51310158 | 16.83799185 | 0.001551448 |
| GLIS2 | 0.000793352 | 4.51310158 | 13.99302475 | 0.005007402 |

| FCGR3A | 7.42E-05 | 4.510826085 | 18.62891561 | 0.000729837 |
| --- | --- | --- | --- | --- |
| C7orf25 | 0.000793352 | 4.505798237 | 13.97038049 | 0.005007402 |
| TBKBP1 | 0.001365629 | 4.503300316 | 12.90045636 | 0.00773321 |
| AC010327.6 | 3.67E-08 | 4.484854536 | 33.34633653 | 1.17E-06 |
| AC087292.2 | 0.000113773 | 4.480049261 | 17.66913023 | 0.001036546 |
| ZNF671 | 0.001105638 | 4.47272458 | 13.22310446 | 0.006529908 |
| ABTB1 | 9.59E-06 | 4.463910834 | 22.39979143 | 0.000134228 |
| AC016588.1 | 0.001078163 | 4.463910834 | 13.24583182 | 0.006416884 |
| PLOD1 | 5.68E-26 | 4.445262656 | 112.2241155 | 7.42E-23 |
| MYORG | 1.74E-07 | 4.442239591 | 30.02838434 | 4.48E-06 |
| TMEM91 | 4.04E-05 | 4.442239591 | 19.51520698 | 0.000438668 |
| ZNF436-AS1 | 0.000447877 | 4.421598332 | 14.8072299 | 0.00316158 |
| RHBDF1 | 0.000803521 | 4.418401526 | 13.67496412 | 0.00505588 |
| DEFA1 | 1.01E-08 | 4.41572646 | 35.3146283 | 3.84E-07 |
| DEFA1B | 1.01E-08 | 4.41572646 | 35.3146283 | 3.84E-07 |
| CCDC71L | 4.98E-12 | 4.399736334 | 49.73014528 | 5.83E-10 |
| CEBPB | 1.77E-08 | 4.362840428 | 33.82189782 | 6.21E-07 |
| TTYH3 | 0.001432169 | 4.361293112 | 12.40354288 | 0.00802796 |
| BORA | 0.001205283 | 4.351903775 | 12.70281896 | 0.006994163 |
| ENDOG | 0.000183096 | 4.346369013 | 16.24377573 | 0.001532886 |
| TPRA1 | 0.000803521 | 4.334381379 | 13.41492155 | 0.00505588 |
| KCTD17 | 1.23E-07 | 4.330095084 | 29.9216769 | 3.30E-06 |
| HPR | 0.000216132 | 4.330095084 | 15.8710127 | 0.001752748 |
| APEH | 0.000164186 | 4.312438096 | 16.32113416 | 0.001400902 |
| MT1M | 1.81E-06 | 4.299566717 | 24.69048085 | 3.30E-05 |
| ENOPH1 | 0.000963949 | 4.243775684 | 12.79899775 | 0.005859644 |
| UBL4A | 2.96E-08 | 4.185583083 | 31.51325176 | 9.69E-07 |
| PRG4 | 0.000613414 | 4.17767631 | 13.41972508 | 0.004086449 |
| METRN | 0.000191683 | 4.167475279 | 15.49224309 | 0.001593145 |
| AGPAT2 | 0.001018817 | 4.146829713 | 12.40691642 | 0.006139681 |
| FOXP4 | 2.23E-06 | 4.111774612 | 23.23747937 | 3.93E-05 |
| ANKRD65 | 8.87E-05 | 4.111774612 | 16.6613371 | 0.000842058 |
| AC138150.1 | 0.000482397 | 4.111774612 | 13.63709372 | 0.003359771 |
| RABIF | 0.001365629 | 4.081745388 | 11.69284182 | 0.00773321 |
| AC068831.5 | 3.61E-05 | 4.070501654 | 18.08171525 | 0.000398693 |
| AC015922.3 | 7.39E-05 | 4.070501654 | 16.81616712 | 0.000727873 |
| AC139530.1 | 0.000126328 | 4.070501654 | 15.86885591 | 0.001130643 |
| IMPDH1 | 0.000388892 | 4.070501654 | 13.88110458 | 0.002834156 |
| STMN3 | 0.000749486 | 4.070501654 | 12.72128032 | 0.004798442 |
| DLAT | 2.04E-07 | 4.03558197 | 26.99682511 | 5.08E-06 |
| PDCD5 | 6.42E-05 | 4.015927077 | 16.83548666 | 0.000643654 |
| SH3BP5L | 0.000121521 | 4.015927077 | 15.72374867 | 0.00109513 |
| SNX8 | 0.001045338 | 4.015927077 | 11.97044833 | 0.006262778 |
| AL031736.2 | 2.74E-08 | 3.959389327 | 29.94410306 | 9.08E-07 |
| RAI2 | 2.51E-06 | 3.959389327 | 22.17587191 | 4.33E-05 |
| AC005606.1 | 0.00124098 | 3.959389327 | 11.50691726 | 0.007158288 |
| TEX14 | 6.03E-05 | 3.958238171 | 16.70204768 | 0.000611353 |
| UBALD1 | 2.11E-06 | 3.946166229 | 22.39600579 | 3.76E-05 |
| OSBPL5 | 9.87E-08 | 3.87694778 | 27.16075119 | 2.74E-06 |
| PNPLA2 | 3.24E-06 | 3.872739059 | 21.25972296 | 5.38E-05 |
| RPL36AP13 | 2.32E-05 | 3.815681812 | 17.6837631 | 0.000278 |
| PRR13P2 | 0.001225406 | 3.805425723 | 11.08033375 | 0.007088439 |

| SPP1 | 0.000807852 | 3.797307207 | 11.74381109 | 0.005077888 |
| --- | --- | --- | --- | --- |
| SLC12A7 | 3.60E-06 | 3.780557847 | 20.57922947 | 5.88E-05 |
| ATAD3A | 0.000912872 | 3.780557847 | 11.49134597 | 0.005606942 |
| SHARPIN | 1.52E-08 | 3.768557206 | 29.46630593 | 5.48E-07 |
| HS1BP3 | 1.96E-07 | 3.768557206 | 25.2812876 | 4.91E-06 |
| ZNF34 | 0.000117302 | 3.736779716 | 14.6881461 | 0.001064446 |
| SUMO4 | 0.000216073 | 3.723966877 | 13.64982903 | 0.001752748 |
| CEP170B | 1.88E-09 | 3.693846899 | 32.22811679 | 9.26E-08 |
| DNAJC4 | 0.001087678 | 3.689684012 | 10.9343768 | 0.006457207 |
| PLPP1 | 1.21E-20 | 3.646217599 | 72.62038757 | 7.64E-18 |
| AL158847.1 | 1.49E-06 | 3.627481386 | 21.13954018 | 2.79E-05 |
| MRPL53 | 2.51E-06 | 3.620620945 | 20.27778225 | 4.33E-05 |
| CDK16 | 4.10E-08 | 3.616149917 | 26.71342247 | 1.29E-06 |
| P4HA2-AS1 | 1.46E-06 | 3.586567273 | 20.93060785 | 2.74E-05 |
| HS6ST3 | 4.77E-06 | 3.580217573 | 19.05103273 | 7.40E-05 |
| CHCHD4 | 8.96E-08 | 3.56794332 | 25.14543041 | 2.52E-06 |
| MFSD5 | 2.44E-05 | 3.56794332 | 16.46034404 | 0.000288997 |
| GNAZ | 0.000137222 | 3.56794332 | 13.78144828 | 0.0012066 |
| SNORD38C | 0.000435905 | 3.56794332 | 11.99045943 | 0.003101074 |
| CCDC181 | 0.000537246 | 3.56794332 | 11.66655609 | 0.003664791 |
| FZD5 | 0.001667788 | 3.56794332 | 9.911243711 | 0.009041064 |
| PTPMT1 | 2.57E-08 | 3.559887297 | 27.02215105 | 8.59E-07 |
| ADO | 0.00032262 | 3.547895016 | 12.38679584 | 0.002428617 |
| AC011477.2 | 0.000333771 | 3.530641349 | 12.27445658 | 0.002496115 |
| AXIN1 | 0.0006567 | 3.527846712 | 11.22784182 | 0.004316231 |
| AC126615.1 | 0.001664589 | 3.527846712 | 9.802802444 | 0.009026399 |
| CNPPD1 | 1.77E-05 | 3.489192999 | 16.57720542 | 0.000222497 |
| AC093663.2 | 1.66E-08 | 3.479393114 | 27.068217 | 5.94E-07 |
| AC005253.2 | 2.07E-08 | 3.460139088 | 26.58441595 | 7.13E-07 |
| AP000766.1 | 7.74E-09 | 3.388276855 | 27.48335053 | 3.09E-07 |
| MYDGF | 4.01E-07 | 3.379786623 | 21.61864777 | 9.16E-06 |
| ALG3 | 1.39E-07 | 3.378970692 | 23.17034367 | 3.65E-06 |
| AC004542.2 | 1.41E-05 | 3.361743975 | 16.30430797 | 0.000184506 |
| AC091231.2 | 8.70E-12 | 3.352089659 | 37.07547815 | 9.41E-10 |
| AC079150.1 | 1.50E-06 | 3.340163537 | 19.45143037 | 2.81E-05 |
| TCAF2P1 | 2.57E-14 | 3.325831464 | 45.2003792 | 5.18E-12 |
| MRPL22 | 2.50E-06 | 3.318291183 | 18.58793486 | 4.33E-05 |
| ZBTB33 | 5.33E-06 | 3.306811501 | 17.43690125 | 8.13E-05 |
| ASPN | 2.19E-06 | 3.300111773 | 18.67532543 | 3.88E-05 |
| FAM104A | 2.17E-08 | 3.299412875 | 25.2849902 | 7.38E-07 |
| EIF3FP3 | 7.63E-05 | 3.295100772 | 13.56678977 | 0.000746436 |
| ID3 | 4.94E-06 | 3.288559826 | 17.44888963 | 7.62E-05 |
| AC010460.4 | 0.001134206 | 3.288559826 | 9.68582215 | 0.00665567 |
| DEFA3 | 3.05E-06 | 3.280756362 | 18.09726351 | 5.10E-05 |
| AC008533.1 | 0.001155126 | 3.280756362 | 9.636797129 | 0.006758875 |
| AL109925.5 | 5.00E-05 | 3.277800748 | 14.09752711 | 0.000521381 |
| AL731563.3 | 3.97E-10 | 3.250256481 | 30.55649867 | 2.40E-08 |
| B9D2 | 0.000636887 | 3.249040904 | 10.3837323 | 0.004219376 |
| AL133410.3 | 2.00E-08 | 3.248380265 | 25.00985354 | 6.95E-07 |
| HINT2 | 2.00E-08 | 3.248380265 | 25.00985354 | 6.95E-07 |
| AL445685.3 | 0.000710281 | 3.224919441 | 10.15388386 | 0.004592464 |
| PIP4P2 | 2.47E-09 | 3.22374886 | 27.74910429 | 1.17E-07 |

| FAM177B | 1.02E-21 | 3.1526997 | 66.18040676 | 7.17E-19 |
| --- | --- | --- | --- | --- |
| NR4A3 | 2.17E-05 | 3.150281701 | 14.69244012 | 0.000263107 |
| NFIL3 | 9.58E-23 | 3.143148188 | 69.20764939 | 7.62E-20 |
| RNF139 | 2.34E-06 | 3.142855702 | 17.69942162 | 4.10E-05 |
| TST | 1.74E-08 | 3.113434351 | 24.15991431 | 6.13E-07 |
| FBRS | 6.83E-08 | 3.092811527 | 22.1622659 | 1.99E-06 |
| GYS1 | 0.000128222 | 3.089624635 | 12.02493145 | 0.001143681 |
| S100A8 | 2.32E-20 | 3.088547103 | 60.64188783 | 1.37E-17 |
| TRIAP1 | 2.16E-12 | 3.059394693 | 35.69095552 | 2.74E-10 |
| RPA1 | 8.10E-08 | 3.05260024 | 21.64683273 | 2.32E-06 |
| MRPL49 | 0.000186479 | 3.05260024 | 11.38427517 | 0.001554843 |
| PHF5A | 0.00099382 | 3.05260024 | 9.166019056 | 0.006025199 |
| AC011462.1 | 0.00181537 | 3.038504644 | 8.328646761 | 0.009683212 |
| AC068946.2 | 1.55E-09 | 3.00597923 | 26.47972503 | 7.83E-08 |
| STK32B | 0.000647606 | 2.978640738 | 9.497959846 | 0.004268735 |
| CD14 | 0.00015395 | 2.978498715 | 11.35588119 | 0.001325946 |
| KIAA1522 | 0.000179208 | 2.977244196 | 11.15466854 | 0.001507008 |
| CKS1B | 0.000464229 | 2.964650902 | 9.881975714 | 0.003254764 |
| CHPF | 1.23E-07 | 2.951029382 | 20.39317082 | 3.30E-06 |
| ZNF253 | 0.00142034 | 2.941983767 | 8.377615617 | 0.007981228 |
| IL1B | 9.73E-07 | 2.898838049 | 17.42720644 | 1.99E-05 |
| TXNDC9 | 5.45E-06 | 2.881895561 | 15.17028471 | 8.25E-05 |
| TSTD1 | 0.000202309 | 2.874646391 | 10.61889989 | 0.001661824 |
| ZNF384 | 0.000417488 | 2.872112472 | 9.70588932 | 0.002998341 |
| REPIN1 | 7.07E-07 | 2.86908257 | 17.64576671 | 1.49E-05 |
| ZNF768 | 4.85E-11 | 2.867814282 | 29.57885649 | 4.22E-09 |
| TBPL1 | 1.96E-05 | 2.862926888 | 13.47549337 | 0.000241323 |
| ABT1 | 8.45E-05 | 2.853801627 | 11.62421576 | 0.000808766 |
| AL590133.2 | 5.28E-05 | 2.848535786 | 12.18458994 | 0.000545909 |
| ATP6V1C2 | 1.84E-10 | 2.831024194 | 27.56108918 | 1.27E-08 |
| PCDHGA4 | 0.000200924 | 2.828890242 | 10.45832027 | 0.00165267 |
| LRATD2 | 0.000687509 | 2.822939038 | 8.92817076 | 0.004477592 |
| AC005632.3 | 0.000410736 | 2.80066331 | 9.484271863 | 0.002960396 |
| DUSP23 | 9.53E-06 | 2.79810596 | 14.04856286 | 0.000133683 |
| DDIT4 | 0.00017453 | 2.7941911 | 10.50093342 | 0.001475395 |
| NKX6-2 | 5.69E-16 | 2.78899337 | 42.51857334 | 1.60E-13 |
| PTPRU | 9.06E-05 | 2.770956232 | 11.20256068 | 0.000858396 |
| AC104794.5 | 0.000384678 | 2.769892792 | 9.458914158 | 0.002810163 |
| GCOM2 | 0.001127059 | 2.769181353 | 8.163693872 | 0.006624327 |
| KAZN-AS1 | 0.000304109 | 2.76458539 | 9.722965733 | 0.002315966 |
| POLR2K | 5.34E-08 | 2.747382811 | 19.97949892 | 1.63E-06 |
| FAM199X | 6.91E-06 | 2.74545672 | 14.16798243 | 0.000101242 |
| MKRN2 | 0.000172058 | 2.74545672 | 10.33479144 | 0.001457194 |
| MAOB | 1.15E-06 | 2.735129909 | 16.24362261 | 2.25E-05 |
| SLC35A4 | 5.76E-08 | 2.722414999 | 19.70819364 | 1.72E-06 |
| AC010442.1 | 7.67E-05 | 2.722230439 | 11.2030729 | 0.000748437 |
| PHF2P2 | 0.000105395 | 2.720670191 | 10.82059726 | 0.000966471 |
| NCDN | 0.000145067 | 2.715352789 | 10.42269527 | 0.001260718 |
| PPP6R1 | 2.11E-06 | 2.714829053 | 15.40945615 | 3.76E-05 |
| PCDHGA7 | 9.09E-05 | 2.701784612 | 10.91947766 | 0.000859574 |
| PCDHGA12 | 0.000200924 | 2.701784612 | 9.988414662 | 0.00165267 |
| PCDHGA2 | 0.000200924 | 2.701784612 | 9.988414662 | 0.00165267 |

| PCDHGB1 | 0.000200924 | 2.701784612 | 9.988414662 | 0.00165267 |
| --- | --- | --- | --- | --- |
| PCDHGB3 | 0.000200924 | 2.701784612 | 9.988414662 | 0.00165267 |
| PCDHGB5 | 0.000200924 | 2.701784612 | 9.988414662 | 0.00165267 |
| PCDHGC5 | 0.000200924 | 2.701784612 | 9.988414662 | 0.00165267 |
| IMP4 | 8.13E-09 | 2.696357442 | 21.81355515 | 3.23E-07 |
| CU633906.2 | 0.000215393 | 2.689478182 | 9.861693319 | 0.001750428 |
| CU634019.2 | 0.000215393 | 2.689478182 | 9.861693319 | 0.001750428 |
| SLC2A12 | 0.000253125 | 2.686112239 | 9.661046259 | 0.001988984 |
| PYGO2 | 1.78E-05 | 2.684736857 | 12.75379731 | 0.000222616 |
| SEC22A | 2.99E-06 | 2.683122155 | 14.82167869 | 5.02E-05 |
| RPL10P15 | 3.35E-08 | 2.681497864 | 20.04254114 | 1.09E-06 |
| KLHDC3 | 4.12E-12 | 2.68019242 | 30.51555342 | 4.95E-10 |
| MASP1 | 1.90E-10 | 2.674807643 | 26.00280186 | 1.31E-08 |
| KLHL15 | 0.000338564 | 2.674453995 | 9.28131719 | 0.002521644 |
| ZFAND1 | 6.83E-07 | 2.652662011 | 16.35452257 | 1.46E-05 |
| FOXO4 | 5.44E-05 | 2.647798633 | 11.29220863 | 0.000558997 |
| CU633904.2 | 0.000365361 | 2.633648199 | 9.052579599 | 0.002688383 |
| COMT | 2.58E-05 | 2.627562722 | 12.05463705 | 0.000304005 |
| LAMTOR1 | 0.000119283 | 2.623843045 | 10.29444311 | 0.001079212 |
| NUDT16 | 1.52E-06 | 2.619257975 | 15.24231765 | 2.83E-05 |
| FRZB | 1.50E-05 | 2.60725954 | 12.58066427 | 0.00019283 |
| KBTBD11 | 0.001516487 | 2.605178143 | 7.344417529 | 0.00837733 |
| AC004771.3 | 0.00044108 | 2.604360643 | 8.738886012 | 0.003125965 |
| HLA-DRB1 | 1.17E-07 | 2.602189678 | 18.03908507 | 3.16E-06 |
| RNU6-437P | 8.71E-06 | 2.584266025 | 13.07614493 | 0.000123865 |
| WFDC2 | 3.47E-10 | 2.580692247 | 24.41265171 | 2.17E-08 |
| AL645922.1 | 1.13E-12 | 2.577168471 | 30.78960396 | 1.55E-10 |
| PDZD11 | 1.19E-10 | 2.567924788 | 25.48984417 | 8.84E-09 |
| PCDHGC3 | 1.29E-05 | 2.565415678 | 12.5464421 | 0.000171459 |
| FN3KRP | 0.00071876 | 2.550848675 | 8.018378328 | 0.004639087 |
| AC012085.2 | 3.82E-05 | 2.548699357 | 11.26111794 | 0.000418834 |
| FAM20C | 0.000668186 | 2.548357458 | 8.09129565 | 0.004374432 |
| GIHCG | 0.00052315 | 2.545931995 | 8.354154028 | 0.003585452 |
| DDIT4-AS1 | 0.001499614 | 2.545493579 | 7.188526082 | 0.008321834 |
| AC005841.1 | 7.96E-10 | 2.518304081 | 22.91404514 | 4.36E-08 |
| IDH3B | 5.69E-08 | 2.517368455 | 18.23838666 | 1.71E-06 |
| SMIM3 | 5.23E-07 | 2.504496178 | 15.73205619 | 1.15E-05 |
| IKZF4 | 2.18E-05 | 2.500494672 | 11.6568227 | 0.000264172 |
| RNASE4 | 0.001011995 | 2.495441087 | 7.47340045 | 0.006109058 |
| SLC38A10 | 5.96E-08 | 2.482476263 | 17.93610955 | 1.77E-06 |
| HS2ST1 | 7.35E-06 | 2.469371072 | 12.67735107 | 0.000107057 |
| OSGIN2 | 3.39E-07 | 2.465033294 | 15.94928606 | 7.95E-06 |
| MFSD12 | 0.000354901 | 2.44102416 | 8.421271746 | 0.002626883 |
| RCC1L | 2.16E-09 | 2.440171683 | 21.14490771 | 1.04E-07 |
| ULK1 | 4.10E-07 | 2.440171683 | 15.58664685 | 9.29E-06 |
| C8orf44 | 5.53E-05 | 2.436750214 | 10.37418005 | 0.000566652 |
| AC104563.1 | 2.27E-05 | 2.422082436 | 11.24771845 | 0.000273505 |
| SSR4 | 2.49E-09 | 2.413218667 | 20.76491919 | 1.17E-07 |
| PSMB8 | 0.000188061 | 2.412911788 | 8.989788387 | 0.001566604 |
| NFYC-AS1 | 0.000950363 | 2.409595419 | 7.282064087 | 0.005798258 |
| AC011448.1 | 6.38E-05 | 2.406710957 | 10.09661635 | 0.000639905 |
| FBXO33 | 2.07E-08 | 2.399957419 | 18.43938811 | 7.13E-07 |

| AL354733.3 | 0.0011073 | 2.387046649 | 7.055476883 | 0.006535499 |
| --- | --- | --- | --- | --- |
| AL355075.3 | 0.000540015 | 2.383900508 | 7.789618704 | 0.003679549 |
| TRAPPC1 | 5.98E-11 | 2.382542537 | 24.35747613 | 4.96E-09 |
| ZNF132 | 0.001496242 | 2.376776168 | 6.714388603 | 0.008313211 |
| SDF2L1 | 8.44E-08 | 2.363061519 | 16.71592131 | 2.40E-06 |
| TMEM54 | 1.63E-10 | 2.362384797 | 23.12032251 | 1.16E-08 |
| AC016747.4 | 7.19E-09 | 2.360294521 | 19.22021599 | 2.91E-07 |
| EIF5AP2 | 1.79E-07 | 2.354985134 | 15.88721064 | 4.59E-06 |
| GSTT2 | 0.000183175 | 2.352692523 | 8.792326678 | 0.001532886 |
| GSTT2B | 0.000183175 | 2.352692523 | 8.792326678 | 0.001532886 |
| RNF5 | 0.00059659 | 2.351871306 | 7.583194589 | 0.003985625 |
| NEDD1 | 2.78E-06 | 2.350968983 | 13.06075194 | 4.71E-05 |
| PCDHGA1 | 0.00012558 | 2.350968983 | 9.171316188 | 0.001124501 |
| PCDHGA11 | 0.000817354 | 2.350968983 | 7.258827586 | 0.00512704 |
| ALKBH7 | 0.00044108 | 2.349893892 | 7.885027336 | 0.003125965 |
| TP53INP1 | 3.03E-16 | 2.34589377 | 36.40571986 | 9.23E-14 |
| NPC2 | 7.16E-15 | 2.34402254 | 33.15645892 | 1.56E-12 |
| AC135983.4 | 0.000512106 | 2.329615835 | 7.665928226 | 0.003525546 |
| SLC9A9 | 0.001129442 | 2.323023827 | 6.846266942 | 0.006631938 |
| ARMCX5 | 0.000231437 | 2.321547781 | 8.440144388 | 0.00185119 |
| ZNF124 | 1.60E-06 | 2.321166639 | 13.45236785 | 2.98E-05 |
| FAM89B | 4.27E-06 | 2.316570517 | 12.43958922 | 6.80E-05 |
| SRF | 8.31E-08 | 2.3131758 | 16.37862152 | 2.37E-06 |
| MTG1 | 0.001377725 | 2.311302571 | 6.612261038 | 0.007787267 |
| GSK3A | 0.000135758 | 2.296362012 | 8.880572486 | 0.001196881 |
| AIP | 2.92E-06 | 2.295302068 | 12.7039926 | 4.91E-05 |
| BBS9 | 9.96E-06 | 2.294488096 | 11.47602204 | 0.000138762 |
| GRIN3B | 0.000217745 | 2.293824381 | 8.400102397 | 0.001764837 |
| SYVN1 | 0.000359514 | 2.292885979 | 7.897350687 | 0.002653902 |
| AC114546.3 | 5.66E-11 | 2.292350457 | 23.48986322 | 4.75E-09 |
| OPA1-AS1 | 5.05E-06 | 2.28512629 | 12.10278667 | 7.75E-05 |
| RBIS | 4.98E-10 | 2.280958985 | 21.21902981 | 2.94E-08 |
| TRAFD1 | 1.56E-05 | 2.27548695 | 10.93715679 | 0.000200059 |
| ABHD14B | 0.000284198 | 2.274183925 | 8.06511639 | 0.002188029 |
| XBP1 | 2.96E-09 | 2.273315869 | 19.38923328 | 1.36E-07 |
| MANBAL | 1.04E-11 | 2.267034908 | 24.90345925 | 1.08E-09 |
| SERPINE1 | 4.34E-16 | 2.266659179 | 34.82241852 | 1.26E-13 |
| TYROBP | 2.59E-07 | 2.266659179 | 14.93101576 | 6.26E-06 |
| EARS2 | 6.63E-05 | 2.266431861 | 9.470133869 | 0.000661454 |
| ACKR1 | 1.73E-05 | 2.265952145 | 10.78948518 | 0.000218052 |
| HIRIP3 | 0.001561491 | 2.255923339 | 6.331160011 | 0.008579284 |
| SNHG19 | 1.21E-06 | 2.249361469 | 13.30694503 | 2.34E-05 |
| AC013643.2 | 0.001361614 | 2.246796992 | 6.439198633 | 0.007721886 |
| COPE | 0.000572652 | 2.241464805 | 7.267072941 | 0.003862394 |
| BCL2A1 | 0.001122024 | 2.232157974 | 6.58486095 | 0.006605349 |
| AC008124.1 | 0.000418103 | 2.229471431 | 7.532753329 | 0.003001573 |
| ZBTB16 | 2.83E-27 | 2.225202127 | 59.07390776 | 4.71E-24 |
| PPP1R1A | 7.61E-07 | 2.221922517 | 13.59539945 | 1.60E-05 |
| RANBP17 | 6.91E-07 | 2.218632051 | 13.66814534 | 1.47E-05 |
| AC011558.1 | 5.51E-13 | 2.212441563 | 27.1217977 | 8.19E-11 |
| NNMT | 7.57E-28 | 2.210829491 | 59.95911971 | 1.39E-24 |
| GPR183 | 1.28E-06 | 2.202745942 | 12.98362513 | 2.44E-05 |

| AC114947.2 | 2.34E-06 | 2.199003303 | 12.38303317 | 4.10E-05 |
| --- | --- | --- | --- | --- |
| SRP9 | 6.67E-07 | 2.197640523 | 13.57174116 | 1.43E-05 |
| RAB5A | 5.44E-14 | 2.188283615 | 29.02708952 | 1.04E-11 |
| AP002982.1 | 1.20E-05 | 2.179662471 | 10.72814855 | 0.000161878 |
| SOD2 | 3.04E-29 | 2.17520435 | 62.02985708 | 6.18E-26 |
| MIR125B1 | 3.74E-08 | 2.174064788 | 16.14731757 | 1.19E-06 |
| FPR1 | 2.86E-05 | 2.168769976 | 9.853238967 | 0.000331283 |
| MARCHF8 | 1.85E-05 | 2.167860948 | 10.26223178 | 0.000230384 |
| AC098583.1 | 0.001338631 | 2.163715044 | 6.217087003 | 0.007613334 |
| CAT | 4.70E-11 | 2.159847648 | 22.30769163 | 4.12E-09 |
| GSTM4 | 0.000100538 | 2.15892127 | 8.630650235 | 0.000929396 |
| HPCA | 7.44E-10 | 2.157680287 | 19.69641338 | 4.12E-08 |
| NRBF2P3 | 0.000832935 | 2.153022697 | 6.629994247 | 0.005204202 |
| AL049839.2 | 0.000447766 | 2.150399448 | 7.201578486 | 0.00316158 |
| SERPINA3 | 0.000984187 | 2.150399448 | 6.46608443 | 0.005972737 |
| ABHD13 | 5.49E-05 | 2.134886646 | 9.094872452 | 0.000564042 |
| AC009630.1 | 5.46E-10 | 2.134785319 | 19.77330557 | 3.21E-08 |
| ELL2P1 | 0.000884126 | 2.131960627 | 6.50991195 | 0.005457879 |
| ATE1-AS1 | 1.32E-06 | 2.126356999 | 12.50321927 | 2.50E-05 |
| TMEM120A | 1.12E-05 | 2.124713735 | 10.51869922 | 0.000153222 |
| INPP5A | 5.27E-13 | 2.124161341 | 26.08065773 | 7.94E-11 |
| NAMPTP1 | 3.79E-31 | 2.120700355 | 64.51355668 | 1.39E-27 |
| AC007066.1 | 0.001702184 | 2.116399152 | 5.860295343 | 0.009181251 |
| HSPA5P1 | 4.52E-26 | 2.113643339 | 53.5690794 | 6.36E-23 |
| KCNAB3 | 0.000700855 | 2.112605858 | 6.663943901 | 0.004549216 |
| C1orf216 | 2.11E-08 | 2.111636742 | 16.21007106 | 7.20E-07 |
| OLFML3 | 2.98E-05 | 2.107717018 | 9.539446079 | 0.000342344 |
| GPN1 | 2.90E-05 | 2.103320249 | 9.545113947 | 0.000334366 |
| TM6SF1 | 0.000644442 | 2.099412112 | 6.698838333 | 0.004252479 |
| FH | 0.000544403 | 2.097679698 | 6.846993034 | 0.00369915 |
| LSM1 | 0.000103565 | 2.096812568 | 8.355352778 | 0.000953036 |
| GRINA | 9.18E-09 | 2.095010808 | 16.83802733 | 3.56E-07 |
| RPL29P11 | 1.12E-06 | 2.084309612 | 12.39956923 | 2.21E-05 |
| EBPL | 0.000954372 | 2.08179307 | 6.287602748 | 0.005814959 |
| LATS2 | 1.07E-07 | 2.081080589 | 14.50932814 | 2.92E-06 |
| AC084024.3 | 0.000932987 | 2.079720935 | 6.301813384 | 0.005707482 |
| ZNF169 | 2.33E-05 | 2.075032089 | 9.613099108 | 0.000278756 |
| AC105250.1 | 4.56E-24 | 2.074014826 | 48.40971204 | 4.63E-21 |
| MT1X | 1.26E-16 | 2.070851197 | 32.92511545 | 4.04E-14 |
| MIR3652 | 1.29E-13 | 2.067636514 | 26.64815919 | 2.25E-11 |
| AP003419.1 | 0.00021027 | 2.065422014 | 7.595016668 | 0.001717189 |
| FER1L4 | 7.41E-13 | 2.061488579 | 25.00635773 | 1.07E-10 |
| GOLGA7 | 1.05E-09 | 2.060231776 | 18.49976647 | 5.52E-08 |
| CCDC85B | 0.001735505 | 2.059897241 | 5.686498927 | 0.009322477 |
| LRRC8A | 6.93E-07 | 2.059006682 | 12.68136203 | 1.47E-05 |
| PLPPR2 | 0.000244604 | 2.055263695 | 7.422660671 | 0.001934936 |
| AL354794.1 | 9.58E-09 | 2.04929822 | 16.43213564 | 3.68E-07 |
| ERO1B | 6.61E-05 | 2.048474779 | 8.562211775 | 0.000659737 |
| AL671710.1 | 0.001536858 | 2.041038318 | 5.742188351 | 0.008459196 |
| UQCRC1 | 6.43E-10 | 2.040772217 | 18.7586561 | 3.64E-08 |
| RN7SL608P | 5.52E-08 | 2.0402712 | 14.80886119 | 1.66E-06 |
| AC079089.2 | 2.88E-07 | 2.038214559 | 13.33089767 | 6.89E-06 |

| SYS1 | 1.43E-13 | 2.031601188 | 26.09672103 | 2.46E-11 |
| --- | --- | --- | --- | --- |
| SLC9A3 | 0.000120401 | 2.029469654 | 7.954244329 | 0.001087174 |
| MZT2A | 5.38E-05 | 2.028646785 | 8.660721418 | 0.000554224 |
| NAMPT | 7.28E-30 | 2.026987988 | 59.0615803 | 1.66E-26 |
| AC125611.3 | 1.86E-05 | 2.025704887 | 9.581735331 | 0.000231739 |
| GOLGA8VP | 0.001182477 | 2.016908837 | 5.90390995 | 0.006883668 |
| RDH14 | 1.27E-07 | 2.013392338 | 13.88694984 | 3.39E-06 |
| SNAPIN | 2.73E-06 | 2.003531677 | 11.14575702 | 4.65E-05 |
| C2orf74 | 3.58E-07 | 1.989558076 | 12.82476739 | 8.33E-06 |
| NR1D2 | 5.75E-10 | 1.983891538 | 18.33171495 | 3.34E-08 |
| MRPS2 | 4.72E-06 | 1.983717513 | 10.5647072 | 7.36E-05 |
| PIGK | 0.00078349 | 1.980682125 | 6.151932789 | 0.004959265 |
| TFE3 | 2.61E-13 | 1.98025329 | 24.91746822 | 4.19E-11 |
| AC018362.1 | 0.00061347 | 1.977109319 | 6.350883564 | 0.004086449 |
| ZDHHC4 | 0.00049501 | 1.974902585 | 6.527815802 | 0.003428505 |
| AC092017.2 | 7.21E-09 | 1.974759655 | 16.07899339 | 2.91E-07 |
| NDUFA1 | 3.11E-13 | 1.971007143 | 24.65293254 | 4.85E-11 |
| CEBPD | 1.40E-19 | 1.966164069 | 37.06974248 | 7.53E-17 |
| LYSMD3 | 0.000182783 | 1.965552792 | 7.347361781 | 0.001532412 |
| UBXN6 | 1.95E-05 | 1.963254808 | 9.248926306 | 0.000239525 |
| SEC24A | 6.24E-06 | 1.959171759 | 10.19753234 | 9.26E-05 |
| RPA3 | 3.81E-05 | 1.951199405 | 8.622007466 | 0.000418655 |
| APOC1 | 0.000210987 | 1.949037141 | 7.164164149 | 0.001721503 |
| GLO1 | 2.51E-09 | 1.946878768 | 16.744647 | 1.18E-07 |
| SELENBP1 | 2.90E-14 | 1.942669722 | 26.2988214 | 5.64E-12 |
| SIGMAR1 | 0.000296171 | 1.940862908 | 6.848250823 | 0.002264003 |
| GPR180 | 0.000729319 | 1.940804804 | 6.088464448 | 0.0047006 |
| MRPL24 | 1.42E-06 | 1.940121918 | 11.34563955 | 2.67E-05 |
| NDUFB4 | 1.40E-07 | 1.937300297 | 13.27879622 | 3.67E-06 |
| MT1G | 2.84E-08 | 1.929818339 | 14.56305115 | 9.40E-07 |
| DEPP1 | 1.63E-08 | 1.929747525 | 15.0271666 | 5.85E-07 |
| USP5 | 6.16E-06 | 1.925226642 | 10.03084737 | 9.18E-05 |
| LDHA | 1.91E-30 | 1.921967146 | 57.11784723 | 5.00E-27 |
| ALG5 | 1.31E-05 | 1.918994573 | 9.371885142 | 0.000173168 |
| SQOR | 2.41E-12 | 1.914222277 | 22.24073377 | 2.99E-10 |
| LIN7C | 2.47E-08 | 1.910342326 | 14.53190242 | 8.31E-07 |
| C1orf52 | 1.01E-06 | 1.905725368 | 11.42255966 | 2.05E-05 |
| AL034380.1 | 3.26E-05 | 1.903687115 | 8.540475816 | 0.000368382 |
| BZW1P2 | 0.00096985 | 1.894733226 | 5.709391098 | 0.005891596 |
| RPS6KA2 | 3.00E-08 | 1.893575824 | 14.24643836 | 9.78E-07 |
| NOP14-AS1 | 1.56E-07 | 1.892799677 | 12.88364421 | 4.06E-06 |
| SCARA3 | 3.17E-05 | 1.884553949 | 8.478862281 | 0.000359637 |
| IFITM1 | 9.86E-12 | 1.884433175 | 20.74024196 | 1.04E-09 |
| MT1E | 9.43E-07 | 1.876946599 | 11.30981837 | 1.94E-05 |
| BZW1-AS1 | 0.00016967 | 1.876022029 | 7.073344163 | 0.001439636 |
| MED4-AS1 | 0.001313551 | 1.870775857 | 5.390739868 | 0.007486995 |
| XPOT | 0.000359181 | 1.869518751 | 6.439906361 | 0.002652514 |
| MIF4GD | 2.76E-07 | 1.865119748 | 12.2337261 | 6.65E-06 |
| AC009088.2 | 9.97E-07 | 1.862808393 | 11.17927276 | 2.03E-05 |
| NCBP2AS2 | 0.001312205 | 1.862483275 | 5.367673348 | 0.007481658 |
| EIF1P5 | 1.89E-05 | 1.859919551 | 8.7856134 | 0.000234552 |
| SMDT1 | 4.47E-06 | 1.856433819 | 9.931047881 | 7.04E-05 |

| CTSA | 6.61E-09 | 1.856327425 | 15.18443302 | 2.71E-07 |
| --- | --- | --- | --- | --- |
| RPL31P17 | 3.64E-09 | 1.855435297 | 15.65796392 | 1.62E-07 |
| MED23 | 5.61E-05 | 1.85489216 | 7.885440931 | 0.000573547 |
| FAM92A | 1.10E-06 | 1.844963767 | 10.99491068 | 2.17E-05 |
| AC005261.1 | 1.38E-12 | 1.843844186 | 21.86819806 | 1.86E-10 |
| DAG1 | 0.001054158 | 1.842241127 | 5.484525727 | 0.006307354 |
| AL136380.1 | 6.44E-08 | 1.839992935 | 13.23212277 | 1.89E-06 |
| QARS1 | 9.71E-11 | 1.83546453 | 18.37839811 | 7.43E-09 |
| ANGPTL4 | 0.000447085 | 1.835360095 | 6.147740745 | 0.00316001 |
| MED8 | 0.000103287 | 1.834125255 | 7.31073851 | 0.000951438 |
| RAP2A | 6.26E-05 | 1.83221926 | 7.701815829 | 0.000630483 |
| NCAM2 | 1.65E-05 | 1.831807017 | 8.762209414 | 0.00020925 |
| RPL9P3 | 1.60E-05 | 1.830056925 | 8.777082865 | 0.000204355 |
| AC099518.5 | 5.79E-07 | 1.820355796 | 11.35439889 | 1.26E-05 |
| PXN | 0.000405665 | 1.815008323 | 6.156204562 | 0.002933009 |
| MAT2B | 1.98E-05 | 1.812058881 | 8.520896867 | 0.000243545 |
| STK25 | 1.24E-05 | 1.808385732 | 8.872691916 | 0.000166168 |
| ALKBH4 | 4.07E-05 | 1.806716307 | 7.931455642 | 0.000441043 |
| AC079742.1 | 0.000600819 | 1.802573276 | 5.80655088 | 0.004012407 |
| ADM | 1.20E-05 | 1.802471468 | 8.86853798 | 0.000162161 |
| AL355802.1 | 7.68E-10 | 1.800872556 | 16.41382217 | 4.23E-08 |
| TMEM259 | 0.001704218 | 1.800838415 | 4.985575835 | 0.00918951 |
| BSCL2 | 4.99E-08 | 1.799495505 | 13.14030408 | 1.53E-06 |
| PLEKHO2 | 0.00031584 | 1.799495505 | 6.29919426 | 0.002385734 |
| NDUFA8 | 1.37E-09 | 1.797770932 | 15.93210315 | 7.04E-08 |
| H2AC18 | 1.36E-06 | 1.796558116 | 10.54114273 | 2.56E-05 |
| AL589765.7 | 0.001353324 | 1.796377517 | 5.153085275 | 0.007687335 |
| DEGS1 | 3.19E-05 | 1.791241764 | 8.052887117 | 0.000361371 |
| RABAC1 | 1.04E-05 | 1.790867328 | 8.925231639 | 0.000144132 |
| PSMB1 | 1.54E-12 | 1.787263936 | 21.11111631 | 2.03E-10 |
| HIGD1A | 4.29E-07 | 1.778744511 | 11.3264151 | 9.68E-06 |
| NDUFA12 | 0.00050422 | 1.776533365 | 5.857905215 | 0.003481747 |
| CCT4 | 1.61E-15 | 1.774880203 | 26.25446094 | 3.99E-13 |
| AC091053.1 | 8.54E-05 | 1.763837107 | 7.175878661 | 0.000815816 |
| ARMCX7P | 2.18E-06 | 1.76349847 | 9.984552465 | 3.86E-05 |
| ZBTB14 | 0.000414736 | 1.762762698 | 5.962065651 | 0.002980916 |
| ACAT2 | 1.05E-06 | 1.758541717 | 10.51675474 | 2.10E-05 |
| CDC26 | 0.000197845 | 1.756334967 | 6.504892867 | 0.001636183 |
| KXD1 | 0.000124574 | 1.755585103 | 6.854812315 | 0.001117678 |
| AC009094.1 | 0.001200695 | 1.750723549 | 5.113105983 | 0.006974176 |
| FLOT1 | 1.58E-11 | 1.750557067 | 18.90666773 | 1.57E-09 |
| WASF4P | 0.001525265 | 1.744532163 | 4.913744821 | 0.008408039 |
| HILPDA | 1.94E-18 | 1.742238832 | 30.85834666 | 8.87E-16 |
| CREBL2 | 1.92E-07 | 1.740179871 | 11.68856071 | 4.85E-06 |
| MYCBP | 0.001386043 | 1.736831445 | 4.964252072 | 0.007824575 |
| COIL | 1.99E-10 | 1.735287863 | 16.83611255 | 1.36E-08 |
| S100A11 | 2.83E-08 | 1.72149848 | 12.99412943 | 9.38E-07 |
| SRPX | 8.97E-08 | 1.717096733 | 12.10054457 | 2.52E-06 |
| GNA13 | 5.68E-09 | 1.704233622 | 14.05271196 | 2.37E-07 |
| DPP7 | 0.000197147 | 1.703944955 | 6.313472938 | 0.0016312 |
| RUSC1-AS1 | 1.45E-05 | 1.703382297 | 8.242228719 | 0.000188167 |
| AC107081.2 | 2.22E-12 | 1.700370464 | 19.81630597 | 2.78E-10 |

| UGCG | 9.92E-15 | 1.698656576 | 23.78720688 | 2.13E-12 |
| --- | --- | --- | --- | --- |
| SH2D7 | 7.69E-05 | 1.698116903 | 6.986235329 | 0.00074992 |
| AC090825.1 | 5.34E-05 | 1.696936273 | 7.250339214 | 0.00055084 |
| TSC22D3 | 4.92E-19 | 1.696091711 | 31.05215671 | 2.37E-16 |
| EIF2S2P2 | 0.000325865 | 1.693092295 | 5.903748837 | 0.002446999 |
| AL136131.3 | 0.000220325 | 1.692628136 | 6.189832386 | 0.001782587 |
| RFNG | 5.79E-05 | 1.687630141 | 7.151621129 | 0.000589989 |
| IFI27L2 | 9.70E-06 | 1.682374357 | 8.434117341 | 0.000135509 |
| AC020915.5 | 0.000952033 | 1.680068914 | 5.076072993 | 0.005805476 |
| NDUFB11 | 4.11E-11 | 1.677276155 | 17.4207462 | 3.68E-09 |
| AL161457.2 | 1.16E-08 | 1.675657645 | 13.29967257 | 4.34E-07 |
| SCMH1 | 4.26E-05 | 1.675384803 | 7.321685026 | 0.000457856 |
| RPL15P2 | 7.58E-05 | 1.673528938 | 6.895027551 | 0.000743243 |
| GAS5-AS1 | 0.000881369 | 1.673277685 | 5.111599083 | 0.00544454 |
| KLF10 | 2.69E-07 | 1.670988985 | 10.97901072 | 6.50E-06 |
| AC016831.4 | 3.61E-05 | 1.669277782 | 7.415389569 | 0.000398693 |
| AL450306.1 | 4.19E-06 | 1.663720952 | 8.946871798 | 6.70E-05 |
| ATP6V1G1 | 4.53E-11 | 1.662460794 | 17.19686825 | 4.00E-09 |
| AFG3L2P1 | 0.00047039 | 1.66106377 | 5.527259727 | 0.003289781 |
| AC092756.1 | 3.68E-07 | 1.654104119 | 10.64273741 | 8.49E-06 |
| PIKFYVE | 1.32E-06 | 1.653257172 | 9.720531674 | 2.50E-05 |
| AC087393.2 | 4.71E-11 | 1.65303969 | 17.07084768 | 4.12E-09 |
| RAB8B | 5.19E-06 | 1.651501435 | 8.727653991 | 7.94E-05 |
| DTX3 | 0.000293796 | 1.649497633 | 5.825949583 | 0.002249608 |
| NAP1L2 | 7.62E-05 | 1.647014693 | 6.7820216 | 0.000745967 |
| RBM18 | 0.000463258 | 1.637039625 | 5.458179263 | 0.003251701 |
| PLPBP | 6.96E-07 | 1.636230164 | 10.07453901 | 1.48E-05 |
| NUDCD1 | 2.11E-08 | 1.636037646 | 12.55930236 | 7.20E-07 |
| HSPA5 | 3.39E-19 | 1.633161182 | 30.16425054 | 1.72E-16 |
| NBL1 | 9.27E-18 | 1.631950173 | 27.79716317 | 3.85E-15 |
| AL031133.1 | 3.86E-05 | 1.628222549 | 7.185933715 | 0.00042218 |
| MRPL18 | 0.000131021 | 1.621556885 | 6.2959507 | 0.001163538 |
| MXD3 | 0.000889024 | 1.618744872 | 4.938930642 | 0.005484414 |
| AC069218.1 | 0.001159133 | 1.61860548 | 4.752009936 | 0.006775815 |
| EIF1AXP1 | 5.24E-12 | 1.617425903 | 18.24546429 | 5.95E-10 |
| PLSCR1 | 1.95E-06 | 1.616231621 | 9.227229646 | 3.53E-05 |
| AL137129.1 | 0.000304537 | 1.612548614 | 5.670301388 | 0.002317294 |
| AC107075.1 | 1.91E-05 | 1.608722325 | 7.592348145 | 0.00023633 |
| TAS2R13 | 9.45E-05 | 1.607320413 | 6.468940113 | 0.000885951 |
| RPS8P10 | 5.37E-07 | 1.604640413 | 10.06141457 | 1.18E-05 |
| REEP5 | 1.40E-08 | 1.602808936 | 12.58937927 | 5.09E-07 |
| ATG4C | 0.000243102 | 1.602075249 | 5.790238786 | 0.001926797 |
| UXT | 4.06E-05 | 1.601664175 | 7.033107401 | 0.000440408 |
| MAPRE1 | 3.21E-06 | 1.597652137 | 8.776245601 | 5.35E-05 |
| FAM166A | 7.46E-05 | 1.594058928 | 6.579341616 | 0.000732714 |
| SIRT1 | 0.000339124 | 1.59063451 | 5.518931663 | 0.002524789 |
| RN7SL737P | 3.51E-10 | 1.586860668 | 15.00305025 | 2.18E-08 |
| BRSK2 | 2.76E-06 | 1.58537728 | 8.813454474 | 4.68E-05 |
| PCDH18 | 0.00076161 | 1.584407633 | 4.940606618 | 0.004852278 |
| AK6 | 3.91E-06 | 1.583910596 | 8.56541863 | 6.32E-05 |
| HSP90B1 | 6.88E-16 | 1.583682529 | 24.01265416 | 1.77E-13 |
| RPL7AP66 | 1.23E-12 | 1.575384402 | 18.76321585 | 1.68E-10 |

| AC125807.2 | 6.15E-05 | 1.57369424 | 6.626834638 | 0.000621114 |
| --- | --- | --- | --- | --- |
| BCL2 | 4.04E-05 | 1.572931459 | 6.910803002 | 0.000438646 |
| AL031280.1 | 0.000337614 | 1.57273557 | 5.459875931 | 0.002516624 |
| ACTR1B | 8.43E-05 | 1.569362555 | 6.393894421 | 0.000807827 |
| FKBP4 | 2.65E-08 | 1.566208114 | 11.8678483 | 8.84E-07 |
| TUBA1B | 9.44E-13 | 1.565674483 | 18.82715166 | 1.34E-10 |
| FAT3 | 4.89E-05 | 1.565255389 | 6.74764165 | 0.000512754 |
| EPHX1 | 0.000395312 | 1.563051245 | 5.319156601 | 0.002868367 |
| S100A16 | 2.38E-06 | 1.561210308 | 8.779510285 | 4.16E-05 |
| AL365205.1 | 4.01E-06 | 1.561108821 | 8.425892599 | 6.45E-05 |
| AC093827.1 | 0.00048422 | 1.558891788 | 5.167659674 | 0.003366526 |
| TNFAIP3 | 7.98E-11 | 1.558333387 | 15.73632266 | 6.21E-09 |
| RTL6 | 3.09E-05 | 1.553656474 | 7.006460065 | 0.000352389 |
| GADD45GIP1 | 2.15E-11 | 1.551616778 | 16.55221912 | 2.08E-09 |
| AC027319.1 | 9.07E-10 | 1.550477878 | 14.02001846 | 4.91E-08 |
| PPP1R7 | 6.57E-06 | 1.550457697 | 8.035475071 | 9.68E-05 |
| SCYL1 | 8.47E-05 | 1.548608743 | 6.305957396 | 0.000809777 |
| EMD | 3.15E-06 | 1.547027039 | 8.512325763 | 5.25E-05 |
| C1QA | 2.76E-13 | 1.546760573 | 19.42666025 | 4.35E-11 |
| ADSS2 | 3.97E-05 | 1.541946584 | 6.7872289 | 0.000431577 |
| UTY | 8.97E-06 | 1.541339024 | 7.779709646 | 0.000127097 |
| TXNDC15 | 4.68E-05 | 1.539209056 | 6.663690081 | 0.000495375 |
| JMJD8 | 3.90E-09 | 1.535105591 | 12.90829002 | 1.70E-07 |
| CARD10 | 3.45E-08 | 1.532532868 | 11.43587337 | 1.11E-06 |
| HCFC1 | 1.61E-10 | 1.532037398 | 15.00319563 | 1.15E-08 |
| ADAMTS8 | 1.24E-06 | 1.531586326 | 9.046298277 | 2.38E-05 |
| NDUFAB1 | 2.61E-14 | 1.531460146 | 20.80279853 | 5.18E-12 |
| PRDM1 | 5.20E-12 | 1.52676644 | 17.22804539 | 5.95E-10 |
| PLIN3 | 8.67E-10 | 1.526017241 | 13.82848033 | 4.71E-08 |
| BCL6 | 3.96E-18 | 1.526007302 | 26.55584968 | 1.72E-15 |
| C2orf15 | 0.001493104 | 1.52503036 | 4.309598669 | 0.008298297 |
| SNHG20 | 0.000224811 | 1.521776725 | 5.55171955 | 0.001812463 |
| DPY30 | 5.31E-05 | 1.521616328 | 6.504692453 | 0.000548611 |
| AL451074.1 | 3.70E-07 | 1.519192983 | 9.770450906 | 8.53E-06 |
| ATAD1 | 1.92E-05 | 1.512682278 | 7.135031764 | 0.000237151 |
| RPA4 | 1.63E-07 | 1.512557522 | 10.26528348 | 4.23E-06 |
| PPP6C | 3.56E-06 | 1.512202328 | 8.23893222 | 5.84E-05 |
| MTRR | 8.74E-05 | 1.510404595 | 6.129800654 | 0.000833396 |
| BMERB1 | 1.01E-08 | 1.507506534 | 12.05253038 | 3.85E-07 |
| TESK1 | 0.001520893 | 1.506757803 | 4.245894911 | 0.008396596 |
| SPRY2 | 0.001060849 | 1.505744708 | 4.478606218 | 0.006332886 |
| PDGFC | 5.61E-10 | 1.502680592 | 13.90172525 | 3.28E-08 |
| TUSC2 | 0.000468262 | 1.501931762 | 5.000698408 | 0.003278013 |
| POLR2J | 4.24E-06 | 1.501259183 | 8.066176439 | 6.75E-05 |
| PPP1R3B | 3.09E-05 | 1.498076414 | 6.756389098 | 0.000352389 |
| AL138721.1 | 0.000671492 | 1.497995608 | 4.75307913 | 0.004391354 |
| RNF39 | 1.16E-06 | 1.494894791 | 8.871687282 | 2.26E-05 |
| FBH1 | 2.11E-07 | 1.492603885 | 9.96274835 | 5.22E-06 |
| SOX12 | 2.75E-06 | 1.491796193 | 8.29590664 | 4.66E-05 |
| DAD1 | 2.97E-05 | 1.491702771 | 6.752413315 | 0.000342019 |
| ECI1 | 0.000149016 | 1.490195632 | 5.702633733 | 0.001288907 |
| KCNJ15 | 7.32E-14 | 1.490115505 | 19.57373784 | 1.37E-11 |

| C11orf96 | 1.91E-12 | 1.486806924 | 17.42448776 | 2.46E-10 |
| --- | --- | --- | --- | --- |
| PSMF1 | 4.55E-05 | 1.483732456 | 6.442834352 | 0.000482217 |
| TOMM6 | 1.44E-05 | 1.483413537 | 7.180893178 | 0.000187638 |
| MSR1 | 4.48E-05 | 1.482681317 | 6.447286508 | 0.00047634 |
| IRAK3 | 0.000338152 | 1.481998095 | 5.143849001 | 0.002519606 |
| BACE1 | 9.68E-05 | 1.481448861 | 5.946728162 | 0.000902124 |
| MUC1 | 0.000205965 | 1.476720971 | 5.443498426 | 0.001686119 |
| AL133481.1 | 6.48E-11 | 1.474460571 | 15.02237339 | 5.24E-09 |
| AC096631.2 | 4.96E-05 | 1.474168087 | 6.345819813 | 0.000518354 |
| RTCB | 0.000915871 | 1.472036059 | 4.472289673 | 0.005621583 |
| MT2A | 4.00E-24 | 1.465326878 | 34.28631948 | 4.30E-21 |
| MIR4458HG | 1.70E-05 | 1.453530612 | 6.931831836 | 0.000214667 |
| EIF2S3 | 5.82E-09 | 1.452648831 | 11.96270946 | 2.42E-07 |
| AC026271.1 | 3.84E-09 | 1.449185111 | 12.1966727 | 1.68E-07 |
| GBP1 | 1.48E-10 | 1.447733489 | 14.23205969 | 1.06E-08 |
| AP001086.1 | 2.71E-05 | 1.444912319 | 6.598144188 | 0.000316755 |
| MRPL15 | 0.000229155 | 1.443470284 | 5.254045186 | 0.001836152 |
| AC087521.1 | 2.07E-09 | 1.440937463 | 12.51220843 | 1.00E-07 |
| RHOA-IT1 | 0.001736744 | 1.439516833 | 3.973446856 | 0.009324692 |
| MAP2K7 | 1.41E-08 | 1.438304672 | 11.29037318 | 5.14E-07 |
| TSTA3 | 0.000593592 | 1.434425275 | 4.62819005 | 0.003967045 |
| VAMP2 | 1.62E-06 | 1.432382535 | 8.293665634 | 3.01E-05 |
| RRAGA | 0.000180373 | 1.432064585 | 5.361405339 | 0.001514822 |
| PDP1 | 0.000253232 | 1.432064585 | 5.150392678 | 0.001988984 |
| STUB1 | 1.08E-06 | 1.430909284 | 8.538586365 | 2.15E-05 |
| ACLY | 0.001458385 | 1.430897899 | 4.058209137 | 0.008142466 |
| MPP1 | 4.45E-05 | 1.43054761 | 6.225081855 | 0.000474562 |
| BTG3 | 2.17E-06 | 1.430296867 | 8.099703303 | 3.85E-05 |
| CHID1 | 0.000958896 | 1.429228832 | 4.31373939 | 0.005836687 |
| FAM168B | 4.60E-07 | 1.426711197 | 9.04190192 | 1.03E-05 |
| RIC8A | 3.38E-10 | 1.425783067 | 13.50288791 | 2.13E-08 |
| ABI1 | 0.000116572 | 1.422154101 | 5.59390707 | 0.001058355 |
| DNPEP | 1.52E-11 | 1.418534086 | 15.3461858 | 1.51E-09 |
| ATP9A | 2.97E-07 | 1.41716859 | 9.250238601 | 7.10E-06 |
| MRPL51 | 3.01E-07 | 1.416914176 | 9.239796558 | 7.18E-06 |
| VKORC1 | 6.78E-08 | 1.415217713 | 10.14546491 | 1.98E-06 |
| SMCR8 | 0.000242845 | 1.415035355 | 5.114886247 | 0.001925597 |
| HSDL1 | 0.001156797 | 1.414779148 | 4.154842332 | 0.006764324 |
| GYPC | 1.75E-13 | 1.412971861 | 18.02659471 | 2.98E-11 |
| ATP5MF | 1.10E-07 | 1.411685443 | 9.821302428 | 3.00E-06 |
| P4HA1 | 6.39E-07 | 1.411493023 | 8.743585718 | 1.38E-05 |
| EPB41L4A-AS1 | 5.19E-05 | 1.408547152 | 6.035239171 | 0.000539025 |
| BTF3L4 | 2.00E-05 | 1.405952599 | 6.606445643 | 0.000244977 |
| POLE3 | 1.15E-06 | 1.405651014 | 8.349808799 | 2.25E-05 |
| USP27X | 2.09E-05 | 1.404713225 | 6.57371728 | 0.000254827 |
| WDR48 | 5.52E-05 | 1.404098681 | 5.978542878 | 0.000566421 |
| NDUFB10 | 3.20E-09 | 1.404066406 | 11.92743512 | 1.45E-07 |
| PSMB4 | 8.75E-05 | 1.3987236 | 5.675864542 | 0.000833499 |
| UBLCP1 | 5.24E-05 | 1.397363335 | 5.982022438 | 0.000542519 |
| CTNNA3 | 1.17E-05 | 1.394757187 | 6.879874045 | 0.000159102 |
| AC009220.2 | 3.95E-05 | 1.393764948 | 6.137702978 | 0.000429891 |
| AP001527.2 | 3.34E-05 | 1.390767843 | 6.22469778 | 0.000375675 |

| FTL | 1.52E-09 | 1.389471085 | 12.25277872 | 7.70E-08 |
| --- | --- | --- | --- | --- |
| AC027279.4 | 2.91E-05 | 1.388633763 | 6.299593172 | 0.000335371 |
| CSF1R | 0.00128174 | 1.388127635 | 4.014743068 | 0.007343197 |
| VHL | 5.08E-12 | 1.387147876 | 15.66662697 | 5.88E-10 |
| NOP53-AS1 | 4.16E-10 | 1.386956153 | 13.01025045 | 2.50E-08 |
| UBE2E1 | 2.02E-09 | 1.386740676 | 12.05656253 | 9.81E-08 |
| ARMCX1 | 1.67E-05 | 1.386212336 | 6.623356312 | 0.000211254 |
| PACRG | 0.000312144 | 1.383085362 | 4.848606659 | 0.002362396 |
| PSMG2 | 0.000700841 | 1.382183469 | 4.359932828 | 0.004549216 |
| NDN | 5.97E-15 | 1.381418006 | 19.64963861 | 1.31E-12 |
| CLIC1 | 0.000850081 | 1.372257281 | 4.213570566 | 0.005292383 |
| GALNT1 | 3.90E-09 | 1.372114257 | 11.53830703 | 1.70E-07 |
| RPS19BP1 | 0.001000918 | 1.371255061 | 4.113218474 | 0.006060197 |
| CCR1 | 0.001733147 | 1.36914481 | 3.780434264 | 0.009312544 |
| AP4M1 | 0.001181706 | 1.367341948 | 4.002880535 | 0.006881369 |
| CIB1 | 7.77E-05 | 1.365797593 | 5.612749144 | 0.000755446 |
| RNMT | 1.06E-11 | 1.365296764 | 14.98475415 | 1.10E-09 |
| AC026765.3 | 0.00103944 | 1.364816386 | 4.071520836 | 0.006241762 |
| TIMM17B | 1.25E-05 | 1.362884134 | 6.682246321 | 0.000167228 |
| AL355073.1 | 0.001121004 | 1.35977208 | 4.011861613 | 0.00660147 |
| JTB | 3.57E-07 | 1.359442719 | 8.764853956 | 8.31E-06 |
| HNRNPA3P4 | 1.93E-05 | 1.351062008 | 6.370732425 | 0.000237466 |
| AC021723.1 | 2.28E-08 | 1.348219372 | 10.30244429 | 7.73E-07 |
| ARHGEF25 | 1.18E-05 | 1.346933232 | 6.638787426 | 0.000159944 |
| LMLN | 6.79E-06 | 1.337436422 | 6.912201126 | 9.95E-05 |
| NEPRO | 0.00029151 | 1.334976511 | 4.719605153 | 0.002235845 |
| PACSIN2 | 0.000714824 | 1.334827501 | 4.199101265 | 0.004620206 |
| AZIN1 | 0.001596422 | 1.333214426 | 3.728803904 | 0.008718809 |
| ERP29 | 5.16E-09 | 1.330233941 | 11.02369446 | 2.18E-07 |
| BAG4 | 8.36E-05 | 1.328135036 | 5.415623351 | 0.000803602 |
| RPL10P16 | 2.06E-08 | 1.326897062 | 10.19971795 | 7.11E-07 |
| SBDSP1 | 0.000106621 | 1.321872831 | 5.250688417 | 0.000976733 |
| PDLIM7 | 2.70E-06 | 1.319487883 | 7.348807808 | 4.59E-05 |
| APBB1 | 0.000239829 | 1.318819579 | 4.774256008 | 0.001906642 |
| FP236241.2 | 0.000125133 | 1.31607067 | 5.136136351 | 0.001122143 |
| PRKAR2B | 5.47E-07 | 1.314920626 | 8.234417948 | 1.20E-05 |
| SPAG7 | 0.001427388 | 1.313427699 | 3.737303474 | 0.008008525 |
| CHCHD1 | 0.000382518 | 1.311404493 | 4.48152575 | 0.002796618 |
| GTF3C6 | 2.52E-06 | 1.309469764 | 7.330487959 | 4.35E-05 |
| HIPK1 | 1.27E-09 | 1.308565281 | 11.64097655 | 6.60E-08 |
| CTSB | 1.82E-10 | 1.30282425 | 12.68836127 | 1.27E-08 |
| RPL12P47 | 0.001745331 | 1.302452607 | 3.592323599 | 0.009358759 |
| POMZP3 | 6.98E-07 | 1.302085204 | 8.015483328 | 1.48E-05 |
| GDE1 | 1.10E-11 | 1.299363271 | 14.23843277 | 1.13E-09 |
| MED20 | 8.84E-08 | 1.296158389 | 9.142541962 | 2.50E-06 |
| DCTN4 | 2.76E-05 | 1.294187426 | 5.900885509 | 0.00032071 |
| PSMD11 | 2.03E-06 | 1.292531955 | 7.35788157 | 3.64E-05 |
| PBDC1 | 4.83E-05 | 1.292313179 | 5.577302682 | 0.000508503 |
| IKBIP | 0.000868794 | 1.289899343 | 3.948489133 | 0.005385051 |
| PDLIM1 | 0.000254401 | 1.288120627 | 4.630125984 | 0.001995589 |
| RPS13P2 | 1.25E-11 | 1.287211356 | 14.0353912 | 1.26E-09 |
| LCP2 | 9.70E-05 | 1.283008391 | 5.149226515 | 0.00090318 |

| HSP90AB2P | 2.43E-06 | 1.282705419 | 7.200901287 | 4.23E-05 |
| --- | --- | --- | --- | --- |
| GPD1L | 0.000762151 | 1.282045078 | 3.997363736 | 0.004854036 |
| ALYREF | 0.000704949 | 1.281026641 | 4.037593612 | 0.004562142 |
| DDIT3 | 2.23E-06 | 1.280856201 | 7.239146529 | 3.93E-05 |
| ZNF135 | 0.000560152 | 1.280091572 | 4.162466697 | 0.00378926 |
| MAPKAPK2 | 1.64E-10 | 1.279282198 | 12.51642431 | 1.16E-08 |
| VEGFB | 0.000236449 | 1.278648449 | 4.636715487 | 0.001883866 |
| MDH2 | 3.54E-06 | 1.278615725 | 6.969728951 | 5.81E-05 |
| NIPSNAP2 | 6.67E-06 | 1.274543811 | 6.597020913 | 9.81E-05 |
| MIER1 | 8.60E-09 | 1.274115617 | 10.27620338 | 3.38E-07 |
| KLHL23 | 5.43E-07 | 1.273236711 | 7.977353186 | 1.19E-05 |
| FKBP5 | 5.36E-11 | 1.270802063 | 13.05197492 | 4.54E-09 |
| CMPK1 | 4.14E-05 | 1.269278583 | 5.563378193 | 0.000446255 |
| WDR55 | 0.000523827 | 1.268645766 | 4.162188129 | 0.003588678 |
| PALM | 7.51E-05 | 1.26830494 | 5.230725319 | 0.000737388 |
| HLA-C | 3.97E-05 | 1.268182626 | 5.58125213 | 0.000432059 |
| RPL9P32 | 1.45E-05 | 1.266605613 | 6.128649776 | 0.000188167 |
| MRPL43 | 0.000214077 | 1.265734898 | 4.644526519 | 0.001742243 |
| AC130456.5 | 1.12E-12 | 1.265025697 | 15.11584912 | 1.55E-10 |
| SF3B5 | 1.20E-11 | 1.263851722 | 13.80032972 | 1.22E-09 |
| AP003499.4 | 1.29E-05 | 1.262427165 | 6.171928332 | 0.000171733 |
| MAT2A | 7.60E-06 | 1.262363779 | 6.462449712 | 0.000110168 |
| UBE2L6 | 0.00018748 | 1.260871733 | 4.699324857 | 0.001562478 |
| APOL2 | 0.000833187 | 1.259284669 | 3.877661734 | 0.005204202 |
| AL137186.2 | 8.06E-08 | 1.257822101 | 8.922626147 | 2.31E-06 |
| C22orf15 | 3.78E-08 | 1.256842558 | 9.328852658 | 1.20E-06 |
| CANX | 3.24E-07 | 1.255714093 | 8.148390871 | 7.67E-06 |
| TRMT10A | 0.000282382 | 1.254039918 | 4.450792752 | 0.002175874 |
| GFPT1 | 3.69E-06 | 1.253164356 | 6.809042798 | 6.00E-05 |
| RPS2P48 | 0.000325802 | 1.252960985 | 4.369133704 | 0.002446999 |
| REEP1 | 2.18E-09 | 1.24991506 | 10.82514648 | 1.05E-07 |
| AL021707.3 | 1.00E-04 | 1.249702814 | 4.998892573 | 0.000926153 |
| H2AC19 | 5.18E-06 | 1.248875224 | 6.601233241 | 7.94E-05 |
| EME1 | 0.000851642 | 1.248777562 | 3.833426 | 0.005300298 |
| GAN | 0.000132048 | 1.247810466 | 4.840592995 | 0.001170479 |
| EIF6 | 4.38E-06 | 1.246804375 | 6.68144024 | 6.95E-05 |
| IL1RL1 | 1.26E-06 | 1.242198105 | 7.329862328 | 2.41E-05 |
| GPX3 | 1.01E-06 | 1.241082819 | 7.438988782 | 2.05E-05 |
| PSMD1 | 1.10E-06 | 1.23866874 | 7.382616534 | 2.17E-05 |
| PLEKHJ1 | 2.91E-05 | 1.2359803 | 5.606479742 | 0.000335531 |
| CFLAR-AS1 | 3.38E-08 | 1.23520558 | 9.228788794 | 1.09E-06 |
| RPL31P12 | 3.50E-08 | 1.234521616 | 9.204793123 | 1.11E-06 |
| ATG9A | 0.000264016 | 1.233298341 | 4.41319702 | 0.00205374 |
| TMEM230 | 7.66E-06 | 1.232142598 | 6.303374604 | 0.000110982 |
| DYRK2 | 1.27E-05 | 1.230661159 | 6.025084494 | 0.000169728 |
| CHCHD2 | 5.70E-13 | 1.228409063 | 15.04124774 | 8.33E-11 |
| LAMTOR5 | 1.68E-06 | 1.226780868 | 7.084110573 | 3.10E-05 |
| MTF1 | 0.000128093 | 1.226108746 | 4.772598049 | 0.001143083 |
| AL138478.1 | 2.15E-11 | 1.22608357 | 13.08052684 | 2.08E-09 |
| RPS2P5 | 4.67E-06 | 1.223377676 | 6.521273286 | 7.29E-05 |
| AC020909.3 | 0.000345089 | 1.221796072 | 4.229942196 | 0.002563979 |
| TMEM176A | 5.81E-05 | 1.220259052 | 5.169093338 | 0.000591848 |

| EMP3 | 0.000215823 | 1.21998159 | 4.472333861 | 0.001752362 |
| --- | --- | --- | --- | --- |
| EGLN3 | 1.86E-05 | 1.218597141 | 5.764847583 | 0.00023155 |
| CENPX | 0.00032345 | 1.217511516 | 4.249349858 | 0.002433862 |
| RIPOR3 | 8.29E-11 | 1.216794901 | 12.26698405 | 6.42E-09 |
| RAB4A | 0.000105966 | 1.215955865 | 4.833220563 | 0.000971225 |
| DUSP22 | 7.77E-11 | 1.213979978 | 12.27272488 | 6.15E-09 |
| GSTP1 | 7.98E-11 | 1.210493504 | 12.22364736 | 6.21E-09 |
| SAP30L | 5.01E-08 | 1.210478863 | 8.836169003 | 1.54E-06 |
| AC078820.3 | 4.03E-09 | 1.209391415 | 10.15309521 | 1.75E-07 |
| ATP5MC1 | 3.21E-10 | 1.207711232 | 11.46535039 | 2.05E-08 |
| KCND3 | 0.001528285 | 1.205412384 | 3.394195057 | 0.008422147 |
| HIVEP2 | 1.30E-05 | 1.204878965 | 5.887001885 | 0.000172409 |
| CTSL | 1.66E-06 | 1.204246159 | 6.960357833 | 3.07E-05 |
| BNIP3P1 | 4.53E-06 | 1.203148385 | 6.429471015 | 7.13E-05 |
| AC010655.2 | 3.78E-06 | 1.202593 | 6.521057265 | 6.13E-05 |
| AC010655.3 | 3.78E-06 | 1.202593 | 6.521057265 | 6.13E-05 |
| AC135068.6 | 0.000227268 | 1.202188686 | 4.38012785 | 0.001824802 |
| NDUFA13 | 1.61E-12 | 1.202168364 | 14.17875361 | 2.10E-10 |
| SIRT3 | 3.64E-09 | 1.202041328 | 10.14357094 | 1.62E-07 |
| TIMM13 | 3.31E-05 | 1.200993162 | 5.380383234 | 0.000373335 |
| RRBP1 | 2.88E-14 | 1.200774385 | 16.25872038 | 5.64E-12 |
| AL157871.4 | 0.000130225 | 1.199829863 | 4.661705239 | 0.001158155 |
| RPL10P3 | 7.20E-09 | 1.198160039 | 9.756129013 | 2.91E-07 |
| GOLPH3 | 1.98E-07 | 1.197038444 | 8.023503836 | 4.96E-06 |
| FAM107A | 1.06E-05 | 1.195963901 | 5.948583295 | 0.000146853 |
| NOS1AP | 0.000295098 | 1.192526384 | 4.209657573 | 0.002258633 |
| ETS2 | 5.42E-09 | 1.191425684 | 9.848679319 | 2.28E-07 |
| FGD5-AS1 | 0.000169293 | 1.190470703 | 4.489693709 | 0.001437107 |
| AC103810.5 | 1.11E-10 | 1.190438655 | 11.8506686 | 8.38E-09 |
| GBP2 | 3.50E-10 | 1.189526745 | 11.24818287 | 2.18E-08 |
| GARS1 | 7.75E-05 | 1.187472943 | 4.881220944 | 0.00075435 |
| LINC00641 | 0.00141988 | 1.185970355 | 3.377345104 | 0.007981097 |
| STARD7 | 3.38E-05 | 1.185580528 | 5.300422375 | 0.000379228 |
| ELOVL5 | 2.53E-05 | 1.185101503 | 5.447997633 | 0.000298654 |
| CZIB | 0.000743708 | 1.181620395 | 3.696814511 | 0.004769396 |
| YIF1A | 3.53E-05 | 1.180687972 | 5.256136807 | 0.000392323 |
| TRAP1 | 0.000855478 | 1.18025282 | 3.620769149 | 0.00532055 |
| AC034102.4 | 1.94E-06 | 1.180194598 | 6.741818215 | 3.51E-05 |
| LITAF | 0.000258729 | 1.177369718 | 4.223406742 | 0.002024333 |
| RAMP1 | 3.23E-12 | 1.177290175 | 13.52726387 | 3.94E-10 |
| STAT1 | 0.000519657 | 1.177231029 | 3.866360523 | 0.003568126 |
| PEG10 | 5.42E-05 | 1.177048389 | 5.021428672 | 0.000557556 |
| AP000350.4 | 3.50E-06 | 1.17628591 | 6.417556967 | 5.76E-05 |
| MIF | 4.75E-06 | 1.17628591 | 6.261374781 | 7.38E-05 |
| MIF-AS1 | 4.75E-06 | 1.17628591 | 6.261374781 | 7.38E-05 |
| Z83840.2 | 0.000178724 | 1.17594963 | 4.407243734 | 0.001503898 |
| AL022342.1 | 6.52E-07 | 1.173651549 | 7.259925307 | 1.40E-05 |
| CAP2 | 2.28E-15 | 1.173479071 | 17.18201331 | 5.49E-13 |
| ARL2BP | 4.65E-07 | 1.17153764 | 7.418321501 | 1.04E-05 |
| HSP90B3P | 0.000451591 | 1.171107131 | 3.917651612 | 0.003180794 |
| ZBTB18 | 0.000884591 | 1.170197773 | 3.572915198 | 0.005458908 |
| TEX9 | 0.001511336 | 1.169222368 | 3.297954166 | 0.008364037 |

| RILPL1 | 2.46E-09 | 1.167708647 | 10.05230842 | 1.17E-07 |
| --- | --- | --- | --- | --- |
| HNRNPCP2 | 0.000213222 | 1.16752684 | 4.286187485 | 0.001737416 |
| AL713999.1 | 1.90E-09 | 1.166526423 | 10.17419748 | 9.30E-08 |
| AURKAIP1 | 1.94E-06 | 1.163713128 | 6.646188479 | 3.52E-05 |
| ZYX | 3.28E-11 | 1.163075249 | 12.19307909 | 3.02E-09 |
| MCTP2 | 6.46E-07 | 1.162721676 | 7.19693658 | 1.39E-05 |
| MEX3D | 6.31E-05 | 1.162325931 | 4.881536397 | 0.000634545 |
| CCDC47 | 7.20E-05 | 1.160643113 | 4.808257203 | 0.000711878 |
| AC141586.4 | 1.36E-07 | 1.159694605 | 7.963306583 | 3.59E-06 |
| SEL1L | 1.06E-12 | 1.158045025 | 13.86708111 | 1.49E-10 |
| SLC39A1 | 0.00028263 | 1.157859939 | 4.10899286 | 0.002176867 |
| ACVR1 | 6.86E-07 | 1.15784349 | 7.136428747 | 1.46E-05 |
| SF3B6 | 0.001041606 | 1.156360745 | 3.448610732 | 0.00625066 |
| BNIP3L | 2.08E-09 | 1.155975056 | 10.03566307 | 1.00E-07 |
| CA8 | 0.001682237 | 1.155337484 | 3.205036452 | 0.009103199 |
| USP31 | 0.001002973 | 1.154458244 | 3.461886377 | 0.006070626 |
| FAM104B | 0.000610698 | 1.153775316 | 3.708434086 | 0.004070949 |
| POMP | 3.10E-07 | 1.15363333 | 7.508052848 | 7.36E-06 |
| RNH1 | 0.00080433 | 1.153163796 | 3.568541428 | 0.005059225 |
| RPL37P6 | 0.001265514 | 1.152466982 | 3.339541571 | 0.007269848 |
| PKD1 | 1.94E-07 | 1.151194487 | 7.727759321 | 4.89E-06 |
| AP002981.1 | 3.44E-07 | 1.149699839 | 7.431669325 | 8.03E-06 |
| ATAD2 | 1.16E-06 | 1.147311393 | 6.811803427 | 2.26E-05 |
| TGFB1I1 | 8.58E-08 | 1.146693119 | 8.102946138 | 2.44E-06 |
| TMEM254 | 0.000175237 | 1.146455653 | 4.306515307 | 0.001480007 |
| PPP1R11 | 1.82E-05 | 1.145603304 | 5.430471115 | 0.000227289 |
| SMAD1-AS1 | 9.37E-06 | 1.145347676 | 5.759166454 | 0.000131777 |
| COX8A | 3.48E-07 | 1.14417081 | 7.388990443 | 8.14E-06 |
| PABPC4 | 1.40E-05 | 1.143123515 | 5.548144314 | 0.000183271 |
| VPS28 | 0.000723688 | 1.142269543 | 3.587238986 | 0.004666924 |
| AL033529.1 | 0.000330239 | 1.141677732 | 3.974376789 | 0.002473745 |
| CASP4 | 9.70E-08 | 1.14145578 | 8.005116972 | 2.70E-06 |
| SEM1 | 9.32E-06 | 1.141025492 | 5.739866096 | 0.000131232 |
| GLUL | 8.57E-10 | 1.140332116 | 10.33967096 | 4.66E-08 |
| EIF1AY | 0.000672195 | 1.139779862 | 3.615956779 | 0.004394384 |
| ZDHHC20 | 1.35E-05 | 1.139272232 | 5.547040314 | 0.000178016 |
| C1D | 3.00E-05 | 1.138304623 | 5.14873694 | 0.000344125 |
| PNRC1 | 5.12E-07 | 1.137334559 | 7.154895418 | 1.13E-05 |
| TMEM18 | 5.45E-05 | 1.133558908 | 4.832646569 | 0.000560304 |
| LIMS2 | 1.21E-05 | 1.1314152 | 5.565294939 | 0.000162538 |
| FKBP14 | 2.27E-07 | 1.13132382 | 7.5172977 | 5.54E-06 |
| LINC01252 | 3.05E-05 | 1.131100476 | 5.107947834 | 0.000349044 |
| DHRS4 | 0.000225403 | 1.130230256 | 4.121995357 | 0.001816376 |
| COPS2 | 1.03E-09 | 1.128605717 | 10.1442684 | 5.44E-08 |
| POLR3E | 8.00E-06 | 1.128601245 | 5.752617196 | 0.000114944 |
| UQCRBP1 | 5.51E-08 | 1.128310995 | 8.190071256 | 1.66E-06 |
| GCC1 | 0.000169751 | 1.128047897 | 4.25295346 | 0.001439652 |
| AKTIP | 0.000733438 | 1.125771135 | 3.528883454 | 0.004722152 |
| AC006042.2 | 4.84E-09 | 1.125287225 | 9.357182212 | 2.06E-07 |
| YTHDF2 | 8.87E-05 | 1.123509095 | 4.55262622 | 0.000842058 |
| SOAT1 | 3.22E-05 | 1.122318251 | 5.041260559 | 0.000364157 |
| CD163 | 7.72E-11 | 1.121111607 | 11.33716859 | 6.14E-09 |

| RPL35P5 | 2.14E-06 | 1.116543307 | 6.329243339 | 3.82E-05 |
| --- | --- | --- | --- | --- |
| TGFBI | 1.67E-07 | 1.114161805 | 7.551930218 | 4.30E-06 |
| GALE | 1.41E-09 | 1.113883873 | 9.860154784 | 7.18E-08 |
| AL080276.2 | 0.001683826 | 1.112663078 | 3.086196651 | 0.009107382 |
| DDX3Y | 0.0002141 | 1.1087783 | 4.068532393 | 0.001742243 |
| MCFD2 | 3.62E-07 | 1.108724219 | 7.142242851 | 8.38E-06 |
| P2RY14 | 0.000465693 | 1.107727455 | 3.690837096 | 0.003261664 |
| LMAN1 | 2.82E-06 | 1.107489953 | 6.146822895 | 4.76E-05 |
| EIF1AX | 7.23E-09 | 1.107048222 | 9.012387298 | 2.91E-07 |
| BATF3 | 0.001004607 | 1.105666342 | 3.314792063 | 0.006076491 |
| TIMM9 | 3.43E-05 | 1.10535112 | 4.935433537 | 0.000383301 |
| PITHD1 | 0.000733284 | 1.104265782 | 3.461572425 | 0.004722152 |
| RPS13 | 1.83E-09 | 1.104052276 | 9.646483411 | 9.05E-08 |
| AC018809.3 | 0.001502505 | 1.103183401 | 3.114489767 | 0.008330293 |
| CRELD1 | 0.001502505 | 1.103183401 | 3.114489767 | 0.008330293 |
| INTS10 | 0.000814059 | 1.101296597 | 3.402284349 | 0.005109966 |
| PLEKHM2 | 5.02E-06 | 1.099056842 | 5.82395433 | 7.72E-05 |
| AL049779.1 | 0.001773282 | 1.098849518 | 3.023179175 | 0.009491939 |
| AC133134.1 | 2.12E-10 | 1.098778082 | 10.62946584 | 1.43E-08 |
| COL4A5 | 4.23E-05 | 1.097516511 | 4.800324352 | 0.00045482 |
| RNF167 | 2.89E-06 | 1.095734284 | 6.069411997 | 4.87E-05 |
| PLTP | 4.41E-06 | 1.093514253 | 5.8568344 | 6.98E-05 |
| SAE1 | 1.04E-06 | 1.088937713 | 6.513414079 | 2.09E-05 |
| TMEM147 | 3.66E-07 | 1.087375877 | 6.999073444 | 8.45E-06 |
| RAN | 3.37E-06 | 1.084969945 | 5.937176754 | 5.57E-05 |
| MBTPS1 | 1.66E-05 | 1.084127712 | 5.182164853 | 0.000210571 |
| SLC25A5 | 0.00060586 | 1.083451771 | 3.486144531 | 0.00404312 |
| BASP1 | 2.63E-05 | 1.08262961 | 4.959203233 | 0.000307603 |
| AKR7A2 | 0.000162408 | 1.082163356 | 4.100743003 | 0.001387026 |
| YKT6 | 3.56E-05 | 1.080746398 | 4.808341029 | 0.000393781 |
| NRBP1 | 0.000938865 | 1.0803606 | 3.270680509 | 0.005737198 |
| AC034236.1 | 4.32E-08 | 1.080022423 | 7.954303043 | 1.35E-06 |
| PSMD12 | 2.64E-05 | 1.077014505 | 4.930231369 | 0.00030955 |
| AC090559.2 | 4.92E-06 | 1.076795834 | 5.715905656 | 7.59E-05 |
| ARRDC3 | 0.000215628 | 1.076274421 | 3.945939887 | 0.001751556 |
| TSG101 | 0.000515319 | 1.075267904 | 3.535398528 | 0.003545003 |
| NAA50 | 0.000774273 | 1.074563669 | 3.343081291 | 0.004915828 |
| RNF145 | 2.06E-06 | 1.073893249 | 6.105813026 | 3.69E-05 |
| VPS51 | 0.000140227 | 1.072603519 | 4.132922827 | 0.001228915 |
| GBP1P1 | 8.68E-06 | 1.072160847 | 5.426542217 | 0.00012356 |
| AP001324.1 | 2.68E-08 | 1.071344028 | 8.112707304 | 8.93E-07 |
| PDLIM4 | 4.43E-05 | 1.071079104 | 4.662581876 | 0.000473065 |
| BRD3OS | 7.01E-05 | 1.070884452 | 4.448613356 | 0.000695321 |
| AC099789.1 | 1.75E-06 | 1.070080147 | 6.161117862 | 3.21E-05 |
| SRP14 | 9.76E-12 | 1.066336661 | 11.74100192 | 1.04E-09 |
| HNRNPCP3 | 0.000356058 | 1.065242977 | 3.673467886 | 0.002632997 |
| CKAP4 | 1.36E-08 | 1.062138372 | 8.35532503 | 5.00E-07 |
| AL139287.1 | 8.90E-06 | 1.059372276 | 5.350631912 | 0.000126207 |
| AC138811.2 | 0.001187221 | 1.057733402 | 3.094365638 | 0.006906884 |
| SOD3 | 4.81E-12 | 1.057291381 | 11.96589008 | 5.72E-10 |
| RPS19P3 | 0.000164296 | 1.056489595 | 3.998151185 | 0.001401126 |
| DDX3X | 0.000145139 | 1.056169807 | 4.053807323 | 0.001260745 |

| DDAH2 | 1.07E-05 | 1.055567235 | 5.247271912 | 0.000147433 |
| --- | --- | --- | --- | --- |
| FOSL2 | 1.31E-06 | 1.055481963 | 6.208685457 | 2.49E-05 |
| CD302 | 4.48E-05 | 1.055326936 | 4.589193705 | 0.00047634 |
| AC011511.3 | 0.00013213 | 1.055031935 | 4.092466342 | 0.001170544 |
| ATP8B5P | 0.000249125 | 1.05500589 | 3.801801408 | 0.001963468 |
| AC011603.3 | 1.02E-06 | 1.054431237 | 6.319168015 | 2.05E-05 |
| RPL29 | 3.73E-10 | 1.053808832 | 9.935049133 | 2.31E-08 |
| AC093591.2 | 0.00066128 | 1.053632378 | 3.350144884 | 0.004340095 |
| COG5 | 4.68E-06 | 1.053373005 | 5.614231862 | 7.30E-05 |
| AL954705.1 | 4.95E-05 | 1.05320718 | 4.534443168 | 0.00051783 |
| JOSD1 | 0.000684051 | 1.051756597 | 3.328716444 | 0.00446391 |
| AC015813.2 | 0.000777079 | 1.051592201 | 3.269962496 | 0.0049268 |
| TWF1P1 | 0.001459857 | 1.050408458 | 2.978632551 | 0.008143221 |
| SLA2 | 0.000334141 | 1.047770501 | 3.642124145 | 0.002497859 |
| SNRPF | 0.000535183 | 1.047380172 | 3.426501741 | 0.003655518 |
| ZACN | 0.000227602 | 1.046917189 | 3.813735367 | 0.001825307 |
| EMC3 | 6.42E-06 | 1.046068086 | 5.431454813 | 9.51E-05 |
| PSMB5 | 3.04E-07 | 1.045432909 | 6.812885658 | 7.24E-06 |
| FBXO7 | 0.000420514 | 1.045209592 | 3.528856946 | 0.003014172 |
| KMT2B | 1.36E-08 | 1.044253254 | 8.213577776 | 5.00E-07 |
| SRSF1 | 0.001884772 | 1.044039007 | 2.844736154 | 0.009977815 |
| NUCB1 | 5.51E-08 | 1.043395861 | 7.573657218 | 1.66E-06 |
| PHKA1 | 0.000131841 | 1.041489071 | 4.04092605 | 0.001170038 |
| KSR2 | 0.001442686 | 1.04146688 | 2.958628577 | 0.008074535 |
| RPS29 | 1.34E-08 | 1.041128456 | 8.195310879 | 4.98E-07 |
| TXN | 0.000296895 | 1.040718322 | 3.6710263 | 0.00226764 |
| TCEAL4 | 0.000108685 | 1.04037611 | 4.123874416 | 0.00099415 |
| FAM153B | 3.31E-06 | 1.039775823 | 5.697624826 | 5.50E-05 |
| PLP2 | 0.000274788 | 1.034026235 | 3.682170263 | 0.002122726 |
| MAP4K3 | 0.001103304 | 1.032152838 | 3.052390401 | 0.006520459 |
| RN7SL731P | 1.57E-05 | 1.031587425 | 4.956388449 | 0.000200791 |
| HEBP2 | 3.91E-10 | 1.031287623 | 9.702272735 | 2.39E-08 |
| FRG1FP | 1.75E-05 | 1.031006803 | 4.903689362 | 0.000220302 |
| SH3GLB1 | 5.02E-06 | 1.0305795 | 5.461005 | 7.72E-05 |
| ANKRD39 | 0.000511787 | 1.030341441 | 3.390761746 | 0.003525546 |
| PKM | 2.63E-11 | 1.0295998 | 10.89282868 | 2.49E-09 |
| SETP14 | 0.000755756 | 1.028557724 | 3.21076448 | 0.004825072 |
| AP2S1 | 1.87E-06 | 1.028535688 | 5.890800333 | 3.41E-05 |
| NUP62 | 4.90E-05 | 1.028252121 | 4.431635471 | 0.000513366 |
| CCT7 | 2.81E-11 | 1.026375517 | 10.83034227 | 2.63E-09 |
| AP001107.2 | 0.000119818 | 1.026301241 | 4.024617477 | 0.001082447 |
| RAB1B | 0.000157234 | 1.026301241 | 3.90348922 | 0.001345984 |
| AC073130.3 | 6.02E-10 | 1.025096157 | 9.45182534 | 3.47E-08 |
| SNX18 | 0.000248758 | 1.023584474 | 3.689226962 | 0.001961422 |
| ATIC | 0.000703532 | 1.02353023 | 3.226900316 | 0.004560114 |
| UGP2 | 3.39E-08 | 1.023495765 | 7.645568825 | 1.09E-06 |
| AC138305.3 | 0.000737274 | 1.022396062 | 3.202523691 | 0.004741843 |
| RPL7L1 | 3.61E-05 | 1.020933687 | 4.535420591 | 0.000398693 |
| RHOQP1 | 7.87E-06 | 1.020415259 | 5.208330709 | 0.000113372 |
| FAM102A | 0.000190792 | 1.0190827 | 3.790416665 | 0.001587185 |
| RPL19 | 7.91E-11 | 1.018350656 | 10.28721473 | 6.21E-09 |
| UBE2D3 | 1.46E-05 | 1.01658007 | 4.916285217 | 0.000189136 |

| TSPAN14 | 1.66E-07 | 1.01376093 | 6.872304393 | 4.30E-06 |
| --- | --- | --- | --- | --- |
| ANAPC16 | 5.64E-07 | 1.012949017 | 6.329387745 | 1.23E-05 |
| GLUD1 | 2.00E-06 | 1.010335989 | 5.7576794 | 3.59E-05 |
| KLHL24 | 1.92E-05 | 1.009497623 | 4.761042302 | 0.000237296 |
| ADAT1 | 0.000155281 | 1.009220568 | 3.844001668 | 0.001334264 |
| PDIA6 | 0.001511999 | 1.009163083 | 2.846292625 | 0.008365172 |
| NFE2L1 | 1.23E-05 | 1.00907193 | 4.953940173 | 0.000165487 |
| RASSF6 | 7.78E-09 | 1.008195629 | 8.175479953 | 3.10E-07 |
| ATP5MPL | 0.000100173 | 1.008108679 | 4.031678931 | 0.000926484 |
| ZNFX1 | 0.000566717 | 1.007982564 | 3.272549901 | 0.003828012 |
| CCDC200 | 3.84E-05 | 1.006919458 | 4.446540398 | 0.00042014 |
| DCAF1 | 8.19E-06 | 1.005146588 | 5.11313992 | 0.000117205 |
| SHFL | 0.000863103 | 1.0049094 | 3.078979228 | 0.005360682 |
| HIF1A-AS1 | 0.000575831 | 1.004841575 | 3.255390004 | 0.003876685 |
| SQSTM1 | 4.29E-08 | 1.004832839 | 7.402931938 | 1.34E-06 |
| SYF2 | 0.000684887 | 1.004801823 | 3.179575878 | 0.00446777 |
| COPA | 2.70E-06 | 1.003771344 | 5.589261078 | 4.60E-05 |
| ANAPC13 | 0.000190595 | 1.00360865 | 3.733312317 | 0.001586267 |
| ST13P10 | 0.001281897 | 1.003054128 | 2.900979907 | 0.007343197 |
| RHOQP2 | 2.17E-06 | 1.002901469 | 5.68071522 | 3.85E-05 |
| UQCRFS1 | 6.63E-06 | 1.002687775 | 5.192357585 | 9.77E-05 |
| NAPG | 0.000125273 | 1.001819988 | 3.909245149 | 0.001122849 |
| HMGN3 | 0.001042581 | 1.001722417 | 2.987026168 | 0.006254461 |
| NBN | 1.06E-05 | 1.001713502 | 4.98526911 | 0.000146144 |
| RAB1A | 9.26E-06 | 1.000817135 | 5.037304078 | 0.000130606 |
| COL1A1 | 8.24E-09 | -1.001579668 | -8.096741445 | 3.26E-07 |
| POLR1C | 9.94E-05 | -1.003798344 | -4.017982245 | 0.000922722 |
| AC211486.2 | 0.000489255 | -1.006280806 | -3.331257453 | 0.003395074 |
| TNC | 0.000766748 | -1.006743681 | -3.136356489 | 0.004876515 |
| KCNH6 | 4.02E-05 | -1.007689891 | -4.429938209 | 0.000436653 |
| MTATP6P1 | 6.14E-06 | -1.008921622 | -5.258297685 | 9.16E-05 |
| ENAH | 0.001018265 | -1.013131255 | -3.031429703 | 0.006138799 |
| MT-TC | 1.74E-09 | -1.015189995 | -8.893334304 | 8.65E-08 |
| SELENOW | 2.25E-08 | -1.017343022 | -7.780804691 | 7.63E-07 |
| NR2F1-AS1 | 0.000876959 | -1.017376585 | -3.110141147 | 0.005424628 |
| CORO1C | 1.00E-05 | -1.017448428 | -5.086878687 | 0.00013927 |
| IRF2BP2 | 8.76E-05 | -1.017821229 | -4.129628973 | 0.000834152 |
| FGL2 | 2.60E-06 | -1.018132313 | -5.6868878 | 4.46E-05 |
| LINC01102 | 1.53E-05 | -1.018832232 | -4.905075463 | 0.000196868 |
| TEX41 | 3.57E-06 | -1.018875128 | -5.549717403 | 5.85E-05 |
| HHIP | 2.67E-06 | -1.020459954 | -5.688136511 | 4.56E-05 |
| ZNF600 | 0.000221517 | -1.023680745 | -3.741137403 | 0.001789059 |
| AC008677.1 | 0.000126798 | -1.027806068 | -4.005244267 | 0.001134297 |
| AL158154.3 | 0.000156598 | -1.028139398 | -3.912290997 | 0.001341794 |
| EPCAM | 2.77E-07 | -1.029721376 | -6.753153384 | 6.65E-06 |
| RNU1-28P | 5.65E-06 | -1.03056836 | -5.408094819 | 8.54E-05 |
| ANTXR2 | 0.000231884 | -1.03516595 | -3.762547271 | 0.001853149 |
| FAM215B | 7.03E-12 | -1.036400922 | -11.55909424 | 7.79E-10 |
| CPS1 | 1.41E-05 | -1.037129283 | -5.031728247 | 0.000183944 |
| AHNAK2 | 9.39E-06 | -1.041602991 | -5.236643632 | 0.000131903 |
| ASNSD1 | 0.000837376 | -1.045594613 | -3.217377951 | 0.005222956 |
| SMIM12 | 0.001687488 | -1.04598544 | -2.900265976 | 0.009120813 |

| TPM2 | 9.79E-10 | -1.052978378 | -9.486560325 | 5.23E-08 |
| --- | --- | --- | --- | --- |
| PEA15 | 0.001446351 | -1.054919627 | -2.995683095 | 0.008085149 |
| HSPA1B | 4.79E-10 | -1.055889032 | -9.840583648 | 2.83E-08 |
| MT-TW | 9.23E-07 | -1.056850751 | -6.37796642 | 1.90E-05 |
| TYRO3 | 0.001726145 | -1.057079593 | -2.920629121 | 0.009288572 |
| FIRRE | 1.97E-05 | -1.057373111 | -4.976512523 | 0.000241387 |
| MTRNR2L10 | 6.93E-05 | -1.058302103 | -4.401809875 | 0.000687848 |
| ZEB2-AS1 | 0.000264016 | -1.062008789 | -3.800260781 | 0.00205374 |
| COX7A1 | 2.53E-07 | -1.06571254 | -7.030249357 | 6.15E-06 |
| AC027612.2 | 0.000233441 | -1.069605847 | -3.884618555 | 0.001863962 |
| KLF3-AS1 | 0.001593347 | -1.075020661 | -3.007574268 | 0.008709819 |
| AL356273.6 | 0.001040082 | -1.076690778 | -3.21169568 | 0.006243567 |
| AC105052.3 | 0.001445482 | -1.077653289 | -3.060521742 | 0.008085149 |
| ITPR3 | 2.76E-05 | -1.07781104 | -4.914640183 | 0.000320689 |
| TRG-AS1 | 1.71E-06 | -1.078458697 | -6.219078347 | 3.15E-05 |
| IRS1 | 5.71E-07 | -1.079613354 | -6.740476678 | 1.25E-05 |
| MT-TR | 0.000232932 | -1.079613828 | -3.921990414 | 0.001860705 |
| GJC1 | 1.14E-10 | -1.082854674 | -10.76678019 | 8.55E-09 |
| SYNM | 5.30E-13 | -1.0848353 | -13.31735651 | 7.94E-11 |
| LIMK2 | 0.000231431 | -1.086267083 | -3.949208691 | 0.00185119 |
| FBXL2 | 0.000316042 | -1.086273472 | -3.802233874 | 0.002385734 |
| MT-ND4L | 1.33E-07 | -1.086455772 | -7.470450812 | 3.54E-06 |
| CTRC | 3.71E-05 | -1.088372911 | -4.821659755 | 0.000408365 |
| CLIC4P1 | 0.000219268 | -1.090295847 | -3.989420244 | 0.001775602 |
| ACSS3 | 0.001055465 | -1.094695208 | -3.258421736 | 0.006308983 |
| LILRB1 | 1.31E-06 | -1.095089323 | -6.443081848 | 2.49E-05 |
| NTM | 0.000591925 | -1.096716377 | -3.539907949 | 0.003958796 |
| LRIG1 | 0.000479619 | -1.099562676 | -3.649562407 | 0.003348543 |
| KITLG | 0.000184917 | -1.099839267 | -4.105724536 | 0.001545345 |
| LINC01220 | 3.37E-05 | -1.101331935 | -4.925177981 | 0.000378345 |
| CSAD | 0.000344109 | -1.101981446 | -3.816496114 | 0.00255878 |
| MT-ATP6 | 2.89E-08 | -1.102288217 | -8.311054715 | 9.51E-07 |
| DZIP3 | 0.001592971 | -1.10561389 | -3.093277901 | 0.008709819 |
| ZNF350 | 0.000360218 | -1.108365581 | -3.816583816 | 0.002658028 |
| CACNA1C | 1.95E-07 | -1.115744445 | -7.486749264 | 4.90E-06 |
| FOS | 3.75E-08 | -1.115779926 | -8.285554974 | 1.19E-06 |
| NNT-AS1 | 0.00022703 | -1.117581114 | -4.072373251 | 0.00182447 |
| AC004990.1 | 0.000242078 | -1.11839933 | -4.04418213 | 0.001920345 |
| AC138776.1 | 5.87E-07 | -1.119032959 | -6.972766683 | 1.27E-05 |
| IL31RA | 0.00019861 | -1.119148275 | -4.143085109 | 0.00164091 |
| BUB1B | 4.46E-06 | -1.122015117 | -6.003063858 | 7.04E-05 |
| MKI67 | 1.61E-10 | -1.124679464 | -11.01444995 | 1.15E-08 |
| SNRPC | 0.000546373 | -1.126044848 | -3.673733174 | 0.003709779 |
| ANKRD20A8P | 5.44E-06 | -1.126105265 | -5.928484593 | 8.24E-05 |
| BX005214.2 | 0.000136226 | -1.130513372 | -4.370272277 | 0.001199848 |
| CENPJ | 0.000352637 | -1.130559537 | -3.90345167 | 0.002614746 |
| LINC00265 | 0.000196217 | -1.132218793 | -4.197433831 | 0.00162492 |
| RPS14P4 | 3.73E-09 | -1.133074445 | -9.549666517 | 1.65E-07 |
| ARHGAP15 | 0.001599058 | -1.136237671 | -3.17707494 | 0.008730597 |
| VPS33B-DT | 0.00031339 | -1.137600457 | -3.986055189 | 0.002369866 |
| NUDT6 | 0.000697743 | -1.13871404 | -3.594128592 | 0.004532228 |
| CAVIN2 | 2.35E-15 | -1.139747517 | -16.67262713 | 5.59E-13 |

| C20orf27 | 2.14E-05 | -1.143415112 | -5.339970762 | 0.000259808 |
| --- | --- | --- | --- | --- |
| LINC00299 | 0.001861584 | -1.143640656 | -3.122273244 | 0.009886454 |
| RPL6P4 | 9.64E-05 | -1.144114895 | -4.594512023 | 0.000899623 |
| KCNN3 | 0.000245118 | -1.144537232 | -4.13249404 | 0.001936063 |
| GTDC1 | 0.000284697 | -1.146013376 | -4.063324673 | 0.002190945 |
| KLRD1 | 1.87E-05 | -1.146975398 | -5.422644913 | 0.000232809 |
| AC005899.9 | 0.001747469 | -1.147019203 | -3.163009177 | 0.009367481 |
| RESF1 | 1.23E-05 | -1.147485852 | -5.632690736 | 0.00016553 |
| MTND2P28 | 0.000236686 | -1.149360511 | -4.167383443 | 0.00188493 |
| PRUNE2 | 6.43E-08 | -1.149542131 | -8.267566832 | 1.89E-06 |
| MYRFL | 0.000161009 | -1.150375577 | -4.363547045 | 0.001377655 |
| CAMK2G | 1.66E-06 | -1.153583046 | -6.668351869 | 3.07E-05 |
| CES1 | 0.000183672 | -1.153592416 | -4.309771451 | 0.00153632 |
| MTATP8P1 | 0.000199906 | -1.155561608 | -4.274623012 | 0.001650242 |
| SEC22B4P | 1.96E-06 | -1.156567822 | -6.600880099 | 3.54E-05 |
| MTND4P12 | 4.35E-09 | -1.157764681 | -9.680162458 | 1.87E-07 |
| TRIM58 | 1.84E-10 | -1.16125039 | -11.30614237 | 1.27E-08 |
| FBXL17 | 3.49E-05 | -1.16267969 | -5.182422802 | 0.000388993 |
| SULT1E1 | 0.000654613 | -1.166424531 | -3.71391375 | 0.004307159 |
| MARVELD3 | 5.36E-06 | -1.174864339 | -6.192248201 | 8.15E-05 |
| AC126603.1 | 0.000150942 | -1.175584436 | -4.492131972 | 0.001302488 |
| AC073389.2 | 0.000214001 | -1.17608232 | -4.315734006 | 0.001742243 |
| LINC00649 | 5.78E-06 | -1.176208948 | -6.161401886 | 8.66E-05 |
| PHLPP2 | 6.35E-05 | -1.177220526 | -4.941440895 | 0.000637492 |
| SERTAD1 | 0.001829351 | -1.180522836 | -3.231920727 | 0.009743575 |
| HULC | 2.19E-13 | -1.182729472 | -14.97342768 | 3.67E-11 |
| SNAP25-AS1 | 1.55E-06 | -1.183561078 | -6.875206026 | 2.90E-05 |
| FAM241A | 4.17E-06 | -1.184748621 | -6.373302117 | 6.69E-05 |
| TIGD5 | 0.000117473 | -1.187081924 | -4.665305496 | 0.001064945 |
| WTIP | 0.001385233 | -1.188071887 | -3.396076415 | 0.007822416 |
| FABP5P2 | 0.000285786 | -1.190002457 | -4.217319383 | 0.002198403 |
| UEVLD | 0.000409232 | -1.190667111 | -4.03401653 | 0.002955565 |
| AC106872.6 | 0.000425703 | -1.192472552 | -4.019697223 | 0.003042997 |
| SLC8A1 | 0.000143166 | -1.192929738 | -4.585811769 | 0.001250141 |
| ZNF23 | 0.00020419 | -1.194555931 | -4.407870011 | 0.001674267 |
| TMEM154 | 0.001607525 | -1.195883177 | -3.341108942 | 0.008763744 |
| Z95331.1 | 0.000564339 | -1.203124667 | -3.908302704 | 0.003813622 |
| ATP1A2 | 9.92E-06 | -1.205494719 | -6.031674767 | 0.000138359 |
| CIP2A | 0.000690236 | -1.206406353 | -3.813453079 | 0.004489851 |
| CYB5R3 | 3.10E-08 | -1.208735415 | -9.075163983 | 1.01E-06 |
| CEP126 | 0.000440511 | -1.213522646 | -4.072634183 | 0.003125329 |
| AC074386.1 | 1.34E-05 | -1.214200015 | -5.91517959 | 0.000177154 |
| RNU6-758P | 0.0005018 | -1.214354883 | -4.006726696 | 0.003467654 |
| AC005165.1 | 0.000146193 | -1.215084562 | -4.659936915 | 0.001268096 |
| GCNT4 | 0.001819112 | -1.215321454 | -3.330151586 | 0.009697512 |
| AC007342.1 | 0.000578303 | -1.216939592 | -3.940261014 | 0.003887601 |
| AC007744.1 | 4.35E-05 | -1.218962474 | -5.31681452 | 0.000465429 |
| SEMA4A | 0.00056815 | -1.221109132 | -3.963154813 | 0.003836272 |
| CCDC26 | 8.53E-05 | -1.222272999 | -4.973743883 | 0.000814508 |
| MT-TY | 5.06E-12 | -1.223862921 | -13.82448709 | 5.88E-10 |
| WDR66 | 0.000430905 | -1.234370158 | -4.154419332 | 0.003072969 |
| GOLGA2P2Y | 0.001176348 | -1.236159829 | -3.621286155 | 0.006858901 |

| AL136164.3 | 2.01E-07 | -1.240580211 | -8.307999429 | 5.01E-06 |
| --- | --- | --- | --- | --- |
| AL669831.3 | 1.08E-05 | -1.242342652 | -6.171500107 | 0.000148454 |
| GPATCH1 | 2.18E-05 | -1.243868481 | -5.798230756 | 0.000264214 |
| AC092279.1 | 0.000268374 | -1.244022748 | -4.442727754 | 0.002083746 |
| MTRNR2L1 | 0.000101604 | -1.245426281 | -4.973098501 | 0.000938297 |
| FABP5P7 | 0.001721251 | -1.24637096 | -3.445163447 | 0.009267691 |
| TRPM3 | 0.00041919 | -1.246545024 | -4.210316877 | 0.003007018 |
| KCNAB2 | 0.001031405 | -1.247145837 | -3.724689087 | 0.0062037 |
| XKR4 | 0.00068761 | -1.247860589 | -3.946555756 | 0.004477592 |
| IDO1 | 0.000702885 | -1.248980747 | -3.938181124 | 0.004559151 |
| AC215522.2 | 0.000914924 | -1.250067238 | -3.79847277 | 0.005617661 |
| AC004922.1 | 0.001411606 | -1.251766527 | -3.567893069 | 0.007941915 |
| PDK3 | 4.25E-05 | -1.253074966 | -5.478555328 | 0.000456086 |
| MAP3K13 | 0.000776405 | -1.25444394 | -3.901209936 | 0.004924233 |
| AC036108.2 | 1.83E-09 | -1.25518915 | -10.96824379 | 9.05E-08 |
| IRX2 | 2.73E-10 | -1.255222611 | -12.0053708 | 1.78E-08 |
| STMND1 | 6.13E-05 | -1.258392458 | -5.301186429 | 0.000620109 |
| TMEM67 | 0.000162241 | -1.259365274 | -4.772793718 | 0.001386247 |
| OR4K2 | 1.66E-06 | -1.260816603 | -7.286224303 | 3.07E-05 |
| SLX4IP | 1.03E-06 | -1.26103413 | -7.55063771 | 2.07E-05 |
| ZNF727 | 1.07E-05 | -1.261896064 | -6.273646973 | 0.000147433 |
| AL031320.2 | 4.91E-06 | -1.262275491 | -6.701012668 | 7.59E-05 |
| VDAC1P8 | 5.85E-06 | -1.262275491 | -6.605560667 | 8.74E-05 |
| AC114801.3 | 1.69E-24 | -1.262470462 | -30.01030256 | 2.06E-21 |
| SNORA81 | 0.000390372 | -1.263572771 | -4.306914332 | 0.002841675 |
| MIB1 | 0.000643187 | -1.264840027 | -4.036943143 | 0.004248796 |
| KYNU | 5.74E-06 | -1.266018252 | -6.634880565 | 8.62E-05 |
| C2orf92 | 0.001122434 | -1.266455416 | -3.735839591 | 0.006605637 |
| ZFP82 | 7.62E-05 | -1.269032775 | -5.225871325 | 0.000745967 |
| ZNF583 | 0.001475665 | -1.270143411 | -3.59579161 | 0.008216361 |
| FAM131A | 0.001593864 | -1.27078766 | -3.555090398 | 0.008710043 |
| PCDH7 | 4.23E-10 | -1.273299642 | -11.93586416 | 2.53E-08 |
| OR10H1 | 0.001459724 | -1.278199333 | -3.624627141 | 0.008143221 |
| AL591684.1 | 4.47E-05 | -1.279641222 | -5.566561656 | 0.000475586 |
| UPK3BL1 | 0.000298045 | -1.281123397 | -4.516879837 | 0.002273573 |
| GRIA4 | 0.001101354 | -1.2816436 | -3.79119518 | 0.006516741 |
| SH3BGRL2 | 2.97E-05 | -1.28191225 | -5.803148926 | 0.000341998 |
| NR2F2-AS1 | 0.001254252 | -1.283571795 | -3.724431549 | 0.007216486 |
| MT-CO1 | 1.46E-05 | -1.28527701 | -6.213242793 | 0.000189577 |
| STAR | 0.001608581 | -1.285578296 | -3.591336226 | 0.008766521 |
| MUC20 | 5.55E-05 | -1.287308066 | -5.478833669 | 0.000567772 |
| PSMD10P2 | 0.001080254 | -1.293178832 | -3.836181383 | 0.006421473 |
| PRDM6 | 0.000718728 | -1.293908797 | -4.067318739 | 0.004639087 |
| AC114498.2 | 2.18E-07 | -1.294839781 | -8.62506607 | 5.36E-06 |
| SAMD9L | 0.000520128 | -1.299171246 | -4.266335241 | 0.003568929 |
| ZNF605 | 5.58E-05 | -1.301702893 | -5.536253732 | 0.000571327 |
| LILRA2 | 0.001881783 | -1.301900065 | -3.54823815 | 0.009970559 |
| MIR99AHG | 9.05E-07 | -1.30494882 | -7.886281241 | 1.87E-05 |
| AC025884.1 | 0.000211487 | -1.305646889 | -4.797880806 | 0.00172482 |
| DLX6-AS1 | 0.001357534 | -1.306925101 | -3.747280127 | 0.007701677 |
| CRYAB | 3.08E-07 | -1.307520078 | -8.51388117 | 7.31E-06 |
| ABCC13 | 0.000315988 | -1.308147787 | -4.57894885 | 0.002385734 |

| DNAH7 | 3.26E-07 | -1.30824602 | -8.486990156 | 7.69E-06 |
| --- | --- | --- | --- | --- |
| AC008132.1 | 1.26E-08 | -1.308293765 | -10.33447709 | 4.70E-07 |
| RIMKLB | 0.000645884 | -1.308371878 | -4.173503797 | 0.00426046 |
| AL136441.1 | 2.02E-08 | -1.309780983 | -10.07949726 | 6.99E-07 |
| AL034546.1 | 0.000396786 | -1.315511605 | -4.474638087 | 0.00287678 |
| KIF20B | 0.000568648 | -1.316776797 | -4.273147252 | 0.003838213 |
| BX470102.2 | 1.51E-06 | -1.317136205 | -7.667246601 | 2.82E-05 |
| PPP1R14A | 1.97E-06 | -1.321093074 | -7.537764206 | 3.55E-05 |
| BCL2L12 | 2.41E-05 | -1.322516037 | -6.107961766 | 0.000286778 |
| DNAH5 | 2.44E-05 | -1.325237803 | -6.113042773 | 0.000289215 |
| AL031595.2 | 5.66E-06 | -1.326052012 | -6.958380378 | 8.54E-05 |
| WDR7 | 0.001017902 | -1.327079185 | -3.971011194 | 0.006138799 |
| TTN | 2.47E-06 | -1.328263287 | -7.447826947 | 4.29E-05 |
| ARHGAP11B | 0.000650154 | -1.330293775 | -4.239625034 | 0.004282442 |
| METTL7B | 8.42E-05 | -1.330538497 | -5.421516778 | 0.000807357 |
| CBX1P1 | 0.000550099 | -1.334010509 | -4.348285715 | 0.003728157 |
| PTK2B | 0.000261757 | -1.334174209 | -4.779147113 | 0.002040462 |
| NDUFAF2 | 0.001091482 | -1.339206714 | -3.966708286 | 0.006471382 |
| ALDH1A1 | 0.000427407 | -1.341683168 | -4.520343234 | 0.003051595 |
| AL136982.2 | 0.000259222 | -1.342327097 | -4.814026403 | 0.00202645 |
| AC074351.1 | 5.26E-06 | -1.347555257 | -7.114000357 | 8.03E-05 |
| LINC00882 | 1.22E-07 | -1.349242176 | -9.326258748 | 3.30E-06 |
| MT-TM | 9.55E-06 | -1.351829196 | -6.78592292 | 0.000133868 |
| SPARC | 3.31E-05 | -1.360891353 | -6.097261484 | 0.000373223 |
| MIR1248 | 0.000207705 | -1.365412448 | -5.02820477 | 0.001699275 |
| GUCY1A1 | 5.19E-06 | -1.366090034 | -7.219959007 | 7.94E-05 |
| ANAPC1 | 8.35E-06 | -1.366303087 | -6.93858343 | 0.000119454 |
| CRB1 | 7.81E-05 | -1.371232933 | -5.63223584 | 0.000757861 |
| RPS14P5 | 5.42E-07 | -1.37254808 | -8.60070525 | 1.19E-05 |
| FP700107.1 | 6.17E-05 | -1.375149505 | -5.789231567 | 0.00062202 |
| RNF149 | 7.08E-05 | -1.376181694 | -5.710775213 | 0.000701299 |
| ZBTB40 | 0.000147697 | -1.382659691 | -5.296456254 | 0.001279316 |
| GRHPR | 0.000243382 | -1.383996579 | -5.001364593 | 0.001927345 |
| GABPA | 4.35E-05 | -1.384416898 | -6.038705506 | 0.000465429 |
| AL031963.2 | 0.00014722 | -1.391431457 | -5.332009634 | 0.001275794 |
| COL1A2 | 3.60E-05 | -1.396794732 | -6.20757937 | 0.000397797 |
| JAKMIP2 | 2.42E-08 | -1.399370495 | -10.65728404 | 8.19E-07 |
| RPL4P5 | 0.000334874 | -1.406827703 | -4.888892622 | 0.002500301 |
| ULK4P2 | 0.000317161 | -1.409427495 | -4.931192653 | 0.002392445 |
| AL008725.1 | 0.001164098 | -1.412403204 | -4.144005996 | 0.006800487 |
| AASS | 0.000852121 | -1.414440152 | -4.34162246 | 0.005301472 |
| DCN | 2.31E-07 | -1.415436153 | -9.39257937 | 5.63E-06 |
| COL19A1 | 9.57E-05 | -1.415680821 | -5.689893744 | 0.000893947 |
| TENM3 | 0.000128781 | -1.418953104 | -5.519937714 | 0.001148104 |
| SULF1 | 0.000909369 | -1.428817046 | -4.345403943 | 0.00558918 |
| PCBD1 | 1.35E-08 | -1.432001768 | -11.26967223 | 4.98E-07 |
| NFS1 | 2.28E-05 | -1.433398891 | -6.654006713 | 0.000273881 |
| LINC00278 | 1.14E-08 | -1.434059733 | -11.38938508 | 4.30E-07 |
| HEMK1 | 0.00078388 | -1.43416847 | -4.45416951 | 0.004959599 |
| H19 | 4.22E-06 | -1.435044141 | -7.712365315 | 6.74E-05 |
| LINC02805 | 4.64E-06 | -1.435617145 | -7.656172336 | 7.27E-05 |
| ATAD3C | 2.44E-05 | -1.43619681 | -6.62389284 | 0.000289483 |

| LINC00907 | 3.18E-05 | -1.438341715 | -6.469511093 | 0.000360441 |
| --- | --- | --- | --- | --- |
| TCIRG1 | 0.000906058 | -1.439101206 | -4.378960377 | 0.005570704 |
| IL18R1 | 0.000323655 | -1.440427301 | -5.026973632 | 0.002434399 |
| KCNJ16 | 8.44E-06 | -1.443086431 | -7.32177354 | 0.000120464 |
| PCAT1 | 6.75E-10 | -1.445551895 | -13.25637856 | 3.81E-08 |
| DLG2 | 0.001058996 | -1.448882321 | -4.310578227 | 0.006328018 |
| AF117829.1 | 0.001388767 | -1.448901526 | -4.140048567 | 0.007837533 |
| FLVCR1-DT | 0.001567098 | -1.460877375 | -4.097620384 | 0.008599758 |
| PROSER1 | 1.28E-06 | -1.461771799 | -8.614327214 | 2.44E-05 |
| MALL | 2.41E-05 | -1.464244497 | -6.76139927 | 0.0002871 |
| ZFHX4 | 1.38E-08 | -1.469223483 | -11.54607452 | 5.06E-07 |
| AC002094.5 | 0.001111285 | -1.470453394 | -4.343975713 | 0.006554792 |
| LIX1 | 0.001655347 | -1.477289068 | -4.108504677 | 0.008984276 |
| CENPF | 1.57E-09 | -1.477892288 | -13.01081036 | 7.90E-08 |
| AC006122.1 | 0.000878132 | -1.479171227 | -4.520998526 | 0.005429191 |
| MT-ND3 | 1.34E-12 | -1.479681432 | -17.56852121 | 1.81E-10 |
| RNF141 | 1.20E-05 | -1.480436525 | -7.284873421 | 0.000162075 |
| ETFBKMT | 0.000576491 | -1.481401934 | -4.798568421 | 0.003879696 |
| FADS6 | 0.000745208 | -1.481786955 | -4.634618518 | 0.004776069 |
| MYCT1 | 9.68E-05 | -1.483327416 | -5.954416592 | 0.000902124 |
| AC007402.1 | 0.001185805 | -1.484612694 | -4.343956814 | 0.006900844 |
| CCDC80 | 1.68E-08 | -1.486528502 | -11.5579487 | 5.98E-07 |
| BCL2L11 | 3.13E-09 | -1.486970503 | -12.64566569 | 1.43E-07 |
| SNHG1 | 9.08E-08 | -1.487048898 | -10.47189109 | 2.55E-06 |
| AC099792.1 | 5.33E-05 | -1.489085335 | -6.363706574 | 0.000549932 |
| CR381670.1 | 1.38E-05 | -1.489803363 | -7.242954408 | 0.000180363 |
| AP003465.1 | 0.001426767 | -1.494958515 | -4.254124353 | 0.008007497 |
| AL133353.1 | 0.000326927 | -1.49623535 | -5.21520202 | 0.002452958 |
| DCDC1 | 1.16E-05 | -1.497236879 | -7.388411058 | 0.000158478 |
| AC005480.1 | 3.94E-05 | -1.498847204 | -6.600880784 | 0.000429864 |
| LINC01579 | 5.17E-09 | -1.502062836 | -12.44692475 | 2.18E-07 |
| DMGDH | 0.000135755 | -1.502472978 | -5.810431623 | 0.001196881 |
| LINC00665 | 0.000634654 | -1.502651287 | -4.804671532 | 0.004209807 |
| COL6A1 | 1.11E-11 | -1.510116998 | -16.54453197 | 1.13E-09 |
| SLC1A2 | 0.000172741 | -1.511236153 | -5.686183637 | 0.001462301 |
| MT-RNR1 | 7.30E-10 | -1.513932583 | -13.8318957 | 4.08E-08 |
| ZNF33B | 1.32E-05 | -1.513947077 | -7.385707033 | 0.000174663 |
| NME4 | 5.59E-08 | -1.515146705 | -10.98842935 | 1.68E-06 |
| AL450998.2 | 2.81E-07 | -1.515312503 | -9.927844931 | 6.73E-06 |
| ANKRD20A4P | 0.000745857 | -1.517064003 | -4.744381222 | 0.004778558 |
| CDRT1 | 1.35E-07 | -1.518705796 | -10.43385821 | 3.57E-06 |
| AL139353.1 | 0.000837032 | -1.518758293 | -4.673611042 | 0.005222956 |
| WBP1 | 0.001884789 | -1.527364904 | -4.161667952 | 0.009977815 |
| AC078883.1 | 0.000480893 | -1.528315022 | -5.070874536 | 0.003353599 |
| CSNK1G2 | 0.000133715 | -1.530316597 | -5.928169783 | 0.001182293 |
| TRDN-AS1 | 2.38E-06 | -1.532084728 | -8.614169798 | 4.16E-05 |
| ZNF613 | 2.22E-05 | -1.532363485 | -7.131279179 | 0.000268547 |
| CLN3 | 0.00024685 | -1.536738273 | -5.54388587 | 0.001948901 |
| FOXC2 | 0.000418487 | -1.536865534 | -5.19202055 | 0.003003153 |
| LINC02671 | 0.000137347 | -1.538695541 | -5.942720085 | 0.001206819 |
| AC105339.2 | 3.09E-05 | -1.543175238 | -6.960058214 | 0.000352389 |
| AL049828.1 | 6.99E-05 | -1.55000992 | -6.440705971 | 0.000693908 |

| CLSTN3 | 7.79E-05 | -1.551001894 | -6.371897905 | 0.00075683 |
| --- | --- | --- | --- | --- |
| SGSH | 4.71E-06 | -1.551765258 | -8.266777372 | 7.34E-05 |
| STX18-AS1 | 0.000864603 | -1.553936378 | -4.759991533 | 0.005368175 |
| CNN1 | 2.83E-16 | -1.558227221 | -24.22802024 | 8.77E-14 |
| ITGA6 | 2.73E-09 | -1.560098331 | -13.35976807 | 1.26E-07 |
| WDPCP | 4.42E-06 | -1.560355294 | -8.355620106 | 6.99E-05 |
| MTRNR2L12 | 9.86E-09 | -1.56081371 | -12.49591437 | 3.78E-07 |
| TPRG1 | 2.03E-07 | -1.565211545 | -10.47402596 | 5.07E-06 |
| TNFAIP8 | 0.000388707 | -1.565746554 | -5.339786955 | 0.002833935 |
| NWD1 | 3.45E-05 | -1.566579613 | -6.990334272 | 0.000385374 |
| SNORA61 | 1.12E-05 | -1.569347017 | -7.771318255 | 0.000152878 |
| CDH13 | 0.000138176 | -1.572794885 | -6.070306361 | 0.00121294 |
| XKR6 | 0.000424126 | -1.572877875 | -5.304538745 | 0.003033637 |
| ZDHHC13 | 5.10E-07 | -1.57631408 | -9.918671968 | 1.13E-05 |
| CCDC85A | 1.46E-05 | -1.578592034 | -7.632537629 | 0.00018947 |
| HLF | 0.000335313 | -1.578996443 | -5.486302079 | 0.002501507 |
| FLVCR1 | 5.62E-14 | -1.582205141 | -20.96458543 | 1.06E-11 |
| LINC01426 | 9.17E-06 | -1.589151027 | -8.005624786 | 0.000129765 |
| COMMD10 | 0.001089096 | -1.589336575 | -4.709098989 | 0.006463526 |
| COX18 | 0.001363293 | -1.592459443 | -4.563050674 | 0.007724762 |
| AC027644.4 | 4.04E-05 | -1.592940968 | -6.999171625 | 0.000438646 |
| CSMD2 | 0.00071905 | -1.594424936 | -5.011661471 | 0.00463932 |
| SYCP2 | 0.000952181 | -1.594748523 | -4.818182851 | 0.005805476 |
| CCDC150 | 7.69E-07 | -1.595768622 | -9.757084781 | 1.62E-05 |
| ECD | 0.000931473 | -1.598076395 | -4.843497815 | 0.005702855 |
| GREB1 | 1.79E-07 | -1.600845481 | -10.79996346 | 4.59E-06 |
| ST8SIA5 | 0.000156583 | -1.604248573 | -6.104573438 | 0.001341794 |
| Z95114.1 | 1.74E-06 | -1.609222508 | -9.267273854 | 3.20E-05 |
| MOB3A | 0.000148112 | -1.610165838 | -6.165985669 | 0.001281696 |
| KCTD16 | 7.32E-10 | -1.61399899 | -14.74488601 | 4.08E-08 |
| C2orf50 | 0.001011349 | -1.615165042 | -4.8375794 | 0.006107169 |
| PAPPA2 | 7.42E-06 | -1.617313829 | -8.296104862 | 0.000107859 |
| MTND4P24 | 0.001548028 | -1.620954422 | -4.555240503 | 0.008511046 |
| RDH13 | 5.95E-05 | -1.622584403 | -6.856511289 | 0.000604156 |
| DPP6 | 1.33E-06 | -1.623969235 | -9.54215156 | 2.52E-05 |
| EFR3B | 8.12E-06 | -1.627491965 | -8.284746541 | 0.000116527 |
| LINC02614 | 1.40E-06 | -1.628363342 | -9.529744519 | 2.65E-05 |
| MTATP8P2 | 3.59E-06 | -1.628709608 | -8.868077293 | 5.88E-05 |
| ITGA7 | 3.19E-05 | -1.629289419 | -7.326192889 | 0.000360882 |
| CC2D1B | 2.25E-05 | -1.629918188 | -7.575885674 | 0.000271435 |
| LINC02427 | 0.000590536 | -1.63039671 | -5.264149875 | 0.003953842 |
| LUZP2 | 0.000735014 | -1.632750623 | -5.116558133 | 0.004728967 |
| PGM5P4 | 1.27E-05 | -1.639035115 | -8.024093138 | 0.000169728 |
| GDA | 2.55E-10 | -1.641355012 | -15.7466365 | 1.68E-08 |
| AL078581.2 | 1.99E-05 | -1.64993973 | -7.756738515 | 0.000244001 |
| RGL3 | 0.001574134 | -1.651842608 | -4.630045797 | 0.008630599 |
| SGCB | 0.000318559 | -1.655241591 | -5.788064711 | 0.002401013 |
| AC077690.1 | 0.000591233 | -1.655680469 | -5.34493577 | 0.003955617 |
| DGKI | 0.000261934 | -1.656989204 | -5.935016213 | 0.002040678 |
| TRPC3 | 1.24E-07 | -1.659131338 | -11.45780946 | 3.33E-06 |
| BANK1 | 3.48E-08 | -1.660186489 | -12.38336673 | 1.11E-06 |
| ZFP64 | 0.000770867 | -1.661905336 | -5.173545665 | 0.004901008 |

| RUNDC3A-AS1 | 3.17E-08 | -1.663072041 | -12.47164035 | 1.03E-06 |
| --- | --- | --- | --- | --- |
| BX664727.3 | 0.000583707 | -1.664695758 | -5.383301077 | 0.003919607 |
| RNF217-AS1 | 8.88E-07 | -1.66560452 | -10.07985674 | 1.84E-05 |
| BRF1 | 5.22E-05 | -1.674139512 | -7.168765585 | 0.000541423 |
| LINC01002 | 1.50E-08 | -1.674622424 | -13.09988011 | 5.46E-07 |
| CTCFL | 3.47E-05 | -1.680499795 | -7.493760188 | 0.000387499 |
| AL591845.1 | 0.000410014 | -1.683938522 | -5.703838954 | 0.002958605 |
| AF001548.2 | 6.62E-09 | -1.685217297 | -13.78355293 | 2.71E-07 |
| EHD1 | 6.06E-07 | -1.69096142 | -10.51413519 | 1.31E-05 |
| NDUFA6-DT | 1.15E-06 | -1.690962855 | -10.04394909 | 2.25E-05 |
| FP236383.4 | 2.92E-17 | -1.692769559 | -27.98976367 | 1.09E-14 |
| FP236383.5 | 2.89E-17 | -1.693030056 | -28.00192793 | 1.09E-14 |
| FP671120.7 | 3.45E-17 | -1.694841876 | -27.90163565 | 1.19E-14 |
| DLG4 | 2.87E-05 | -1.69628545 | -7.705699059 | 0.000331492 |
| AC097382.2 | 0.000308184 | -1.6963853 | -5.956330698 | 0.002339206 |
| FP671120.8 | 3.05E-17 | -1.696662148 | -28.0205628 | 1.10E-14 |
| LINC00470 | 4.31E-06 | -1.700565604 | -9.123633118 | 6.85E-05 |
| LINC02241 | 5.48E-08 | -1.703377857 | -12.36872211 | 1.66E-06 |
| CRNDE | 3.59E-07 | -1.707152777 | -11.00290912 | 8.34E-06 |
| AF107885.1 | 0.001096094 | -1.708042141 | -5.056064921 | 0.006490309 |
| TCF7 | 4.80E-06 | -1.713972941 | -9.116447637 | 7.44E-05 |
| AL138921.2 | 0.001262164 | -1.718527107 | -4.98181102 | 0.007257444 |
| MGAT3 | 0.000643952 | -1.723761762 | -5.500776593 | 0.004250779 |
| AC138123.2 | 1.37E-07 | -1.72416289 | -11.83365908 | 3.61E-06 |
| LNPK | 4.37E-10 | -1.726963016 | -16.16365565 | 2.61E-08 |
| AL356124.1 | 8.37E-05 | -1.727439734 | -7.043214319 | 0.000803844 |
| ARHGAP30 | 2.49E-06 | -1.730184834 | -9.695012118 | 4.31E-05 |
| DBN1 | 3.51E-06 | -1.730408961 | -9.438447766 | 5.77E-05 |
| CCDC39 | 2.59E-13 | -1.735331814 | -21.84162653 | 4.19E-11 |
| ZC2HC1C | 4.37E-08 | -1.736337988 | -12.77879593 | 1.36E-06 |
| AP000550.1 | 1.35E-08 | -1.739414802 | -13.69051037 | 4.98E-07 |
| PWRN1 | 8.98E-11 | -1.740302146 | -17.48457734 | 6.93E-09 |
| OBSCN | 0.000365189 | -1.742763342 | -5.990718578 | 0.002688195 |
| URGCP | 0.000704202 | -1.744302898 | -5.498570731 | 0.004562142 |
| POU2F2 | 2.65E-10 | -1.744948397 | -16.7102026 | 1.74E-08 |
| OLAH | 0.000140265 | -1.745085663 | -6.72390318 | 0.001228915 |
| AP006621.3 | 2.37E-05 | -1.746683174 | -8.078587975 | 0.000282966 |
| MTCO3P12 | 9.56E-09 | -1.751299064 | -14.04431355 | 3.68E-07 |
| AC012459.1 | 2.10E-06 | -1.756905739 | -9.975032237 | 3.75E-05 |
| AL390198.1 | 0.000460203 | -1.761425274 | -5.877964638 | 0.003235224 |
| LINC01091 | 1.14E-05 | -1.762975124 | -8.71507228 | 0.00015557 |
| CCDC151 | 0.0017956 | -1.767594823 | -4.853444969 | 0.009588944 |
| ATXN2L | 9.21E-07 | -1.771922312 | -10.69495531 | 1.90E-05 |
| GPCPD1 | 0.000923445 | -1.776796944 | -5.391848685 | 0.005660471 |
| TMEM159 | 0.00066346 | -1.778754552 | -5.653211067 | 0.004348162 |
| AAAS | 6.70E-05 | -1.78405457 | -7.446154753 | 0.000666843 |
| RN7SL1 | 3.18E-13 | -1.785839775 | -22.31940187 | 4.92E-11 |
| FHIT | 1.67E-05 | -1.787533089 | -8.538170628 | 0.000211845 |
| ZNNT1 | 5.86E-07 | -1.788860329 | -11.14808584 | 1.27E-05 |
| ATP8A2 | 0.00154814 | -1.793411767 | -5.039827446 | 0.008511046 |
| MTRNR2L8 | 7.43E-11 | -1.804081757 | -18.27389418 | 5.93E-09 |
| AC006230.1 | 5.28E-06 | -1.81086941 | -9.556346193 | 8.05E-05 |

| TRIM47 | 0.000489612 | -1.815182144 | -6.008521439 | 0.003396265 |
| --- | --- | --- | --- | --- |
| PLPP2 | 4.36E-07 | -1.817104756 | -11.55725895 | 9.84E-06 |
| SNORD89 | 1.02E-06 | -1.822242887 | -10.92163381 | 2.05E-05 |
| KANK4 | 0.000771565 | -1.834746444 | -5.710882437 | 0.004902037 |
| HBB | 2.46E-06 | -1.834808445 | -10.29133202 | 4.27E-05 |
| AC092910.3 | 0.001565337 | -1.835275285 | -5.148666819 | 0.008592672 |
| CBX4 | 1.53E-05 | -1.838295458 | -8.854082431 | 0.000196077 |
| FAM222A-AS1 | 2.05E-08 | -1.840272157 | -14.14671224 | 7.11E-07 |
| MTCO1P12 | 3.22E-10 | -1.842757113 | -17.49091131 | 2.05E-08 |
| FP671120.4 | 1.45E-10 | -1.843373792 | -18.1346106 | 1.05E-08 |
| RNA5-8SN1 | 1.45E-10 | -1.843373792 | -18.1346106 | 1.05E-08 |
| RNA5-8SN2 | 1.45E-10 | -1.843373792 | -18.1346106 | 1.05E-08 |
| RNA5-8SN3 | 1.45E-10 | -1.843373792 | -18.1346106 | 1.05E-08 |
| ZKSCAN3 | 0.000400127 | -1.849433865 | -6.284010965 | 0.002896404 |
| AC011306.1 | 5.75E-07 | -1.849540471 | -11.54214415 | 1.25E-05 |
| NABP1 | 0.001017129 | -1.855748666 | -5.553557721 | 0.006138023 |
| LRRC8C-DT | 3.66E-05 | -1.856359004 | -8.23525836 | 0.000403408 |
| KLHL11 | 0.000967714 | -1.860450874 | -5.607869507 | 0.005880576 |
| CR381653.1 | 2.13E-10 | -1.861238252 | -18.00145985 | 1.44E-08 |
| CECR2 | 2.26E-05 | -1.861858814 | -8.648665589 | 0.000272855 |
| AC079949.2 | 3.85E-05 | -1.862625985 | -8.222244655 | 0.000421492 |
| AC243772.3 | 0.001883812 | -1.864394735 | -5.08040582 | 0.009977815 |
| STARD4 | 5.26E-05 | -1.866226036 | -7.985057452 | 0.000544677 |
| AC024587.1 | 0.000817736 | -1.866284461 | -5.761941993 | 0.005127677 |
| AC073335.2 | 3.60E-05 | -1.870948103 | -8.314153359 | 0.00039787 |
| AC078799.1 | 2.57E-05 | -1.872578826 | -8.59613387 | 0.000302267 |
| RN7SL5P | 4.05E-17 | -1.876128871 | -30.75419968 | 1.37E-14 |
| SIM2 | 5.47E-07 | -1.880295349 | -11.77469053 | 1.20E-05 |
| MMADHC-DT | 6.22E-11 | -1.880950386 | -19.19722695 | 5.08E-09 |
| FO393400.1 | 2.62E-05 | -1.892088828 | -8.669685956 | 0.000307225 |
| CAD | 6.46E-06 | -1.895178495 | -9.835175705 | 9.55E-05 |
| AC104260.3 | 0.000640015 | -1.895228475 | -6.0529997 | 0.004235494 |
| PARVB | 8.04E-08 | -1.895835224 | -13.45058213 | 2.31E-06 |
| PIK3CG | 3.53E-05 | -1.89644374 | -8.442752529 | 0.000392323 |
| GABRB1 | 1.54E-07 | -1.89757177 | -12.9288028 | 4.00E-06 |
| AC087894.3 | 0.000376933 | -1.899436725 | -6.503170608 | 0.002763719 |
| FABP5P11 | 0.000880676 | -1.899526078 | -5.803401475 | 0.005442095 |
| RN7SL2 | 4.53E-15 | -1.899747015 | -27.25042512 | 1.03E-12 |
| LARP4P | 0.000131881 | -1.905334877 | -7.392350359 | 0.001170038 |
| AL627171.4 | 4.98E-15 | -1.906610927 | -27.26944915 | 1.12E-12 |
| AL133353.2 | 0.000932404 | -1.911611408 | -5.792939015 | 0.005705828 |
| SULT1C2 | 3.40E-05 | -1.919253844 | -8.5751094 | 0.000381215 |
| AP006222.1 | 9.73E-05 | -1.919359637 | -7.700393879 | 0.000905487 |
| H2BC4 | 1.48E-09 | -1.924667157 | -16.99318443 | 7.55E-08 |
| SNORD26 | 2.41E-10 | -1.931091884 | -18.57183017 | 1.61E-08 |
| RN7SL4P | 1.16E-17 | -1.937843504 | -32.81686591 | 4.62E-15 |
| AC009126.1 | 1.74E-08 | -1.941703526 | -15.06707629 | 6.13E-07 |
| AC008434.1 | 6.65E-08 | -1.942415914 | -13.94132077 | 1.95E-06 |
| GARNL3 | 0.001848819 | -1.944229751 | -5.313785179 | 0.009831825 |
| AC126773.4 | 0.000564379 | -1.944487509 | -6.316530603 | 0.003813622 |
| SH3TC2 | 6.28E-10 | -1.944592735 | -17.89465087 | 3.61E-08 |
| DYNC1I1 | 0.001876126 | -1.946741731 | -5.308254463 | 0.009949238 |

| SNORA35B | 8.13E-05 | -1.948260956 | -7.968217538 | 0.000783224 |
| --- | --- | --- | --- | --- |
| AL627309.1 | 4.98E-05 | -1.950551588 | -8.393565045 | 0.000519154 |
| LINC00607 | 3.24E-08 | -1.954758279 | -14.64097994 | 1.05E-06 |
| SYNE3 | 0.000249709 | -1.955233 | -7.043856813 | 0.001966375 |
| ASPM | 0.000156035 | -1.959120317 | -7.457935787 | 0.001338227 |
| AL591543.1 | 0.001094706 | -1.963234416 | -5.812553143 | 0.006484191 |
| PTPN7 | 2.10E-07 | -1.964339911 | -13.11801235 | 5.19E-06 |
| BCRP2 | 2.07E-08 | -1.964966174 | -15.09836629 | 7.13E-07 |
| MCOLN2 | 1.91E-05 | -1.966142644 | -9.280147953 | 0.000236225 |
| CCDC141 | 0.000297504 | -1.968530005 | -6.942035461 | 0.002270392 |
| NOTCH2 | 6.56E-12 | -1.969794985 | -22.0284498 | 7.31E-10 |
| AC091646.1 | 1.89E-05 | -1.974442236 | -9.32487803 | 0.000234859 |
| AL031772.1 | 9.55E-07 | -1.974515648 | -11.88635016 | 1.96E-05 |
| RN7SL128P | 1.80E-13 | -1.976185891 | -25.18633391 | 3.05E-11 |
| FAM89A | 2.83E-05 | -1.982313248 | -9.016166685 | 0.00032848 |
| AC027307.3 | 1.18E-06 | -1.982766673 | -11.75082458 | 2.30E-05 |
| AC004083.1 | 9.45E-05 | -1.985759137 | -7.991768675 | 0.000885951 |
| PCSK2 | 0.000421816 | -1.987957791 | -6.709113496 | 0.003020741 |
| RPS15AP10 | 0.000354992 | -1.988360892 | -6.859410594 | 0.002626883 |
| AC099506.1 | 1.21E-06 | -1.989541021 | -11.7723006 | 2.34E-05 |
| FSD2 | 0.000173916 | -1.998850521 | -7.515000699 | 0.001471564 |
| OR11G2 | 1.32E-05 | -2.001308174 | -9.764915952 | 0.000174585 |
| AL117337.1 | 0.001319095 | -2.001566819 | -5.763959974 | 0.007516251 |
| SH3TC2-DT | 4.49E-05 | -2.00903367 | -8.734168269 | 0.000477105 |
| SMAD7 | 0.001844537 | -2.011347561 | -5.499250583 | 0.009813025 |
| LINC01006 | 8.93E-09 | -2.011523194 | -16.19066125 | 3.49E-07 |
| PSD4 | 1.76E-06 | -2.012526243 | -11.58326111 | 3.22E-05 |
| LINC00884 | 3.60E-09 | -2.015068217 | -17.01389025 | 1.61E-07 |
| FAM27C | 1.43E-12 | -2.015793032 | -23.87418254 | 1.90E-10 |
| ETF1P2 | 4.86E-09 | -2.023319154 | -16.82054406 | 2.07E-07 |
| AL627309.5 | 7.05E-08 | -2.025318149 | -14.48497639 | 2.05E-06 |
| TTYH1 | 0.000743906 | -2.034778676 | -6.365768633 | 0.004769396 |
| PAK3 | 2.30E-05 | -2.039372716 | -9.460830982 | 0.000275617 |
| ARMC4 | 0.000202996 | -2.042570912 | -7.542217215 | 0.001666506 |
| AL669831.1 | 6.12E-07 | -2.044355221 | -12.70249905 | 1.32E-05 |
| FDX2 | 0.000774705 | -2.052433726 | -6.384841013 | 0.004916865 |
| AC079416.3 | 1.46E-05 | -2.052871292 | -9.924399698 | 0.000189577 |
| AC010210.1 | 3.32E-06 | -2.054619406 | -11.25663925 | 5.51E-05 |
| AC087565.1 | 3.50E-08 | -2.055944797 | -15.33002976 | 1.11E-06 |
| RELN | 5.39E-09 | -2.057059889 | -17.00781755 | 2.27E-07 |
| SKP2 | 0.000544166 | -2.062257284 | -6.731761085 | 0.003698915 |
| VNN2 | 0.000297422 | -2.064219559 | -7.279732706 | 0.002270392 |
| LINC01197 | 0.000133623 | -2.064927602 | -7.999771537 | 0.001182054 |
| AC105219.3 | 0.000311958 | -2.066160661 | -7.243760231 | 0.002362396 |
| BISPR | 3.40E-09 | -2.073859495 | -17.56191499 | 1.54E-07 |
| NUP153 | 1.14E-07 | -2.078675268 | -14.43002795 | 3.10E-06 |
| IKZF1 | 0.001276086 | -2.079750501 | -6.019047523 | 0.007314494 |
| CCDC136 | 6.26E-09 | -2.079955106 | -17.06265278 | 2.59E-07 |
| AL592429.2 | 8.96E-07 | -2.084181507 | -12.60439632 | 1.86E-05 |
| LINC00930 | 0.000129419 | -2.088678037 | -8.120783464 | 0.001152107 |
| AC011346.1 | 7.91E-12 | -2.09597199 | -23.26884237 | 8.72E-10 |
| AL445524.1 | 2.16E-08 | -2.113431223 | -16.20189517 | 7.34E-07 |

| ADCY1 | 1.48E-07 | -2.125661879 | -14.51709284 | 3.87E-06 |
| --- | --- | --- | --- | --- |
| AC087564.1 | 2.43E-08 | -2.128342926 | -16.20613572 | 8.20E-07 |
| NEBL | 5.95E-07 | -2.129208497 | -13.25567868 | 1.29E-05 |
| TMTC2 | 2.89E-06 | -2.134139799 | -11.82163372 | 4.87E-05 |
| GOLGA8F | 4.73E-05 | -2.13828163 | -9.247740351 | 0.00049969 |
| PIK3C2B | 8.31E-07 | -2.138586435 | -13.00346321 | 1.74E-05 |
| LBH | 0.000273637 | -2.14688599 | -7.648979234 | 0.002115625 |
| NR4A1 | 1.75E-10 | -2.147767094 | -20.95395258 | 1.22E-08 |
| RFPL1S | 7.47E-06 | -2.155372935 | -11.04968134 | 0.000108516 |
| RBFOX1 | 1.57E-06 | -2.157711353 | -12.52193259 | 2.93E-05 |
| AC007406.5 | 4.42E-05 | -2.169565006 | -9.447250486 | 0.000471947 |
| PIM2 | 0.000393839 | -2.172956424 | -7.39822352 | 0.002861085 |
| LINC01347 | 0.00067465 | -2.174766268 | -6.896012878 | 0.004408855 |
| UTP15 | 0.001872661 | -2.176036766 | -5.935229383 | 0.009933739 |
| MYH7 | 8.40E-06 | -2.184982636 | -11.08985006 | 0.000120154 |
| CHRFAM7A | 9.12E-05 | -2.186395879 | -8.833141605 | 0.000861722 |
| AC007938.2 | 0.000325006 | -2.1876066 | -7.630608519 | 0.002442557 |
| AP005264.5 | 3.49E-05 | -2.193319976 | -9.775373156 | 0.000389137 |
| ZNF91 | 1.41E-10 | -2.19796973 | -21.64988729 | 1.04E-08 |
| TOGARAM2 | 4.07E-08 | -2.198404423 | -16.24787506 | 1.28E-06 |
| GTF2IP20 | 1.49E-07 | -2.199895206 | -15.01865115 | 3.89E-06 |
| LNC-LBCS | 0.000261188 | -2.200004099 | -7.882716606 | 0.002039209 |
| CCDC113 | 1.21E-05 | -2.201760168 | -10.82568539 | 0.000163 |
| NTNG2 | 0.000239195 | -2.205703281 | -7.987398936 | 0.001902426 |
| SLC26A4-AS1 | 8.24E-05 | -2.206709493 | -9.011840454 | 0.000793006 |
| GALC | 0.000344507 | -2.210897848 | -7.655901182 | 0.002560696 |
| HBA2 | 6.11E-16 | -2.223957576 | -33.83519459 | 1.65E-13 |
| GOLGA6D | 1.75E-07 | -2.232445952 | -15.08297786 | 4.51E-06 |
| LINC02254 | 4.02E-07 | -2.238269953 | -14.31474002 | 9.17E-06 |
| LINC01814 | 0.001515733 | -2.243895276 | -6.326387195 | 0.008375698 |
| GSTZ1 | 0.00083621 | -2.247645222 | -6.917543339 | 0.005220245 |
| RTKN2 | 7.08E-05 | -2.251932769 | -9.345755026 | 0.000701067 |
| SGIP1 | 1.37E-07 | -2.253763156 | -15.4659867 | 3.62E-06 |
| AHSA1 | 1.09E-11 | -2.254125911 | -24.70889207 | 1.13E-09 |
| FANCC | 3.42E-08 | -2.254290848 | -16.82929173 | 1.10E-06 |
| RN7SL396P | 8.33E-07 | -2.255959475 | -13.71519542 | 1.74E-05 |
| SLC24A4 | 0.000895583 | -2.257668048 | -6.881133646 | 0.005521149 |
| CCDC163 | 0.000421929 | -2.262308984 | -7.634751109 | 0.003020741 |
| TNNT2 | 0.00156392 | -2.266332598 | -6.358843226 | 0.008587469 |
| AL109947.1 | 3.27E-10 | -2.26966471 | -21.52802982 | 2.08E-08 |
| AC020910.6 | 1.32E-06 | -2.275377993 | -13.37853419 | 2.50E-05 |
| GREB1L | 4.93E-07 | -2.287457445 | -14.42765667 | 1.10E-05 |
| PELI1 | 0.000505676 | -2.288340367 | -7.542662317 | 0.00349048 |
| DNAAF1 | 0.000227339 | -2.301804506 | -8.386222956 | 0.001824802 |
| MTCO1P40 | 4.22E-19 | -2.303227287 | -42.32102235 | 2.09E-16 |
| MTND6P22 | 1.06E-05 | -2.317764697 | -11.52916691 | 0.000146853 |
| SDR42E1 | 2.22E-06 | -2.327264573 | -13.15971329 | 3.91E-05 |
| CLEC3B | 1.86E-05 | -2.334431926 | -11.04572428 | 0.000231212 |
| SEC24B-AS1 | 0.000186177 | -2.334846793 | -8.709152724 | 0.00155303 |
| AC004974.1 | 1.83E-05 | -2.338729551 | -11.07945318 | 0.000228651 |
| STXBP5-AS1 | 2.06E-11 | -2.341325909 | -25.01805916 | 2.03E-09 |
| MCPH1-AS1 | 3.34E-05 | -2.342406293 | -10.48374045 | 0.000375675 |

| AC241377.3 | 0.000259758 | -2.344687288 | -8.406713171 | 0.002029779 |
| --- | --- | --- | --- | --- |
| AC241585.3 | 0.000479818 | -2.344687288 | -7.781838179 | 0.003348652 |
| AC134043.3 | 0.000657254 | -2.34610814 | -7.465942724 | 0.004318318 |
| SOX2-OT | 1.29E-14 | -2.347217516 | -32.59908511 | 2.72E-12 |
| Z99129.3 | 0.001440327 | -2.349858835 | -6.677215635 | 0.008065339 |
| AL731769.2 | 2.78E-11 | -2.35731421 | -24.88226132 | 2.62E-09 |
| TIMP3 | 6.00E-11 | -2.357950304 | -24.10286808 | 4.96E-09 |
| H1-4 | 8.84E-09 | -2.363285689 | -19.03265516 | 3.46E-07 |
| NME9 | 1.09E-05 | -2.366795642 | -11.74700116 | 0.000149846 |
| BPHL | 1.12E-06 | -2.367019833 | -14.08705247 | 2.21E-05 |
| LINC00941 | 0.00144624 | -2.37428347 | -6.742394282 | 0.008085149 |
| EYS | 1.14E-09 | -2.376294663 | -21.25258685 | 5.94E-08 |
| AL355472.1 | 4.82E-05 | -2.377651125 | -10.26328579 | 0.000507844 |
| AC127024.8 | 3.84E-05 | -2.381062008 | -10.51505826 | 0.00042014 |
| AC009120.5 | 1.17E-05 | -2.391227311 | -11.79234422 | 0.000159173 |
| AC092353.1 | 2.96E-09 | -2.39465636 | -20.42159318 | 1.36E-07 |
| AREL1 | 1.21E-07 | -2.416503031 | -16.71571848 | 3.26E-06 |
| HHAT | 2.86E-09 | -2.425455635 | -20.72134922 | 1.32E-07 |
| MT-TV | 9.15E-19 | -2.435546334 | -43.93422206 | 4.29E-16 |
| TNR | 3.02E-06 | -2.450408289 | -13.52583026 | 5.07E-05 |
| TMEM217 | 0.000723839 | -2.450642404 | -7.695894514 | 0.004666924 |
| SPOCK2 | 0.000878291 | -2.461964432 | -7.52465369 | 0.005429191 |
| CHEK2 | 2.54E-05 | -2.464490754 | -11.32553254 | 0.000299418 |
| ZC3H10 | 4.52E-05 | -2.477924615 | -10.76519742 | 0.000480141 |
| CELF2-DT | 9.76E-14 | -2.480902076 | -32.27774422 | 1.77E-11 |
| ERBB4 | 0.000254132 | -2.493318131 | -8.963330663 | 0.001994336 |
| LINC01550 | 4.57E-08 | -2.496119311 | -18.32265588 | 1.41E-06 |
| FGF12 | 1.51E-05 | -2.500704775 | -12.05608294 | 0.000194416 |
| MTND6P3 | 6.01E-16 | -2.503085416 | -38.09969183 | 1.65E-13 |
| SOX9-AS1 | 0.00129169 | -2.504830894 | -7.236059787 | 0.007390036 |
| RN7SL3 | 5.00E-13 | -2.517592209 | -30.96855668 | 7.62E-11 |
| LINC00499 | 8.75E-08 | -2.519492317 | -17.78233463 | 2.48E-06 |
| KCNH5 | 2.23E-13 | -2.524322914 | -31.9359596 | 3.69E-11 |
| AC073257.1 | 2.54E-07 | -2.528529459 | -16.67673325 | 6.16E-06 |
| LINC00654 | 6.17E-05 | -2.536172031 | -10.67732866 | 0.00062202 |
| AC013486.1 | 0.001482327 | -2.539652075 | -7.184818074 | 0.008248431 |
| TNNI3K | 0.001477101 | -2.542056176 | -7.195518236 | 0.008221855 |
| LINC02588 | 4.81E-16 | -2.549482352 | -39.05358912 | 1.37E-13 |
| MT-TE | 6.89E-18 | -2.556430802 | -43.87306514 | 2.93E-15 |
| SRSF3P5 | 4.79E-08 | -2.56168969 | -18.74987151 | 1.48E-06 |
| AL390957.1 | 2.02E-05 | -2.573094867 | -12.08112507 | 0.000246434 |
| LINC00534 | 1.69E-08 | -2.573579906 | -20.00141577 | 6.00E-07 |
| LINC02680 | 4.55E-08 | -2.582349321 | -18.96075371 | 1.41E-06 |
| AC027313.1 | 1.18E-08 | -2.592978102 | -20.55554256 | 4.43E-07 |
| AC008691.1 | 0.0014885 | -2.59757177 | -7.3439876 | 0.008277746 |
| LINC01608 | 2.51E-10 | -2.603838202 | -24.99687156 | 1.66E-08 |
| AL080317.1 | 9.35E-12 | -2.606277806 | -28.74551996 | 1.00E-09 |
| AC084064.1 | 3.54E-05 | -2.607089068 | -11.60477534 | 0.000392554 |
| ABCB11 | 3.07E-07 | -2.613137832 | -17.01871821 | 7.30E-06 |
| LINC02388 | 1.97E-07 | -2.6156952 | -17.53905662 | 4.94E-06 |
| GOLGA8M | 7.60E-05 | -2.621967116 | -10.79992035 | 0.000744623 |
| OR7E122P | 3.17E-09 | -2.627635225 | -22.33061283 | 1.44E-07 |

| AL591379.1 | 1.76E-10 | -2.628013096 | -25.63807924 | 1.22E-08 |
| --- | --- | --- | --- | --- |
| CACNA2D3 | 1.57E-06 | -2.632222196 | -15.27558202 | 2.93E-05 |
| MT-TS1 | 2.15E-23 | -2.649445663 | -60.05866589 | 2.07E-20 |
| LINC00535 | 1.51E-08 | -2.652135519 | -20.74452294 | 5.46E-07 |
| ETV5 | 3.82E-09 | -2.652432879 | -22.32665231 | 1.68E-07 |
| PLCXD3 | 1.29E-13 | -2.659380468 | -34.27879839 | 2.25E-11 |
| HBA1 | 2.59E-17 | -2.665859774 | -44.219024 | 1.01E-14 |
| CLLU1 | 6.40E-10 | -2.680841001 | -24.64645096 | 3.64E-08 |
| LRRC34 | 0.000258106 | -2.699138248 | -9.685055256 | 0.002020316 |
| AP000356.1 | 3.56E-16 | -2.702641577 | -41.75255933 | 1.07E-13 |
| RN7SL543P | 2.34E-10 | -2.706939129 | -26.06967287 | 1.56E-08 |
| AC240565.3 | 1.14E-06 | -2.727399558 | -16.21093154 | 2.23E-05 |
| NCOR1P4 | 6.39E-10 | -2.737517021 | -25.16998147 | 3.64E-08 |
| TRAM2-AS1 | 1.99E-12 | -2.74510822 | -32.1227137 | 2.54E-10 |
| AC113414.1 | 1.50E-05 | -2.749885281 | -13.26729452 | 0.000192948 |
| MT-TD | 5.53E-23 | -2.758552285 | -61.39770415 | 4.75E-20 |
| ASIC2 | 1.42E-05 | -2.775949302 | -13.45581198 | 0.000185248 |
| AC008467.1 | 2.54E-06 | -2.797413368 | -15.65155251 | 4.37E-05 |
| LINC00426 | 0.000462337 | -2.807836356 | -9.364250104 | 0.003247727 |
| GABRB3 | 0.00012115 | -2.808886066 | -11.00149908 | 0.001092321 |
| EFNA5 | 7.85E-06 | -2.814588085 | -14.36888406 | 0.000113196 |
| OR4D9 | 2.90E-06 | -2.834605855 | -15.6987635 | 4.88E-05 |
| LIPC | 0.001243226 | -2.839451761 | -8.249884648 | 0.007164321 |
| ZNF311 | 2.02E-10 | -2.84328328 | -27.56504208 | 1.37E-08 |
| RN7SL674P | 4.52E-11 | -2.845862414 | -29.44020417 | 4.00E-09 |
| AC097467.3 | 1.39E-06 | -2.879217734 | -16.86721999 | 2.62E-05 |
| AC005046.1 | 7.88E-08 | -2.893974295 | -20.55708545 | 2.27E-06 |
| AC010319.1 | 1.08E-06 | -2.902489423 | -17.31746634 | 2.15E-05 |
| ENTR1 | 4.51E-11 | -2.908808392 | -30.09412427 | 4.00E-09 |
| LIPA | 1.29E-07 | -2.914784715 | -20.07762092 | 3.45E-06 |
| GPR85 | 3.16E-06 | -2.916037465 | -16.04094547 | 5.26E-05 |
| CASP17P | 1.69E-08 | -2.920458662 | -22.701629 | 6.00E-07 |
| AQP1 | 3.79E-12 | -2.924029433 | -33.39511938 | 4.59E-10 |
| PPARGC1A | 4.34E-12 | -2.930376394 | -33.29618191 | 5.19E-10 |
| LINC00639 | 3.00E-07 | -2.946226849 | -19.21644882 | 7.17E-06 |
| MTCO1P53 | 3.27E-21 | -2.947324646 | -60.37686074 | 2.22E-18 |
| AC108471.3 | 1.04E-07 | -2.948950804 | -20.58676836 | 2.87E-06 |
| SLC6A1 | 5.82E-06 | -2.95605369 | -15.47456443 | 8.72E-05 |
| KDM8 | 1.41E-12 | -2.988252127 | -35.41003557 | 1.89E-10 |
| CFL1P1 | 6.22E-06 | -2.99405085 | -15.58712995 | 9.25E-05 |
| AL449043.2 | 0.000919214 | -3.004015315 | -9.121943168 | 0.005636427 |
| AC130888.1 | 6.36E-09 | -3.005802436 | -24.63678443 | 2.63E-07 |
| CKAP2LP1 | 5.43E-08 | -3.017795593 | -21.92411001 | 1.65E-06 |
| AC003102.1 | 2.14E-05 | -3.021173783 | -14.10758033 | 0.000260009 |
| C2CD3 | 9.70E-18 | -3.022140193 | -51.41593069 | 3.94E-15 |
| PLB1 | 0.000431764 | -3.024135976 | -10.17547281 | 0.003075497 |
| LAMA1 | 1.55E-09 | -3.03319411 | -26.71770039 | 7.83E-08 |
| UTS2 | 5.26E-07 | -3.037978538 | -19.07429887 | 1.16E-05 |
| MYL2 | 1.99E-06 | -3.038682912 | -17.32111384 | 3.59E-05 |
| ANKRD1 | 4.19E-06 | -3.072800473 | -16.52533154 | 6.70E-05 |
| LMO2 | 2.46E-05 | -3.078012753 | -14.18861293 | 0.000290925 |
| CDK5R1 | 1.15E-05 | -3.11152688 | -15.36373407 | 0.00015751 |

| NHLH2 | 5.93E-07 | -3.122133973 | -19.44084778 | 1.29E-05 |
| --- | --- | --- | --- | --- |
| TMEM143 | 1.79E-09 | -3.161170359 | -27.64970159 | 8.90E-08 |
| AC118755.1 | 1.59E-14 | -3.184527629 | -43.93970194 | 3.31E-12 |
| AC246785.3 | 1.05E-06 | -3.195502894 | -19.10086069 | 2.11E-05 |
| MYOZ3 | 1.12E-13 | -3.223472436 | -41.74928053 | 1.98E-11 |
| FP671120.5 | 1.03E-30 | -3.264401437 | -97.89490826 | 3.13E-27 |
| AC006148.1 | 5.18E-08 | -3.317343525 | -24.16823431 | 1.58E-06 |
| LINC00461 | 2.22E-12 | -3.333143995 | -38.84589862 | 2.78E-10 |
| FP236383.3 | 3.72E-34 | -3.335629422 | -111.5085806 | 1.70E-30 |
| GOLGA6A | 7.03E-11 | -3.360479447 | -34.11855519 | 5.64E-09 |
| SCN1A | 6.41E-12 | -3.429840001 | -38.39162154 | 7.19E-10 |
| TNNI3 | 3.28E-07 | -3.434205985 | -22.2656425 | 7.73E-06 |
| AC138207.8 | 6.06E-12 | -3.506220475 | -39.33011311 | 6.84E-10 |
| ZNF548 | 1.67E-09 | -3.597832567 | -31.57585094 | 8.36E-08 |
| LINC02754 | 2.11E-09 | -3.625925832 | -31.45962311 | 1.01E-07 |
| LINC00562 | 7.19E-22 | -4.153977502 | -87.82790007 | 5.26E-19 |
| SPATA18 | 4.47E-07 | -4.371850378 | -27.75936777 | 1.01E-05 |

DEGs KIDNEY

| gene_ID | metap | metafc | idx | fdr |
| --- | --- | --- | --- | --- |
| ELK1 | 1.04E-05 | 5.200068289 | 25.90288308 | 0.000351107 |
| ADA | 7.80E-05 | 4.893881709 | 20.10348337 | 0.001893893 |
| TNFRSF11A | 7.32E-05 | 4.086863852 | 16.90101862 | 0.00180596 |
| HPR | 1.70E-68 | 3.981389223 | 269.8208562 | 3.13E-64 |
| MFSD2A | 3.43E-28 | 3.467414543 | 95.23286753 | 3.71E-25 |
| FABP5P11 | 0.000232948 | 3.439079514 | 12.4932878 | 0.00470723 |
| HP | 2.53E-61 | 3.388967725 | 205.3624119 | 2.33E-57 |
| AC126564.1 | 6.14E-10 | 3.375979106 | 31.09939755 | 4.96E-08 |
| LEAP2 | 1.03E-07 | 3.358281329 | 23.45910226 | 5.72E-06 |
| FLYWCH2 | 2.11E-05 | 3.289202134 | 15.37712195 | 0.00064265 |
| AC106822.1 | 2.16E-06 | 3.277080418 | 18.56942986 | 8.67E-05 |
| ZNF613 | 1.47E-06 | 3.244560288 | 18.92629164 | 6.23E-05 |
| MYH7 | 3.02E-08 | 3.080972698 | 23.16916209 | 1.86E-06 |
| RNVU1-28 | 7.62E-07 | 3.019161193 | 18.47164413 | 3.45E-05 |
| GREB1L | 2.66E-12 | 2.934932308 | 33.97133219 | 3.08E-10 |
| AC004805.1 | 0.000147786 | 2.892465536 | 11.07919997 | 0.003230615 |
| PKHD1L1 | 4.71E-14 | 2.868958793 | 38.23581236 | 7.29E-12 |
| MYL2 | 0.000258178 | 2.779689141 | 9.97374688 | 0.005104842 |
| DEFA3 | 1.46E-13 | 2.70809681 | 34.75907139 | 2.10E-11 |
| LERFS | 0.000191006 | 2.692530002 | 10.01339457 | 0.004022688 |
| LINC01410 | 1.25E-05 | 2.691564364 | 13.19664442 | 0.000407096 |
| HAS2 | 1.58E-05 | 2.674093952 | 12.83907163 | 0.000507326 |
| AC244669.2 | 0.000116732 | 2.639506349 | 10.38067499 | 0.002639437 |
| AC104297.1 | 2.03E-05 | 2.452193953 | 11.50570868 | 0.000626284 |
| AC009779.3 | 8.35E-05 | 2.447790296 | 9.982509126 | 0.002001613 |
| ERI1 | 2.44E-05 | 2.437309594 | 11.24359549 | 0.00072462 |
| SLC39A14 | 3.95E-12 | 2.433409909 | 27.74876254 | 4.49E-10 |
| RNU1-28P | 3.84E-09 | 2.398680521 | 20.18730673 | 2.79E-07 |
| RNVU1-7 | 5.34E-07 | 2.370192307 | 14.86677396 | 2.49E-05 |
| CDKN2A | 4.70E-06 | 2.340237205 | 12.46906836 | 0.000173483 |
| F2RL3 | 9.61E-16 | 2.294078019 | 34.45089971 | 2.01E-13 |
| CU638689.4 | 0.000157802 | 2.291538552 | 8.712173047 | 0.003409111 |
| HAP1 | 9.58E-05 | 2.252258495 | 9.051284967 | 0.002246215 |
| S100A9 | 5.77E-26 | 2.24219833 | 56.59054993 | 4.43E-23 |
| RNVU1-29 | 4.31E-08 | 2.240154724 | 16.4994447 | 2.53E-06 |
| S100A8 | 1.92E-24 | 2.217308373 | 52.58812597 | 1.26E-21 |
| IL1RL1 | 3.06E-30 | 2.216805151 | 65.42623521 | 4.03E-27 |
| LINC01224 | 2.87E-05 | 2.196014487 | 9.97619108 | 0.000817304 |
| AL591379.1 | 8.28E-09 | 2.186332771 | 17.66982505 | 5.62E-07 |
| MT-TN | 1.32E-42 | 2.157028807 | 90.3360634 | 4.05E-39 |
| EID3 | 3.59E-15 | 2.141671253 | 30.93539538 | 6.90E-13 |
| RNU1-1 | 3.23E-07 | 2.123747678 | 13.78507035 | 1.62E-05 |
| HARBI1 | 9.70E-09 | 2.114981724 | 16.94805702 | 6.50E-07 |
| SERPINA3 | 9.85E-57 | 2.108100944 | 118.0675397 | 6.05E-53 |
| BLOC1S3 | 9.04E-05 | 2.104720416 | 8.511399616 | 0.002140628 |
| GFM2 | 2.29E-08 | 2.08531542 | 15.93093666 | 1.45E-06 |
| RPL12P41 | 3.65E-05 | 2.083016325 | 9.242584237 | 0.001009803 |
| SNX16 | 0.000224689 | 2.076738406 | 7.576811237 | 0.00458027 |

| AC096632.1 | 3.90E-05 | 2.048129149 | 9.030671888 | 0.001061866 |
| --- | --- | --- | --- | --- |
| AL049839.2 | 3.06E-55 | 2.027300348 | 110.5172179 | 1.41E-51 |
| RNU1-4 | 3.08E-06 | 2.022771982 | 11.14832265 | 0.000119001 |
| ITGA10 | 0.000176201 | 2.00077493 | 7.510890466 | 0.003758147 |
| PTGS1 | 5.76E-07 | 1.995305213 | 12.44938679 | 2.67E-05 |
| AC136944.1 | 0.000197509 | 1.993980521 | 7.386528328 | 0.004121962 |
| LUCAT1 | 3.24E-17 | 1.989670663 | 32.80866146 | 8.53E-15 |
| H19 | 5.78E-29 | 1.980311551 | 55.92090907 | 6.65E-26 |
| AC008677.1 | 6.06E-05 | 1.973399327 | 8.323072187 | 0.001546107 |
| MTRNR2L8 | 4.53E-21 | 1.941933089 | 39.50732396 | 1.85E-18 |
| MT-TA | 4.94E-26 | 1.929847643 | 48.83695322 | 4.14E-23 |
| AC105250.1 | 1.67E-12 | 1.916781823 | 22.57378065 | 2.00E-10 |
| PDIA4 | 2.76E-22 | 1.905674033 | 41.08364202 | 1.31E-19 |
| UGT1A10 | 2.16E-27 | 1.893930642 | 50.50211787 | 2.10E-24 |
| HIF1A-AS1 | 4.75E-16 | 1.887129513 | 28.91788475 | 1.09E-13 |
| PHLPP2 | 6.23E-06 | 1.883038327 | 9.802218033 | 0.000222914 |
| CD163 | 9.64E-15 | 1.871866353 | 26.23609401 | 1.71E-12 |
| HMGN1P36 | 0.000322279 | 1.85727751 | 6.485182877 | 0.00604166 |
| RNU1-27P | 1.12E-05 | 1.848661223 | 9.152853799 | 0.000370964 |
| SPP1 | 4.08E-35 | 1.836584454 | 63.15911316 | 9.40E-32 |
| MT-TD | 3.32E-31 | 1.832068545 | 55.83996989 | 5.56E-28 |
| CRYAB | 3.72E-32 | 1.815763014 | 57.06880243 | 7.61E-29 |
| UGT1A1 | 8.66E-28 | 1.814070794 | 49.09328123 | 8.87E-25 |
| MTCO1P24 | 2.81E-06 | 1.793275433 | 9.956124243 | 0.000110305 |
| AC010655.3 | 4.68E-05 | 1.787317242 | 7.738578079 | 0.001241013 |
| IGFBPL1 | 1.88E-05 | 1.784743521 | 8.434170612 | 0.000587057 |
| AC124319.3 | 4.74E-05 | 1.782054427 | 7.705234481 | 0.001254447 |
| AL031963.2 | 7.77E-05 | 1.781141053 | 7.319409537 | 0.001889798 |
| ANGPTL4 | 1.22E-12 | 1.777173933 | 21.17413425 | 1.53E-10 |
| PLEKHD1 | 0.000427012 | 1.75849437 | 5.925352694 | 0.007610224 |
| HSPA5P1 | 3.78E-12 | 1.758101997 | 20.08155168 | 4.33E-10 |
| HAVCR2 | 9.66E-08 | 1.753694148 | 12.30227618 | 5.39E-06 |
| CDRT1 | 3.81E-05 | 1.716580576 | 7.585696866 | 0.001044469 |
| BRSK1 | 2.84E-11 | 1.715276917 | 18.0908794 | 2.80E-09 |
| GCNT3 | 0.000246463 | 1.704862926 | 6.151568585 | 0.004931402 |
| TKT | 5.39E-14 | 1.695813181 | 22.50014121 | 8.08E-12 |
| CDC42-AS1 | 7.35E-06 | 1.688144089 | 8.666563856 | 0.000257427 |
| HSP90AA5P | 0.000103916 | 1.686791931 | 6.71902505 | 0.002414844 |
| RSC1A1 | 0.00015746 | 1.677137619 | 6.377866855 | 0.003405729 |
| LINC00689 | 9.41E-05 | 1.675089958 | 6.744270523 | 0.002215657 |
| HSP90AB2P | 5.75E-09 | 1.63579397 | 13.48009622 | 3.98E-07 |
| IL18R1 | 0.000110051 | 1.632528931 | 6.462214256 | 0.002525547 |
| ARMCX3-AS1 | 9.24E-05 | 1.625478592 | 6.557701062 | 0.002183049 |
| AC069287.1 | 1.88E-05 | 1.624102223 | 7.674225329 | 0.000587057 |
| AL137847.2 | 5.59E-05 | 1.61856606 | 6.883354268 | 0.001437705 |
| HMOX1 | 9.90E-13 | 1.616284027 | 19.4024428 | 1.27E-10 |
| AC010655.2 | 5.00E-05 | 1.58991215 | 6.83849433 | 0.00131209 |
| AC025647.2 | 0.000143572 | 1.578758436 | 6.067060069 | 0.003145944 |
| RNU1-2 | 2.91E-06 | 1.578001303 | 8.735867449 | 0.000113153 |
| C3 | 2.14E-13 | 1.555784296 | 19.70964935 | 2.95E-11 |
| PPP1R1B | 2.68E-05 | 1.555729831 | 7.112475861 | 0.000782808 |
| SOD2 | 5.78E-13 | 1.548992632 | 18.95659385 | 7.61E-11 |

| SERPINE1 | 2.68E-10 | 1.543827986 | 14.77730898 | 2.27E-08 |
| --- | --- | --- | --- | --- |
| ZFAND2A | 4.91E-12 | 1.537513077 | 17.38742349 | 5.49E-10 |
| MTRNR2L12 | 2.06E-16 | 1.527857661 | 23.96652432 | 4.86E-14 |
| AL137129.1 | 0.00013752 | 1.523424614 | 5.882906525 | 0.003027749 |
| MIR4485 | 1.98E-06 | 1.518897875 | 8.663843827 | 8.08E-05 |
| ZNF622 | 0.000124601 | 1.515930432 | 5.918915716 | 0.00279161 |
| MT-TQ | 1.87E-14 | 1.515866257 | 20.81075765 | 3.16E-12 |
| AC215522.2 | 1.60E-07 | 1.511986601 | 10.27355249 | 8.57E-06 |
| AC012467.2 | 5.02E-07 | 1.509719725 | 9.510247949 | 2.35E-05 |
| AC009879.1 | 0.000218542 | 1.509507514 | 5.525499184 | 0.004479749 |
| TCAF2P1 | 1.61E-05 | 1.503941738 | 7.208167995 | 0.000514579 |
| BCL6 | 9.44E-12 | 1.500751052 | 16.54550001 | 1.00E-09 |
| FJX1 | 0.000586755 | 1.4839482 | 4.795442462 | 0.009919932 |
| MFAP3 | 4.44E-05 | 1.482146156 | 6.451853122 | 0.00118807 |
| SLC25A51P4 | 0.000444938 | 1.479960042 | 4.960383109 | 0.007846233 |
| NEK10 | 7.30E-06 | 1.474627234 | 7.574532434 | 0.000256298 |
| AC022144.1 | 3.99E-09 | 1.473540815 | 12.37669604 | 2.88E-07 |
| ABCC3 | 1.28E-08 | 1.460808921 | 11.53135388 | 8.52E-07 |
| HILPDA | 7.19E-11 | 1.456771169 | 14.77683366 | 6.69E-09 |
| RUNX1 | 1.39E-05 | 1.456119401 | 7.071380674 | 0.000450074 |
| LINC01725 | 0.000495551 | 1.446343384 | 4.780037569 | 0.008623236 |
| FAM20A | 1.09E-06 | 1.441983529 | 8.599450628 | 4.74E-05 |
| TIGD5 | 9.56E-07 | 1.433933809 | 8.631409955 | 4.22E-05 |
| DEFA1 | 2.63E-13 | 1.414926238 | 17.79976405 | 3.54E-11 |
| DEFA1B | 2.63E-13 | 1.414926238 | 17.79976405 | 3.54E-11 |
| AC240565.3 | 2.10E-05 | 1.413295185 | 6.60967979 | 0.00064265 |
| TRIM58 | 0.000336854 | 1.41282214 | 4.906107521 | 0.006254306 |
| FAM177B | 1.89E-13 | 1.400239406 | 17.81735716 | 2.63E-11 |
| HSP90AA2P | 8.15E-15 | 1.385128165 | 19.51457064 | 1.49E-12 |
| AL391097.2 | 5.49E-05 | 1.384826469 | 5.899901444 | 0.001419052 |
| PLIN2 | 2.07E-10 | 1.376766755 | 13.33390364 | 1.76E-08 |
| PIWIL1 | 5.32E-05 | 1.376316538 | 5.882780252 | 0.001380152 |
| SERPINE2 | 9.67E-10 | 1.375146953 | 12.39655679 | 7.58E-08 |
| LST1 | 7.97E-05 | 1.374312002 | 5.632408931 | 0.001923255 |
| ZUP1 | 0.000311199 | 1.373599456 | 4.817160434 | 0.005906055 |
| AC092979.1 | 0.000102199 | 1.372922451 | 5.478721418 | 0.002377929 |
| AP5B1 | 0.000178162 | 1.359144997 | 5.095685945 | 0.003786816 |
| AC083862.2 | 2.85E-06 | 1.355077068 | 7.51431209 | 0.000111233 |
| CR788268.1 | 0.000503372 | 1.352336692 | 4.460156944 | 0.00872637 |
| HSPA5 | 4.03E-11 | 1.343966791 | 13.97068841 | 3.84E-09 |
| AC025627.3 | 2.27E-08 | 1.343759344 | 10.27121586 | 1.44E-06 |
| FGFR1 | 6.15E-06 | 1.326866084 | 6.914787201 | 0.000220797 |
| SLC35F6 | 1.79E-05 | 1.322158211 | 6.276366406 | 0.000563014 |
| AL592429.2 | 6.45E-07 | 1.309512532 | 8.106359913 | 2.94E-05 |
| AL805961.1 | 8.83E-05 | 1.309429226 | 5.308473535 | 0.002096912 |
| AGT | 5.61E-24 | 1.307607416 | 30.40338371 | 3.23E-21 |
| RNVU1-18 | 7.53E-07 | 1.29009136 | 7.899787244 | 3.42E-05 |
| GDF5-AS1 | 2.27E-08 | 1.286492822 | 9.834088179 | 1.44E-06 |
| UBAP1 | 0.000182747 | 1.28372739 | 4.798765709 | 0.003870872 |
| SLC16A1 | 1.25E-06 | 1.280694765 | 7.5617489 | 5.37E-05 |
| RPL4P5 | 3.76E-05 | 1.279870309 | 5.663468292 | 0.001035188 |
| QSOX2 | 0.0001504 | 1.278473177 | 4.887286487 | 0.003279964 |

| HMGB2 | 3.74E-05 | 1.277253427 | 5.655043107 | 0.001030858 |
| --- | --- | --- | --- | --- |
| NTNG2 | 4.59E-09 | 1.277210631 | 10.64953646 | 3.29E-07 |
| TTC39A | 8.14E-05 | 1.271089794 | 5.197700834 | 0.001959208 |
| C1RL-AS1 | 0.000385881 | 1.257221489 | 4.291583732 | 0.006999036 |
| ABLIM3 | 8.69E-09 | 1.251180617 | 10.08586722 | 5.84E-07 |
| AP001372.1 | 0.000207778 | 1.245909789 | 4.587937912 | 0.00429735 |
| KIR3DX1 | 0.000322018 | 1.240932799 | 4.333486141 | 0.00604166 |
| NPIPA3 | 6.12E-06 | 1.239680437 | 6.462699653 | 0.000220729 |
| PAPPA-AS1 | 3.34E-08 | 1.23916111 | 9.263825724 | 2.04E-06 |
| IL17RB | 3.98E-17 | 1.231916635 | 20.20342326 | 1.03E-14 |
| GCLM | 6.30E-06 | 1.228207893 | 6.387620664 | 0.000224503 |
| GBP3 | 5.09E-07 | 1.218329653 | 7.667493021 | 2.38E-05 |
| MTCO1P22 | 5.22E-07 | 1.212482253 | 7.616894422 | 2.44E-05 |
| MLKL | 0.000553656 | 1.203521018 | 3.919578636 | 0.009482139 |
| FTH1P23 | 0.000124826 | 1.197090949 | 4.673079489 | 0.00279161 |
| RNU1-3 | 2.05E-06 | 1.196909662 | 6.809228354 | 8.27E-05 |
| AZGP1 | 1.69E-06 | 1.193552738 | 6.889198243 | 7.06E-05 |
| C4B | 1.31E-11 | 1.19301309 | 12.98235869 | 1.34E-09 |
| PTPN1 | 1.51E-08 | 1.192954921 | 9.329594067 | 9.88E-07 |
| EEF1A1P9 | 9.24E-07 | 1.191840663 | 7.191905949 | 4.09E-05 |
| TNNT2 | 8.29E-09 | 1.188909656 | 9.608186507 | 5.62E-07 |
| AC114801.3 | 4.81E-24 | 1.183904761 | 27.60584103 | 2.86E-21 |
| C4A | 1.29E-11 | 1.183451343 | 12.8859749 | 1.33E-09 |
| HSP90AA1 | 1.33E-14 | 1.177684679 | 16.34115774 | 2.31E-12 |
| CTSA | 6.47E-12 | 1.176665823 | 13.16606832 | 6.93E-10 |
| RPL26P35 | 0.000290117 | 1.174687792 | 4.155371284 | 0.005586504 |
| AP000295.1 | 2.27E-05 | 1.157707559 | 5.37629047 | 0.000682507 |
| ADAMTS1 | 2.73E-07 | 1.140664226 | 7.48757214 | 1.38E-05 |
| MTATP6P2 | 2.87E-21 | 1.140269507 | 23.42376476 | 1.20E-18 |
| AC119427.2 | 0.000133975 | 1.139237593 | 4.412241491 | 0.00297098 |
| VCAM1 | 4.07E-11 | 1.138959643 | 11.83465121 | 3.84E-09 |
| AKAP12 | 8.16E-14 | 1.13579488 | 14.86548694 | 1.21E-11 |
| GBP2 | 1.94E-06 | 1.134474031 | 6.480100901 | 7.97E-05 |
| TNC | 1.23E-07 | 1.132806853 | 7.827046746 | 6.74E-06 |
| LACTB2-AS1 | 3.91E-05 | 1.13128129 | 4.986387418 | 0.001062953 |
| H1-4 | 2.20E-15 | 1.12905412 | 16.55019126 | 4.30E-13 |
| HIF1A | 4.55E-09 | 1.126464817 | 9.396433213 | 3.28E-07 |
| SLC1A1 | 1.57E-08 | 1.102016908 | 8.599243021 | 1.02E-06 |
| FAM241A | 4.40E-05 | 1.101733902 | 4.799693886 | 0.001180403 |
| AC245060.5 | 6.98E-08 | 1.095031964 | 7.836240856 | 3.99E-06 |
| AL133230.1 | 6.26E-12 | 1.083040253 | 12.133705 | 6.79E-10 |
| HSP90B1 | 5.65E-12 | 1.080127083 | 12.14936052 | 6.20E-10 |
| CFB | 2.34E-07 | 1.079671867 | 7.159919224 | 1.20E-05 |
| POLR1C | 2.48E-06 | 1.074077381 | 6.020975158 | 9.91E-05 |
| AL353729.1 | 1.83E-09 | 1.07398123 | 9.38344253 | 1.38E-07 |
| LDB1 | 2.11E-05 | 1.064972717 | 4.979882665 | 0.00064265 |
| PLTP | 1.29E-11 | 1.064952363 | 11.59648777 | 1.33E-09 |
| CALU | 5.95E-06 | 1.063842561 | 5.55906845 | 0.000215898 |
| SEC24D | 0.000383189 | 1.06189633 | 3.628060589 | 0.006963921 |
| AL133230.2 | 2.23E-07 | 1.056628621 | 7.028578166 | 1.15E-05 |
| AC126283.2 | 3.83E-08 | 1.055791199 | 7.830753818 | 2.29E-06 |
| SLC9B1 | 3.96E-07 | 1.051881239 | 6.734107048 | 1.91E-05 |

| AKR1C1 | 2.66E-07 | 1.050643919 | 6.908770824 | 1.35E-05 |
| --- | --- | --- | --- | --- |
| CFI | 6.96E-09 | 1.049196426 | 8.558558037 | 4.75E-07 |
| IL10RB | 7.47E-05 | 1.048680425 | 4.327607087 | 0.001827959 |
| TGIF2-RAB5IF | 3.87E-06 | 1.047983673 | 5.671507914 | 0.000145106 |
| CLLU1 | 0.000277995 | 1.041507198 | 3.703560569 | 0.005400864 |
| FPR1 | 0.000433342 | 1.039812493 | 3.497065733 | 0.007715575 |
| MFHAS1 | 2.81E-06 | 1.038745987 | 5.766816127 | 0.000110305 |
| AC104041.1 | 5.57E-05 | 1.035812536 | 4.40615914 | 0.001436657 |
| AL731769.2 | 0.000184758 | 1.033951157 | 3.860151079 | 0.003904486 |
| NAMPTP1 | 0.000159795 | 1.033819686 | 3.924831924 | 0.003440067 |
| AL591888.1 | 1.76E-05 | 1.029500497 | 4.89427608 | 0.000554999 |
| FLVCR1 | 2.44E-05 | 1.024742929 | 4.726397471 | 0.00072462 |
| AC012066.1 | 2.92E-05 | 1.023215026 | 4.640553276 | 0.000828604 |
| TTLL7 | 0.000113729 | 1.022178333 | 4.031604219 | 0.002581025 |
| MT1M | 0.000415915 | 1.019077988 | 3.445497544 | 0.007441252 |
| AL360270.2 | 0.000195855 | 1.018779204 | 3.777699276 | 0.004092087 |
| TNFAIP2 | 1.45E-08 | 1.018214079 | 7.982420226 | 9.56E-07 |
| SULF2 | 6.10E-05 | 1.017229498 | 4.287090843 | 0.001553324 |
| SCIMP | 1.11E-06 | 1.010803132 | 6.018604131 | 4.83E-05 |
| ZBTB16 | 0.000269481 | 1.010585309 | 3.607255815 | 0.0052886 |
| NFIL3 | 2.03E-05 | 1.010553734 | 4.741584946 | 0.000626284 |
| SELENOS | 0.000176536 | 1.00780639 | 3.782464342 | 0.00376094 |
| LMOD1 | 6.30E-05 | -1.002042989 | -4.209058881 | 0.001593211 |
| SLC16A7 | 7.51E-05 | -1.002305234 | -4.133628401 | 0.001833586 |
| CALM3 | 0.000199007 | -1.005482427 | -3.721422164 | 0.004143849 |
| AC018738.1 | 0.000112145 | -1.015858499 | -4.012866262 | 0.002560843 |
| C14orf132 | 0.000200982 | -1.021798366 | -3.777428623 | 0.004180238 |
| RIDA | 1.08E-05 | -1.027194235 | -5.101305471 | 0.000360817 |
| RPS28P7 | 4.96E-05 | -1.028831348 | -4.428820742 | 0.001305179 |
| CYP4F3 | 1.83E-10 | -1.032854593 | -10.05838887 | 1.58E-08 |
| FBP1 | 4.18E-12 | -1.033058454 | -11.75498662 | 4.73E-10 |
| CYP3A5 | 2.22E-07 | -1.041609483 | -6.930204819 | 1.15E-05 |
| RPL15P3 | 8.96E-06 | -1.04307833 | -5.265035698 | 0.000309274 |
| AC078817.1 | 3.51E-08 | -1.048837972 | -7.819244102 | 2.13E-06 |
| AC090559.1 | 0.000242934 | -1.054300045 | -3.810780041 | 0.004876673 |
| PIPOX | 6.07E-05 | -1.055801712 | -4.452421637 | 0.001546107 |
| RPS23 | 8.21E-05 | -1.059258474 | -4.327615782 | 0.001970618 |
| MUC1 | 6.53E-05 | -1.05966258 | -4.435021642 | 0.001642744 |
| XIST | 0.000138619 | -1.060304738 | -4.090844632 | 0.003048288 |
| MXRA5 | 4.34E-05 | -1.065116099 | -4.646727811 | 0.001167182 |
| CD81 | 0.000559206 | -1.065818752 | -3.466498965 | 0.009568289 |
| GTF3C5 | 0.000402257 | -1.069894968 | -3.632825008 | 0.007253213 |
| KCTD12 | 0.000243307 | -1.073941615 | -3.88105943 | 0.004878843 |
| A2M | 0.00040759 | -1.074448739 | -3.642141087 | 0.007335027 |
| PTPRC | 1.39E-06 | -1.077003547 | -6.307024473 | 5.93E-05 |
| SORL1 | 4.78E-06 | -1.084742176 | -5.771015265 | 0.000176334 |
| AC021723.1 | 0.000107998 | -1.087936533 | -4.315391351 | 0.002490851 |
| CYP1B1 | 1.01E-05 | -1.090127158 | -5.445096847 | 0.000342108 |
| AL355032.1 | 0.000100389 | -1.094073399 | -4.37445093 | 0.002347665 |
| SERPING1 | 2.02E-06 | -1.113727324 | -6.341558415 | 8.20E-05 |
| CLMP | 2.06E-10 | -1.114300314 | -10.79351261 | 1.76E-08 |
| ALDH4A1 | 9.90E-08 | -1.115412241 | -7.812624691 | 5.50E-06 |

| RPL26P27 | 1.28E-08 | -1.121843461 | -8.85425358 | 8.52E-07 |
| --- | --- | --- | --- | --- |
| ESR1 | 0.000570289 | -1.122695088 | -3.641916117 | 0.009730822 |
| MSRA | 9.52E-06 | -1.125578663 | -5.65183322 | 0.000324909 |
| MT-TF | 6.48E-23 | -1.127190075 | -25.01053059 | 3.23E-20 |
| TOMM7 | 2.19E-05 | -1.12730272 | -5.25326299 | 0.000659794 |
| MNDA | 3.43E-09 | -1.149967677 | -9.734111019 | 2.52E-07 |
| AC107983.1 | 2.64E-05 | -1.152518437 | -5.276973063 | 0.000771778 |
| UNC5B | 5.57E-05 | -1.165974026 | -4.960676197 | 0.001436303 |
| AL121845.3 | 4.12E-05 | -1.170710455 | -5.133469958 | 0.001112088 |
| ESPN | 0.000315033 | -1.177254454 | -4.122326379 | 0.00595428 |
| APBB1IP | 0.000112718 | -1.178848278 | -4.654102265 | 0.002567567 |
| MT1E | 1.19E-10 | -1.18091507 | -11.72119954 | 1.05E-08 |
| RNA5SP498 | 0.000240983 | -1.192725955 | -4.315299563 | 0.004848065 |
| TMEM176A | 1.70E-07 | -1.201720987 | -8.134646578 | 9.04E-06 |
| NUP50-DT | 9.34E-07 | -1.201749129 | -7.245994297 | 4.13E-05 |
| JAML | 2.74E-05 | -1.206238451 | -5.503713541 | 0.000794322 |
| MRPS16 | 1.48E-06 | -1.206973574 | -7.037293401 | 6.26E-05 |
| SRSF5 | 0.000575301 | -1.21614358 | -3.940432525 | 0.009798199 |
| RGS5 | 4.13E-07 | -1.226124624 | -7.827650228 | 1.98E-05 |
| PXMP4 | 9.32E-05 | -1.233094869 | -4.970091248 | 0.002199096 |
| MYH11 | 0.000274259 | -1.24491901 | -4.434200968 | 0.005348198 |
| CREB3L1 | 1.50E-09 | -1.256311811 | -11.08615867 | 1.14E-07 |
| RYR2 | 1.44E-13 | -1.260623324 | -16.18666304 | 2.10E-11 |
| S100A6 | 0.000311847 | -1.277492467 | -4.478963523 | 0.005912257 |
| NDRG2 | 0.000124057 | -1.281020329 | -5.004151029 | 0.002784556 |
| ABHD3 | 2.35E-06 | -1.289139493 | -7.256897014 | 9.41E-05 |
| NDUFAB1 | 0.0001954 | -1.291129477 | -4.788897749 | 0.004091471 |
| CAPN3 | 0.000154156 | -1.293712514 | -4.93168369 | 0.003346036 |
| ATXN7L1 | 0.000182074 | -1.293921543 | -4.838946503 | 0.003861053 |
| FUS | 0.000224415 | -1.295351509 | -4.72667068 | 0.004579754 |
| COL15A1 | 4.02E-05 | -1.298515197 | -5.70802709 | 0.001089328 |
| ADAM28 | 0.00027757 | -1.298854579 | -4.619541415 | 0.005400864 |
| PPARGC1A | 6.33E-06 | -1.299917189 | -6.757875294 | 0.000225139 |
| COA3 | 6.64E-05 | -1.30747919 | -5.462316643 | 0.001662853 |
| CCND2 | 0.000156496 | -1.307632317 | -4.976188793 | 0.003388857 |
| HMGB1P8 | 1.03E-10 | -1.310253179 | -13.08753412 | 9.23E-09 |
| ABHD14A-ACY1 | 5.17E-10 | -1.325138949 | -12.30573646 | 4.24E-08 |
| TMEM52B | 2.79E-09 | -1.32858554 | -11.36490319 | 2.07E-07 |
| FGFR3 | 3.19E-06 | -1.337580411 | -7.352366622 | 0.000122058 |
| PROM1 | 0.00029242 | -1.337684258 | -4.727367151 | 0.005615949 |
| TSTD1 | 0.000371838 | -1.343377968 | -4.607311865 | 0.006788806 |
| HLA-DPB1 | 0.000454877 | -1.347348853 | -4.502982282 | 0.00799462 |
| PER1 | 0.00038377 | -1.355751617 | -4.631150527 | 0.006967608 |
| FOXC1 | 2.13E-05 | -1.356690276 | -6.337626591 | 0.000646996 |
| BHLHE40 | 6.50E-08 | -1.364561617 | -9.806901862 | 3.75E-06 |
| GPD1 | 9.60E-08 | -1.367900968 | -9.599676779 | 5.38E-06 |
| SLC7A8 | 1.93E-17 | -1.369448293 | -22.89039569 | 5.30E-15 |
| SLC8A1-AS1 | 3.81E-05 | -1.373867978 | -6.070816768 | 0.001044469 |
| BCAT2 | 2.23E-14 | -1.374247736 | -18.76068709 | 3.70E-12 |
| NPR3 | 3.67E-06 | -1.382570689 | -7.515091111 | 0.000138222 |
| MAP2 | 0.000119586 | -1.382666784 | -5.423259455 | 0.002700658 |
| AC090607.4 | 6.91E-09 | -1.383414077 | -11.28937251 | 4.73E-07 |

| HES1 | 2.27E-05 | -1.397592066 | -6.491304245 | 0.000682484 |
| --- | --- | --- | --- | --- |
| C7 | 1.08E-06 | -1.399365483 | -8.349211214 | 4.72E-05 |
| IGFBP7 | 1.55E-07 | -1.410226166 | -9.602612555 | 8.34E-06 |
| ACTA2-AS1 | 1.03E-09 | -1.410730405 | -12.67986918 | 8.02E-08 |
| AC008670.1 | 6.31E-10 | -1.424284234 | -13.10292718 | 5.08E-08 |
| SUSD2 | 0.00033423 | -1.426903202 | -4.959851609 | 0.006227686 |
| ALDOB | 9.57E-40 | -1.431877968 | -55.87064808 | 2.52E-36 |
| NDUFS3 | 0.000497844 | -1.437773619 | -4.748832161 | 0.008648466 |
| COL1A2 | 3.27E-06 | -1.454536633 | -7.978380425 | 0.000124842 |
| HMGB1P11 | 2.25E-12 | -1.481501865 | -17.2568671 | 2.66E-10 |
| IFITM1 | 0.000273251 | -1.486392954 | -5.296668373 | 0.00533985 |
| CPE | 0.000370848 | -1.494903024 | -5.128718635 | 0.006779757 |
| CKB | 4.82E-06 | -1.498951511 | -7.970216337 | 0.000177193 |
| SLC20A1 | 7.05E-05 | -1.501262164 | -6.233308069 | 0.001748486 |
| PLCXD3 | 0.000292288 | -1.506115272 | -5.322896315 | 0.005615949 |
| MAL2 | 5.59E-05 | -1.516163122 | -6.447164223 | 0.001437705 |
| COL3A1 | 4.82E-08 | -1.521019044 | -11.12860432 | 2.81E-06 |
| EHHADH | 2.82E-05 | -1.532928615 | -6.975372551 | 0.000805815 |
| AGMAT | 9.09E-15 | -1.558235742 | -21.87984629 | 1.63E-12 |
| AL137127.1 | 0.000219603 | -1.562940156 | -5.717801622 | 0.004491239 |
| ENGASE | 0.000153998 | -1.565728371 | -5.969315309 | 0.003346036 |
| METTL7A | 2.76E-11 | -1.574892773 | -16.62874296 | 2.74E-09 |
| TREH | 0.000318307 | -1.58201485 | -5.532550056 | 0.006001793 |
| SNAP25 | 7.45E-05 | -1.58429878 | -6.540133034 | 0.001827959 |
| CXCL12 | 8.12E-05 | -1.587390842 | -6.492938743 | 0.00195657 |
| ASS1 | 9.64E-12 | -1.588302326 | -17.49681603 | 1.01E-09 |
| DNPH1 | 2.09E-07 | -1.59079264 | -10.62719086 | 1.09E-05 |
| AC012651.1 | 2.55E-07 | -1.594841683 | -10.51688171 | 1.30E-05 |
| SSPN | 0.000153035 | -1.612788813 | -6.153125035 | 0.003329558 |
| FBLN5 | 1.69E-05 | -1.62122099 | -7.738694548 | 0.000533557 |
| GK | 6.58E-07 | -1.659920135 | -10.26161235 | 2.99E-05 |
| AL590867.2 | 0.000198112 | -1.668842404 | -6.179872021 | 0.004129875 |
| BTG2 | 0.00010867 | -1.685848631 | -6.682520748 | 0.002503208 |
| ITGA7 | 0.000124821 | -1.688959409 | -6.59321409 | 0.00279161 |
| SLC4A1 | 1.09E-07 | -1.689957381 | -11.76530972 | 6.01E-06 |
| PER3 | 2.43E-05 | -1.707244163 | -7.877057935 | 0.00072462 |
| AIF1 | 0.000219834 | -1.712619253 | -6.264599435 | 0.004491239 |
| DNAJC9-AS1 | 7.21E-10 | -1.717618637 | -15.70215689 | 5.73E-08 |
| GATM | 1.37E-18 | -1.723074643 | -30.7821525 | 4.19E-16 |
| TIMP3 | 1.97E-05 | -1.755470103 | -8.258638789 | 0.000612597 |
| SFRP1 | 4.87E-07 | -1.764613421 | -11.13857687 | 2.29E-05 |
| ATF3 | 9.01E-18 | -1.773042629 | -30.22157863 | 2.64E-15 |
| RAP1GAP | 0.000426463 | -1.774387567 | -5.979896848 | 0.0076078 |
| AC008686.1 | 0.000337016 | -1.774678934 | -6.162306052 | 0.006254306 |
| COL6A1 | 6.53E-05 | -1.778128732 | -7.441120906 | 0.001642744 |
| GPAT3 | 1.58E-07 | -1.783967848 | -12.13260282 | 8.47E-06 |
| AC079328.2 | 9.00E-05 | -1.785229824 | -7.222396108 | 0.002135098 |
| CELF4 | 0.000234632 | -1.786571936 | -6.484564985 | 0.00473581 |
| SPOCK2 | 0.000583945 | -1.790468784 | -5.789709772 | 0.009908787 |
| GOLGA8CP | 7.90E-11 | -1.804787595 | -18.23293113 | 7.29E-09 |
| MS4A7 | 0.000444327 | -1.819630004 | -6.099941188 | 0.007842963 |
| CDH11 | 3.21E-06 | -1.821335006 | -10.00665967 | 0.000122545 |

| HBB | 4.90E-12 | -1.828895322 | -20.68407551 | 5.49E-10 |
| --- | --- | --- | --- | --- |
| AC025031.3 | 0.000169096 | -1.845226326 | -6.959948164 | 0.003631817 |
| EGR1 | 2.42E-11 | -1.846892934 | -19.60859111 | 2.41E-09 |
| TCL6 | 9.58E-05 | -1.861567669 | -7.480903869 | 0.002246215 |
| DCXR | 1.11E-05 | -1.893562506 | -9.381001803 | 0.000369687 |
| MAL | 4.15E-08 | -1.896792977 | -14.00143574 | 2.45E-06 |
| MXRA8 | 2.01E-06 | -1.90331623 | -10.84107517 | 8.19E-05 |
| AC068631.1 | 9.54E-06 | -1.922996488 | -9.654440678 | 0.000324909 |
| ABCA8 | 3.45E-05 | -1.93381301 | -8.628823897 | 0.000963511 |
| AP000757.1 | 4.08E-06 | -1.941090579 | -10.46099264 | 0.000152545 |
| FLNA | 5.10E-08 | -1.968250951 | -14.35375107 | 2.94E-06 |
| CARNMT1-AS1 | 2.84E-06 | -2.00150048 | -11.10073798 | 0.000111233 |
| MIR7705 | 0.000206495 | -2.034297233 | -7.496569085 | 0.004277793 |
| AC022509.1 | 0.000310751 | -2.042889452 | -7.165613274 | 0.005906055 |
| EGF | 7.46E-05 | -2.054874723 | -8.481580258 | 0.001827959 |
| TMTC2 | 2.04E-10 | -2.058996862 | -19.9542746 | 1.75E-08 |
| CADM2 | 6.13E-07 | -2.082244543 | -12.93654369 | 2.81E-05 |
| CAMK2A | 6.79E-06 | -2.085247268 | -10.77620079 | 0.000240332 |
| AC019080.1 | 5.39E-09 | -2.096552187 | -17.33463712 | 3.77E-07 |
| NR4A1 | 2.63E-20 | -2.100139804 | -41.12204637 | 9.88E-18 |
| NRXN1 | 4.69E-07 | -2.12047842 | -13.42025586 | 2.21E-05 |
| A2M-AS1 | 1.33E-10 | -2.128607492 | -21.01910128 | 1.17E-08 |
| LTBP1 | 9.82E-12 | -2.129862793 | -23.44506619 | 1.02E-09 |
| DCN | 5.72E-11 | -2.149513985 | -22.0173315 | 5.35E-09 |
| PTN | 4.04E-11 | -2.172329211 | -22.57852245 | 3.84E-09 |
| MRC2 | 7.89E-07 | -2.184425971 | -13.33088786 | 3.57E-05 |
| AC002398.2 | 3.82E-10 | -2.196250283 | -20.68445741 | 3.16E-08 |
| HSPB6 | 1.30E-09 | -2.196250283 | -19.5149839 | 9.99E-08 |
| WNK4 | 8.82E-11 | -2.197462493 | -22.09401036 | 8.05E-09 |
| FBLN1 | 6.13E-09 | -2.210339097 | -18.15314613 | 4.21E-07 |
| MOXD1 | 5.02E-09 | -2.228395169 | -18.49318331 | 3.56E-07 |
| ECRG4 | 0.000121598 | -2.264337193 | -8.865044006 | 0.002742734 |
| COL14A1 | 1.67E-06 | -2.27607678 | -13.14667461 | 7.01E-05 |
| AF001548.2 | 5.29E-05 | -2.292341652 | -9.804129218 | 0.001373809 |
| DRICH1 | 2.81E-09 | -2.300246763 | -19.67178282 | 2.07E-07 |
| CEP170B | 3.64E-05 | -2.325574368 | -10.32423864 | 0.00100593 |
| SPON2 | 5.34E-10 | -2.412917625 | -22.37432843 | 4.35E-08 |
| LTB | 7.12E-05 | -2.434871951 | -10.09917568 | 0.001762714 |
| TRAC | 5.94E-07 | -2.441193774 | -15.19877606 | 2.73E-05 |
| SYT1 | 3.63E-11 | -2.442136149 | -25.4975162 | 3.50E-09 |
| SCARNA9 | 5.58E-09 | -2.467438285 | -20.36536303 | 3.88E-07 |
| ADH1B | 3.06E-11 | -2.471241864 | -25.98431772 | 2.98E-09 |
| HLA-DPA1 | 6.11E-09 | -2.520705902 | -20.70475821 | 4.21E-07 |
| NDN | 0.000281673 | -2.546299694 | -9.040013466 | 0.005458114 |
| CXCR4 | 0.000230871 | -2.552966023 | -9.284193115 | 0.004690734 |
| PAQR5 | 3.06E-24 | -2.694699634 | -63.36571007 | 1.94E-21 |
| GPR183 | 0.000113478 | -2.712072178 | -10.69936108 | 0.002581025 |
| SCG3 | 0.000244491 | -2.712991549 | -9.798613277 | 0.004897257 |
| AP003499.4 | 1.05E-09 | -2.733209064 | -24.54540688 | 8.07E-08 |
| AP003396.5 | 4.03E-11 | -2.811968014 | -29.22891675 | 3.84E-09 |
| AL117190.2 | 0.000273831 | -2.821039219 | -10.04999967 | 0.005345513 |
| THY1 | 1.21E-12 | -2.858571886 | -34.06486819 | 1.53E-10 |

| KNG1 | 2.80E-19 | -3.018233773 | -55.99727218 | 9.38E-17 |
| --- | --- | --- | --- | --- |
| ANGPT1 | 2.26E-10 | -3.09939657 | -29.89368037 | 1.92E-08 |
| ISLR | 0.000288222 | -3.164792829 | -11.20423175 | 0.005555804 |
| IGKC | 8.34E-21 | -3.662826319 | -73.54620359 | 3.34E-18 |
| SLC12A1 | 3.11E-27 | -3.85003258 | -102.0514858 | 2.87E-24 |

DEGs LUNG

| gene_ID | metap | metafc | idx | fdr |
| --- | --- | --- | --- | --- |
| GDNF-AS1 | 7.75E-12 | 3.902826993 | 43.36277363 | 3.51E-10 |
| AC007622.2 | 5.35E-10 | 3.890705676 | 36.0747227 | 1.71E-08 |
| AC034111.1 | 3.12E-08 | 3.890705676 | 29.20468099 | 6.82E-07 |
| TCN2 | 1.47E-09 | 3.877305158 | 34.24505055 | 4.25E-08 |
| KIF5A | 7.27E-15 | 3.811662565 | 53.89036951 | 5.47E-13 |
| FES | 2.61E-12 | 3.780075118 | 43.78800713 | 1.28E-10 |
| CD163 | 4.12E-75 | 3.617710712 | 269.1023567 | 3.80E-71 |
| AC009951.4 | 1.03E-12 | 3.494113528 | 41.87716183 | 5.45E-11 |
| AC095038.3 | 0.000805936 | 3.435440934 | 10.62822171 | 0.0048464 |
| MIR210HG | 0.000788754 | 3.404022863 | 10.5628823 | 0.004761726 |
| SPP1 | 1.47E-16 | 3.396396095 | 53.77681673 | 1.49E-14 |
| AC006148.1 | 1.82E-07 | 3.305723724 | 22.28137152 | 3.33E-06 |
| SH3TC2-DT | 0.000828678 | 3.212104949 | 9.898468543 | 0.004957269 |
| TM4SF18 | 2.85E-06 | 3.211388861 | 17.80966236 | 3.77E-05 |
| RN7SL543P | 1.93E-14 | 3.204065474 | 43.94043349 | 1.36E-12 |
| NPL | 0.000248328 | 3.095186958 | 11.15807102 | 0.001792181 |
| PNMA8C | 0.000806411 | 3.084588883 | 9.542001005 | 0.004847677 |
| LINC02588 | 1.15E-28 | 3.084497234 | 86.18207105 | 5.71E-26 |
| SEMA6B | 0.001808004 | 3.079561495 | 8.446623061 | 0.009560746 |
| TMCC1-AS1 | 2.07E-05 | 3.06959023 | 14.37968299 | 0.000213526 |
| AC124242.1 | 6.67E-05 | 3.062371289 | 12.78716825 | 0.00058146 |
| AL772363.1 | 1.45E-05 | 3.05549672 | 14.78510978 | 0.000156981 |
| ST3GAL6-AS1 | 4.08E-05 | 2.970499761 | 13.03986049 | 0.000382366 |
| AC084064.1 | 3.60E-25 | 2.955443246 | 72.24332837 | 1.16E-22 |
| AC098934.2 | 4.87E-09 | 2.905397077 | 24.1517458 | 1.26E-07 |
| FPR1 | 9.90E-64 | 2.865811937 | 180.5589767 | 3.65E-60 |
| AP000356.1 | 2.17E-24 | 2.856406749 | 67.58984113 | 6.17E-22 |
| FAM222A-AS1 | 1.97E-17 | 2.849440134 | 47.6006176 | 2.26E-15 |
| LINC00562 | 1.05E-20 | 2.819351962 | 56.33232728 | 1.87E-18 |
| LINC00941 | 2.25E-08 | 2.791668389 | 21.3507362 | 5.09E-07 |
| LINC00461 | 4.56E-17 | 2.779221406 | 45.41589675 | 4.85E-15 |
| RNF144A-AS1 | 0.000102904 | 2.77226541 | 11.05459661 | 0.000837828 |
| AC010624.5 | 0.000430712 | 2.759045978 | 9.286434252 | 0.002861832 |
| AC066613.2 | 3.19E-15 | 2.750029801 | 39.86679034 | 2.54E-13 |
| RNU6-4P | 0.000884401 | 2.747430172 | 8.388867608 | 0.005236213 |
| NTNG2 | 3.31E-33 | 2.741039298 | 89.02838239 | 3.39E-30 |
| AL121929.4 | 6.24E-05 | 2.73378143 | 11.4945518 | 0.000550862 |
| AC004083.1 | 2.24E-07 | 2.733098261 | 18.17287035 | 4.03E-06 |
| AP000355.1 | 3.80E-42 | 2.727803756 | 112.9869154 | 8.75E-39 |
| KDM8 | 3.90E-16 | 2.708915196 | 41.74212908 | 3.66E-14 |
| XRCC2 | 1.38E-05 | 2.693472139 | 13.09336643 | 0.000150009 |
| AC087164.1 | 0.000251377 | 2.679918889 | 9.646835108 | 0.001810642 |
| ST8SIA5 | 0.000260441 | 2.673483262 | 9.582541279 | 0.001865717 |
| SLC30A2 | 2.99E-07 | 2.663397126 | 17.37666882 | 5.24E-06 |
| SCN1B | 4.90E-16 | 2.657363441 | 40.68362965 | 4.49E-14 |
| AL096794.1 | 1.85E-13 | 2.648486453 | 33.72389972 | 1.09E-11 |
| TCAF2C | 2.16E-11 | 2.648113771 | 28.24206026 | 8.92E-10 |
| AC092118.1 | 4.82E-07 | 2.626867924 | 16.59370673 | 7.94E-06 |

| CABP4 | 2.39E-06 | 2.619720547 | 14.72904945 | 3.24E-05 |
| --- | --- | --- | --- | --- |
| IL17RA | 1.29E-30 | 2.613629696 | 78.1202488 | 7.66E-28 |
| PIM1 | 2.18E-07 | 2.612740895 | 17.40340409 | 3.92E-06 |
| ZBTB6 | 0.000541145 | 2.606738824 | 8.515398114 | 0.003475927 |
| LY6E-DT | 1.63E-08 | 2.604934244 | 20.28581385 | 3.80E-07 |
| AL731769.2 | 8.39E-29 | 2.561291303 | 71.91170846 | 4.29E-26 |
| ARL11 | 6.42E-23 | 2.517448371 | 55.86773283 | 1.58E-20 |
| AL031846.2 | 1.70E-09 | 2.503037042 | 21.95185954 | 4.82E-08 |
| AC010328.1 | 4.65E-07 | 2.500206355 | 15.83355438 | 7.70E-06 |
| IL1B | 7.03E-45 | 2.496900567 | 110.2462551 | 1.85E-41 |
| LYL1 | 0.000795023 | 2.479607063 | 7.685840791 | 0.004788589 |
| LINC00608 | 1.87E-19 | 2.455899822 | 45.99675686 | 2.71E-17 |
| HCN1 | 3.20E-08 | 2.450923908 | 18.36778553 | 6.99E-07 |
| AC109454.2 | 0.000296874 | 2.440224279 | 8.607714862 | 0.002092542 |
| ATP10B | 0.000608225 | 2.437701242 | 7.839490391 | 0.00382476 |
| PPIF | 3.34E-07 | 2.435849292 | 15.77361001 | 5.78E-06 |
| AC126763.1 | 0.001803087 | 2.422248997 | 6.646611 | 0.009554752 |
| ATRNL1 | 1.37E-07 | 2.406640006 | 16.51823248 | 2.59E-06 |
| AC023355.2 | 0.000624328 | 2.35589273 | 7.549664379 | 0.003903371 |
| FOLR2 | 2.56E-05 | 2.3530674 | 10.80564904 | 0.000257213 |
| AL355132.1 | 3.05E-11 | 2.334649932 | 24.54903248 | 1.22E-09 |
| SLC25A40 | 8.06E-05 | 2.318278551 | 9.490131695 | 0.000684441 |
| ETF1P2 | 4.27E-05 | 2.316734058 | 10.12305272 | 0.00039738 |
| VNN2 | 2.53E-19 | 2.314797487 | 43.04826733 | 3.56E-17 |
| TTYH1 | 2.31E-06 | 2.309945011 | 13.01825248 | 3.16E-05 |
| GALNT15 | 1.28E-09 | 2.297029875 | 20.42805601 | 3.75E-08 |
| SIGLEC14 | 1.45E-14 | 2.294132583 | 31.74884516 | 1.05E-12 |
| OLAH | 1.34E-05 | 2.291196449 | 11.16157875 | 0.000147169 |
| PFAS | 1.17E-06 | 2.286159245 | 13.5597854 | 1.75E-05 |
| AC004974.1 | 1.80E-07 | 2.283825537 | 15.40320326 | 3.30E-06 |
| GRM1 | 0.001802885 | 2.283559605 | 6.266160599 | 0.009554752 |
| AL138921.2 | 2.68E-18 | 2.274442234 | 39.9668849 | 3.40E-16 |
| AC016598.1 | 0.000313605 | 2.269137433 | 7.950187687 | 0.002186219 |
| LAPTM5 | 2.14E-21 | 2.265093022 | 46.81841922 | 4.25E-19 |
| MAPKAPK3 | 0.000158252 | 2.257774793 | 8.581016647 | 0.001214404 |
| LY6G5B | 2.04E-06 | 2.241753242 | 12.75838321 | 2.83E-05 |
| PSMD10P2 | 0.000133572 | 2.237467268 | 8.668585918 | 0.001048599 |
| MILR1 | 1.74E-12 | 2.236456928 | 26.30208973 | 8.71E-11 |
| AL162586.2 | 1.17E-05 | 2.231887605 | 11.00466739 | 0.000131005 |
| CFP | 1.40E-09 | 2.187257982 | 19.36372445 | 4.06E-08 |
| OR8D4 | 0.000399421 | 2.186231152 | 7.430056647 | 0.002688565 |
| AL161670.2 | 0.000138238 | 2.168922249 | 8.370679122 | 0.001076515 |
| TMEM143 | 0.000247344 | 2.163936213 | 7.804663961 | 0.001787685 |
| ANK1 | 7.61E-09 | 2.159918872 | 17.53547613 | 1.90E-07 |
| AC083967.1 | 0.001256144 | 2.159368901 | 6.264244297 | 0.007054083 |
| ZNF257 | 8.83E-08 | 2.157722558 | 15.22013728 | 1.74E-06 |
| AC005523.2 | 1.13E-06 | 2.157582669 | 12.82772627 | 1.71E-05 |
| AC104333.4 | 6.01E-08 | 2.157532711 | 15.57941122 | 1.24E-06 |
| AC092353.1 | 7.02E-20 | 2.149129741 | 41.16391019 | 1.08E-17 |
| PKHD1L1 | 2.80E-07 | 2.141459898 | 14.03111135 | 4.97E-06 |
| LINC01291 | 6.19E-08 | 2.129113225 | 15.34698438 | 1.27E-06 |
| CASP16P | 5.80E-08 | 2.128960288 | 15.40610958 | 1.20E-06 |

| SLITRK4 | 0.00029477 | 2.116371725 | 7.471885668 | 0.002080099 |
| --- | --- | --- | --- | --- |
| CT75 | 2.82E-19 | 2.111066324 | 39.16098574 | 3.90E-17 |
| AC010525.1 | 6.93E-21 | 2.109697551 | 42.53043929 | 1.26E-18 |
| LINC02340 | 4.96E-09 | 2.103329033 | 17.46701262 | 1.28E-07 |
| EEF1A1P10 | 8.41E-05 | 2.102084461 | 8.565974743 | 0.000708872 |
| AC010632.2 | 1.49E-06 | 2.101084365 | 12.24366762 | 2.15E-05 |
| NUDT18 | 0.000739006 | 2.088646294 | 6.540286847 | 0.004504196 |
| KIR3DX1 | 9.51E-12 | 2.084046981 | 22.97046442 | 4.19E-10 |
| LINC01844 | 0.001500182 | 2.067306365 | 5.837775661 | 0.008165687 |
| LINC00173 | 1.67E-05 | 2.065066954 | 9.866180677 | 0.000177138 |
| LILRA5 | 1.87E-16 | 2.054963143 | 32.32243487 | 1.84E-14 |
| SLC47A1 | 0.001036122 | 2.052211468 | 6.125008024 | 0.005982622 |
| ASIC2 | 1.75E-06 | 2.050982717 | 11.80613239 | 2.49E-05 |
| AC007389.3 | 3.18E-06 | 2.048095693 | 11.26028314 | 4.15E-05 |
| LINC00488 | 0.001459119 | 2.039510247 | 5.783865822 | 0.007989383 |
| TLR1 | 1.33E-05 | 2.029691391 | 9.899028468 | 0.000145629 |
| AOAH | 2.48E-06 | 2.028841041 | 11.37139146 | 3.35E-05 |
| LAMA1 | 6.09E-06 | 2.018369122 | 10.52686519 | 7.38E-05 |
| AMDHD2 | 1.87E-06 | 2.014484666 | 11.54004389 | 2.63E-05 |
| MEI1 | 9.69E-05 | 2.011079289 | 8.071954245 | 0.000798707 |
| MLANA | 3.94E-05 | 2.007612686 | 8.843329223 | 0.000371688 |
| TSHR | 0.00025676 | 2.003473239 | 7.193416446 | 0.001842929 |
| AL121989.1 | 4.32E-08 | 2.002809323 | 14.74980476 | 9.27E-07 |
| AC022154.1 | 1.18E-09 | 1.998077583 | 17.83871631 | 3.49E-08 |
| AC008250.1 | 0.000228248 | 1.997477503 | 7.274000007 | 0.001667514 |
| SLC2A6 | 2.67E-14 | 1.995949461 | 27.09165882 | 1.82E-12 |
| AP5B1 | 1.19E-10 | 1.994992079 | 19.79862072 | 4.20E-09 |
| MTND6P3 | 5.03E-31 | 1.989071861 | 60.26654358 | 3.19E-28 |
| ALOX5AP | 3.57E-20 | 1.986898287 | 38.63921242 | 5.58E-18 |
| PXYLP1 | 4.67E-18 | 1.985784919 | 34.41466107 | 5.74E-16 |
| SLC1A3 | 2.28E-13 | 1.984732286 | 25.08945908 | 1.33E-11 |
| AC132825.2 | 1.03E-18 | 1.983629809 | 35.67873479 | 1.40E-16 |
| CD163L1 | 2.11E-12 | 1.980974694 | 23.12930426 | 1.04E-10 |
| AC011498.4 | 3.15E-07 | 1.980327372 | 12.87667734 | 5.47E-06 |
| AL121757.2 | 5.52E-08 | 1.978837495 | 14.36194308 | 1.15E-06 |
| FTH1P12 | 4.10E-05 | 1.976389314 | 8.670280154 | 0.000384367 |
| CACNA1E | 1.31E-07 | 1.973421896 | 13.58219519 | 2.50E-06 |
| AC027796.1 | 4.54E-06 | 1.972442787 | 10.53923233 | 5.69E-05 |
| LCMT2 | 0.000101753 | 1.970649269 | 7.867720488 | 0.000830663 |
| AC131649.1 | 1.22E-09 | 1.970189762 | 17.559827 | 3.60E-08 |
| ADA | 0.000117361 | 1.969753125 | 7.742069678 | 0.000936903 |
| RPS2P45 | 3.83E-05 | 1.969028812 | 8.69592594 | 0.000363752 |
| PRELID3A | 0.001778683 | 1.965795662 | 5.405744135 | 0.00945535 |
| IFI35 | 2.53E-05 | 1.964592321 | 9.030583064 | 0.000254851 |
| AL121929.3 | 0.001656887 | 1.961805983 | 5.455207969 | 0.008876265 |
| RPS26P21 | 1.86E-08 | 1.961047518 | 15.1615642 | 4.28E-07 |
| RIPOR3 | 2.93E-14 | 1.96044498 | 26.52981495 | 1.99E-12 |
| MT-TQ | 1.15E-17 | 1.958919941 | 33.17967457 | 1.36E-15 |
| PTK2B | 7.39E-15 | 1.953883535 | 27.6106573 | 5.51E-13 |
| KLHL33 | 0.001555239 | 1.945307343 | 5.4628176 | 0.008408239 |
| AC073335.2 | 4.14E-05 | 1.943020855 | 8.516831235 | 0.000386747 |
| SLCO4A1 | 3.01E-06 | 1.927736885 | 10.64446612 | 3.96E-05 |

| AC093835.1 | 2.22E-08 | 1.921586058 | 14.70826402 | 5.02E-07 |
| --- | --- | --- | --- | --- |
| ZBTB16 | 4.11E-28 | 1.921076785 | 52.61118836 | 1.99E-25 |
| SIM2 | 5.06E-06 | 1.920256395 | 10.16903662 | 6.28E-05 |
| AC005041.4 | 1.66E-11 | 1.913655567 | 20.62873952 | 7.07E-10 |
| WWTR1-IT1 | 0.000570813 | 1.906678389 | 6.184323351 | 0.003640438 |
| AF228727.1 | 3.11E-07 | 1.893016019 | 12.3184245 | 5.41E-06 |
| POU2F2 | 7.48E-12 | 1.886744355 | 20.99260476 | 3.40E-10 |
| SLC38A7 | 2.16E-11 | 1.884672021 | 20.10185739 | 8.91E-10 |
| PTH1R | 2.46E-13 | 1.879373781 | 23.69744842 | 1.42E-11 |
| HELB | 2.33E-07 | 1.877150628 | 12.44904556 | 4.18E-06 |
| ADCY1 | 4.16E-07 | 1.87590717 | 11.96950405 | 7.04E-06 |
| KCNH5 | 4.26E-26 | 1.87462818 | 47.56039336 | 1.60E-23 |
| LINC02241 | 7.12E-09 | 1.870208105 | 15.23717305 | 1.79E-07 |
| MBOAT7 | 2.17E-14 | 1.867257112 | 25.51219553 | 1.51E-12 |
| AC009716.2 | 4.71E-08 | 1.865953354 | 13.67110205 | 9.98E-07 |
| ANO2 | 6.04E-09 | 1.860882457 | 15.29512168 | 1.54E-07 |
| AC099521.3 | 1.34E-05 | 1.860742556 | 9.068901426 | 0.000146566 |
| SDHD | 1.48E-07 | 1.858558301 | 12.69516695 | 2.78E-06 |
| HRH2 | 9.31E-10 | 1.854343513 | 16.74680024 | 2.82E-08 |
| AC010623.1 | 2.63E-08 | 1.853996415 | 14.05312644 | 5.84E-07 |
| BIRC5 | 3.58E-06 | 1.847367238 | 10.06098039 | 4.60E-05 |
| FDX2 | 8.14E-16 | 1.841593504 | 27.78805768 | 7.28E-14 |
| EAF1-AS1 | 3.56E-06 | 1.841384048 | 10.03309422 | 4.58E-05 |
| NOSIP | 2.12E-09 | 1.839296435 | 15.95421399 | 5.88E-08 |
| AC215522.2 | 3.85E-33 | 1.839133733 | 59.61436868 | 3.73E-30 |
| AC018521.7 | 0.000659645 | 1.833934605 | 5.833177165 | 0.004083991 |
| LINC02327 | 0.000247414 | 1.829616742 | 6.5986522 | 0.001787685 |
| AC079416.3 | 1.58E-05 | 1.827420418 | 8.775217696 | 0.000168593 |
| Z82215.1 | 1.13E-10 | 1.824005321 | 18.13989971 | 4.02E-09 |
| AC097532.2 | 8.29E-10 | 1.81596569 | 16.49198577 | 2.53E-08 |
| AC020913.2 | 1.31E-09 | 1.810783136 | 16.08668726 | 3.82E-08 |
| TRPM2 | 1.80E-06 | 1.807813357 | 10.38549065 | 2.54E-05 |
| MIR646HG | 2.27E-09 | 1.807573406 | 15.62555565 | 6.24E-08 |
| LINC01579 | 4.57E-05 | 1.807158972 | 7.843824459 | 0.000420268 |
| SLC11A1 | 1.20E-20 | 1.804055148 | 35.93736572 | 2.13E-18 |
| AC092723.1 | 2.20E-06 | 1.803467905 | 10.20442662 | 3.02E-05 |
| C1orf167-AS1 | 0.000187701 | 1.800492351 | 6.709594141 | 0.001407568 |
| PLAGL2 | 0.000572081 | 1.799182573 | 5.833925382 | 0.003646005 |
| CCR4 | 1.66E-09 | 1.799071122 | 15.79799223 | 4.71E-08 |
| TMEM132A | 3.41E-14 | 1.794733651 | 24.17064369 | 2.29E-12 |
| SLC22A9 | 3.97E-18 | 1.793692933 | 31.21272933 | 4.91E-16 |
| ZFYVE26 | 1.55E-06 | 1.789435337 | 10.39813766 | 2.22E-05 |
| RAB19 | 1.42E-06 | 1.780438278 | 10.41418079 | 2.06E-05 |
| LINC01426 | 6.09E-05 | 1.77643822 | 7.48862649 | 0.000538246 |
| LINC00668 | 7.84E-09 | 1.767837968 | 14.32957565 | 1.95E-07 |
| AC140912.1 | 0.001427057 | 1.7673751 | 5.02916962 | 0.007848476 |
| MARCHF3 | 1.64E-05 | 1.764811484 | 8.444685817 | 0.000174713 |
| MPP1 | 2.03E-08 | 1.763453919 | 13.56668664 | 4.64E-07 |
| ANKRD13B | 2.38E-06 | 1.758523701 | 9.889364053 | 3.24E-05 |
| AL161772.1 | 0.001774354 | 1.758043079 | 4.836305747 | 0.009435057 |
| RN7SL774P | 2.06E-05 | 1.755710409 | 8.228634971 | 0.000212556 |
| PTAFR | 1.14E-15 | 1.754919172 | 26.22103447 | 9.99E-14 |

| PCBP3 | 6.78E-14 | 1.753373828 | 23.090014 | 4.38E-12 |
| --- | --- | --- | --- | --- |
| AC018695.2 | 0.001246539 | 1.748430612 | 5.077956765 | 0.007008691 |
| AC008429.1 | 0.000103587 | 1.746262857 | 6.958327318 | 0.000843014 |
| AL356362.1 | 7.95E-07 | 1.743342906 | 10.63408792 | 1.24E-05 |
| PNPLA3 | 0.000980593 | 1.739468729 | 5.233211428 | 0.005719348 |
| SULT1B1 | 0.001486066 | 1.736315103 | 4.910233157 | 0.008108013 |
| AC015910.1 | 1.02E-10 | 1.731001337 | 17.29599098 | 3.65E-09 |
| ABTB1 | 5.14E-12 | 1.7232989 | 19.45373161 | 2.39E-10 |
| MTMR9LP | 0.0012865 | 1.722149006 | 4.978027183 | 0.007200413 |
| GALNT10 | 1.64E-07 | 1.721144828 | 11.67914216 | 3.05E-06 |
| GAB3 | 3.91E-11 | 1.714490875 | 17.84334603 | 1.52E-09 |
| NOCT | 0.000167615 | 1.709791286 | 6.455637903 | 0.001273527 |
| ZNF563 | 5.24E-05 | 1.709614838 | 7.318787756 | 0.00047342 |
| AC124290.1 | 3.19E-05 | 1.708122772 | 7.680197207 | 0.000309133 |
| NEUROD2 | 9.48E-10 | 1.703172668 | 15.36797404 | 2.87E-08 |
| AC002463.1 | 0.000613392 | 1.701859395 | 5.466818468 | 0.003849367 |
| AC021752.1 | 0.001843051 | 1.699888387 | 4.648281206 | 0.009705121 |
| SH3BP2 | 2.40E-14 | 1.697207727 | 23.1151037 | 1.66E-12 |
| DYSF | 2.46E-10 | 1.694598765 | 16.28459285 | 8.26E-09 |
| DISP1 | 9.22E-05 | 1.694449122 | 6.837467044 | 0.000766695 |
| AC139792.1 | 3.53E-07 | 1.687803561 | 10.89070728 | 6.05E-06 |
| TMEM39B | 7.44E-05 | 1.686201017 | 6.960963316 | 0.000638528 |
| AC126564.1 | 0.000973652 | 1.685369959 | 5.075654041 | 0.005682462 |
| CLCN7 | 4.32E-10 | 1.684122369 | 15.77026733 | 1.41E-08 |
| SLA | 1.49E-26 | 1.677337762 | 43.31880073 | 6.11E-24 |
| AC087501.2 | 1.72E-08 | 1.676940952 | 13.02146266 | 3.98E-07 |
| AL355075.4 | 4.80E-05 | 1.673671723 | 7.228285901 | 0.000439065 |
| ZFP64 | 2.46E-06 | 1.669046715 | 9.36135007 | 3.33E-05 |
| FABP3 | 4.05E-05 | 1.666507012 | 7.320052564 | 0.000380414 |
| AC130448.1 | 7.37E-08 | 1.663792672 | 11.86736642 | 1.49E-06 |
| LFNG | 9.43E-07 | 1.663206891 | 10.02136124 | 1.45E-05 |
| AC016027.2 | 0.000770767 | 1.660239304 | 5.16845294 | 0.00467458 |
| NCF1 | 4.79E-65 | 1.657513788 | 106.6106236 | 2.21E-61 |
| AL592295.5 | 8.07E-07 | 1.657107846 | 10.09712938 | 1.26E-05 |
| RIPOR2 | 5.42E-12 | 1.654070287 | 18.63436083 | 2.51E-10 |
| MTERF1 | 2.42E-06 | 1.647877662 | 9.255936548 | 3.27E-05 |
| AC006967.3 | 4.57E-07 | 1.642843788 | 10.41574922 | 7.59E-06 |
| NANOG | 0.001582797 | 1.639687109 | 4.592066569 | 0.008537186 |
| ZNF578 | 1.31E-05 | 1.638026465 | 8.000214858 | 0.000143918 |
| FTH1P11 | 5.96E-21 | 1.63444898 | 33.05669297 | 1.10E-18 |
| AL355472.1 | 2.30E-09 | 1.632553852 | 14.10213253 | 6.32E-08 |
| AL160412.1 | 5.78E-06 | 1.632200043 | 8.549884441 | 7.05E-05 |
| SLC15A3 | 2.18E-14 | 1.629024393 | 22.25535761 | 1.51E-12 |
| AC103740.1 | 1.26E-05 | 1.626241135 | 7.968873597 | 0.000138971 |
| NLRC4 | 2.33E-09 | 1.626049273 | 14.03807241 | 6.38E-08 |
| SNRNP40 | 1.22E-05 | 1.624667447 | 7.980848561 | 0.000135669 |
| ADAMTSL4-AS1 | 3.50E-05 | 1.619419355 | 7.216280967 | 0.000335749 |
| AC007342.1 | 8.61E-11 | 1.614265913 | 16.24742559 | 3.13E-09 |
| AC104041.1 | 3.69E-08 | 1.613033701 | 11.99058029 | 7.97E-07 |
| NCF1C | 1.89E-72 | 1.611942586 | 115.6158082 | 1.16E-68 |
| AC007382.1 | 0.000109114 | 1.609596339 | 6.377414331 | 0.000880605 |
| RGS16 | 5.02E-15 | 1.608445038 | 22.99983569 | 3.87E-13 |

| AC078962.2 | 8.56E-08 | 1.608027109 | 11.36438125 | 1.70E-06 |
| --- | --- | --- | --- | --- |
| TRHDE-AS1 | 5.73E-08 | 1.605200991 | 11.62418861 | 1.19E-06 |
| KY | 1.06E-09 | 1.605026883 | 14.40470332 | 3.16E-08 |
| SH3PXD2B | 0.00029739 | 1.603084236 | 5.653554626 | 0.002094577 |
| LIPA | 7.07E-13 | 1.60266865 | 19.47343791 | 3.81E-11 |
| SH3BGRL3 | 1.62E-09 | 1.602250928 | 14.08379222 | 4.63E-08 |
| AL606970.3 | 5.42E-05 | 1.602107384 | 6.835146874 | 0.000486988 |
| STAT5A | 1.98E-12 | 1.597794229 | 18.70011523 | 9.85E-11 |
| OR1L8 | 0.001410387 | 1.595691169 | 4.548775656 | 0.007775698 |
| CHKB-DT | 7.92E-08 | 1.594608293 | 11.3239295 | 1.58E-06 |
| AC106791.3 | 9.33E-05 | 1.592909915 | 6.419486255 | 0.000774144 |
| RN7SKP264 | 0.000294625 | 1.590951442 | 5.617221096 | 0.00207987 |
| RPL23AP19 | 1.16E-08 | 1.588196389 | 12.6040948 | 2.79E-07 |
| C10orf143 | 1.26E-06 | 1.585369152 | 9.353194218 | 1.87E-05 |
| ANKRD30BL | 9.20E-14 | 1.582789656 | 20.63341289 | 5.75E-12 |
| AC005280.3 | 8.46E-06 | 1.57965306 | 8.012904553 | 9.84E-05 |
| GSTZ1 | 0.000162015 | 1.577636868 | 5.979947502 | 0.001240182 |
| CYBA | 4.48E-11 | 1.573550129 | 16.28399101 | 1.72E-09 |
| AC005899.7 | 2.82E-11 | 1.573366746 | 16.59848873 | 1.13E-09 |
| UPB1 | 2.01E-07 | 1.568582187 | 10.50412347 | 3.64E-06 |
| HJURP | 0.001318804 | 1.568473081 | 4.516919851 | 0.007352182 |
| ZNF583 | 9.56E-05 | 1.561869227 | 6.278268217 | 0.000788858 |
| AL031600.3 | 3.12E-11 | 1.561202466 | 16.4028353 | 1.23E-09 |
| HPR | 5.99E-13 | 1.560235891 | 19.07050319 | 3.24E-11 |
| AFMID | 2.91E-09 | 1.558641491 | 13.30368846 | 7.92E-08 |
| POLM | 1.21E-05 | 1.550474882 | 7.622284283 | 0.000134649 |
| AL354707.1 | 0.000266635 | 1.548664323 | 5.535055136 | 0.001905642 |
| AC005899.9 | 8.13E-05 | 1.548632082 | 6.333847535 | 0.000688629 |
| AC068205.2 | 0.000407813 | 1.547631745 | 5.245758509 | 0.00273036 |
| AL031595.2 | 0.001660399 | 1.547236627 | 4.300989163 | 0.008890685 |
| RBL1 | 0.00080721 | 1.547143132 | 4.785334506 | 0.004850895 |
| FKBP5 | 3.29E-21 | 1.544212032 | 31.62928977 | 6.25E-19 |
| AC091825.3 | 4.36E-11 | 1.54298794 | 15.98678015 | 1.67E-09 |
| RNF112 | 0.000348228 | 1.540065399 | 5.325755992 | 0.002391391 |
| LINC01301 | 4.57E-06 | 1.539857973 | 8.222677816 | 5.73E-05 |
| TIGD5 | 6.50E-08 | 1.53824119 | 11.05501785 | 1.33E-06 |
| AC069257.4 | 0.000145034 | 1.536790533 | 5.89901498 | 0.00112327 |
| TRG-AS1 | 8.66E-05 | 1.53481158 | 6.235164052 | 0.000726075 |
| LINC01220 | 2.22E-05 | 1.531715984 | 7.12702719 | 0.00022747 |
| AC008079.1 | 2.44E-10 | 1.525428208 | 14.66291711 | 8.22E-09 |
| BIN2 | 1.26E-17 | 1.524194101 | 25.75931024 | 1.48E-15 |
| ANPEP | 4.10E-06 | 1.523463585 | 8.206665221 | 5.22E-05 |
| AL591379.1 | 1.05E-05 | 1.52331397 | 7.586780059 | 0.000118478 |
| FCGR2A | 2.75E-15 | 1.522765427 | 22.1718753 | 2.22E-13 |
| TFEC | 1.01E-12 | 1.522586294 | 18.26315914 | 5.34E-11 |
| RHBDF2 | 0.000257481 | 1.521204996 | 5.459991637 | 0.001847388 |
| RBM22P2 | 0.001104891 | 1.516810198 | 4.48472349 | 0.00631048 |
| UBXN11 | 5.61E-09 | 1.515589831 | 12.50571929 | 1.44E-07 |
| GRHPR | 9.32E-12 | 1.514393006 | 16.70438023 | 4.12E-10 |
| DUSP19 | 2.01E-06 | 1.513067062 | 8.619042778 | 2.80E-05 |
| IGSF6 | 5.11E-17 | 1.511186907 | 24.6199968 | 5.41E-15 |
| GMFG | 2.00E-05 | 1.511086237 | 7.101104484 | 0.000207313 |

| PCOLCE2 | 0.000261097 | 1.506209844 | 5.397047878 | 0.001868373 |
| --- | --- | --- | --- | --- |
| KCNH6 | 0.00184605 | 1.505708587 | 4.116240726 | 0.009718134 |
| PLIN2 | 2.07E-17 | 1.504876719 | 25.1066712 | 2.36E-15 |
| RPS14P5 | 2.86E-11 | 1.503700864 | 15.85432117 | 1.14E-09 |
| MT-TN | 3.22E-24 | 1.498603167 | 35.20440542 | 8.74E-22 |
| C1orf174 | 7.38E-05 | 1.496887658 | 6.185083126 | 0.000634192 |
| OR1I1 | 0.000113298 | 1.495401303 | 5.900523935 | 0.000909986 |
| AL158825.2 | 4.17E-07 | 1.492434126 | 9.522097888 | 7.04E-06 |
| BEAN1 | 0.001044513 | 1.491410047 | 4.446021938 | 0.006023521 |
| ST18 | 9.10E-05 | 1.485239623 | 6.001767604 | 0.000758705 |
| AC099506.1 | 5.51E-10 | 1.48385527 | 13.73932296 | 1.75E-08 |
| AC120114.3 | 2.91E-06 | 1.483598089 | 8.213557181 | 3.85E-05 |
| PRSS40A | 0.001086208 | 1.482231436 | 4.393462758 | 0.006223068 |
| AC004908.1 | 0.000108279 | 1.481638038 | 5.87537245 | 0.000876065 |
| PTPRB | 8.36E-16 | 1.479296624 | 22.3044493 | 7.44E-14 |
| SLC4A1 | 0.000366638 | 1.477602754 | 5.07669278 | 0.002500514 |
| TCIRG1 | 2.49E-13 | 1.476001568 | 18.60250717 | 1.44E-11 |
| RNF157 | 0.000667758 | 1.474289502 | 4.681430842 | 0.004128671 |
| PCSK2 | 0.00051651 | 1.473230239 | 4.842391851 | 0.003353311 |
| STOML1 | 0.001548873 | 1.470439984 | 4.131912923 | 0.008383664 |
| AC012636.1 | 0.000149545 | 1.469379135 | 5.620712386 | 0.001153352 |
| BISPR | 6.78E-05 | 1.468689281 | 6.123040186 | 0.00058804 |
| ZNF311 | 0.00083405 | 1.468667755 | 4.521745782 | 0.004982936 |
| MAP3K5-AS1 | 0.001847425 | 1.46593819 | 4.007044137 | 0.009722594 |
| P2RX4 | 2.89E-13 | 1.463797815 | 18.35544533 | 1.65E-11 |
| PPP1R14D | 0.001525833 | 1.459143325 | 4.109666876 | 0.008271856 |
| LAIR1 | 2.00E-15 | 1.458966182 | 21.4441032 | 1.66E-13 |
| ABCC8 | 2.60E-05 | 1.458591436 | 6.687734894 | 0.000260898 |
| AL357552.3 | 3.42E-11 | 1.453993625 | 15.21798226 | 1.34E-09 |
| ZNF180 | 9.80E-07 | 1.453200581 | 8.731936245 | 1.50E-05 |
| AP005900.2 | 4.77E-05 | 1.452925878 | 6.279326765 | 0.00043688 |
| GTF3C2-AS1 | 4.91E-05 | 1.450472254 | 6.250159916 | 0.000447718 |
| TYROBP | 1.25E-10 | 1.448956236 | 14.34704534 | 4.40E-09 |
| FAAP24 | 0.001511774 | 1.44815111 | 4.084529312 | 0.008209382 |
| OR10A3 | 0.000457817 | 1.447487266 | 4.833605614 | 0.003012601 |
| TNFSF14 | 8.72E-11 | 1.447050684 | 14.55671364 | 3.16E-09 |
| SLC36A1 | 0.00018168 | 1.446928961 | 5.412516676 | 0.001367983 |
| RPS14P4 | 6.53E-07 | 1.44671687 | 8.947769982 | 1.04E-05 |
| AL022724.3 | 2.00E-06 | 1.446126358 | 8.240615708 | 2.79E-05 |
| FAM71F2 | 1.25E-06 | 1.446123081 | 8.5361867 | 1.86E-05 |
| MCOLN1 | 0.000873264 | 1.443764721 | 4.416266372 | 0.005183596 |
| TCAF1P1 | 0.000165999 | 1.44333483 | 5.45565169 | 0.001264905 |
| CKLF | 7.77E-08 | 1.442471014 | 10.25557579 | 1.56E-06 |
| TYRO3 | 1.60E-05 | 1.441674477 | 6.915401372 | 0.000170346 |
| MCOLN3 | 0.000912466 | 1.441384527 | 4.381496945 | 0.005383342 |
| AC005776.2 | 2.45E-12 | 1.43995749 | 16.72025687 | 1.20E-10 |
| FBXO48 | 0.000645212 | 1.438809762 | 4.590231282 | 0.004010807 |
| TFCP2L1 | 4.26E-06 | 1.437820893 | 7.721239333 | 5.39E-05 |
| TNFRSF13C | 1.35E-06 | 1.437072576 | 8.433854662 | 1.98E-05 |
| IGFBPL1 | 5.53E-05 | 1.437060894 | 6.118287032 | 0.000495332 |
| AC005005.4 | 3.79E-06 | 1.436906051 | 7.79039488 | 4.84E-05 |
| C21orf91-OT1 | 4.70E-09 | 1.435777875 | 11.95748951 | 1.22E-07 |

| AC087620.1 | 1.46E-05 | 1.43540426 | 6.941766233 | 0.000157886 |
| --- | --- | --- | --- | --- |
| RNU4-62P | 6.57E-05 | 1.435091443 | 6.002358589 | 0.000574629 |
| AC114495.2 | 6.81E-08 | 1.433537138 | 10.27436255 | 1.38E-06 |
| VSIR | 1.25E-10 | 1.433174166 | 14.19102243 | 4.40E-09 |
| BRCA1 | 2.71E-11 | 1.433019423 | 15.14206744 | 1.09E-09 |
| H6PD | 1.20E-29 | 1.432057992 | 41.41456771 | 6.52E-27 |
| NPFFR1 | 2.02E-06 | 1.428632026 | 8.134471484 | 2.82E-05 |
| FFAR4 | 0.001340764 | 1.428444048 | 4.103416558 | 0.007447565 |
| AC118755.1 | 4.15E-06 | 1.427614017 | 7.682958631 | 5.27E-05 |
| NINJ1 | 9.17E-07 | 1.424890005 | 8.603056832 | 1.42E-05 |
| UPP1 | 1.91E-08 | 1.422773628 | 10.9817932 | 4.39E-07 |
| U52112.1 | 0.000586188 | 1.421842053 | 4.595340713 | 0.003720427 |
| TPP1 | 8.11E-14 | 1.421822935 | 18.61334704 | 5.11E-12 |
| AL031658.1 | 7.49E-10 | 1.42110253 | 12.968083 | 2.31E-08 |
| CEMIP | 0.00022608 | 1.419824559 | 5.176307824 | 0.001653643 |
| AL139099.4 | 1.44E-07 | 1.413774139 | 9.671916624 | 2.72E-06 |
| LINC02811 | 0.000406445 | 1.412002306 | 4.788096553 | 0.002723185 |
| CA12 | 1.18E-08 | 1.411194354 | 11.18854861 | 2.82E-07 |
| SFMBT2 | 1.80E-09 | 1.409386118 | 12.32532163 | 5.05E-08 |
| C2CD3 | 6.65E-28 | 1.406703746 | 38.23052589 | 3.14E-25 |
| SNORA75 | 0.000585888 | 1.406601829 | 4.546398205 | 0.003720427 |
| LARGE-AS1 | 0.001505014 | 1.403866828 | 3.962357037 | 0.008185185 |
| CC2D1B | 8.22E-06 | 1.403577663 | 7.13738378 | 9.57E-05 |
| OAS3 | 2.11E-06 | 1.402089724 | 7.958310241 | 2.91E-05 |
| AC100849.2 | 0.000292255 | 1.400773374 | 4.950666239 | 0.002066309 |
| LINC01482 | 1.16E-06 | 1.396437153 | 8.29097504 | 1.73E-05 |
| FCGR1CP | 1.00E-07 | 1.394729209 | 9.760864844 | 1.96E-06 |
| AC009220.2 | 1.00E-05 | 1.394724933 | 6.973391676 | 0.000114201 |
| ADORA2A | 4.29E-13 | 1.393942024 | 17.23993023 | 2.37E-11 |
| AL031963.2 | 0.000133776 | 1.393710237 | 5.398706578 | 0.001049754 |
| DENND4C | 6.33E-08 | 1.39337936 | 10.03045053 | 1.30E-06 |
| AC027307.3 | 5.72E-05 | 1.391521031 | 5.903672492 | 0.000509874 |
| SLC36A2 | 0.001175938 | 1.389535156 | 4.070804087 | 0.006658466 |
| AP002807.1 | 8.87E-13 | 1.388790209 | 16.73772617 | 4.71E-11 |
| VASH1 | 1.79E-06 | 1.388248943 | 7.978759353 | 2.53E-05 |
| FCGR1A | 2.16E-08 | 1.388038993 | 10.63918281 | 4.92E-07 |
| CCNB3P1 | 1.77E-06 | 1.38606622 | 7.973593787 | 2.50E-05 |
| AC087721.1 | 3.64E-05 | 1.385818832 | 6.151725542 | 0.000347193 |
| AP000350.7 | 0.000801286 | 1.382641578 | 4.280952191 | 0.004821584 |
| TMEM44 | 9.12E-09 | 1.382621253 | 11.1161764 | 2.24E-07 |
| GRINA | 3.36E-31 | 1.380570595 | 42.07079713 | 2.21E-28 |
| AL450998.2 | 1.17E-09 | 1.377389631 | 12.30132381 | 3.47E-08 |
| KIF14 | 2.44E-05 | 1.376159812 | 6.348056802 | 0.000247136 |
| FGR | 1.38E-20 | 1.375306421 | 27.3143849 | 2.40E-18 |
| AL358394.2 | 0.000209898 | 1.373607295 | 5.052117609 | 0.001551288 |
| AC106795.2 | 0.000988992 | 1.372624453 | 4.124471658 | 0.005755686 |
| NR5A2 | 8.66E-05 | 1.371854086 | 5.572901517 | 0.000726075 |
| AC084756.1 | 0.000902394 | 1.369747515 | 4.170338211 | 0.005330753 |
| AC010542.6 | 7.95E-07 | 1.36498614 | 8.325884293 | 1.24E-05 |
| AL117329.1 | 3.72E-07 | 1.364947307 | 8.775260397 | 6.35E-06 |
| VIPR1 | 2.10E-08 | 1.363118119 | 10.46491912 | 4.80E-07 |
| AC245060.6 | 0.000702729 | 1.361362186 | 4.292664167 | 0.00432298 |

| ITGA10 | 0.001218827 | 1.361167224 | 3.966520325 | 0.006869648 |
| --- | --- | --- | --- | --- |
| LPCAT2 | 0.000370405 | 1.361000504 | 4.670032624 | 0.002519273 |
| MAFB | 3.96E-18 | 1.358983314 | 23.64945983 | 4.91E-16 |
| AC011396.2 | 0.00085168 | 1.357710362 | 4.167795171 | 0.00507346 |
| AL031864.2 | 0.000220837 | 1.356859959 | 4.960582229 | 0.001622378 |
| CLEC18A | 0.000402364 | 1.354966519 | 4.600627462 | 0.002702718 |
| MT-TL2 | 0.000152873 | 1.353321201 | 5.163824985 | 0.001176562 |
| B3GNT6 | 2.59E-06 | 1.352140594 | 7.553533306 | 3.47E-05 |
| FCGR1B | 4.90E-08 | 1.3518616 | 9.881506967 | 1.03E-06 |
| ZNF438 | 3.94E-13 | 1.351841594 | 16.76828944 | 2.21E-11 |
| SKP2 | 0.000661169 | 1.351805589 | 4.298319431 | 0.004091687 |
| AC007040.2 | 6.23E-05 | 1.350130099 | 5.677989179 | 0.000550012 |
| AC011498.7 | 2.43E-13 | 1.349716335 | 17.02686811 | 1.41E-11 |
| AC243829.4 | 1.31E-77 | 1.347390799 | 103.5909548 | 2.41E-73 |
| TDRKH-AS1 | 0.000889143 | 1.346944656 | 4.109566695 | 0.005259214 |
| RSKR | 1.37E-11 | 1.345421587 | 14.61531403 | 5.90E-10 |
| AC003102.1 | 7.90E-06 | 1.34421712 | 6.858833422 | 9.26E-05 |
| AC025569.1 | 6.53E-06 | 1.34401562 | 6.969255805 | 7.85E-05 |
| AC104532.2 | 5.93E-07 | 1.342965224 | 8.36217439 | 9.56E-06 |
| GREB1L | 0.000184013 | 1.341888571 | 5.012156979 | 0.001383289 |
| HP | 1.81E-11 | 1.340908708 | 14.40307269 | 7.65E-10 |
| ATP6V0C | 1.59E-16 | 1.340660292 | 21.18173075 | 1.60E-14 |
| AC093525.2 | 1.62E-16 | 1.340660292 | 21.1698259 | 1.62E-14 |
| KCNG4 | 1.12E-05 | 1.337973366 | 6.621817498 | 0.000125999 |
| C2 | 5.85E-08 | 1.335605268 | 9.659766874 | 1.21E-06 |
| LINC00907 | 0.000323375 | 1.335248473 | 4.660409538 | 0.002244981 |
| AC016876.2 | 9.78E-16 | 1.334032993 | 20.02331824 | 8.62E-14 |
| SCN1A-AS1 | 0.000678951 | 1.33354046 | 4.224871512 | 0.00419225 |
| ZNF135 | 9.47E-05 | 1.332482847 | 5.361173772 | 0.000783512 |
| ANKRD49P2 | 3.07E-06 | 1.332126338 | 7.343952567 | 4.03E-05 |
| MHENCR | 0.001530851 | 1.331671675 | 3.748745252 | 0.008295859 |
| TSPAN14 | 1.83E-24 | 1.330906929 | 31.59144042 | 5.36E-22 |
| GLDN | 2.44E-18 | 1.330655769 | 23.43635039 | 3.17E-16 |
| CCDC150 | 0.000534707 | 1.327773696 | 4.344321803 | 0.003447157 |
| PTCHD3P2 | 0.000323891 | 1.327177522 | 4.631319731 | 0.002245446 |
| FAM107A | 6.43E-18 | 1.326314897 | 22.80156733 | 7.80E-16 |
| AC018628.2 | 3.26E-06 | 1.324850899 | 7.269355502 | 4.23E-05 |
| MIRLET7BHG | 0.000437294 | 1.324505309 | 4.449313401 | 0.002897644 |
| IL4I1 | 0.000164457 | 1.323186742 | 5.006869187 | 0.001255748 |
| PSMB9 | 3.51E-05 | 1.320941127 | 5.88376806 | 0.000336845 |
| CSTF3-DT | 0.001363899 | 1.318387121 | 3.777466404 | 0.007557844 |
| KCNAB2 | 0.000107425 | 1.317477491 | 5.228930211 | 0.000870137 |
| RPL23AP64 | 0.001635504 | 1.315685299 | 3.665957548 | 0.008777793 |
| AC008708.2 | 0.000101812 | 1.314960624 | 5.249586788 | 0.000830774 |
| CDH18 | 4.89E-06 | 1.312983235 | 6.973336564 | 6.09E-05 |
| AL034550.2 | 0.000148306 | 1.312669984 | 5.026003133 | 0.001145251 |
| AC020908.3 | 0.001780314 | 1.312165491 | 3.607803427 | 0.009461289 |
| AC079915.1 | 1.75E-05 | 1.31130297 | 6.239370618 | 0.000184592 |
| NQO1 | 2.63E-08 | 1.306036901 | 9.900484681 | 5.84E-07 |
| CRYM-AS1 | 5.21E-08 | 1.305470631 | 9.507853248 | 1.09E-06 |
| VHL | 1.17E-08 | 1.302988208 | 10.33676294 | 2.80E-07 |
| AC068580.4 | 0.00040179 | 1.299782651 | 4.414062825 | 0.002700833 |

| MIR194-2HG | 1.31E-06 | 1.299727596 | 7.647188164 | 1.92E-05 |
| --- | --- | --- | --- | --- |
| AC023818.1 | 1.63E-05 | 1.295515054 | 6.202621418 | 0.000173719 |
| KREMEN1 | 0.00133957 | 1.295256285 | 3.721316291 | 0.007447253 |
| IBA57 | 8.52E-06 | 1.294592018 | 6.562960293 | 9.90E-05 |
| CTSD | 8.62E-18 | 1.294301333 | 22.08676493 | 1.04E-15 |
| CSRP3 | 0.000253532 | 1.293768132 | 4.652346886 | 0.001823316 |
| TMEM150B | 0.000441159 | 1.2922998 | 4.33618963 | 0.002919664 |
| FAM157B | 4.81E-32 | 1.290286915 | 40.40960114 | 3.85E-29 |
| C11orf1 | 4.99E-05 | 1.289725902 | 5.54849211 | 0.000453906 |
| TEAD3 | 1.68E-11 | 1.28904639 | 13.88800316 | 7.13E-10 |
| TBC1D17 | 0.000921964 | 1.2854801 | 3.901799902 | 0.00542895 |
| LINC01569 | 0.000102805 | 1.284537434 | 5.12271876 | 0.00083739 |
| GPX1 | 8.20E-06 | 1.282763644 | 6.524256021 | 9.57E-05 |
| SHISAL2A | 2.98E-06 | 1.282029913 | 7.083826538 | 3.93E-05 |
| C5orf66 | 8.11E-05 | 1.280591925 | 5.238588369 | 0.00068769 |
| SGSH | 0.000692445 | 1.279826478 | 4.043758765 | 0.004265562 |
| BAK1 | 9.13E-06 | 1.27967494 | 6.448712815 | 0.000105514 |
| AC010655.2 | 1.67E-06 | 1.279191761 | 7.390738985 | 2.39E-05 |
| AC010655.3 | 1.67E-06 | 1.279191761 | 7.390738985 | 2.39E-05 |
| MOB3A | 2.13E-11 | 1.278747547 | 13.64649009 | 8.84E-10 |
| LINC01934 | 0.000844433 | 1.277677769 | 3.926859313 | 0.005036801 |
| SUSD6 | 1.63E-07 | 1.269852034 | 8.620357567 | 3.04E-06 |
| AC011468.2 | 0.000607094 | 1.26962082 | 4.084045365 | 0.00381895 |
| CLSTN3 | 6.57E-11 | 1.269387829 | 12.92570492 | 2.42E-09 |
| MT-TM | 1.60E-22 | 1.264738044 | 27.56740732 | 3.82E-20 |
| AL513282.1 | 0.000250615 | 1.264573777 | 4.55372163 | 0.001806564 |
| COL9A1 | 2.13E-05 | 1.261115149 | 5.892701114 | 0.000218381 |
| HNRNPA1P54 | 0.000556497 | 1.258709392 | 4.096516916 | 0.003563938 |
| AC090643.1 | 2.17E-06 | 1.25840089 | 7.126569327 | 2.99E-05 |
| SNX22 | 1.33E-08 | 1.257112151 | 9.901498344 | 3.14E-07 |
| YPEL1 | 2.35E-06 | 1.256274341 | 7.070881325 | 3.21E-05 |
| TIGAR | 0.000461782 | 1.25276045 | 4.178661384 | 0.003034356 |
| AC090559.1 | 0.0001878 | 1.252155674 | 4.665914049 | 0.001407734 |
| HAVCR2 | 0.001514897 | 1.251002993 | 3.527349136 | 0.008223918 |
| NUGGC | 1.69E-05 | 1.25000421 | 5.965362652 | 0.000179198 |
| CSK | 1.12E-06 | 1.246724821 | 7.417106892 | 1.70E-05 |
| RN7SL32P | 3.00E-05 | 1.242732181 | 5.620790273 | 0.000294295 |
| ARSB | 0.00146928 | 1.242721871 | 3.520501119 | 0.008033081 |
| AMER2 | 9.39E-05 | 1.242220695 | 5.002896708 | 0.00077759 |
| TCAF2P1 | 1.72E-07 | 1.242122906 | 8.401864845 | 3.17E-06 |
| RPL13AP12 | 0.001367462 | 1.240091239 | 3.551726467 | 0.007573034 |
| AC080097.2 | 2.41E-05 | 1.239084339 | 5.721930364 | 0.00024485 |
| AL162458.1 | 1.56E-05 | 1.238873628 | 5.954294041 | 0.000167338 |
| AC007272.1 | 1.51E-07 | 1.238571196 | 8.448130658 | 2.83E-06 |
| GFOD1 | 1.15E-05 | 1.238177855 | 6.11661808 | 0.000128517 |
| C12orf66 | 0.0016007 | 1.236239743 | 3.456143134 | 0.008616097 |
| PGLS | 0.000540478 | 1.235978293 | 4.038215562 | 0.003475927 |
| AC079174.1 | 0.000147052 | 1.235734447 | 4.735986511 | 0.001137465 |
| RNF125 | 6.22E-09 | 1.235587997 | 10.13959967 | 1.58E-07 |
| JAK3 | 3.69E-12 | 1.234216324 | 14.11043314 | 1.77E-10 |
| GNPTG | 1.31E-05 | 1.233402353 | 6.021160551 | 0.000144598 |
| GTF3C5 | 0.00072323 | 1.227786548 | 3.856138392 | 0.004431495 |

| AL356273.6 | 6.12E-11 | 1.227544016 | 12.53733026 | 2.27E-09 |
| --- | --- | --- | --- | --- |
| HIF3A | 2.65E-19 | 1.227095143 | 22.79524263 | 3.70E-17 |
| CCR1 | 1.66E-25 | 1.223897911 | 30.32904293 | 5.55E-23 |
| MTMR9 | 8.24E-08 | 1.221406181 | 8.652537985 | 1.64E-06 |
| WAS | 3.03E-08 | 1.221216428 | 9.182220322 | 6.65E-07 |
| EBAG9P1 | 0.000284177 | 1.220986558 | 4.330120813 | 0.002016155 |
| LINC01859 | 0.000990023 | 1.219454825 | 3.663674707 | 0.005757948 |
| BCL6 | 7.62E-09 | 1.218134666 | 9.888774604 | 1.90E-07 |
| TNFRSF6B | 5.30E-05 | 1.21589897 | 5.198518302 | 0.000478519 |
| DGAT2 | 6.26E-05 | 1.215680538 | 5.10981269 | 0.000551387 |
| AC138123.2 | 0.000637926 | 1.214587652 | 3.880886174 | 0.003974905 |
| ITGAL | 0.000368253 | 1.214425591 | 4.170159207 | 0.002507417 |
| XYLT2 | 0.000605821 | 1.213022343 | 3.903088352 | 0.003812244 |
| IFITM1 | 1.39E-14 | 1.211866397 | 16.79190955 | 1.01E-12 |
| RASL10B | 3.19E-06 | 1.211259859 | 6.657427803 | 4.16E-05 |
| AL353625.1 | 0.000164903 | 1.209743323 | 4.576184078 | 0.001258107 |
| CFLAR-AS1 | 4.35E-07 | 1.207491223 | 7.681395584 | 7.29E-06 |
| RHD | 6.68E-05 | 1.205460746 | 5.033235952 | 0.000581472 |
| PPM1F-AS1 | 0.000703705 | 1.205366798 | 3.80005068 | 0.004324805 |
| PGD | 3.94E-05 | 1.205155 | 5.308405554 | 0.000371688 |
| TMC6 | 1.13E-09 | 1.205065004 | 10.78121675 | 3.35E-08 |
| ITGB2 | 0.000158341 | 1.201489791 | 4.56615025 | 0.001214584 |
| PRR11 | 2.62E-05 | 1.201345539 | 5.503251631 | 0.000263121 |
| CXorf56 | 5.22E-05 | 1.200606965 | 5.141307622 | 0.000472468 |
| CD68 | 5.68E-16 | 1.199651315 | 18.28924905 | 5.18E-14 |
| AC005037.1 | 2.17E-21 | 1.199531652 | 24.78640439 | 4.25E-19 |
| LINC01224 | 9.61E-05 | 1.197114394 | 4.809123109 | 0.000792969 |
| MT-TD | 2.23E-11 | 1.197098919 | 12.75048662 | 9.16E-10 |
| ZNF844 | 3.32E-10 | 1.196791961 | 11.34441235 | 1.10E-08 |
| AC012100.2 | 9.49E-13 | 1.195321116 | 14.37124463 | 5.02E-11 |
| AC073065.1 | 0.001868594 | 1.193601741 | 3.256724635 | 0.009825581 |
| AL139317.1 | 0.000360009 | 1.19226953 | 4.105802105 | 0.00246407 |
| KCNJ15 | 3.00E-05 | 1.190563829 | 5.384527755 | 0.000294314 |
| CD58 | 9.59E-07 | 1.188676284 | 7.153642023 | 1.47E-05 |
| BHMT2 | 0.000323929 | 1.188369386 | 4.146874303 | 0.002245446 |
| AL513497.1 | 1.78E-07 | 1.187980413 | 8.017545249 | 3.27E-06 |
| AC007998.3 | 2.65E-05 | 1.187455886 | 5.435160766 | 0.000264832 |
| ALPL | 1.22E-19 | 1.186427199 | 22.44035253 | 1.80E-17 |
| AC126474.2 | 0.000115952 | 1.184407273 | 4.661498016 | 0.000927541 |
| TMEM266 | 2.72E-05 | 1.183591272 | 5.404337191 | 0.000270219 |
| ABCG8 | 2.28E-06 | 1.182723787 | 6.67224484 | 3.13E-05 |
| PSTK | 4.13E-05 | 1.18186761 | 5.18182441 | 0.000386122 |
| SLC15A1 | 2.52E-08 | 1.179806004 | 8.96586673 | 5.64E-07 |
| RGPD4-AS1 | 0.000167045 | 1.178435106 | 4.451146989 | 0.001271291 |
| ARID3A | 2.56E-08 | 1.178251976 | 8.944985728 | 5.71E-07 |
| ZFHX2-AS1 | 8.35E-07 | 1.174974324 | 7.141796123 | 1.30E-05 |
| MGAT1 | 4.36E-08 | 1.173553001 | 8.637821119 | 9.34E-07 |
| NR1H3 | 1.89E-05 | 1.173499553 | 5.543419598 | 0.000197949 |
| CDC42-AS1 | 0.00018483 | 1.171265769 | 4.372600308 | 0.001388867 |
| ETFBKMT | 0.001576952 | 1.170734787 | 3.280611501 | 0.008508151 |
| LIN52 | 0.00182429 | 1.170475738 | 3.205823301 | 0.009625583 |
| U2AF1L4 | 1.02E-05 | 1.16088516 | 5.792879028 | 0.000116154 |

| ISY1 | 3.28E-06 | 1.156885762 | 6.344936365 | 4.25E-05 |
| --- | --- | --- | --- | --- |
| SPNS1 | 1.01E-07 | 1.155887853 | 8.085374351 | 1.97E-06 |
| NSUN4 | 1.43E-05 | 1.155340151 | 5.596379748 | 0.000155434 |
| NCKAP1L | 4.27E-13 | 1.155014781 | 14.28711028 | 2.36E-11 |
| AUXG01000058.1 | 4.57E-08 | 1.15443359 | 8.473308797 | 9.71E-07 |
| MT-TE | 9.61E-10 | 1.154210795 | 10.40779467 | 2.89E-08 |
| TTC9C | 5.09E-05 | 1.152770921 | 4.949582877 | 0.000461379 |
| CORO1A | 3.52E-06 | 1.150517791 | 6.27475506 | 4.53E-05 |
| SEC31B | 2.50E-05 | 1.149273547 | 5.288232696 | 0.000252384 |
| PTPRJ | 2.88E-07 | 1.144194248 | 7.484478985 | 5.06E-06 |
| LINC02485 | 4.27E-06 | 1.143791333 | 6.141631088 | 5.39E-05 |
| CLEC18C | 9.79E-06 | 1.14254789 | 5.72347404 | 0.000112131 |
| PTPDC1 | 0.000106508 | 1.141150158 | 4.533354509 | 0.000863355 |
| ZNF366 | 2.17E-08 | 1.140675024 | 8.7413765 | 4.93E-07 |
| LILRB3 | 6.05E-15 | 1.139852894 | 16.20653566 | 4.61E-13 |
| FTH1P2 | 9.41E-14 | 1.13984146 | 14.84778612 | 5.84E-12 |
| FTH1P16 | 3.22E-20 | 1.138520699 | 22.19174631 | 5.12E-18 |
| HMBS | 0.001083904 | 1.138418354 | 3.375420926 | 0.00621373 |
| RFTN2 | 9.07E-05 | 1.136152837 | 4.592744606 | 0.000756908 |
| KCNMA1 | 0.001187135 | 1.135552772 | 3.322059504 | 0.006715678 |
| RND1 | 8.84E-05 | 1.135394925 | 4.602568809 | 0.000739391 |
| AC010768.2 | 5.23E-07 | 1.134187044 | 7.124075297 | 8.56E-06 |
| SIGLEC10 | 3.22E-06 | 1.13007915 | 6.206386041 | 4.19E-05 |
| LGALS9 | 0.000117578 | 1.128293114 | 4.433825544 | 0.000938228 |
| AC112504.1 | 0.000616442 | 1.127888169 | 3.620642784 | 0.003863832 |
| AC018730.1 | 0.000368619 | 1.127711898 | 3.871910693 | 0.002508981 |
| ARID3B | 7.20E-07 | 1.127670917 | 6.926721211 | 1.14E-05 |
| ADAT1 | 4.03E-05 | 1.127351 | 4.954885343 | 0.000378437 |
| S100A9 | 1.07E-51 | 1.126891181 | 57.43988916 | 3.28E-48 |
| LAT2 | 0.000503424 | 1.123322058 | 3.704790162 | 0.003276436 |
| MTBP | 6.09E-05 | 1.120618959 | 4.724197398 | 0.000538246 |
| LINC02109 | 0.001499528 | 1.112844438 | 3.142723186 | 0.008164541 |
| MGAT4C | 0.00079305 | 1.110652916 | 3.443800606 | 0.004779835 |
| FLT3LG | 2.87E-05 | 1.110600209 | 5.045071919 | 0.000282885 |
| RENBP | 0.000586072 | 1.109313303 | 3.585354651 | 0.003720427 |
| UTP6 | 3.48E-05 | 1.108619126 | 4.942492547 | 0.000334265 |
| AC114980.1 | 3.03E-05 | 1.10748196 | 5.003851367 | 0.000296711 |
| AC136424.2 | 3.79E-05 | 1.105719439 | 4.888325073 | 0.000360111 |
| MT-TI | 1.73E-07 | 1.100663921 | 7.442420028 | 3.19E-06 |
| PARVB | 4.99E-05 | 1.100649623 | 4.735012796 | 0.000453906 |
| SNHG20 | 0.000236437 | 1.099675371 | 3.987736586 | 0.001720515 |
| GCA | 0.000100342 | 1.098560572 | 4.392614303 | 0.000822785 |
| MRPL30 | 0.001384676 | 1.097192124 | 3.136490242 | 0.00766376 |
| GGT5 | 0.001427422 | 1.0966164 | 3.12036464 | 0.007848476 |
| RSAD2 | 3.76E-05 | 1.096357814 | 4.850697073 | 0.000357464 |
| CYB561A3 | 7.08E-05 | 1.095809077 | 4.547758303 | 0.000610762 |
| AL049839.2 | 6.54E-06 | 1.091777268 | 5.660536459 | 7.85E-05 |
| SERPINA3 | 6.54E-06 | 1.091777268 | 5.660536459 | 7.85E-05 |
| DEFA3 | 3.38E-15 | 1.089209604 | 15.76150314 | 2.69E-13 |
| ZNF600 | 1.36E-05 | 1.088935209 | 5.298461266 | 0.000148611 |
| VWF | 3.69E-09 | 1.08781731 | 9.173383412 | 9.86E-08 |
| AC008761.2 | 7.69E-06 | 1.087431104 | 5.561411328 | 9.04E-05 |

| ALDOA | 8.16E-09 | 1.087137442 | 8.793149982 | 2.02E-07 |
| --- | --- | --- | --- | --- |
| AC069277.1 | 2.38E-06 | 1.086581403 | 6.110778496 | 3.24E-05 |
| ACTR3B | 5.68E-07 | 1.086421617 | 6.785378007 | 9.20E-06 |
| IRAK3 | 1.40E-17 | 1.083706221 | 18.26539117 | 1.63E-15 |
| GPLD1 | 0.000838098 | 1.081334102 | 3.326945985 | 0.005003877 |
| CTSB | 4.28E-17 | 1.081077848 | 17.69584569 | 4.61E-15 |
| DEFA1B | 1.29E-13 | 1.080245453 | 13.92271681 | 7.94E-12 |
| ATAD3C | 0.000570729 | 1.079464817 | 3.501319918 | 0.003640438 |
| CCDC124 | 6.22E-06 | 1.078950766 | 5.617134027 | 7.53E-05 |
| RCSD1 | 0.000419253 | 1.078191262 | 3.641616239 | 0.00279477 |
| ZBTB8A | 0.000467098 | 1.078037182 | 3.590501982 | 0.003067099 |
| AL138963.2 | 0.001451339 | 1.076512049 | 3.055389995 | 0.007953873 |
| DEFA1 | 1.44E-13 | 1.076491337 | 13.82400989 | 8.73E-12 |
| FGD2 | 3.22E-05 | 1.074323292 | 4.826497703 | 0.000311114 |
| AC091492.1 | 0.000291982 | 1.073824027 | 3.795586361 | 0.002065167 |
| BTK | 3.62E-08 | 1.073391339 | 7.987570174 | 7.84E-07 |
| GGA1 | 0.000174671 | 1.073045044 | 4.032265058 | 0.001322779 |
| EFR3B | 4.68E-09 | 1.070333056 | 8.915987697 | 1.22E-07 |
| ZNF765 | 3.24E-05 | 1.065863256 | 4.784741156 | 0.000313479 |
| AC011944.1 | 5.21E-08 | 1.064722099 | 7.754791363 | 1.09E-06 |
| BIVM | 0.00110725 | 1.064199297 | 3.14551182 | 0.006321994 |
| SAMD9 | 8.52E-05 | 1.062460187 | 4.323938352 | 0.000716509 |
| KCNK3 | 2.10E-05 | 1.061340999 | 4.964447601 | 0.000216529 |
| PAAF1 | 5.37E-07 | 1.059536276 | 6.643704613 | 8.75E-06 |
| MAN1C1 | 8.88E-10 | 1.058708613 | 9.583049282 | 2.70E-08 |
| FCER1G | 1.17E-11 | 1.057536619 | 11.55884395 | 5.11E-10 |
| NPC1 | 0.00019885 | 1.055786748 | 3.907967602 | 0.001476748 |
| C21orf91 | 0.000108361 | 1.054182886 | 4.179968598 | 0.000876065 |
| FUT11 | 1.20E-05 | 1.05411109 | 5.187849034 | 0.000133213 |
| CLEC7A | 6.64E-08 | 1.054041594 | 7.565910359 | 1.35E-06 |
| AC008895.1 | 0.000885488 | 1.053686847 | 3.216713594 | 0.005239278 |
| MT-TH | 2.53E-15 | 1.051188353 | 15.3447512 | 2.05E-13 |
| HLA-B | 7.66E-08 | 1.050640479 | 7.476135623 | 1.54E-06 |
| PDE1B | 1.33E-05 | 1.04942846 | 5.118578497 | 0.000145587 |
| AL031595.1 | 2.85E-05 | 1.048591068 | 4.766055373 | 0.000281684 |
| LINC00278 | 2.94E-05 | 1.046898516 | 4.74395285 | 0.000289512 |
| AC099689.1 | 0.001884046 | 1.044694946 | 2.846698072 | 0.009895539 |
| ANO10 | 0.001534122 | 1.044588379 | 2.939617936 | 0.008311145 |
| AC009120.5 | 6.03E-05 | 1.044194727 | 4.406024949 | 0.000534833 |
| LINC02080 | 0.000470293 | 1.042924625 | 3.470468832 | 0.003083683 |
| AC246785.3 | 3.24E-06 | 1.040777328 | 5.712867087 | 4.21E-05 |
| C15orf39 | 5.02E-05 | 1.038745753 | 4.465553594 | 0.000456859 |
| HCK | 3.21E-09 | 1.036482114 | 8.803765728 | 8.65E-08 |
| CPB2-AS1 | 0.001140262 | 1.036115 | 3.049281704 | 0.006486361 |
| FUT2 | 0.000230666 | 1.035872417 | 3.767485001 | 0.001683177 |
| IL10RB | 5.37E-05 | 1.034428077 | 4.417389855 | 0.000483328 |
| TCAF2 | 0.000982624 | 1.034167027 | 3.110373663 | 0.005727571 |
| ZNF674 | 0.001066431 | 1.032735038 | 3.069357951 | 0.006128819 |
| AC009948.2 | 7.50E-08 | 1.032430903 | 7.355816408 | 1.51E-06 |
| POLD1 | 0.000996551 | 1.03228513 | 3.098404503 | 0.005790427 |
| VPS9D1 | 0.000605243 | 1.030791269 | 3.317158834 | 0.003809907 |
| AC097376.3 | 1.53E-05 | 1.028391057 | 4.951833337 | 0.000164443 |

| CASP9 | 0.000527952 | 1.027237458 | 3.366673865 | 0.003415559 |
| --- | --- | --- | --- | --- |
| AC114324.1 | 5.84E-13 | 1.026959479 | 12.5636589 | 3.18E-11 |
| SND1-IT1 | 0.000253863 | 1.026244837 | 3.689760724 | 0.001824271 |
| MTCO2P22 | 3.98E-05 | 1.025558767 | 4.513028083 | 0.000374527 |
| ARPIN | 1.95E-05 | 1.022151257 | 4.813465512 | 0.000203368 |
| AC008434.1 | 0.000318846 | 1.021276132 | 3.570809953 | 0.002216043 |
| PEBP4 | 0.001112938 | 1.020740374 | 3.014786174 | 0.006350539 |
| DEPP1 | 1.37E-07 | 1.020061833 | 7.001795218 | 2.59E-06 |
| AC096632.1 | 6.27E-05 | 1.019378422 | 4.284140969 | 0.000551473 |
| ELN | 9.96E-08 | 1.019066582 | 7.13536586 | 1.95E-06 |
| PTPRO | 4.32E-05 | 1.017235892 | 4.43999878 | 0.000400957 |
| SHMT1 | 0.000295282 | 1.012077671 | 3.572393797 | 0.002082119 |
| AC005519.1 | 0.001250731 | 1.011396515 | 2.935918232 | 0.007027973 |
| AC011481.3 | 1.19E-07 | 1.011083841 | 6.999968797 | 2.29E-06 |
| AP4E1 | 0.001427262 | 1.010784585 | 2.876183917 | 0.007848476 |
| ARPC1B | 3.81E-12 | 1.009847758 | 11.53136122 | 1.81E-10 |
| FUT1 | 0.001573154 | 1.009383223 | 2.829531975 | 0.008492637 |
| ZNF233 | 7.15E-05 | 1.008818852 | 4.182203991 | 0.000616248 |
| Z99129.3 | 0.000627806 | 1.008540211 | 3.229521516 | 0.00392379 |
| ASB13 | 5.35E-07 | 1.008484357 | 6.325116157 | 8.73E-06 |
| RNF144B | 0.001446404 | 1.008295637 | 2.863267741 | 0.007929183 |
| MYO1F | 2.17E-07 | 1.008121289 | 6.717129105 | 3.91E-06 |
| DMAC2L | 0.000762339 | 1.007317001 | 3.140665148 | 0.004629564 |
| FAM126B | 1.13E-12 | 1.006891141 | 12.02772805 | 5.93E-11 |
| ABHD5 | 4.72E-11 | 1.006420748 | 10.39212132 | 1.79E-09 |
| SLC35E3 | 1.22E-08 | 1.006282307 | 7.962774488 | 2.91E-07 |
| DRAXIN | 1.56E-05 | 1.005976201 | 4.834753933 | 0.000167338 |
| CXCR4 | 1.01E-11 | 1.001537072 | 11.01189156 | 4.43E-10 |
| VDR | 0.000378012 | 1.001322644 | 3.42702136 | 0.002565329 |
| KLHL24 | 0.000773152 | 1.000450575 | 3.113137362 | 0.004687502 |
| TTN | 1.48E-15 | -1.000395372 | -14.8346172 | 1.27E-13 |
| AC000089.1 | 0.000794996 | -1.001653591 | -3.104760332 | 0.004788589 |
| LMAN1 | 0.000459289 | -1.002216455 | -3.345311837 | 0.003020132 |
| CENPJ | 6.75E-06 | -1.003594011 | -5.189002587 | 8.09E-05 |
| HP1BP3 | 1.14E-07 | -1.004273082 | -6.972501373 | 2.20E-06 |
| H1-2 | 0.000227299 | -1.004850706 | -3.661075112 | 0.001661241 |
| ATG10 | 1.78E-08 | -1.006690248 | -7.801613027 | 4.11E-07 |
| AC006230.1 | 3.91E-09 | -1.010058827 | -8.49218439 | 1.04E-07 |
| PPP1R15A | 8.16E-13 | -1.011074747 | -12.22194296 | 4.36E-11 |
| HMGXB3 | 7.20E-06 | -1.012852108 | -5.208884604 | 8.56E-05 |
| MUC1 | 9.71E-09 | -1.014773381 | -8.131105876 | 2.37E-07 |
| ABCD3 | 0.001504875 | -1.01574881 | -2.866950469 | 0.008185185 |
| KPNA1 | 2.98E-05 | -1.015814175 | -4.596853182 | 0.000292856 |
| AC090227.1 | 4.59E-05 | -1.015847345 | -4.407012606 | 0.000422147 |
| CD93 | 5.89E-09 | -1.017269933 | -8.371657912 | 1.51E-07 |
| CTNND1 | 0.001320333 | -1.017993246 | -2.931124902 | 0.007358478 |
| RPL26P27 | 0.001349285 | -1.018515312 | -2.923033357 | 0.007488147 |
| ST13 | 0.000136426 | -1.019094137 | -3.938903456 | 0.001066462 |
| DYNLT1 | 0.001168454 | -1.019693091 | -2.990135993 | 0.006624238 |
| DIAPH1 | 0.000501603 | -1.021804116 | -3.371585632 | 0.003265737 |
| RPL31 | 6.26E-08 | -1.02281085 | -7.367997072 | 1.28E-06 |
| FHL1 | 0.000860651 | -1.023894163 | -3.138412819 | 0.005120274 |

| TMEM231 | 1.03E-05 | -1.024333532 | -5.108064379 | 0.000116905 |
| --- | --- | --- | --- | --- |
| SEPTIN10 | 2.64E-05 | -1.027078063 | -4.701953713 | 0.000264606 |
| FMO2 | 4.37E-09 | -1.027268328 | -8.587207427 | 1.14E-07 |
| SYNE2 | 1.60E-10 | -1.027375945 | -10.06501011 | 5.55E-09 |
| MGST1 | 1.16E-07 | -1.027855949 | -7.130600597 | 2.22E-06 |
| AL355309.1 | 0.000360581 | -1.027870565 | -3.538955316 | 0.002466864 |
| AL645608.5 | 7.65E-06 | -1.028291308 | -5.261283444 | 9.01E-05 |
| PCA3 | 0.0007821 | -1.029529182 | -3.19847693 | 0.004727756 |
| PLCB1 | 0.000715821 | -1.029554217 | -3.238149059 | 0.004391945 |
| MTATP8P1 | 0.000215565 | -1.031617972 | -3.782347034 | 0.001589984 |
| PABPC1 | 5.40E-06 | -1.031646261 | -5.434361451 | 6.64E-05 |
| DNM3 | 6.31E-05 | -1.033239347 | -4.339468136 | 0.000554551 |
| CRIP1 | 6.93E-09 | -1.033498815 | -8.432734672 | 1.74E-07 |
| AL928654.3 | 7.23E-09 | -1.033498815 | -8.413495274 | 1.81E-07 |
| RGPD2 | 0.001012613 | -1.03422017 | -3.097030746 | 0.005865261 |
| LRBA | 0.000253666 | -1.034306139 | -3.719094374 | 0.001823562 |
| USP36 | 0.001054075 | -1.037732209 | -3.089462288 | 0.006067268 |
| KIF3A | 7.07E-07 | -1.03819246 | -6.385275436 | 1.12E-05 |
| CIC | 3.25E-05 | -1.038791253 | -4.662431647 | 0.000313858 |
| INAVA | 1.95E-06 | -1.039594562 | -5.936109641 | 2.73E-05 |
| ARHGAP18 | 2.06E-10 | -1.039673692 | -10.06955333 | 7.04E-09 |
| LINC00342 | 4.08E-06 | -1.041537921 | -5.612736705 | 5.20E-05 |
| H2BC5 | 0.00024122 | -1.042608285 | -3.771724914 | 0.001751013 |
| ARRDC3 | 8.63E-05 | -1.043138716 | -4.239413179 | 0.000724235 |
| MYLK | 8.62E-05 | -1.043211301 | -4.240044911 | 0.000724027 |
| SARAF | 9.50E-05 | -1.04359719 | -4.197404326 | 0.000785323 |
| DDX3X | 0.001237677 | -1.043929212 | -3.035112183 | 0.006965355 |
| RCAN1 | 0.000119148 | -1.044440554 | -4.098295718 | 0.000949933 |
| PMEPA1 | 0.000997287 | -1.044532166 | -3.134828983 | 0.005792878 |
| ZNF331 | 9.53E-06 | -1.044924242 | -5.246534906 | 0.000109728 |
| ZRANB2 | 1.02E-10 | -1.045233597 | -10.44453398 | 3.65E-09 |
| GLS | 1.90E-06 | -1.046190935 | -5.986335651 | 2.66E-05 |
| SENP7 | 0.000195542 | -1.04730771 | -3.884212085 | 0.001456881 |
| AL353691.2 | 0.001394332 | -1.04740391 | -2.991002157 | 0.007703316 |
| AHSA2P | 0.001272684 | -1.048000914 | -3.034255427 | 0.007136094 |
| SIAE | 0.000243769 | -1.048918442 | -3.789765508 | 0.001766197 |
| ALCAM | 0.001422064 | -1.049622251 | -2.98835934 | 0.007826026 |
| SRGAP2 | 1.52E-05 | -1.049702097 | -5.057668153 | 0.000163572 |
| HLA-L | 0.001747271 | -1.049747359 | -2.894825092 | 0.009304469 |
| LUC7L3 | 9.23E-08 | -1.050196849 | -7.387777792 | 1.81E-06 |
| CCDC171 | 3.97E-05 | -1.050207013 | -4.622612885 | 0.000373992 |
| MECOM | 0.000175326 | -1.050431931 | -3.945582764 | 0.001326648 |
| IRF1-AS1 | 0.000690725 | -1.051092379 | -3.322181995 | 0.004256393 |
| CCDC141 | 7.21E-08 | -1.051923515 | -7.512865656 | 1.46E-06 |
| PLEKHH2 | 7.04E-08 | -1.052421933 | -7.527108691 | 1.42E-06 |
| CCDC7 | 0.000236423 | -1.052488995 | -3.816652183 | 0.001720515 |
| HNRNPA2B1 | 6.59E-05 | -1.053087828 | -4.403033035 | 0.00057568 |
| MCTP2 | 1.48E-05 | -1.053232922 | -5.088225464 | 0.000159171 |
| CES1 | 3.00E-07 | -1.053249061 | -6.869877532 | 5.25E-06 |
| HBA1 | 6.20E-15 | -1.053979406 | -14.97467316 | 4.70E-13 |
| RPL17P43 | 0.001046486 | -1.054352843 | -3.142252683 | 0.006031122 |
| ELOA | 0.00048209 | -1.055184482 | -3.499911269 | 0.003154303 |

| TUBB6 | 0.000632886 | -1.055520219 | -3.376265923 | 0.003950175 |
| --- | --- | --- | --- | --- |
| HSPB1 | 1.03E-08 | -1.056444986 | -8.439091732 | 2.49E-07 |
| ZC3H6 | 0.000167438 | -1.059370493 | -4.00033817 | 0.001272707 |
| PPFIBP1 | 2.03E-07 | -1.059558201 | -7.090617808 | 3.67E-06 |
| CCDC14 | 1.87E-07 | -1.061897918 | -7.145781542 | 3.40E-06 |
| SNHG22 | 0.000311349 | -1.062165699 | -3.724752923 | 0.002173777 |
| SRGAP2B | 5.13E-06 | -1.065569445 | -5.6365576 | 6.35E-05 |
| TPM2 | 3.46E-06 | -1.066356968 | -5.823305051 | 4.47E-05 |
| ATP2B4 | 2.77E-05 | -1.06670176 | -4.860884623 | 0.000275211 |
| EXPH5 | 0.001053016 | -1.066731312 | -3.176261953 | 0.006063067 |
| AC122718.1 | 1.03E-07 | -1.067130785 | -7.455282535 | 2.00E-06 |
| MIF4GD | 0.000295022 | -1.06797832 | -3.770118724 | 0.002081081 |
| FUBP1 | 2.80E-07 | -1.069154021 | -7.00623111 | 4.97E-06 |
| RAB11FIP1 | 0.000168339 | -1.071143612 | -4.042298287 | 0.001278502 |
| STIM2 | 1.75E-06 | -1.07115952 | -6.166392656 | 2.49E-05 |
| MIR1282 | 0.000398126 | -1.071818992 | -3.644162723 | 0.002683054 |
| RASSF8 | 5.27E-05 | -1.072891989 | -4.590344049 | 0.000475897 |
| OSBPL3 | 0.000599763 | -1.073640791 | -3.459292839 | 0.00378187 |
| WWTR1 | 0.001208117 | -1.074040015 | -3.133931557 | 0.00681763 |
| NAP1L4P1 | 2.46E-09 | -1.074515204 | -9.251412343 | 6.72E-08 |
| CTNNAL1 | 0.000118418 | -1.074980653 | -4.220998907 | 0.000944527 |
| BSCL2 | 1.28E-05 | -1.075752491 | -5.263940877 | 0.000141068 |
| RPSAP9 | 0.001331301 | -1.076999864 | -3.097154204 | 0.007410639 |
| TRIP11 | 1.66E-06 | -1.077361355 | -6.226156166 | 2.38E-05 |
| HSPA5P1 | 0.000113617 | -1.077799742 | -4.251440293 | 0.000911739 |
| OFD1 | 6.49E-05 | -1.078233732 | -4.515259926 | 0.000569023 |
| NONO | 0.000306888 | -1.078339538 | -3.78822787 | 0.002149152 |
| NCALD | 0.000524859 | -1.078741049 | -3.538224482 | 0.003401523 |
| AC136469.2 | 5.74E-05 | -1.080178741 | -4.580985919 | 0.00051157 |
| PCGF5 | 0.000765693 | -1.080255275 | -3.366016328 | 0.004646869 |
| HMGB1P1 | 0.001024573 | -1.080921394 | -3.231368004 | 0.005923364 |
| AL671762.1 | 9.12E-05 | -1.081674154 | -4.370000776 | 0.000759949 |
| IFI27 | 0.000594061 | -1.083273681 | -3.494823995 | 0.003753626 |
| GOLGA8A | 6.85E-06 | -1.083829559 | -5.597259501 | 8.18E-05 |
| COL6A1 | 0.000857447 | -1.084249033 | -3.325166967 | 0.005102863 |
| HSPA8 | 0.000838383 | -1.085808226 | -3.340551597 | 0.005003953 |
| APOD | 1.10E-05 | -1.086283933 | -5.385160084 | 0.000124418 |
| IGKC | 9.95E-06 | -1.086882467 | -5.43691881 | 0.000113623 |
| MPHOSPH6 | 4.54E-06 | -1.087512248 | -5.81081265 | 5.69E-05 |
| AHDC1 | 0.000429817 | -1.088625213 | -3.665092326 | 0.00285795 |
| RPL23 | 0.000114205 | -1.089276747 | -4.294272273 | 0.000915279 |
| RAI1 | 0.000567557 | -1.090450947 | -3.539593338 | 0.003622182 |
| FILIP1 | 2.96E-05 | -1.091095709 | -4.940856014 | 0.000291269 |
| CCDC80 | 1.14E-06 | -1.09179928 | -6.487681399 | 1.72E-05 |
| DES | 4.80E-06 | -1.096696681 | -5.833396794 | 5.98E-05 |
| AP005212.4 | 0.001168209 | -1.096722782 | -3.216116864 | 0.006624238 |
| CSPG4P12 | 1.79E-06 | -1.096922174 | -6.304217388 | 2.53E-05 |
| STX2 | 0.00035091 | -1.097471529 | -3.791549384 | 0.002407117 |
| PTGER4 | 2.11E-10 | -1.097908249 | -10.6224374 | 7.17E-09 |
| CALM2 | 0.000127783 | -1.098385681 | -4.276592992 | 0.001010439 |
| FMC1-LUC7L2 | 4.94E-07 | -1.098451587 | -6.926774292 | 8.14E-06 |
| HSPH1 | 8.01E-11 | -1.098510946 | -11.0906991 | 2.92E-09 |

| GAB1 | 0.000559983 | -1.099265795 | -3.574620209 | 0.003583774 |
| --- | --- | --- | --- | --- |
| ZNF326 | 0.000456113 | -1.100725941 | -3.677445638 | 0.003003237 |
| AP000769.5 | 2.53E-18 | -1.100824927 | -19.37184864 | 3.25E-16 |
| COL14A1 | 0.000521792 | -1.100917612 | -3.613764495 | 0.003384029 |
| OCLN | 0.000727451 | -1.101901757 | -3.457983743 | 0.004447011 |
| CCZ1B | 0.001237697 | -1.10204458 | -3.204068533 | 0.006965355 |
| CHCHD2 | 0.000327368 | -1.102610883 | -3.842559225 | 0.00226521 |
| AP1S1 | 0.00109005 | -1.105236945 | -3.27432383 | 0.006237318 |
| AL009174.1 | 2.35E-06 | -1.105615564 | -6.223882915 | 3.20E-05 |
| CERS6 | 0.000335788 | -1.105750105 | -3.841304007 | 0.002316463 |
| RPS7P1 | 0.000105278 | -1.105775122 | -4.398398232 | 0.000854894 |
| LPAR3 | 0.000950951 | -1.106249626 | -3.342911315 | 0.005576472 |
| CASZ1 | 0.000634714 | -1.106478664 | -3.537879322 | 0.003959339 |
| LBH | 0.000108533 | -1.107306157 | -4.389848428 | 0.000877067 |
| TSTD1 | 8.21E-05 | -1.108718449 | -4.529860005 | 0.000693887 |
| TMSB4XP4 | 3.68E-05 | -1.108737685 | -4.916517294 | 0.000350555 |
| AL662795.2 | 0.001443764 | -1.109640375 | -3.151937711 | 0.007921784 |
| HBA2 | 2.89E-20 | -1.109775339 | -21.68397962 | 4.67E-18 |
| MTIF3 | 0.000959304 | -1.110420205 | -3.351296803 | 0.005614731 |
| LAMC1 | 1.34E-05 | -1.113393274 | -5.424812989 | 0.00014698 |
| CDC37 | 0.001752025 | -1.114962075 | -3.073347906 | 0.009324399 |
| TJP1 | 1.78E-05 | -1.115056001 | -5.294856367 | 0.000187997 |
| PCBP1 | 0.000877083 | -1.115409377 | -3.409761101 | 0.005199567 |
| EIF4A2 | 5.55E-05 | -1.117897487 | -4.757149556 | 0.000497186 |
| PLCB4 | 0.001225544 | -1.119606202 | -3.259924957 | 0.006903287 |
| EFHC1 | 4.35E-15 | -1.119642569 | -16.07943156 | 3.41E-13 |
| ZNF462 | 0.000763167 | -1.120637381 | -3.493452808 | 0.004633067 |
| INO80B-WBP1 | 0.000166195 | -1.121404455 | -4.238214543 | 0.001265875 |
| MIR320D1 | 5.21E-06 | -1.121690255 | -5.926525482 | 6.43E-05 |
| GAS8 | 7.28E-06 | -1.123621861 | -5.772738439 | 8.64E-05 |
| DYNC2LI1 | 0.000292991 | -1.124831274 | -3.974192992 | 0.002069129 |
| PIGR | 0.001018387 | -1.12783952 | -3.374594213 | 0.005893146 |
| PRDX5 | 0.000818423 | -1.128309591 | -3.483116881 | 0.004903883 |
| CLU | 0.001505097 | -1.128489605 | -3.185089223 | 0.008185185 |
| CFAP97 | 0.000261116 | -1.128961282 | -4.045256402 | 0.001868373 |
| CALM1 | 1.95E-06 | -1.131240277 | -6.460287874 | 2.72E-05 |
| AL138963.4 | 0.000170703 | -1.13184416 | -4.264515368 | 0.001294856 |
| CLIC5 | 2.43E-05 | -1.133340863 | -5.230003731 | 0.000246393 |
| PRKAR1A | 5.93E-05 | -1.133888223 | -4.792755517 | 0.000526946 |
| SLC9B1 | 5.49E-05 | -1.135128894 | -4.836163932 | 0.00049268 |
| RPL15P3 | 0.000456231 | -1.13614979 | -3.795666291 | 0.003003237 |
| APBB2 | 0.000246584 | -1.138108469 | -4.106335243 | 0.001783089 |
| MBNL2 | 0.000170971 | -1.138236044 | -4.287823185 | 0.001296355 |
| RPL34 | 1.35E-07 | -1.138303641 | -7.819435387 | 2.56E-06 |
| FERMT2 | 8.08E-06 | -1.13863135 | -5.798754809 | 9.45E-05 |
| FILIP1L | 3.55E-12 | -1.138865595 | -13.03938651 | 1.71E-10 |
| ETS2 | 0.000334326 | -1.139880937 | -3.962032415 | 0.002308828 |
| CCN1 | 2.30E-05 | -1.142123137 | -5.29708399 | 0.000234965 |
| GTF2IP4 | 2.43E-05 | -1.146687821 | -5.291578621 | 0.000246393 |
| SPATA6 | 1.73E-06 | -1.147445257 | -6.611580775 | 2.46E-05 |
| PMS2P1 | 0.000165893 | -1.147897158 | -4.339247667 | 0.001264619 |
| LAMA2 | 3.55E-08 | -1.147933953 | -8.551506067 | 7.72E-07 |

| THY1 | 9.24E-11 | -1.148098328 | -11.52030375 | 3.34E-09 |
| --- | --- | --- | --- | --- |
| AC098614.2 | 0.000817329 | -1.150143536 | -3.551186763 | 0.004899007 |
| ABCA10 | 7.69E-06 | -1.150832996 | -5.885336209 | 9.04E-05 |
| ALDH1A1 | 4.79E-07 | -1.151276276 | -7.275343112 | 7.92E-06 |
| RPL30 | 1.25E-06 | -1.151377027 | -6.79629428 | 1.86E-05 |
| SRGAP1 | 1.11E-06 | -1.152656658 | -6.863244549 | 1.68E-05 |
| ANTXR1 | 0.001035395 | -1.152683957 | -3.440639427 | 0.005980298 |
| H2AC6 | 0.000647262 | -1.155240854 | -3.683970523 | 0.004020837 |
| HBEGF | 3.87E-06 | -1.155523973 | -6.253626508 | 4.94E-05 |
| POLA1 | 2.73E-05 | -1.156651782 | -5.279103617 | 0.000271273 |
| CFB | 1.70E-06 | -1.157976927 | -6.681941723 | 2.42E-05 |
| AL356585.4 | 2.35E-05 | -1.158298011 | -5.361738441 | 0.000239325 |
| DHRS3 | 0.001751683 | -1.159165529 | -3.195291442 | 0.009324399 |
| GOLGA8B | 3.14E-06 | -1.159521071 | -6.380776448 | 4.11E-05 |
| INTU | 0.00021908 | -1.160363944 | -4.246231801 | 0.001612687 |
| HNRNPDL | 1.69E-07 | -1.16259033 | -7.872794964 | 3.13E-06 |
| CSPP1 | 1.88E-08 | -1.163106724 | -8.985429625 | 4.33E-07 |
| ENAH | 1.21E-05 | -1.164237196 | -5.722757455 | 0.000134766 |
| ID4 | 0.000209406 | -1.164336646 | -4.283606776 | 0.001548278 |
| CD55 | 8.01E-06 | -1.164535151 | -5.934708981 | 9.38E-05 |
| RPS7P10 | 0.000252651 | -1.16476511 | -4.190217623 | 0.001817688 |
| CAMK2N1 | 5.66E-05 | -1.165656314 | -4.95058623 | 0.000505674 |
| PKIG | 0.001309536 | -1.166659457 | -3.363342149 | 0.007309362 |
| CPSF6 | 0.000111549 | -1.167601356 | -4.614985388 | 0.000898682 |
| ATP5F1B | 1.25E-05 | -1.170508235 | -5.737808302 | 0.000138596 |
| DNAJC10 | 8.11E-05 | -1.171498208 | -4.792886339 | 0.00068731 |
| UCKL1-AS1 | 0.000107974 | -1.172746683 | -4.651911 | 0.000874088 |
| AP001931.2 | 0.000203635 | -1.173076874 | -4.330000786 | 0.001508632 |
| NEBL | 5.53E-05 | -1.173893567 | -4.997589633 | 0.000495332 |
| HNRNPH1 | 5.04E-06 | -1.176610164 | -6.233483486 | 6.26E-05 |
| TSPYL4 | 0.001686358 | -1.179415194 | -3.270577677 | 0.009012907 |
| RPS27A | 2.63E-07 | -1.180451095 | -7.766550434 | 4.69E-06 |
| GUCY1A1 | 3.55E-05 | -1.181295267 | -5.256679469 | 0.000339663 |
| RPL3P4 | 0.001677572 | -1.183061939 | -3.283373947 | 0.008972212 |
| UQCC2 | 0.000654747 | -1.185144847 | -3.773413785 | 0.004060491 |
| CNTRL | 1.23E-07 | -1.185471966 | -8.192970521 | 2.35E-06 |
| TSPAN13 | 0.001224381 | -1.186407984 | -3.454919005 | 0.006898844 |
| ELOVL5 | 0.000593776 | -1.186812885 | -3.829105908 | 0.003753115 |
| HNRNPM | 6.26E-05 | -1.187259338 | -4.990529406 | 0.000551387 |
| DAAM1 | 0.00040221 | -1.187533073 | -4.032324578 | 0.002702669 |
| CCP110 | 0.000855086 | -1.18795312 | -3.644628373 | 0.005092102 |
| RACK1 | 7.13E-07 | -1.189479267 | -7.311454855 | 1.13E-05 |
| MAPK10 | 0.000102642 | -1.191221944 | -4.751397122 | 0.000836435 |
| AC022868.1 | 0.000172165 | -1.191346154 | -4.484293899 | 0.001304867 |
| TARDBP | 0.00032718 | -1.192385875 | -4.155719139 | 0.00226521 |
| AC010883.3 | 0.001210113 | -1.192715673 | -3.479359412 | 0.006824709 |
| FUT8 | 8.45E-05 | -1.19289426 | -4.858651577 | 0.000711813 |
| ANXA2 | 1.61E-09 | -1.193308448 | -10.49269717 | 4.60E-08 |
| RPSAP17 | 0.000390797 | -1.193314059 | -4.066873097 | 0.002643328 |
| ANKRD20A7P | 9.73E-07 | -1.193717872 | -7.176244376 | 1.49E-05 |
| RBM5 | 0.001044271 | -1.194365727 | -3.560627428 | 0.006023521 |
| SH3GLB2 | 0.000162385 | -1.194651326 | -4.527077468 | 0.001241983 |

| AP003352.1 | 5.57E-07 | -1.196467312 | -7.482547675 | 9.06E-06 |
| --- | --- | --- | --- | --- |
| PTP4A1 | 0.001398409 | -1.197800311 | -3.418960143 | 0.00772156 |
| LRRC37A2 | 3.09E-06 | -1.199301055 | -6.608854533 | 4.05E-05 |
| RPS21 | 0.000541245 | -1.20073041 | -3.922313551 | 0.003475927 |
| AHR | 5.30E-05 | -1.20116778 | -5.13613872 | 0.000478201 |
| GBP2 | 4.72E-05 | -1.203797295 | -5.20775307 | 0.000432839 |
| TPM1 | 0.001781983 | -1.203843769 | -3.309482582 | 0.009467428 |
| LAMC2 | 2.66E-05 | -1.204489937 | -5.510129099 | 0.000265922 |
| SRSF6 | 0.001805521 | -1.20676069 | -3.310624086 | 0.009559355 |
| UBE2Q2P2 | 0.001137877 | -1.207421997 | -3.554535443 | 0.006476792 |
| CIB1 | 4.14E-08 | -1.208022262 | -8.918567411 | 8.91E-07 |
| SOD1 | 5.04E-05 | -1.20908416 | -5.195840257 | 0.00045792 |
| GADD45B | 8.14E-05 | -1.21030544 | -4.949390334 | 0.000688932 |
| PIEZO2 | 0.000341633 | -1.212254159 | -4.202206729 | 0.00235048 |
| RPS23 | 4.32E-10 | -1.212436865 | -11.35441822 | 1.41E-08 |
| EMP1 | 1.68E-11 | -1.212916959 | -13.06942898 | 7.12E-10 |
| ANAPC4 | 0.000761297 | -1.214046454 | -3.78593809 | 0.004624761 |
| RPS7 | 7.81E-06 | -1.214793582 | -6.204339959 | 9.17E-05 |
| ATP9A | 9.27E-05 | -1.215476578 | -4.901784303 | 0.00077026 |
| COL4A1 | 1.00E-08 | -1.217478458 | -9.738233399 | 2.44E-07 |
| CCDC186 | 0.000154996 | -1.218004533 | -4.640208061 | 0.001191901 |
| MMACHC | 1.88E-06 | -1.219854524 | -6.984736699 | 2.64E-05 |
| LRRK2 | 8.42E-09 | -1.219968114 | -9.850827523 | 2.07E-07 |
| RSRP1 | 0.000770423 | -1.220037121 | -3.798305649 | 0.004674037 |
| AL136982.2 | 1.51E-05 | -1.220122763 | -5.881889336 | 0.000162903 |
| RPL12P38 | 0.000107439 | -1.221854219 | -4.849341719 | 0.000870137 |
| C20orf96 | 3.62E-06 | -1.221927703 | -6.648696508 | 4.64E-05 |
| GBP1P1 | 1.92E-07 | -1.22344065 | -8.216574169 | 3.51E-06 |
| ALMS1P1 | 0.001051144 | -1.223456539 | -3.643866793 | 0.006054183 |
| PRDX1 | 1.40E-06 | -1.224589428 | -7.169991597 | 2.04E-05 |
| DDX60 | 3.30E-10 | -1.22464957 | -11.61109906 | 1.10E-08 |
| VMP1 | 2.06E-06 | -1.224717198 | -6.964517536 | 2.85E-05 |
| EVI5 | 9.40E-06 | -1.225261388 | -6.159195612 | 0.000108322 |
| LAPTM4A | 3.63E-05 | -1.225888878 | -5.442628938 | 0.000347002 |
| SPAG1 | 1.39E-05 | -1.226092407 | -5.95580242 | 0.000151168 |
| FDFT1 | 0.00077837 | -1.227267659 | -3.81534666 | 0.004706751 |
| TVP23C-CDRT4 | 7.41E-05 | -1.22864402 | -5.074319307 | 0.000636447 |
| CLMN | 1.90E-05 | -1.229080578 | -5.802714213 | 0.000198935 |
| ANKRD20A8P | 4.20E-07 | -1.229852327 | -7.842957059 | 7.07E-06 |
| CC2D2A | 0.000188568 | -1.230276164 | -4.582202833 | 0.001412344 |
| AKAP6 | 0.000987955 | -1.233372546 | -3.706608479 | 0.005753185 |
| AC083837.1 | 0.000525274 | -1.236409248 | -4.054945354 | 0.003403014 |
| PCSK5 | 0.001440705 | -1.237731901 | -3.516922444 | 0.007907352 |
| AC244197.3 | 0.001542103 | -1.237877942 | -3.480772365 | 0.008349471 |
| CDC42EP3 | 1.04E-06 | -1.239003571 | -7.414415374 | 1.58E-05 |
| CDK5RAP2 | 2.78E-06 | -1.24294857 | -6.90519651 | 3.69E-05 |
| HSP90AA1 | 3.88E-14 | -1.243737196 | -16.68035326 | 2.59E-12 |
| AC245060.5 | 1.86E-06 | -1.244972521 | -7.134172011 | 2.62E-05 |
| BX664727.3 | 1.76E-09 | -1.244982806 | -10.89868155 | 4.98E-08 |
| AL645922.1 | 1.51E-06 | -1.245556614 | -7.250157552 | 2.18E-05 |
| COX7B | 1.95E-06 | -1.245692952 | -7.113718252 | 2.72E-05 |
| HDGF | 7.22E-06 | -1.248754352 | -6.420329752 | 8.58E-05 |

| SNORA16A | 1.98E-05 | -1.250323674 | -5.881945008 | 0.000205403 |
| --- | --- | --- | --- | --- |
| GOLGA8DP | 0.000123506 | -1.25185184 | -4.892626896 | 0.000980017 |
| TRIM2 | 4.48E-08 | -1.255221695 | -9.223944343 | 9.57E-07 |
| JUND | 3.45E-06 | -1.255545352 | -6.857923471 | 4.46E-05 |
| CISD1 | 0.000161939 | -1.256966617 | -4.764719303 | 0.001240117 |
| RN7SL674P | 1.06E-09 | -1.257096475 | -11.28432263 | 3.15E-08 |
| ADAMTS1 | 3.12E-08 | -1.258961787 | -9.44952579 | 6.82E-07 |
| AGAP5 | 4.34E-06 | -1.260078345 | -6.757733659 | 5.46E-05 |
| APH1A | 0.000534249 | -1.260499356 | -4.124677045 | 0.003446108 |
| C16orf71 | 0.001728404 | -1.261097522 | -3.483598734 | 0.009217321 |
| ANKRD20A2P | 5.79E-09 | -1.262370543 | -10.39900488 | 1.48E-07 |
| ITPR3 | 1.74E-05 | -1.262564142 | -6.007726703 | 0.000184592 |
| SLC39A14 | 0.000639598 | -1.264639438 | -4.039375874 | 0.003979937 |
| ANKRD42 | 0.000540811 | -1.265169297 | -4.133250184 | 0.003475927 |
| PHKG2 | 0.000418542 | -1.266315419 | -4.277944564 | 0.002792045 |
| ANKRD20A3P | 1.16E-08 | -1.266500762 | -10.05214704 | 2.78E-07 |
| ICAM1 | 2.77E-07 | -1.266807141 | -8.30791464 | 4.91E-06 |
| AC011511.5 | 1.17E-06 | -1.267102882 | -7.51665394 | 1.75E-05 |
| PDGFRL | 0.000609333 | -1.268131208 | -4.077226413 | 0.003830418 |
| AGAP9 | 9.18E-06 | -1.270670889 | -6.400432581 | 0.000105872 |
| CHTOP | 0.000178373 | -1.27330354 | -4.773196643 | 0.001345831 |
| ALG13 | 0.000221804 | -1.274356221 | -4.656536214 | 0.001628184 |
| AL353743.1 | 1.95E-05 | -1.275038653 | -6.00580556 | 0.000203064 |
| MSI2 | 0.000989284 | -1.277549871 | -3.838627274 | 0.005755686 |
| NPIPB14P | 9.49E-05 | -1.278350721 | -5.14250053 | 0.000784396 |
| REEP1 | 9.55E-05 | -1.279113865 | -5.141813088 | 0.000788858 |
| COBL | 9.76E-05 | -1.279591631 | -5.131664035 | 0.000804173 |
| GTF2IP1 | 6.51E-07 | -1.281761999 | -7.929515031 | 1.04E-05 |
| TPPP | 0.000134069 | -1.283009965 | -4.968675883 | 0.001051606 |
| SREBF1 | 0.001243175 | -1.285772475 | -3.735770389 | 0.006991911 |
| ALDH1A3 | 7.35E-05 | -1.286421142 | -5.318010099 | 0.000631579 |
| NBPF20 | 7.13E-10 | -1.287290527 | -11.77507183 | 2.21E-08 |
| EIF3K | 0.001076101 | -1.288285012 | -3.823819123 | 0.006178612 |
| ADAMTS9 | 4.72E-14 | -1.289069795 | -17.17877656 | 3.10E-12 |
| S100A6 | 4.10E-05 | -1.290681668 | -5.662036202 | 0.000384367 |
| LMLN | 0.000223212 | -1.292855395 | -4.720579777 | 0.001635913 |
| PMS2P7 | 4.08E-06 | -1.297570563 | -6.992482584 | 5.20E-05 |
| LAMA4 | 0.000220358 | -1.297599683 | -4.745154396 | 0.001619505 |
| NDNF | 3.84E-05 | -1.298687816 | -5.735166964 | 0.000363756 |
| GANC | 0.000125345 | -1.299964475 | -5.072320137 | 0.000992475 |
| VPS13A | 2.07E-05 | -1.302091112 | -6.098488806 | 0.000213874 |
| STAG3L1 | 2.50E-05 | -1.302575327 | -5.995030208 | 0.000252036 |
| SYNM | 2.66E-05 | -1.302585071 | -5.959997212 | 0.000265541 |
| KDM6B | 9.31E-05 | -1.303864492 | -5.255816769 | 0.000773209 |
| PUM1 | 0.000728949 | -1.304307343 | -4.09200701 | 0.004454689 |
| AC074183.1 | 0.000100574 | -1.30437175 | -5.214247344 | 0.000823626 |
| AC074183.2 | 0.000100574 | -1.30437175 | -5.214247344 | 0.000823626 |
| CD46 | 5.84E-07 | -1.305846 | -8.140259368 | 9.42E-06 |
| MRFAP1 | 0.000139974 | -1.307695137 | -5.039795617 | 0.001088194 |
| AC005884.1 | 0.000149089 | -1.310324688 | -5.014030536 | 0.001150359 |
| GPC3 | 0.000113529 | -1.316734487 | -5.194378105 | 0.000911446 |
| AC091053.1 | 0.000173061 | -1.318306395 | -4.959206837 | 0.00131112 |

| AMOTL2 | 0.000949089 | -1.319091492 | -3.987208799 | 0.005567323 |
| --- | --- | --- | --- | --- |
| COL15A1 | 5.12E-06 | -1.319142576 | -6.979647372 | 6.33E-05 |
| AP001931.1 | 5.41E-05 | -1.319805222 | -5.630979851 | 0.000486988 |
| AGAP12P | 5.98E-06 | -1.32040303 | -6.896745589 | 7.28E-05 |
| AC078817.1 | 0.001398472 | -1.323864699 | -3.778768235 | 0.00772156 |
| AL136982.1 | 5.10E-05 | -1.32602648 | -5.691313506 | 0.000462662 |
| LRIG1 | 0.001164486 | -1.32707766 | -3.893467711 | 0.006607839 |
| AC139256.2 | 1.36E-12 | -1.329730985 | -15.77973346 | 6.99E-11 |
| KCTD12 | 0.001816676 | -1.330177094 | -3.645646174 | 0.009593655 |
| ARHGEF28 | 0.001619017 | -1.330363051 | -3.712708862 | 0.008696905 |
| AC006042.2 | 0.001182766 | -1.330568407 | -3.894708279 | 0.006693018 |
| CEP112 | 8.86E-07 | -1.330636002 | -8.053885663 | 1.37E-05 |
| RPL10AP2 | 0.000532942 | -1.332446154 | -4.361523023 | 0.00343986 |
| GRAMD2B | 7.37E-06 | -1.334592701 | -6.849788951 | 8.73E-05 |
| MT-RNR1 | 1.85E-15 | -1.335652216 | -19.6781077 | 1.55E-13 |
| AC002398.2 | 7.23E-05 | -1.335690039 | -5.530781774 | 0.000622916 |
| CYTH1 | 0.000164106 | -1.336705105 | -5.059261255 | 0.00125359 |
| AL049873.1 | 0.001549812 | -1.337056089 | -3.756754654 | 0.008386279 |
| SLC2A14 | 2.26E-05 | -1.339163646 | -6.222555991 | 0.000230448 |
| IL32 | 6.39E-07 | -1.340980494 | -8.306418313 | 1.02E-05 |
| CLUAP1 | 1.53E-05 | -1.343274689 | -6.469064952 | 0.00016425 |
| MIR4435-2HG | 2.45E-05 | -1.344606155 | -6.200651112 | 0.000247649 |
| IFT88 | 1.52E-06 | -1.344711789 | -7.823783251 | 2.19E-05 |
| MEIS1 | 5.80E-06 | -1.346284577 | -7.050046144 | 7.07E-05 |
| SNHG19 | 0.000357227 | -1.347003346 | -4.643195079 | 0.002447718 |
| EFCAB2 | 0.000122172 | -1.349428463 | -5.280349906 | 0.000970688 |
| C8orf34 | 0.000564085 | -1.349804811 | -4.385051167 | 0.003602516 |
| EGR1 | 1.82E-06 | -1.3510015 | -7.754027022 | 2.57E-05 |
| TSC22D1 | 0.000575609 | -1.352385635 | -4.381557336 | 0.003664682 |
| TXLNB | 0.000709081 | -1.35688721 | -4.273250869 | 0.004352035 |
| PPP1R12B | 3.82E-10 | -1.358196826 | -12.79209582 | 1.26E-08 |
| AL592430.2 | 9.75E-05 | -1.358583349 | -5.449112978 | 0.000803632 |
| AL136131.3 | 0.000518287 | -1.359055387 | -4.465080907 | 0.003363663 |
| AL136982.4 | 4.25E-05 | -1.359583157 | -5.94412165 | 0.000395316 |
| MED4 | 0.000999644 | -1.359597651 | -4.079002949 | 0.005804743 |
| RPL23AP65 | 6.26E-07 | -1.360065776 | -8.437485789 | 1.00E-05 |
| MSMO1 | 2.41E-10 | -1.360845572 | -13.08851062 | 8.13E-09 |
| CCDC113 | 4.98E-08 | -1.361398601 | -9.941872079 | 1.05E-06 |
| LLGL2 | 9.82E-06 | -1.364926897 | -6.835212756 | 0.000112347 |
| MAP2 | 1.46E-08 | -1.36544037 | -10.6979591 | 3.43E-07 |
| ZNF280D | 0.000251776 | -1.366282477 | -4.917231862 | 0.001812804 |
| DSTN | 2.02E-07 | -1.367397508 | -9.155607578 | 3.65E-06 |
| PRKAA2 | 0.001011791 | -1.36841189 | -4.098269447 | 0.005862341 |
| GRM7 | 0.000435875 | -1.36851988 | -4.599099969 | 0.00289301 |
| LINC00205 | 0.000586384 | -1.36898865 | -4.424322288 | 0.003720427 |
| GOLGA2P7 | 4.92E-08 | -1.371065972 | -10.0198416 | 1.04E-06 |
| AGAP11 | 2.36E-05 | -1.373766622 | -6.357454139 | 0.000239737 |
| PARD3 | 2.83E-06 | -1.374084884 | -7.624671408 | 3.75E-05 |
| ANKRD36 | 2.66E-14 | -1.374502542 | -18.658582 | 1.82E-12 |
| AKR1C3 | 0.000428109 | -1.376532748 | -4.636776131 | 0.002848647 |
| MAP9 | 2.81E-10 | -1.377557504 | -13.15747768 | 9.38E-09 |
| RPGRIP1L | 2.06E-08 | -1.379775233 | -10.60593324 | 4.70E-07 |

| WDR60 | 2.57E-11 | -1.379784582 | -14.61156417 | 1.04E-09 |
| --- | --- | --- | --- | --- |
| ANKRD20A1 | 2.70E-08 | -1.380393613 | -10.44771436 | 5.98E-07 |
| GCLC | 0.001525972 | -1.380426274 | -3.887906237 | 0.008271856 |
| IGFBP5 | 0.000143406 | -1.38044807 | -5.305657769 | 0.001112063 |
| ALDH3B1 | 9.84E-07 | -1.380906758 | -8.295082732 | 1.50E-05 |
| LTO1 | 0.000647796 | -1.381590382 | -4.405286063 | 0.004022799 |
| SEZ6L2 | 3.44E-06 | -1.381776805 | -7.548908657 | 4.45E-05 |
| DDAH1 | 3.40E-07 | -1.382529041 | -8.942074641 | 5.87E-06 |
| TCIM | 3.15E-06 | -1.382949497 | -7.608203513 | 4.12E-05 |
| SCIN | 4.87E-05 | -1.385810609 | -5.976819748 | 0.000444478 |
| BTG3 | 8.78E-05 | -1.386291009 | -5.623719791 | 0.000734719 |
| FAM215B | 1.70E-10 | -1.387089645 | -13.5516953 | 5.85E-09 |
| CYP2B7P | 1.64E-07 | -1.387355602 | -9.41417189 | 3.05E-06 |
| MORF4L1 | 0.000270008 | -1.388125672 | -4.953697595 | 0.00192527 |
| KIF13A | 2.96E-09 | -1.389022314 | -11.84584608 | 8.04E-08 |
| HMGN3 | 1.03E-08 | -1.392920525 | -11.12309627 | 2.50E-07 |
| HSPB6 | 0.000106257 | -1.394617238 | -5.541707443 | 0.000861705 |
| EPS8L2 | 8.48E-05 | -1.395371095 | -5.681427657 | 0.000713735 |
| EGFR | 0.000196549 | -1.396395877 | -5.175782543 | 0.001463197 |
| NPNT | 1.19E-05 | -1.397443325 | -6.87997877 | 0.000132846 |
| ACSBG1 | 0.00070462 | -1.398844431 | -4.409220862 | 0.00432754 |
| RGPD3 | 1.71E-07 | -1.399371177 | -9.469756609 | 3.16E-06 |
| PPP1R32 | 0.000201499 | -1.40167037 | -5.180191031 | 0.001494614 |
| CPE | 7.48E-06 | -1.404618125 | -7.200118972 | 8.84E-05 |
| TOMM34 | 0.000809245 | -1.405960716 | -4.347118317 | 0.004860406 |
| PTPRM | 7.06E-07 | -1.406757071 | -8.653217159 | 1.12E-05 |
| HOMER1 | 0.0009257 | -1.408751461 | -4.27348975 | 0.005449206 |
| ZNF428 | 7.24E-10 | -1.41329615 | -12.9181425 | 2.24E-08 |
| BTG2 | 0.000556233 | -1.413449442 | -4.600415098 | 0.003563488 |
| PTPN13 | 0.00019641 | -1.416112137 | -5.249297255 | 0.001462751 |
| ZNF248 | 0.000661332 | -1.417336203 | -4.506534477 | 0.004091687 |
| ATP1B3 | 4.84E-08 | -1.417872769 | -10.37206377 | 1.02E-06 |
| NDUFA4 | 0.000655775 | -1.418284299 | -4.514746309 | 0.004064131 |
| PLS3 | 1.68E-06 | -1.420074979 | -8.20098976 | 2.40E-05 |
| Z83843.1 | 0.000130814 | -1.423021546 | -5.526083968 | 0.001030462 |
| MLF1 | 0.000315957 | -1.426415698 | -4.99298546 | 0.002199286 |
| MORF4L2 | 4.12E-05 | -1.426450989 | -6.254714359 | 0.000385988 |
| PPP1R16A | 0.001045548 | -1.427740372 | -4.255602789 | 0.006027605 |
| CXCR5 | 9.18E-05 | -1.427760884 | -5.763822843 | 0.000763954 |
| RIC8B | 0.001482616 | -1.427814319 | -4.039245668 | 0.008093986 |
| NPIPA7 | 8.74E-07 | -1.429512673 | -8.660766632 | 1.36E-05 |
| AL080250.1 | 4.94E-09 | -1.431103832 | -11.88724896 | 1.28E-07 |
| AL031681.3 | 0.000880786 | -1.431371264 | -4.373024505 | 0.005219842 |
| C21orf58 | 2.29E-06 | -1.432815247 | -8.080241549 | 3.14E-05 |
| RPL39 | 3.01E-07 | -1.433566093 | -9.348559148 | 5.25E-06 |
| ANKRD36B | 2.73E-13 | -1.43566565 | -18.03648786 | 1.57E-11 |
| HBB | 8.38E-26 | -1.437938582 | -36.05892896 | 3.03E-23 |
| IL7 | 7.04E-05 | -1.438162115 | -5.972054646 | 0.000607934 |
| MAGED2 | 1.14E-06 | -1.439654658 | -8.55573622 | 1.71E-05 |
| MYO5C | 4.93E-06 | -1.441496585 | -7.649630094 | 6.14E-05 |
| MAK | 3.51E-06 | -1.441644683 | -7.864085395 | 4.52E-05 |
| RHEB | 0.000116087 | -1.442424528 | -5.676250995 | 0.000927943 |

| METTL7A | 1.71E-26 | -1.443649635 | -37.19997194 | 6.83E-24 |
| --- | --- | --- | --- | --- |
| AL590560.2 | 3.08E-05 | -1.445356702 | -6.521189403 | 0.000300321 |
| AHNAK2 | 0.001287332 | -1.445493941 | -4.177924981 | 0.00720288 |
| DPY19L1P1 | 0.001793085 | -1.446370516 | -3.972310809 | 0.009513475 |
| STARD7 | 0.000120292 | -1.447153963 | -5.67250272 | 0.000957813 |
| ITGA1 | 3.19E-07 | -1.449943983 | -9.41904159 | 5.54E-06 |
| RIMKLB | 4.78E-05 | -1.450702418 | -6.267399372 | 0.000438054 |
| PLEKHA5 | 4.66E-10 | -1.452395292 | -13.55352711 | 1.51E-08 |
| CDC16 | 1.91E-05 | -1.452662937 | -6.855305531 | 0.000199578 |
| ETNK1 | 0.000823371 | -1.452741902 | -4.480843734 | 0.004930324 |
| LMNA | 0.000135447 | -1.452825213 | -5.619861765 | 0.00106061 |
| RPS24P8 | 2.55E-06 | -1.455562709 | -8.140756125 | 3.43E-05 |
| BCL9L | 6.08E-05 | -1.460365049 | -6.156534332 | 0.000538246 |
| GPRC5A | 0.00012438 | -1.462508589 | -5.711460312 | 0.000985679 |
| ANKRD54 | 0.001520737 | -1.463055599 | -4.122811364 | 0.008253192 |
| TUBA1B | 1.43E-05 | -1.465140803 | -7.098813835 | 0.000155182 |
| AC005670.3 | 0.001615227 | -1.467971134 | -4.098232392 | 0.008684097 |
| MAL2 | 3.14E-05 | -1.469419461 | -6.617704981 | 0.000305077 |
| RPS29 | 3.52E-07 | -1.471177475 | -9.494501327 | 6.04E-06 |
| CCDC66 | 2.67E-09 | -1.472453136 | -12.62360705 | 7.29E-08 |
| SPOCK2 | 0.0003218 | -1.472569717 | -5.142823483 | 0.002235732 |
| AC016831.6 | 1.36E-05 | -1.474192063 | -7.173189877 | 0.000148611 |
| EZR | 1.83E-10 | -1.475323758 | -14.36521891 | 6.28E-09 |
| FAT1 | 0.000100579 | -1.476359008 | -5.901737325 | 0.000823626 |
| CYTOR | 3.34E-06 | -1.476415284 | -8.084546407 | 4.33E-05 |
| CLIP4 | 2.76E-06 | -1.477455075 | -8.212947427 | 3.67E-05 |
| CKB | 1.25E-07 | -1.477793981 | -10.20025477 | 2.40E-06 |
| VCL | 4.24E-05 | -1.478085588 | -6.463526943 | 0.000394908 |
| MYLK-AS1 | 0.000268377 | -1.481670966 | -5.291424985 | 0.001915121 |
| RERE | 2.76E-11 | -1.483166107 | -15.66174447 | 1.10E-09 |
| TNC | 0.000241293 | -1.483537854 | -5.366632091 | 0.001751013 |
| CCND1 | 5.07E-07 | -1.484867047 | -9.346703914 | 8.34E-06 |
| DCN | 3.45E-07 | -1.485031106 | -9.597048539 | 5.94E-06 |
| ZC2HC1A | 4.19E-06 | -1.486927903 | -7.996849969 | 5.31E-05 |
| CSPG4P10 | 6.38E-09 | -1.48734541 | -12.18951336 | 1.62E-07 |
| CEP170B | 0.000670115 | -1.487726774 | -4.72182239 | 0.004141856 |
| GLRX3 | 0.000935287 | -1.489421716 | -4.511540184 | 0.005496864 |
| GADD45A | 4.61E-07 | -1.490139951 | -9.442564213 | 7.64E-06 |
| CLDN7 | 0.000790827 | -1.490316062 | -4.622839272 | 0.004771114 |
| RGS2 | 1.25E-06 | -1.490753443 | -8.79760056 | 1.86E-05 |
| MTSS2 | 0.000113982 | -1.494023675 | -5.891182768 | 0.000913886 |
| RPL13AP7 | 2.38E-05 | -1.494208324 | -6.909310168 | 0.000241651 |
| LIMA1 | 1.11E-10 | -1.49609001 | -14.89130522 | 3.96E-09 |
| ZNF518A | 0.000484501 | -1.496572696 | -4.960697819 | 0.0031667 |
| CD59 | 7.25E-07 | -1.496873933 | -9.189867613 | 1.15E-05 |
| PTGS2 | 1.28E-06 | -1.497733037 | -8.825738922 | 1.89E-05 |
| FAM161A | 3.63E-05 | -1.499190225 | -6.65568292 | 0.000347002 |
| SLC4A4 | 4.53E-07 | -1.499213216 | -9.511410064 | 7.54E-06 |
| RND3 | 9.11E-06 | -1.499560094 | -7.558797693 | 0.000105309 |
| TC2N | 7.57E-07 | -1.50187745 | -9.193212462 | 1.19E-05 |
| RFX3-AS1 | 0.000388714 | -1.502240778 | -5.123196803 | 0.002631173 |
| AC022400.3 | 1.03E-06 | -1.503951896 | -9.002253864 | 1.57E-05 |

| CXADR | 0.001061025 | -1.505324829 | -4.477248894 | 0.006105369 |
| --- | --- | --- | --- | --- |
| HMGCS1 | 1.80E-06 | -1.505797203 | -8.648755798 | 2.55E-05 |
| AADAT | 0.000631508 | -1.506254209 | -4.819442771 | 0.003942913 |
| C6orf132 | 1.11E-07 | -1.507747067 | -10.48853932 | 2.14E-06 |
| CEP89 | 0.000126877 | -1.508527379 | -5.878153003 | 0.00100374 |
| HLA-DRA | 1.78E-06 | -1.51071154 | -8.684284761 | 2.53E-05 |
| AC079328.2 | 4.67E-05 | -1.510756735 | -6.542275529 | 0.000429155 |
| DDAH2 | 0.000401378 | -1.514276203 | -5.143157675 | 0.002699049 |
| DUSP1 | 1.11E-07 | -1.516225197 | -10.54632971 | 2.14E-06 |
| SYNE1 | 2.26E-10 | -1.516368732 | -14.62620164 | 7.66E-09 |
| AZIN1-AS1 | 6.50E-10 | -1.518790132 | -13.95325022 | 2.03E-08 |
| COL21A1 | 0.000792353 | -1.519927647 | -4.713419275 | 0.004777193 |
| BAIAP2L1 | 8.80E-06 | -1.520007835 | -7.684502231 | 0.000101983 |
| AC087521.1 | 1.71E-06 | -1.522840877 | -8.781694449 | 2.44E-05 |
| SEPTIN5 | 5.37E-05 | -1.523223172 | -6.503805786 | 0.000483642 |
| PTGDS | 4.54E-05 | -1.524587562 | -6.620890075 | 0.000418442 |
| SS18L2 | 0.001557469 | -1.526366239 | -4.28539615 | 0.008417826 |
| FST | 8.01E-11 | -1.527022684 | -15.41749047 | 2.92E-09 |
| ACAA1 | 5.59E-05 | -1.527352277 | -6.495622831 | 0.000499661 |
| LRRC37A17P | 0.00030912 | -1.531660184 | -5.375931582 | 0.002160677 |
| FN1 | 9.25E-10 | -1.532544907 | -13.84488588 | 2.81E-08 |
| SLFN13 | 8.28E-08 | -1.533364767 | -10.85943756 | 1.65E-06 |
| WDR54 | 5.52E-06 | -1.533904469 | -8.065382115 | 6.77E-05 |
| WLS | 0.000131603 | -1.534222726 | -5.953910411 | 0.001036233 |
| RHOB | 0.000235581 | -1.536081518 | -5.572689684 | 0.001716319 |
| ATP1B1 | 1.83E-05 | -1.539050305 | -7.293012345 | 0.000191968 |
| RGS1 | 1.69E-07 | -1.539627815 | -10.42531779 | 3.13E-06 |
| WEE1 | 0.000405922 | -1.540019661 | -5.223064425 | 0.00272265 |
| CCN2 | 0.000562936 | -1.540796309 | -5.006880524 | 0.003597676 |
| SNORD3D | 2.57E-06 | -1.541472857 | -8.618174237 | 3.44E-05 |
| AC007952.4 | 5.80E-06 | -1.541472857 | -8.072026423 | 7.07E-05 |
| RGPD1 | 0.000130811 | -1.543137673 | -5.992552493 | 0.001030462 |
| NDUFAB1 | 1.25E-06 | -1.544711716 | -9.118395206 | 1.86E-05 |
| LAMB3 | 1.25E-06 | -1.548490183 | -9.141105387 | 1.86E-05 |
| AP000426.1 | 0.000314534 | -1.54854836 | -5.423530581 | 0.002191866 |
| CCDC88C | 1.68E-06 | -1.552006095 | -8.961173517 | 2.40E-05 |
| ZMAT1 | 1.31E-11 | -1.553111536 | -16.89977765 | 5.69E-10 |
| AC055811.1 | 0.000505142 | -1.555934366 | -5.12927198 | 0.003286457 |
| RPA3 | 0.00161569 | -1.556401952 | -4.34491711 | 0.008684097 |
| SLC20A1 | 3.20E-06 | -1.556800224 | -8.554762538 | 4.17E-05 |
| RUVBL1 | 6.85E-05 | -1.56381114 | -6.511840828 | 0.000593685 |
| TOX3 | 4.44E-05 | -1.566319333 | -6.81747283 | 0.000411367 |
| MEF2C | 4.27E-05 | -1.566524098 | -6.844401827 | 0.000397519 |
| PTPN21 | 0.00131265 | -1.571759606 | -4.529576858 | 0.007323928 |
| LINC01578 | 0.000565526 | -1.571837292 | -5.104615932 | 0.003610471 |
| DNMBP | 0.000366697 | -1.572181643 | -5.401533314 | 0.002500514 |
| GOLGA2P10 | 4.97E-09 | -1.572464318 | -13.05742043 | 1.28E-07 |
| UGCG | 3.79E-09 | -1.573018647 | -13.24670492 | 1.01E-07 |
| AC027612.3 | 0.001899665 | -1.573252866 | -4.281329229 | 0.009969047 |
| ACLY | 0.000376773 | -1.573477885 | -5.387462371 | 0.002558808 |
| ICAM2 | 0.00010257 | -1.57838828 | -6.296159753 | 0.000836217 |
| IFT57 | 1.03E-06 | -1.579656104 | -9.458502603 | 1.57E-05 |

| PIM3 | 9.87E-07 | -1.584718227 | -9.517370565 | 1.51E-05 |
| --- | --- | --- | --- | --- |
| CASTOR3 | 0.001473437 | -1.584789467 | -4.487598323 | 0.008046258 |
| HSPB2-C11orf52 | 1.99E-05 | -1.588809253 | -7.467701321 | 0.000207024 |
| KIF19 | 0.000177708 | -1.589000508 | -5.959218567 | 0.001341912 |
| NPHP3-ACAD11 | 1.29E-09 | -1.589351342 | -14.12913009 | 3.77E-08 |
| FBN1 | 0.000136399 | -1.595142673 | -6.165529624 | 0.001066462 |
| NEK1 | 4.65E-08 | -1.595185431 | -11.69621611 | 9.87E-07 |
| PALMD | 5.10E-06 | -1.595797146 | -8.445379473 | 6.32E-05 |
| ARHGEF33 | 2.91E-07 | -1.59621147 | -10.43299717 | 5.11E-06 |
| PDGFRB | 0.000132093 | -1.596243527 | -6.192020288 | 0.001039203 |
| FAM118A | 1.57E-05 | -1.596457646 | -7.669681098 | 0.000167855 |
| TTC21B | 4.35E-06 | -1.598745519 | -8.571749402 | 5.47E-05 |
| GBP1 | 1.09E-09 | -1.598903717 | -14.33339459 | 3.23E-08 |
| UACA | 4.25E-17 | -1.599067711 | -26.17990596 | 4.60E-15 |
| NAALADL2 | 0.000268144 | -1.600701996 | -5.717119252 | 0.001914199 |
| JHY | 0.000834556 | -1.601851983 | -4.93137245 | 0.004984343 |
| INSIG1 | 0.001312941 | -1.6028113 | -4.618909272 | 0.007323928 |
| REPS1 | 0.000631122 | -1.605998788 | -5.139013645 | 0.003941841 |
| RAN | 7.57E-05 | -1.60627794 | -6.619646653 | 0.000648425 |
| GSTP1 | 2.60E-09 | -1.606789321 | -13.79363663 | 7.11E-08 |
| SPICE1 | 0.000122763 | -1.606805095 | -6.284106054 | 0.000974541 |
| GLIS3 | 0.000223986 | -1.60681362 | -5.864514357 | 0.00164028 |
| ABCA5 | 7.66E-06 | -1.61025008 | -8.237229174 | 9.02E-05 |
| CCDC92 | 3.05E-06 | -1.612509362 | -8.893587914 | 4.01E-05 |
| AKAP12 | 7.06E-22 | -1.614810399 | -34.15518462 | 1.45E-19 |
| JPT2 | 2.74E-06 | -1.616335933 | -8.990488462 | 3.64E-05 |
| IQCG | 4.00E-07 | -1.618032385 | -10.35202012 | 6.79E-06 |
| AKAP9 | 5.33E-10 | -1.618641562 | -15.00966247 | 1.71E-08 |
| CEP290 | 3.74E-12 | -1.621710944 | -18.53076719 | 1.79E-10 |
| SGSM2 | 0.000518785 | -1.622981667 | -5.331515265 | 0.003365709 |
| WDR34 | 9.88E-07 | -1.629935337 | -9.788161756 | 1.51E-05 |
| PGRMC1 | 0.000824687 | -1.629949651 | -5.026293217 | 0.004936602 |
| TTLL7 | 4.24E-06 | -1.632215443 | -8.768706181 | 5.37E-05 |
| C2orf50 | 6.37E-06 | -1.633415794 | -8.486470821 | 7.68E-05 |
| TTC12 | 0.000282091 | -1.633495821 | -5.798274537 | 0.002004445 |
| AL355312.6 | 9.66E-06 | -1.634866444 | -8.198752178 | 0.000110916 |
| DNAAF4-CCPG1 | 7.24E-10 | -1.636833255 | -14.96065192 | 2.24E-08 |
| HHAT | 0.000158144 | -1.636932411 | -6.221894139 | 0.001214404 |
| SDC4 | 0.000725437 | -1.639163869 | -5.145991887 | 0.004439113 |
| AL513478.4 | 9.71E-11 | -1.641929491 | -16.44034998 | 3.49E-09 |
| BCO2 | 0.000576906 | -1.644042557 | -5.324881665 | 0.00367167 |
| PSENEN | 7.61E-07 | -1.644487171 | -10.06188696 | 1.19E-05 |
| LMO7 | 3.12E-12 | -1.646348451 | -18.94266962 | 1.51E-10 |
| NDEL1 | 6.13E-10 | -1.647907004 | -15.18100083 | 1.93E-08 |
| HSPA12A | 1.46E-05 | -1.650802144 | -7.982505111 | 0.000157912 |
| LRRC27 | 0.000301594 | -1.652793954 | -5.818789525 | 0.00211705 |
| ANK2 | 0.000415057 | -1.653168434 | -5.590837034 | 0.00277483 |
| MFSD4A | 0.00045919 | -1.653457633 | -5.519254323 | 0.003020132 |
| OSBPL6 | 3.21E-05 | -1.654160333 | -7.432047807 | 0.000311027 |
| G0S2 | 1.93E-05 | -1.657857382 | -7.815111846 | 0.000201574 |
| NPIPA3 | 2.43E-11 | -1.658252214 | -17.60058397 | 9.89E-10 |
| AKR1C2 | 2.86E-08 | -1.659487709 | -12.51958341 | 6.30E-07 |

| SLF1 | 7.32E-05 | -1.659905646 | -6.864429335 | 0.00062973 |
| --- | --- | --- | --- | --- |
| NPIPA8 | 3.88E-10 | -1.665415096 | -15.67353579 | 1.27E-08 |
| CGN | 3.88E-05 | -1.66580285 | -7.347633234 | 0.000367809 |
| AC126755.7 | 3.61E-10 | -1.668374207 | -15.75391761 | 1.19E-08 |
| AL591684.1 | 7.78E-07 | -1.670425304 | -10.20503894 | 1.22E-05 |
| PRR29 | 6.33E-05 | -1.670488898 | -7.013885875 | 0.000555776 |
| S100A16 | 5.57E-10 | -1.675143969 | -15.50184631 | 1.77E-08 |
| SRGAP3 | 7.96E-11 | -1.679270185 | -16.95891215 | 2.92E-09 |
| C11orf96 | 2.52E-10 | -1.679378541 | -16.11974007 | 8.44E-09 |
| ALOX12P2 | 0.000291781 | -1.67987527 | -5.938263009 | 0.002065167 |
| TMEM254 | 0.000298116 | -1.681107283 | -5.926936937 | 0.002097283 |
| IGFBP7 | 9.57E-09 | -1.684187903 | -13.50530104 | 2.34E-07 |
| TUBA1A | 2.03E-15 | -1.684912586 | -24.75711463 | 1.67E-13 |
| BBS4 | 0.000405015 | -1.686941522 | -5.722998517 | 0.002717551 |
| AC211476.11 | 0.000494664 | -1.68960125 | -5.585297541 | 0.003223977 |
| C11orf52 | 8.60E-06 | -1.6903362 | -8.562096057 | 9.98E-05 |
| AC073389.2 | 3.46E-07 | -1.692870271 | -10.93720544 | 5.96E-06 |
| LRRC74B | 1.71E-11 | -1.695634872 | -18.25793587 | 7.22E-10 |
| AL353729.1 | 0.00046414 | -1.698835552 | -5.662814746 | 0.003048765 |
| SLC5A3 | 2.21E-05 | -1.69960963 | -7.913760524 | 0.000225961 |
| MUC20 | 8.16E-09 | -1.69992072 | -13.74992559 | 2.02E-07 |
| SYTL2 | 3.03E-08 | -1.706988375 | -12.83288568 | 6.66E-07 |
| THBS1 | 4.11E-11 | -1.707170118 | -17.73178945 | 1.58E-09 |
| MAGI3 | 1.36E-08 | -1.708770034 | -13.44372619 | 3.20E-07 |
| DYNLL1 | 9.67E-07 | -1.70967133 | -10.28277312 | 1.48E-05 |
| JUNB | 3.22E-06 | -1.709984133 | -9.390981381 | 4.19E-05 |
| MORN1 | 0.000417463 | -1.710370462 | -5.779994915 | 0.00278889 |
| MED24 | 2.12E-06 | -1.712283628 | -9.716028306 | 2.92E-05 |
| AL049629.2 | 4.59E-06 | -1.712759905 | -9.143733125 | 5.74E-05 |
| LINC02832 | 3.09E-06 | -1.713860039 | -9.442983442 | 4.05E-05 |
| PEG10 | 3.14E-05 | -1.713946706 | -7.718474285 | 0.000305116 |
| NHS | 3.89E-05 | -1.714705701 | -7.561114309 | 0.000368529 |
| AP001329.1 | 0.000181951 | -1.715343488 | -6.415464612 | 0.00136946 |
| AGER | 3.08E-11 | -1.718568582 | -18.06458511 | 1.22E-09 |
| ANKRD36C | 1.30E-14 | -1.719575219 | -23.87891345 | 9.49E-13 |
| H2BC4 | 4.42E-07 | -1.720854665 | -10.93602776 | 7.38E-06 |
| NFKBIZ | 6.66E-05 | -1.72152713 | -7.189748281 | 0.00058093 |
| DSP | 7.52E-14 | -1.722186582 | -22.60128543 | 4.80E-12 |
| RASEF | 0.000703631 | -1.723140928 | -5.43246866 | 0.004324805 |
| AL357093.1 | 2.37E-07 | -1.723767696 | -11.42108098 | 4.24E-06 |
| ICA1L | 9.09E-06 | -1.724431531 | -8.693680807 | 0.000105193 |
| BHLHE40 | 1.10E-09 | -1.726531493 | -15.46796693 | 3.27E-08 |
| GOLGA8Q | 1.28E-06 | -1.728216349 | -10.18649237 | 1.89E-05 |
| ATP5IF1 | 1.53E-07 | -1.72972858 | -11.78905282 | 2.86E-06 |
| NPIPA9 | 9.81E-09 | -1.731416004 | -13.86573155 | 2.39E-07 |
| PROM1 | 2.78E-15 | -1.733765555 | -25.23657383 | 2.24E-13 |
| CFAP221 | 2.21E-09 | -1.73423876 | -15.01140814 | 6.10E-08 |
| BCL3 | 2.59E-06 | -1.734284341 | -9.687767868 | 3.47E-05 |
| PITPNB | 4.21E-05 | -1.736735739 | -7.599541992 | 0.000392915 |
| NRXN3 | 0.000386888 | -1.737610824 | -5.929448292 | 0.002620741 |
| TTLL6 | 9.24E-07 | -1.738738203 | -10.49242409 | 1.42E-05 |
| CEP126 | 1.78E-14 | -1.740240182 | -23.927408 | 1.27E-12 |

| CFAP298 | 1.83E-06 | -1.741252536 | -9.9892872 | 2.58E-05 |
| --- | --- | --- | --- | --- |
| PLEKHH1 | 0.000239905 | -1.741797713 | -6.305238969 | 0.001743001 |
| PDLIM4 | 0.000138552 | -1.743514155 | -6.727153224 | 0.001078504 |
| CCDC34 | 4.73E-08 | -1.743534647 | -12.7721466 | 1.00E-06 |
| INO80B | 1.11E-06 | -1.745261805 | -10.39458738 | 1.68E-05 |
| PERP | 2.32E-08 | -1.74634927 | -13.33237842 | 5.23E-07 |
| HSPA4L | 2.72E-05 | -1.746474649 | -7.974552029 | 0.000270219 |
| ZNF273 | 2.54E-07 | -1.752226739 | -11.55703608 | 4.54E-06 |
| HLA-DPA1 | 0.00012186 | -1.754878355 | -6.868840022 | 0.000969457 |
| CFAP298-TCP10L | 9.67E-07 | -1.755767904 | -10.56018852 | 1.48E-05 |
| PTPRU | 4.18E-07 | -1.75801072 | -11.21396114 | 7.05E-06 |
| RN7SL128P | 2.43E-14 | -1.761232903 | -23.97685285 | 1.67E-12 |
| COPRS | 4.56E-07 | -1.761537236 | -11.16914376 | 7.58E-06 |
| AP001062.3 | 2.01E-05 | -1.763508726 | -8.284061255 | 0.000207847 |
| NPTN | 1.47E-05 | -1.764787151 | -8.529358405 | 0.000158709 |
| NCKAP5 | 5.99E-14 | -1.766989489 | -23.36456955 | 3.90E-12 |
| APBA3 | 0.000588126 | -1.767451694 | -5.709805545 | 0.003725645 |
| LINC00472 | 1.33E-08 | -1.772791586 | -13.96548068 | 3.13E-07 |
| DDX17 | 3.13E-13 | -1.776522689 | -22.2146207 | 1.77E-11 |
| MORN2 | 0.000702939 | -1.777111965 | -5.603380648 | 0.00432298 |
| CCDC144A | 4.14E-09 | -1.779457442 | -14.91646816 | 1.09E-07 |
| WDR3 | 3.82E-07 | -1.782871006 | -11.44140134 | 6.51E-06 |
| MDM1 | 0.001277162 | -1.78393944 | -5.162282036 | 0.007154669 |
| ANKMY1 | 2.82E-07 | -1.784333011 | -11.68751666 | 4.99E-06 |
| P4HA2 | 3.18E-05 | -1.788558193 | -8.042986598 | 0.000309009 |
| AL137782.1 | 2.91E-07 | -1.790579905 | -11.70343587 | 5.11E-06 |
| MYH10 | 5.13E-12 | -1.792454952 | -20.23724571 | 2.39E-10 |
| DMKN | 2.65E-05 | -1.792528946 | -8.203604773 | 0.00026505 |
| PTOV1-AS1 | 0.000535602 | -1.79371538 | -5.867525625 | 0.003449309 |
| MYEF2 | 3.73E-05 | -1.795012228 | -7.9481925 | 0.000355096 |
| UBA1 | 5.07E-09 | -1.796809315 | -14.9038795 | 1.31E-07 |
| STRBP | 8.17E-08 | -1.797441797 | -12.73966527 | 1.63E-06 |
| AC079594.2 | 0.000238305 | -1.797483021 | -6.512041155 | 0.001733428 |
| DZIP3 | 6.30E-10 | -1.799596907 | -16.55785262 | 1.97E-08 |
| EPCAM | 2.41E-06 | -1.801118995 | -10.11974621 | 3.26E-05 |
| CAP2 | 0.001089475 | -1.801158509 | -5.336441232 | 0.006235967 |
| TMEM234 | 0.000387512 | -1.802301753 | -6.148939262 | 0.002624003 |
| CEP162 | 3.04E-08 | -1.804810496 | -13.56664997 | 6.66E-07 |
| Z80897.1 | 0.000162693 | -1.804841323 | -6.837878801 | 0.001243824 |
| SH2D4A | 1.91E-05 | -1.807201204 | -8.528258177 | 0.000199578 |
| PLPP3 | 0.000124664 | -1.809220778 | -7.063667649 | 0.000987502 |
| RRAS2 | 2.95E-05 | -1.809678266 | -8.198799772 | 0.000289962 |
| ZDBF2 | 2.04E-05 | -1.809940571 | -8.490185417 | 0.000210923 |
| PER3 | 0.00033286 | -1.810399146 | -6.296095778 | 0.002299564 |
| TBC1D19 | 0.001534741 | -1.810535325 | -5.094782998 | 0.008312051 |
| FAM184A | 1.33E-10 | -1.811296761 | -17.89123306 | 4.64E-09 |
| ANKRD45 | 0.000174946 | -1.814522781 | -6.817336275 | 0.001324314 |
| PDCL3P5 | 0.000120292 | -1.814678173 | -7.113108821 | 0.000957813 |
| CCDC162P | 6.07E-05 | -1.820443153 | -7.676918529 | 0.000537125 |
| MGMT | 0.000622539 | -1.821032588 | -5.837927402 | 0.003894831 |
| CFAP69 | 4.55E-08 | -1.821287188 | -13.37149972 | 9.67E-07 |
| MT1F | 1.21E-05 | -1.826287803 | -8.981700268 | 0.000134219 |

| H2AZ1 | 4.70E-07 | -1.832310386 | -11.59449325 | 7.78E-06 |
| --- | --- | --- | --- | --- |
| DNAH1 | 0.000960753 | -1.834876935 | -5.536536075 | 0.005621427 |
| TRIM28 | 0.000968673 | -1.837063943 | -5.536585131 | 0.005658783 |
| CFAP410 | 0.00012859 | -1.838731172 | -7.154120629 | 0.001015549 |
| RSPH9 | 6.08E-06 | -1.84117093 | -9.603570659 | 7.38E-05 |
| ANK3 | 2.12E-11 | -1.841311091 | -19.65446305 | 8.81E-10 |
| C17orf97 | 3.25E-08 | -1.842710119 | -13.79930668 | 7.07E-07 |
| AC125807.1 | 1.76E-09 | -1.84436615 | -16.14698587 | 4.98E-08 |
| ZFP36 | 1.55E-08 | -1.847695888 | -14.43177434 | 3.62E-07 |
| NPIPA1 | 1.40E-12 | -1.856573609 | -22.00666769 | 7.15E-11 |
| SCARA3 | 7.65E-05 | -1.858425795 | -7.649970042 | 0.000654292 |
| ABCA9 | 8.59E-07 | -1.8587768 | -11.27550894 | 1.33E-05 |
| SPATA18 | 2.63E-06 | -1.862765736 | -10.39532966 | 3.51E-05 |
| PTRH1 | 6.73E-06 | -1.865525604 | -9.648984841 | 8.06E-05 |
| IER3-AS1 | 7.03E-10 | -1.866975126 | -17.08851793 | 2.18E-08 |
| DLEC1 | 1.14E-07 | -1.867665703 | -12.9706208 | 2.19E-06 |
| GLB1L | 2.05E-11 | -1.871771072 | -20.00441981 | 8.56E-10 |
| CDRT4 | 0.001461372 | -1.874252643 | -5.31395446 | 0.007999342 |
| NUCB2 | 7.93E-08 | -1.874382495 | -13.30983482 | 1.58E-06 |
| GALNT3 | 2.24E-05 | -1.88107859 | -8.745726615 | 0.000229138 |
| LINC00958 | 0.000190475 | -1.883598637 | -7.007290963 | 0.001424313 |
| SPARC | 8.14E-09 | -1.88371921 | -15.23824252 | 2.02E-07 |
| CEP128 | 1.84E-07 | -1.884747214 | -12.69229916 | 3.37E-06 |
| AC241377.4 | 0.00180218 | -1.885922204 | -5.175351092 | 0.009554752 |
| PLPP2 | 0.000292553 | -1.886517519 | -6.666567568 | 0.002067354 |
| AK9 | 1.34E-11 | -1.888690519 | -20.53604815 | 5.78E-10 |
| MAPRE3 | 0.00010139 | -1.890553904 | -7.550877501 | 0.000828326 |
| IER3 | 1.38E-09 | -1.891085413 | -16.75615027 | 4.01E-08 |
| C19orf44 | 5.41E-06 | -1.891318369 | -9.961631849 | 6.65E-05 |
| AC097658.2 | 0.000621023 | -1.891995495 | -6.067426281 | 0.003886665 |
| ZNF334 | 0.000540952 | -1.896141589 | -6.19439326 | 0.003475927 |
| TSPAN3 | 5.93E-07 | -1.896338673 | -11.80781109 | 9.56E-06 |
| ALMS1 | 2.57E-07 | -1.896567067 | -12.49769762 | 4.59E-06 |
| SAFB | 2.90E-07 | -1.904695259 | -12.45334504 | 5.09E-06 |
| LRRCC1 | 5.76E-06 | -1.908277047 | -9.998070336 | 7.04E-05 |
| AC241585.2 | 0.001486838 | -1.908521949 | -5.396797003 | 0.008109824 |
| DNAJA4 | 9.48E-09 | -1.912347159 | -15.34304202 | 2.33E-07 |
| SRSF5 | 1.42E-12 | -1.915689752 | -22.69734995 | 7.22E-11 |
| AC017099.1 | 5.68E-07 | -1.916448583 | -11.96932311 | 9.20E-06 |
| TGFB2 | 2.75E-05 | -1.919506121 | -8.75315664 | 0.000273646 |
| LINC00271 | 1.64E-08 | -1.92107468 | -14.9574043 | 3.81E-07 |
| SMYD2 | 0.000306275 | -1.921935098 | -6.753465968 | 0.002145671 |
| FLACC1 | 1.97E-11 | -1.92353437 | -20.59211812 | 8.25E-10 |
| CHRM3 | 0.000122064 | -1.924793446 | -7.532510089 | 0.000970246 |
| AC016747.4 | 3.23E-07 | -1.932006013 | -12.53932165 | 5.59E-06 |
| PPOX | 1.37E-05 | -1.936120996 | -9.417491715 | 0.000149079 |
| DCDC2 | 2.12E-05 | -1.936402079 | -9.051407458 | 0.000217755 |
| AC004593.2 | 4.87E-05 | -1.937968456 | -8.358169159 | 0.000444478 |
| AC145124.1 | 1.12E-05 | -1.939620789 | -9.604860489 | 0.000125345 |
| PCLO | 1.32E-09 | -1.941663716 | -17.24153475 | 3.85E-08 |
| FOSB | 8.54E-06 | -1.944534922 | -9.855763075 | 9.92E-05 |
| SORBS1 | 3.85E-08 | -1.946108343 | -14.42870924 | 8.31E-07 |

| ANKRD23 | 1.59E-06 | -1.949898279 | -11.30593831 | 2.29E-05 |
| --- | --- | --- | --- | --- |
| MRPS6 | 1.26E-08 | -1.953906153 | -15.43831767 | 2.99E-07 |
| ZDHHC23 | 3.24E-06 | -1.960183933 | -10.75947276 | 4.21E-05 |
| KHDRBS3 | 4.70E-06 | -1.963826414 | -10.46350272 | 5.87E-05 |
| AC098850.3 | 4.09E-07 | -1.965042805 | -12.5533739 | 6.92E-06 |
| BICDL2 | 5.76E-10 | -1.965545977 | -18.16015743 | 1.82E-08 |
| HSPA1B | 5.12E-12 | -1.970377161 | -22.24659213 | 2.39E-10 |
| TUBB4B | 4.46E-10 | -1.980382187 | -18.51740385 | 1.45E-08 |
| HMGA1 | 2.17E-08 | -1.982433834 | -15.19147926 | 4.93E-07 |
| NET1 | 4.79E-05 | -1.983364065 | -8.568076516 | 0.000438119 |
| ODF2L | 2.50E-10 | -1.984446496 | -19.05414811 | 8.40E-09 |
| MET | 1.46E-06 | -1.98700646 | -11.59379536 | 2.12E-05 |
| LINC02018 | 3.91E-11 | -1.987573829 | -20.68694616 | 1.52E-09 |
| DST | 8.72E-18 | -1.988247417 | -33.91890776 | 1.04E-15 |
| AC138969.1 | 1.58E-09 | -1.992606299 | -17.53950817 | 4.52E-08 |
| TMEM67 | 5.73E-07 | -1.993541846 | -12.44348303 | 9.26E-06 |
| TTC26 | 1.50E-12 | -1.993788546 | -23.57262448 | 7.63E-11 |
| CCDC144B | 3.54E-15 | -1.997826676 | -28.87155052 | 2.80E-13 |
| MOK | 2.58E-07 | -1.998583217 | -13.16617861 | 4.61E-06 |
| AC114947.2 | 1.65E-10 | -1.999152844 | -19.55589754 | 5.72E-09 |
| MDK | 0.000360015 | -2.00020633 | -6.888069363 | 0.00246407 |
| FGF5 | 0.000590014 | -2.00454907 | -6.472964244 | 0.003733179 |
| EFCAB6 | 6.08E-06 | -2.005743103 | -10.46263991 | 7.38E-05 |
| TSPAN19 | 2.34E-06 | -2.010099259 | -11.31671595 | 3.20E-05 |
| CEP83 | 7.21E-15 | -2.010514459 | -28.43333234 | 5.44E-13 |
| NBEA | 2.28E-13 | -2.014088096 | -25.46370539 | 1.33E-11 |
| CPLANE1 | 4.84E-10 | -2.01681044 | -18.7861763 | 1.56E-08 |
| CDH1 | 0.000436815 | -2.018440358 | -6.781359543 | 0.002897644 |
| UGDH | 6.51E-08 | -2.025551893 | -14.5562359 | 1.33E-06 |
| KIAA1841 | 2.00E-11 | -2.027839938 | -21.69521213 | 8.36E-10 |
| CDCA7L | 1.12E-10 | -2.029499956 | -20.19233893 | 3.99E-09 |
| SLC2A3P4 | 6.04E-05 | -2.032925709 | -8.57676418 | 0.000535336 |
| ISYNA1 | 1.02E-06 | -2.033973016 | -12.18938737 | 1.55E-05 |
| ST6GALNAC1 | 8.31E-05 | -2.042653793 | -8.335131326 | 0.000701154 |
| DZIP1L | 1.58E-13 | -2.044050712 | -26.16774297 | 9.47E-12 |
| MAP1A | 6.41E-08 | -2.049773407 | -14.744621 | 1.31E-06 |
| MT-ATP8 | 2.04E-23 | -2.0501953 | -46.52036435 | 5.22E-21 |
| EFCAB12 | 1.31E-06 | -2.056818962 | -12.10067302 | 1.92E-05 |
| SLC1A1 | 2.76E-05 | -2.057568526 | -9.379424509 | 0.000274489 |
| AC005670.2 | 4.83E-06 | -2.057990535 | -10.93955936 | 6.03E-05 |
| BHLHE40-AS1 | 0.000249722 | -2.059902396 | -7.420885699 | 0.001801541 |
| ZNF440 | 2.86E-12 | -2.061997098 | -23.80232468 | 1.40E-10 |
| ANKRD39 | 3.22E-07 | -2.063334914 | -13.39616415 | 5.57E-06 |
| WHRN | 0.000385899 | -2.064446334 | -7.047041672 | 0.002615002 |
| SPATA7 | 9.17E-05 | -2.067381964 | -8.347401355 | 0.000763411 |
| GOLM1 | 3.43E-05 | -2.079251655 | -9.282178619 | 0.000329888 |
| HSPE1 | 9.26E-05 | -2.083516847 | -8.403593592 | 0.000769613 |
| KIF9 | 1.41E-10 | -2.086903278 | -20.55905027 | 4.91E-09 |
| AC006600.2 | 7.55E-07 | -2.0869788 | -12.77656939 | 1.19E-05 |
| NPIPA5 | 1.76E-12 | -2.093711951 | -24.61150692 | 8.80E-11 |
| HMGB3 | 8.67E-05 | -2.097950579 | -8.522296827 | 0.000726075 |
| TMC5 | 8.08E-12 | -2.099746872 | -23.29189355 | 3.64E-10 |

| RFX3 | 9.83E-12 | -2.104896557 | -23.16977275 | 4.31E-10 |
| --- | --- | --- | --- | --- |
| TTLL9 | 5.12E-07 | -2.110607828 | -13.27641692 | 8.42E-06 |
| CAB39L | 2.22E-06 | -2.114517106 | -11.95296308 | 3.05E-05 |
| ZNF473 | 1.84E-07 | -2.114890536 | -14.24188749 | 3.37E-06 |
| AL353743.4 | 5.16E-07 | -2.118109059 | -13.31720718 | 8.46E-06 |
| AC242842.2 | 0.001664499 | -2.11895063 | -5.887963243 | 0.008910048 |
| DYNC2H1 | 5.86E-15 | -2.122536837 | -30.20748437 | 4.48E-13 |
| ENKD1 | 0.000574844 | -2.123400789 | -6.880774263 | 0.003661078 |
| AC138625.1 | 1.25E-06 | -2.130058985 | -12.57680909 | 1.85E-05 |
| CCDC30 | 3.76E-15 | -2.130316906 | -30.72918438 | 2.96E-13 |
| NEK10 | 3.20E-11 | -2.134628517 | -22.40260497 | 1.26E-09 |
| HOOK1 | 3.60E-09 | -2.13480787 | -18.02523003 | 9.65E-08 |
| CAPS2 | 1.37E-07 | -2.138604487 | -14.67656396 | 2.59E-06 |
| WDR90 | 4.59E-06 | -2.139791242 | -11.42162032 | 5.75E-05 |
| NDFIP2 | 0.000530601 | -2.14083492 | -7.011730084 | 0.003427886 |
| HES1 | 5.53E-11 | -2.144328491 | -21.99415951 | 2.07E-09 |
| RIMS1 | 9.83E-07 | -2.144888839 | -12.8848732 | 1.50E-05 |
| ATP2C2 | 1.49E-10 | -2.145379674 | -21.08409967 | 5.18E-09 |
| SPACA9 | 2.09E-05 | -2.145659625 | -10.04166731 | 0.000215544 |
| RABL2B | 1.30E-06 | -2.147136406 | -12.64157185 | 1.91E-05 |
| PTPRF | 2.53E-08 | -2.147684094 | -16.31446884 | 5.67E-07 |
| SSBP4 | 6.48E-08 | -2.1481962 | -15.44202931 | 1.33E-06 |
| AQP4-AS1 | 2.69E-05 | -2.149925995 | -9.826818412 | 0.000267732 |
| EFHC2 | 7.72E-05 | -2.15078362 | -8.844825175 | 0.000659453 |
| TOGARAM2 | 1.51E-07 | -2.158326105 | -14.72237202 | 2.83E-06 |
| PER2 | 2.56E-12 | -2.160428986 | -25.04296727 | 1.26E-10 |
| AKR1C1 | 4.26E-13 | -2.16245623 | -26.75107623 | 2.36E-11 |
| DPCD | 7.23E-06 | -2.162777856 | -11.11895964 | 8.58E-05 |
| TGFB3 | 4.23E-07 | -2.171483018 | -13.83991681 | 7.12E-06 |
| C11orf65 | 4.50E-05 | -2.174223101 | -9.449953858 | 0.000415179 |
| CES4A | 0.00032545 | -2.183962775 | -7.616603837 | 0.002255141 |
| STK33 | 3.23E-10 | -2.185461689 | -20.74235032 | 1.07E-08 |
| CD74 | 2.11E-10 | -2.199225798 | -21.27774296 | 7.17E-09 |
| TAF1C | 8.13E-08 | -2.20432628 | -15.62839548 | 1.62E-06 |
| AC005037.2 | 2.11E-05 | -2.206286084 | -10.31611912 | 0.000217156 |
| PIH1D2 | 1.24E-05 | -2.20735567 | -10.83038236 | 0.000137245 |
| SPINK5 | 2.90E-09 | -2.21404577 | -18.90331346 | 7.89E-08 |
| NSUN7 | 2.30E-09 | -2.215685594 | -19.14002579 | 6.32E-08 |
| ATF3 | 2.36E-13 | -2.219483571 | -28.02717328 | 1.37E-11 |
| FP671120.4 | 8.32E-10 | -2.227494708 | -20.22531897 | 2.53E-08 |
| RNA5-8SN1 | 8.32E-10 | -2.227494708 | -20.22531897 | 2.53E-08 |
| RNA5-8SN2 | 8.32E-10 | -2.227494708 | -20.22531897 | 2.53E-08 |
| RNA5-8SN3 | 8.32E-10 | -2.227494708 | -20.22531897 | 2.53E-08 |
| CCDC191 | 1.91E-14 | -2.229440367 | -30.58614237 | 1.35E-12 |
| DPY19L2P2 | 8.60E-12 | -2.237335281 | -24.75711385 | 3.85E-10 |
| ENKUR | 5.82E-11 | -2.239442859 | -22.92072997 | 2.17E-09 |
| FAM166A | 5.20E-11 | -2.245249632 | -23.09003395 | 1.96E-09 |
| DRAIC | 5.32E-10 | -2.247856604 | -20.84600774 | 1.71E-08 |
| AC027117.1 | 0.000418704 | -2.248716207 | -7.596372176 | 0.002792118 |
| CROCC | 0.000362808 | -2.248920742 | -7.737014512 | 0.002480421 |
| MAP6 | 1.31E-07 | -2.250105557 | -15.48374989 | 2.50E-06 |
| NME5 | 3.02E-06 | -2.250210583 | -12.42114469 | 3.97E-05 |

| RASSF6 | 2.63E-14 | -2.256193795 | -30.63930141 | 1.80E-12 |
| --- | --- | --- | --- | --- |
| SIGIRR | 0.000512764 | -2.259948541 | -7.435417782 | 0.003332511 |
| CCDC81 | 5.05E-09 | -2.262360849 | -18.76926119 | 1.30E-07 |
| ABCA13 | 1.05E-14 | -2.268303921 | -31.70872801 | 7.77E-13 |
| DNAJC12 | 0.00187962 | -2.26997362 | -6.187788947 | 0.009875108 |
| CCDC180 | 1.39E-15 | -2.274150649 | -33.78941482 | 1.19E-13 |
| BEND5 | 1.62E-08 | -2.276294235 | -17.73063116 | 3.79E-07 |
| CSMD1 | 0.000541568 | -2.278783306 | -7.443297882 | 0.003476788 |
| BMS1P7 | 2.12E-06 | -2.279492414 | -12.93412907 | 2.92E-05 |
| NR4A2 | 0.000397732 | -2.281150673 | -7.756846183 | 0.002681381 |
| FBXL13 | 0.000141049 | -2.289243453 | -8.815031449 | 0.001095163 |
| SPA17 | 1.08E-08 | -2.290163934 | -18.24208372 | 2.62E-07 |
| DZANK1 | 2.55E-07 | -2.293244411 | -15.11884861 | 4.56E-06 |
| KIAA1522 | 3.58E-09 | -2.294070544 | -19.37487035 | 9.61E-08 |
| NLRC3 | 7.25E-07 | -2.298180537 | -14.10975588 | 1.15E-05 |
| SGMS2 | 3.35E-05 | -2.302823347 | -10.30566493 | 0.000322618 |
| FGFR2 | 5.45E-05 | -2.308240844 | -9.841751032 | 0.000489437 |
| SOX9 | 0.000486848 | -2.308414125 | -7.646867991 | 0.003178658 |
| SPAG16 | 2.44E-11 | -2.309066139 | -24.50489451 | 9.91E-10 |
| DNAAF4 | 6.92E-14 | -2.311612378 | -30.42109448 | 4.42E-12 |
| GFRA1 | 3.86E-08 | -2.313391843 | -17.14934908 | 8.32E-07 |
| ANKRD26 | 2.52E-15 | -2.314844056 | -33.79238378 | 2.05E-13 |
| ACTA2 | 1.38E-09 | -2.328083599 | -20.62561963 | 4.01E-08 |
| AL049697.1 | 1.95E-09 | -2.329451302 | -20.28994622 | 5.46E-08 |
| TM4SF1 | 1.14E-12 | -2.33335065 | -27.87132212 | 5.93E-11 |
| ARMC2 | 3.12E-11 | -2.339683325 | -24.58088106 | 1.23E-09 |
| FGFR3 | 1.44E-08 | -2.3410373 | -18.35772129 | 3.38E-07 |
| CASC2 | 1.78E-09 | -2.346058355 | -20.52850764 | 5.01E-08 |
| KDM4A | 6.19E-09 | -2.352407154 | -19.30847947 | 1.58E-07 |
| AC005339.1 | 8.11E-05 | -2.358821736 | -9.650399784 | 0.00068731 |
| ERBB4 | 0.001602791 | -2.360847153 | -6.598858729 | 0.008624829 |
| NRAV | 3.48E-06 | -2.363344462 | -12.89948965 | 4.49E-05 |
| KIF6 | 2.63E-10 | -2.363588979 | -22.64521403 | 8.78E-09 |
| ZFHX2 | 1.68E-09 | -2.367794194 | -20.7752047 | 4.78E-08 |
| FDXR | 3.10E-07 | -2.371057279 | -15.43328944 | 5.39E-06 |
| STC1 | 2.46E-19 | -2.372850684 | -44.15776733 | 3.51E-17 |
| PCDH7 | 2.56E-08 | -2.375670858 | -18.03721415 | 5.71E-07 |
| RGS5 | 2.78E-13 | -2.380475289 | -29.89062671 | 1.59E-11 |
| IRX3 | 6.00E-10 | -2.381832742 | -21.96536373 | 1.89E-08 |
| STK36 | 2.98E-06 | -2.385126644 | -13.18107841 | 3.93E-05 |
| MTND4P24 | 5.15E-07 | -2.393815432 | -15.05320847 | 8.45E-06 |
| MTCO1P53 | 1.20E-15 | -2.395444463 | -35.74401133 | 1.04E-13 |
| AC006059.2 | 1.58E-16 | -2.400175547 | -37.92838195 | 1.60E-14 |
| TRAF3IP1 | 1.65E-14 | -2.413543814 | -33.26321462 | 1.18E-12 |
| AL390879.1 | 7.12E-07 | -2.421989426 | -14.88859957 | 1.13E-05 |
| STMND1 | 2.41E-11 | -2.424296995 | -25.74290007 | 9.81E-10 |
| IFT81 | 2.91E-12 | -2.427061971 | -27.99731271 | 1.42E-10 |
| EVA1C | 0.000774732 | -2.429089553 | -7.556529862 | 0.004693993 |
| STRA6LP | 1.38E-18 | -2.438931759 | -43.55835241 | 1.84E-16 |
| GLB1L2 | 0.001042058 | -2.444850068 | -7.290806785 | 0.006013131 |
| KCNRG | 2.04E-06 | -2.449481744 | -13.938122 | 2.84E-05 |
| AL354733.2 | 1.47E-09 | -2.461083176 | -21.73916054 | 4.25E-08 |

| KCNK1 | 0.001686649 | -2.464331011 | -6.833529088 | 0.009012907 |
| --- | --- | --- | --- | --- |
| LRRC6 | 6.56E-14 | -2.466143075 | -32.51149741 | 4.26E-12 |
| MIR1244-1 | 0.000177098 | -2.47609703 | -9.289787468 | 0.001338404 |
| FAM227A | 1.06E-13 | -2.48996014 | -32.30865382 | 6.54E-12 |
| SPAG8 | 0.000100782 | -2.490136488 | -9.952120306 | 0.000824744 |
| ULK4 | 2.00E-12 | -2.491801202 | -29.15318994 | 9.92E-11 |
| LRP11 | 3.40E-12 | -2.49717444 | -28.63948056 | 1.64E-10 |
| FAM229B | 1.48E-09 | -2.499336747 | -22.06524356 | 4.27E-08 |
| MIR125B1 | 3.95E-06 | -2.505110965 | -13.53697141 | 5.03E-05 |
| DNALI1 | 3.15E-11 | -2.518956582 | -26.45454205 | 1.24E-09 |
| H1-0 | 1.59E-12 | -2.519763487 | -29.72923165 | 8.01E-11 |
| RGS22 | 1.35E-10 | -2.520833385 | -24.88269914 | 4.71E-09 |
| AC139256.3 | 1.86E-13 | -2.52276251 | -32.1153336 | 1.10E-11 |
| ECRG4 | 0.000274975 | -2.526445363 | -8.995932102 | 0.001959169 |
| TSPAN6 | 6.33E-06 | -2.526499357 | -13.13430521 | 7.64E-05 |
| ABHD11-AS1 | 0.000335809 | -2.528603014 | -8.784134805 | 0.002316463 |
| MZF1-AS1 | 2.41E-06 | -2.533766281 | -14.2361091 | 3.26E-05 |
| NPIPP1 | 1.59E-13 | -2.533820678 | -32.42596971 | 9.54E-12 |
| ELF3 | 2.04E-16 | -2.535643645 | -39.78667053 | 2.00E-14 |
| LMNTD1 | 3.07E-09 | -2.542947619 | -21.64715524 | 8.32E-08 |
| CLDN1 | 2.03E-07 | -2.560376209 | -17.13538241 | 3.67E-06 |
| MT1E | 1.61E-10 | -2.560521314 | -25.07403969 | 5.59E-09 |
| IQUB | 7.64E-16 | -2.562628881 | -38.73922779 | 6.87E-14 |
| KRT15 | 5.08E-14 | -2.564213234 | -34.0895755 | 3.33E-12 |
| AC112128.1 | 5.88E-13 | -2.567565577 | -31.4031236 | 3.20E-11 |
| RTKN2 | 7.32E-13 | -2.573331469 | -31.22937607 | 3.92E-11 |
| AC017002.4 | 1.09E-15 | -2.574096123 | -38.51666936 | 9.55E-14 |
| AC013470.2 | 6.05E-09 | -2.577642587 | -21.1844772 | 1.54E-07 |
| CCDC181 | 3.54E-13 | -2.585099856 | -32.18648282 | 2.00E-11 |
| CFAP70 | 4.69E-15 | -2.589037805 | -37.09830238 | 3.63E-13 |
| AL451062.4 | 0.000865254 | -2.591828374 | -7.938398607 | 0.005142677 |
| WFDC2 | 1.92E-13 | -2.594131183 | -32.9899776 | 1.13E-11 |
| CFAP74 | 4.22E-17 | -2.594434433 | -42.4819812 | 4.60E-15 |
| NPHP1 | 7.93E-14 | -2.594490499 | -33.98925672 | 5.02E-12 |
| KLF5 | 9.75E-10 | -2.594806596 | -23.38171867 | 2.93E-08 |
| WDR66 | 1.96E-20 | -2.59652349 | -51.16926016 | 3.32E-18 |
| KIF27 | 4.14E-16 | -2.597561745 | -39.95865515 | 3.87E-14 |
| IQCA1 | 6.10E-18 | -2.602158912 | -44.7960265 | 7.44E-16 |
| BBC3 | 1.47E-11 | -2.610057444 | -28.27705871 | 6.30E-10 |
| AC069503.2 | 1.21E-19 | -2.613270761 | -49.43343098 | 1.80E-17 |
| AC007000.4 | 3.10E-12 | -2.623465936 | -30.19410909 | 1.50E-10 |
| E2F3P2 | 8.80E-06 | -2.626092395 | -13.27616193 | 0.000101983 |
| TRIM47 | 4.20E-09 | -2.627837375 | -22.01288264 | 1.10E-07 |
| TTC21A | 1.94E-09 | -2.628985489 | -22.90268727 | 5.45E-08 |
| CFAP44 | 3.40E-21 | -2.636496267 | -53.96542702 | 6.39E-19 |
| LCA5L | 2.51E-06 | -2.638928041 | -14.77660634 | 3.39E-05 |
| LCA5 | 2.66E-11 | -2.648878229 | -28.01011124 | 1.07E-09 |
| IL1RL1 | 4.74E-16 | -2.65036395 | -40.61387418 | 4.37E-14 |
| NR4A3 | 1.16E-10 | -2.6567018 | -26.39957558 | 4.09E-09 |
| KIF21A | 3.79E-17 | -2.660985998 | -43.6973526 | 4.16E-15 |
| CCDC65 | 6.80E-14 | -2.664816536 | -35.08897554 | 4.38E-12 |
| BMPR1B | 5.63E-05 | -2.665367975 | -11.32577959 | 0.000503608 |

| MIR7705 | 1.79E-15 | -2.668381473 | -39.35141422 | 1.51E-13 |
| --- | --- | --- | --- | --- |
| DMD | 1.24E-20 | -2.683326995 | -53.41798121 | 2.17E-18 |
| GOLGA2P5 | 3.68E-16 | -2.684369987 | -41.43017282 | 3.50E-14 |
| N6AMT1 | 6.75E-06 | -2.692139798 | -13.92071125 | 8.08E-05 |
| CCDC114 | 4.11E-10 | -2.702411644 | -25.3657221 | 1.34E-08 |
| CX3CL1 | 8.44E-06 | -2.702845331 | -13.71341806 | 9.82E-05 |
| CCDC13 | 2.02E-18 | -2.704258833 | -47.84864495 | 2.66E-16 |
| CDS1 | 1.36E-12 | -2.714843021 | -32.21236605 | 7.00E-11 |
| CCDC146 | 8.58E-20 | -2.724824713 | -51.95245425 | 1.30E-17 |
| MTATP8P2 | 2.19E-16 | -2.72491556 | -42.66873583 | 2.14E-14 |
| ACTA2-AS1 | 4.52E-11 | -2.739647487 | -28.34123463 | 1.73E-09 |
| CFAP157 | 1.53E-12 | -2.746944509 | -32.45438395 | 7.73E-11 |
| TMC4 | 2.23E-09 | -2.752359955 | -23.81128347 | 6.16E-08 |
| AC012321.1 | 0.000177939 | -2.755868842 | -10.333763 | 0.001343105 |
| GOLGA8CP | 1.86E-08 | -2.758889962 | -21.32940324 | 4.28E-07 |
| ECT2L | 3.19E-21 | -2.776868506 | -56.91636682 | 6.12E-19 |
| LRRC23 | 4.44E-16 | -2.777853584 | -42.64613428 | 4.11E-14 |
| COL12A1 | 8.90E-07 | -2.792221871 | -16.89526098 | 1.38E-05 |
| LRRC37A4P | 2.22E-18 | -2.793342359 | -49.31331145 | 2.90E-16 |
| CFAP45 | 8.50E-17 | -2.795157885 | -44.91935888 | 8.85E-15 |
| RPL9P28 | 0.000231944 | -2.796946478 | -10.16582842 | 0.001691833 |
| PPP1R14C | 4.13E-05 | -2.80146767 | -12.28208211 | 0.000386173 |
| DCDC1 | 3.93E-20 | -2.809562309 | -54.52184851 | 6.08E-18 |
| ABHD11 | 2.52E-06 | -2.813195264 | -15.75100381 | 3.39E-05 |
| SPEF2 | 3.14E-22 | -2.816123657 | -60.55445638 | 6.98E-20 |
| DUSP2 | 1.10E-05 | -2.81827179 | -13.97392844 | 0.000124267 |
| NEK11 | 2.16E-17 | -2.857170936 | -47.61412007 | 2.45E-15 |
| DNAH7 | 3.36E-27 | -2.857672069 | -75.65339063 | 1.47E-24 |
| FOXC1 | 1.24E-09 | -2.866815245 | -25.53112143 | 3.66E-08 |
| AGBL2 | 1.54E-15 | -2.872416176 | -42.54461204 | 1.31E-13 |
| ANKUB1 | 1.70E-10 | -2.872720937 | -28.06700965 | 5.85E-09 |
| MNS1 | 3.76E-21 | -2.879890759 | -58.82056983 | 7.00E-19 |
| TPPP3 | 1.24E-19 | -2.887463927 | -54.58737257 | 1.82E-17 |
| CFAP58 | 5.65E-15 | -2.889448981 | -41.16826723 | 4.34E-13 |
| DNAH11 | 5.30E-25 | -2.918188923 | -70.84059411 | 1.68E-22 |
| CETN2 | 1.08E-14 | -2.924064918 | -40.84312043 | 7.93E-13 |
| CD24 | 9.47E-21 | -2.931076614 | -58.69099522 | 1.71E-18 |
| ARMC4 | 3.63E-16 | -2.931652692 | -45.26577402 | 3.46E-14 |
| FSIP1 | 2.78E-08 | -2.949685156 | -22.28792237 | 6.15E-07 |
| NWD1 | 3.31E-22 | -2.960991276 | -63.60145824 | 7.27E-20 |
| CASC1 | 1.82E-22 | -2.961274075 | -64.37457725 | 4.26E-20 |
| IQCH | 7.72E-10 | -2.962959271 | -26.99923864 | 2.37E-08 |
| WDR78 | 1.66E-17 | -2.977668114 | -49.96577576 | 1.91E-15 |
| NHLRC4 | 4.22E-06 | -2.979768182 | -16.0151349 | 5.34E-05 |
| CCDC40 | 2.44E-16 | -2.986720645 | -46.63243806 | 2.36E-14 |
| RABL2A | 6.75E-10 | -2.994312634 | -27.4598556 | 2.10E-08 |
| RYR3 | 1.98E-14 | -3.00764455 | -41.21176784 | 1.39E-12 |
| DUSP5 | 4.23E-13 | -3.019399252 | -37.36064921 | 2.36E-11 |
| FHAD1 | 4.13E-26 | -3.020162365 | -76.66370926 | 1.59E-23 |
| SAMD15 | 1.17E-12 | -3.040551052 | -36.28346081 | 6.07E-11 |
| CFAP54 | 4.62E-22 | -3.057943966 | -65.24249633 | 9.78E-20 |
| AC105052.5 | 1.39E-16 | -3.058992925 | -48.50373133 | 1.43E-14 |

| PTPRT | 1.68E-15 | -3.062579602 | -45.25238307 | 1.42E-13 |
| --- | --- | --- | --- | --- |
| AKAP14 | 4.49E-07 | -3.064518145 | -19.45405664 | 7.48E-06 |
| KIAA1211L | 2.51E-15 | -3.071867169 | -44.84842752 | 2.05E-13 |
| TNFRSF21 | 2.86E-05 | -3.123641852 | -14.19472699 | 0.000282125 |
| CLHC1 | 2.59E-08 | -3.126175816 | -23.71951178 | 5.76E-07 |
| GIPR | 2.27E-06 | -3.130855264 | -17.67090164 | 3.11E-05 |
| AC004494.1 | 4.01E-07 | -3.133303587 | -20.0426351 | 6.80E-06 |
| HYDIN2 | 4.35E-26 | -3.142905411 | -79.70777074 | 1.60E-23 |
| AC069444.2 | 2.82E-20 | -3.150496882 | -61.58975259 | 4.60E-18 |
| DNAH5 | 1.51E-24 | -3.15465583 | -75.15085142 | 4.55E-22 |
| MAATS1 | 2.44E-21 | -3.196139101 | -65.88092043 | 4.73E-19 |
| RSPH10B | 1.86E-20 | -3.200611325 | -63.14710315 | 3.18E-18 |
| AC090607.4 | 1.18E-16 | -3.202906226 | -51.01651856 | 1.21E-14 |
| ANKRD18A | 1.15E-22 | -3.212012989 | -70.46769518 | 2.79E-20 |
| SPATA17 | 1.69E-20 | -3.229307844 | -63.84662136 | 2.92E-18 |
| HYDIN | 6.24E-27 | -3.229314218 | -84.62438493 | 2.67E-24 |
| OSCP1 | 1.31E-07 | -3.237949408 | -22.28360911 | 2.50E-06 |
| BCAS1 | 7.53E-17 | -3.24365557 | -52.29846916 | 7.88E-15 |
| RSPH4A | 9.42E-14 | -3.250242305 | -42.33788418 | 5.84E-12 |
| CCDC175 | 8.52E-16 | -3.259593791 | -49.12139469 | 7.54E-14 |
| CP | 2.77E-16 | -3.285515837 | -51.11694379 | 2.65E-14 |
| CHST9 | 4.52E-17 | -3.320072248 | -54.26501148 | 4.85E-15 |
| AC018761.4 | 4.40E-07 | -3.331884714 | -21.17971575 | 7.36E-06 |
| FBXW9 | 4.40E-07 | -3.331884714 | -21.17971575 | 7.36E-06 |
| DNAH3 | 1.91E-24 | -3.338465389 | -79.18295093 | 5.51E-22 |
| TSGA10 | 3.15E-19 | -3.361718782 | -62.19951214 | 4.33E-17 |
| NEK5 | 3.45E-22 | -3.392090352 | -72.80211137 | 7.48E-20 |
| WDR63 | 1.67E-23 | -3.392701398 | -77.27448389 | 4.40E-21 |
| DNAH6 | 1.73E-22 | -3.418454521 | -74.39326347 | 4.08E-20 |
| CCDC39 | 2.25E-20 | -3.421874872 | -67.23409435 | 3.73E-18 |
| BBOF1 | 4.61E-23 | -3.427315507 | -76.55358217 | 1.15E-20 |
| CFAP43 | 2.88E-29 | -3.452994658 | -98.55226358 | 1.51E-26 |
| PRSS12 | 5.97E-16 | -3.466650152 | -52.77560668 | 5.42E-14 |
| C12orf75 | 2.83E-07 | -3.469911837 | -22.71993975 | 5.01E-06 |
| AC092802.2 | 3.78E-14 | -3.47216059 | -46.60584842 | 2.53E-12 |
| MED15P9 | 3.00E-31 | -3.476239927 | -106.1064441 | 2.05E-28 |
| FBXW10 | 3.92E-09 | -3.483439172 | -29.28592105 | 1.04E-07 |
| TEX9 | 2.93E-13 | -3.50063386 | -43.87592792 | 1.66E-11 |
| PIFO | 1.34E-12 | -3.514017736 | -41.72551571 | 6.92E-11 |
| TNNI3 | 2.36E-17 | -3.515558242 | -58.45465306 | 2.63E-15 |
| FYB2 | 3.91E-26 | -3.516548505 | -89.3475009 | 1.53E-23 |
| RAB36 | 1.98E-05 | -3.537462812 | -16.64014182 | 0.000205458 |
| CFAP53 | 3.83E-22 | -3.540781499 | -75.8304129 | 8.22E-20 |
| CCDC173 | 4.69E-24 | -3.564322684 | -83.15143915 | 1.25E-21 |
| SPAG17 | 1.82E-33 | -3.577752539 | -117.1377869 | 1.97E-30 |
| AC004224.2 | 3.26E-19 | -3.612066942 | -66.77536261 | 4.45E-17 |
| AL357093.2 | 4.32E-12 | -3.622772256 | -41.17224501 | 2.05E-10 |
| MYCBPAP | 3.35E-14 | -3.64673557 | -49.14190516 | 2.26E-12 |
| EFHB | 1.12E-09 | -3.669136291 | -32.84453865 | 3.32E-08 |
| PTPN20CP | 3.26E-07 | -3.674116738 | -23.83288616 | 5.64E-06 |
| TTC25 | 2.93E-13 | -3.702016762 | -46.39535044 | 1.66E-11 |
| LRRIQ1 | 1.53E-35 | -3.716936075 | -129.4017165 | 2.02E-32 |

| AK7 | 4.62E-11 | -3.731428081 | -38.56563983 | 1.76E-09 |
| --- | --- | --- | --- | --- |
| RSPH10B2 | 2.53E-22 | -3.74655505 | -80.91705427 | 5.74E-20 |
| VWA3A | 5.29E-30 | -3.762564699 | -110.1552228 | 3.05E-27 |
| CDHR3 | 2.75E-24 | -3.762897625 | -88.65444128 | 7.57E-22 |
| CNN1 | 1.23E-14 | -3.803256207 | -52.90539745 | 9.02E-13 |
| TTC16 | 2.68E-17 | -3.804541665 | -63.05038463 | 2.95E-15 |
| AC008670.1 | 9.54E-08 | -3.824563954 | -26.85045196 | 1.87E-06 |
| NR4A1 | 8.93E-25 | -3.87141059 | -93.10475499 | 2.74E-22 |
| CCDC148 | 1.95E-18 | -3.917315269 | -69.37759909 | 2.58E-16 |
| CCDC60 | 4.68E-15 | -4.03165214 | -57.77372814 | 3.63E-13 |
| DNAAF1 | 7.74E-32 | -4.062239688 | -126.3810444 | 5.94E-29 |
| CCDC170 | 3.41E-20 | -4.132597501 | -80.45019983 | 5.37E-18 |
| KIAA2012 | 7.33E-33 | -4.14990982 | -133.3580178 | 6.75E-30 |
| TMEM232 | 6.01E-22 | -4.150298173 | -88.07326205 | 1.24E-19 |
| AC025647.2 | 1.34E-13 | -4.170692636 | -53.69263336 | 8.16E-12 |
| CFAP46 | 3.48E-32 | -4.231745462 | -133.1236158 | 3.05E-29 |
| GNAZ | 1.02E-05 | -4.260465701 | -21.26056511 | 0.000116154 |
| DNAH12 | 7.02E-36 | -4.287781176 | -150.7299655 | 1.04E-32 |
| DNAH10 | 1.41E-31 | -4.299241365 | -132.635075 | 1.04E-28 |
| FANK1 | 1.89E-23 | -4.306923468 | -97.86976191 | 4.90E-21 |
| DRC3 | 1.62E-24 | -4.334509663 | -103.1246405 | 4.80E-22 |
| CCDC74B | 7.34E-36 | -4.373054351 | -153.6432991 | 1.04E-32 |
| CAPS | 4.59E-32 | -4.48157734 | -140.4444423 | 3.84E-29 |
| ANKRD18B | 1.60E-31 | -4.492225632 | -138.3395057 | 1.14E-28 |
| ERICH3 | 1.99E-36 | -4.537247974 | -161.9866035 | 3.66E-33 |
| CCDC74A | 2.79E-36 | -4.641704544 | -165.03519 | 4.67E-33 |
| ANKRD18CP | 6.02E-30 | -4.765227621 | -139.2420872 | 3.36E-27 |
| SOX2 | 6.69E-27 | -6.298995058 | -164.8723361 | 2.80E-24 |

DEGs HEART

| gene_ID | metap | metafc | idx | fdr |
| --- | --- | --- | --- | --- |
| IL1RL1 | 4.35E-95 | 4.448777885 | 419.7954243 | 8.01E-91 |
| SLCO4A1 | 2.28E-59 | 4.074629659 | 238.9415397 | 1.40E-55 |
| ADAMTS9 | 1.84E-60 | 3.476489322 | 207.6670015 | 1.70E-56 |
| CBS | 3.82E-20 | 3.090764678 | 60.01557619 | 1.72E-17 |
| CBSL | 1.24E-19 | 3.090764678 | 58.4308656 | 4.99E-17 |
| TNFSF14 | 6.10E-13 | 2.960841215 | 36.16603103 | 8.03E-11 |
| PNP | 1.06E-17 | 2.908093808 | 49.3582212 | 2.93E-15 |
| RND1 | 3.08E-06 | 2.846311624 | 15.68534466 | 0.000104931 |
| BDKRB2 | 0.000237369 | 2.655037794 | 9.623383938 | 0.004120663 |
| AL355075.3 | 2.52E-16 | 2.607198046 | 40.66679909 | 5.29E-14 |
| AL731577.1 | 3.71E-05 | 2.565123233 | 11.36514951 | 0.00089288 |
| AC022211.4 | 2.67E-05 | 2.49220655 | 11.39823043 | 0.000679796 |
| NNMT | 6.63E-29 | 2.466815863 | 69.5115533 | 6.79E-26 |
| LERFS | 3.12E-06 | 2.406334468 | 13.24870487 | 0.000105951 |
| MT1G | 0.000257433 | 2.328109258 | 8.356367631 | 0.004398542 |
| TYMP | 3.97E-26 | 2.302615141 | 58.4904542 | 2.71E-23 |
| ASRGL1 | 1.79E-23 | 2.218521302 | 50.46330813 | 1.00E-20 |
| CD163 | 9.32E-36 | 2.206400142 | 77.2913558 | 2.15E-32 |
| AL022170.1 | 9.92E-07 | 2.187725765 | 13.13421234 | 3.89E-05 |
| P3H2 | 1.63E-05 | 2.137583875 | 10.23609292 | 0.000454766 |
| AD000671.3 | 3.42E-22 | 2.137007775 | 45.87334204 | 1.70E-19 |
| CD86 | 3.95E-05 | 2.135144807 | 9.401366512 | 0.000937765 |
| SLC7A5 | 6.77E-08 | 2.114896522 | 15.16286661 | 3.52E-06 |
| MT1M | 2.35E-14 | 2.111673131 | 28.77804278 | 3.84E-12 |
| UCK2 | 4.49E-05 | 2.1052276 | 9.152745945 | 0.001036316 |
| MT1F | 0.000357029 | 2.096527382 | 7.227351922 | 0.005669409 |
| SH3GL2 | 0.000167616 | 2.078878245 | 7.849187792 | 0.003087084 |
| SNHG15 | 8.32E-06 | 2.058522723 | 10.45683813 | 0.000252755 |
| ACKR1 | 7.61E-33 | 2.044869631 | 65.67833675 | 1.17E-29 |
| IL4R | 3.11E-18 | 2.039358078 | 35.70468746 | 9.54E-16 |
| CHI3L2 | 4.78E-13 | 1.927632588 | 23.74988819 | 6.34E-11 |
| CACHD1 | 0.000710298 | 1.923648491 | 6.056722074 | 0.009973378 |
| SLC7A8 | 1.88E-12 | 1.918750302 | 22.49987553 | 2.34E-10 |
| NFKB2 | 3.32E-17 | 1.910139896 | 31.47575655 | 8.76E-15 |
| AP003064.1 | 1.90E-05 | 1.882488014 | 8.886691121 | 0.000519574 |
| PSENEN | 6.98E-18 | 1.870360629 | 32.08865506 | 1.98E-15 |
| BCL3 | 7.28E-19 | 1.858149093 | 33.70314212 | 2.63E-16 |
| ISG20 | 3.64E-07 | 1.852282554 | 11.92724681 | 1.62E-05 |
| SERPINE1 | 6.27E-29 | 1.824926289 | 51.46758686 | 6.79E-26 |
| STC1 | 1.06E-35 | 1.811900497 | 63.36823862 | 2.18E-32 |
| ECT2L | 1.06E-06 | 1.791195868 | 10.70503342 | 4.10E-05 |
| SBNO2 | 4.88E-10 | 1.780883051 | 16.5824521 | 3.98E-08 |
| OSMR | 2.72E-11 | 1.771412199 | 18.71561541 | 2.83E-09 |
| ETS2 | 6.78E-15 | 1.732935564 | 24.55362059 | 1.19E-12 |
| MT2A | 4.33E-17 | 1.73007026 | 28.31082772 | 1.09E-14 |
| DLGAP1-AS2 | 0.000217422 | 1.698316582 | 6.220417006 | 0.003829119 |
| IRX2 | 5.61E-11 | 1.696105441 | 17.3863332 | 5.53E-09 |
| P3R3URF-PIK3R3 | 6.28E-17 | 1.692671076 | 27.42476614 | 1.50E-14 |

| VCAM1 | 5.93E-15 | 1.682850108 | 23.94207188 | 1.06E-12 |
| --- | --- | --- | --- | --- |
| YBX3 | 8.11E-20 | 1.668102521 | 31.84612905 | 3.56E-17 |
| HELB | 2.37E-05 | 1.661727407 | 7.685726255 | 0.000617292 |
| SOCS2 | 4.14E-08 | 1.661592023 | 12.26734217 | 2.30E-06 |
| GFPT2 | 5.14E-08 | 1.658382678 | 12.08757101 | 2.73E-06 |
| APBB1IP | 0.000303075 | 1.656804191 | 5.829382899 | 0.004975501 |
| MT1X | 2.17E-16 | 1.621011769 | 25.39228003 | 4.70E-14 |
| MLLT11 | 4.50E-08 | 1.600851043 | 11.76142422 | 2.45E-06 |
| FES | 1.25E-06 | 1.588895282 | 9.37812988 | 4.74E-05 |
| PVT1 | 0.000244669 | 1.578492842 | 5.700603227 | 0.004211682 |
| TLR2 | 2.54E-06 | 1.570357888 | 8.78663395 | 8.82E-05 |
| S100A8 | 2.53E-18 | 1.557394002 | 27.40617122 | 7.90E-16 |
| AL049839.2 | 2.55E-20 | 1.551807598 | 30.40607193 | 1.17E-17 |
| GALNT15 | 2.02E-18 | 1.546306903 | 27.36300483 | 6.41E-16 |
| AC002398.2 | 4.57E-27 | 1.545881793 | 40.71804098 | 3.51E-24 |
| RPGR | 3.01E-09 | 1.545339231 | 13.16957997 | 2.08E-07 |
| HSPB6 | 6.44E-27 | 1.542708328 | 40.40541585 | 4.75E-24 |
| PLSCR1 | 1.52E-15 | 1.532358869 | 22.70580486 | 2.95E-13 |
| PAPPA | 3.36E-07 | 1.514628005 | 9.805108835 | 1.53E-05 |
| CDKN1A | 9.03E-19 | 1.514240398 | 27.32315626 | 3.14E-16 |
| IL1B | 2.38E-06 | 1.501997456 | 8.44639689 | 8.36E-05 |
| TNFAIP2 | 1.06E-15 | 1.496283993 | 22.4082131 | 2.07E-13 |
| UGCG | 1.11E-22 | 1.495583263 | 32.83248419 | 5.87E-20 |
| SLC2A14 | 1.50E-06 | 1.492735265 | 8.692126118 | 5.52E-05 |
| HS6ST3 | 1.37E-07 | 1.475828107 | 10.12931609 | 6.70E-06 |
| ADAMTS9-AS1 | 4.06E-33 | 1.472376641 | 47.69196238 | 7.17E-30 |
| RSPH10B2 | 0.000167352 | 1.470457542 | 5.552990709 | 0.0030853 |
| RPS2P4 | 0.000295992 | 1.4657397 | 5.172185112 | 0.004894088 |
| SERPINA3 | 9.79E-20 | 1.464974721 | 27.84772344 | 4.20E-17 |
| CU638689.4 | 0.000674503 | 1.460448282 | 4.631105378 | 0.009595008 |
| USB1 | 3.73E-05 | 1.449116939 | 6.416398258 | 0.000896107 |
| CEBPD | 6.02E-17 | 1.438836973 | 23.33846649 | 1.46E-14 |
| EVI2A | 4.63E-05 | 1.41819341 | 6.147680269 | 0.001056637 |
| DLGAP1-AS1 | 7.06E-08 | 1.380042011 | 9.868747672 | 3.67E-06 |
| FAM110D | 3.55E-05 | 1.379879067 | 6.139838421 | 0.000860466 |
| PAPPA-AS1 | 5.46E-05 | 1.379397336 | 5.880272019 | 0.001209525 |
| LINC02552 | 1.10E-07 | 1.371653382 | 9.543501679 | 5.51E-06 |
| F2RL3 | 3.32E-08 | 1.370854109 | 10.25239533 | 1.86E-06 |
| SCN3B | 1.10E-12 | 1.353561874 | 16.18639877 | 1.42E-10 |
| CPM | 7.58E-08 | 1.351498944 | 9.623334074 | 3.91E-06 |
| TRIM14 | 0.000273842 | 1.350953831 | 4.812772764 | 0.004614765 |
| MCTP1 | 2.80E-12 | 1.350777449 | 15.60599487 | 3.28E-10 |
| IL18R1 | 3.94E-10 | 1.347004935 | 12.66803517 | 3.30E-08 |
| FCMR | 4.73E-05 | 1.334967897 | 5.774233445 | 0.001074666 |
| SMAD6 | 7.59E-09 | 1.332433007 | 10.81941512 | 4.87E-07 |
| SDF2L1 | 2.10E-05 | 1.327572599 | 6.21077226 | 0.000564526 |
| PIK3R3 | 6.50E-15 | 1.325615296 | 18.80682476 | 1.15E-12 |
| ADIPOR1 | 6.49E-05 | 1.312697119 | 5.497584018 | 0.001387244 |
| SOCS3 | 2.62E-27 | 1.306110928 | 34.71800343 | 2.20E-24 |
| SPRY1 | 3.82E-12 | 1.298871724 | 14.83066422 | 4.40E-10 |
| APOLD1 | 1.02E-10 | 1.293349551 | 12.92293931 | 9.68E-09 |
| GTF2IRD1 | 5.83E-07 | 1.292937752 | 8.061063643 | 2.46E-05 |

| AC010976.2 | 5.00E-07 | 1.291546221 | 8.137981794 | 2.16E-05 |
| --- | --- | --- | --- | --- |
| IGFBP4 | 2.13E-08 | 1.290602353 | 9.90099559 | 1.25E-06 |
| FOSL2 | 4.54E-10 | 1.288659109 | 12.0401314 | 3.73E-08 |
| CTSC | 5.00E-08 | 1.282846296 | 9.365904741 | 2.67E-06 |
| TNFRSF6B | 0.000406188 | 1.280267665 | 4.341737179 | 0.006356944 |
| AC005363.1 | 2.59E-10 | 1.274334151 | 12.21743208 | 2.25E-08 |
| MCTP2 | 1.93E-08 | 1.271082849 | 9.805242379 | 1.15E-06 |
| C1QB | 1.22E-19 | 1.269609599 | 24.01399718 | 4.99E-17 |
| ELF3 | 4.28E-33 | 1.265288374 | 40.95577408 | 7.17E-30 |
| SAMSN1 | 5.22E-05 | 1.265086434 | 5.418006963 | 0.00116687 |
| AC139256.2 | 2.48E-10 | 1.263925085 | 12.14003208 | 2.19E-08 |
| NAMPT | 4.15E-17 | 1.254159352 | 20.54573704 | 1.06E-14 |
| CLEC2B | 1.85E-07 | 1.253017708 | 8.435195563 | 8.90E-06 |
| IL1R1 | 1.88E-08 | 1.251325374 | 9.667507214 | 1.12E-06 |
| EVA1C | 1.96E-08 | 1.249678122 | 9.631558245 | 1.16E-06 |
| PTPN20CP | 1.20E-05 | 1.247405038 | 6.137298293 | 0.000348463 |
| ERAP2 | 3.11E-07 | 1.245765316 | 8.106212709 | 1.43E-05 |
| NCF2 | 0.000264354 | 1.238168751 | 4.429937681 | 0.004497388 |
| S1PR1 | 0.000257737 | 1.237541006 | 4.441316604 | 0.00439966 |
| FMN1 | 8.01E-09 | 1.225215856 | 9.920018756 | 5.09E-07 |
| SNAP47 | 2.22E-09 | 1.224804528 | 10.59917171 | 1.60E-07 |
| SLC11A1 | 3.61E-13 | 1.221707133 | 15.20100419 | 4.89E-11 |
| RPS12P26 | 3.85E-11 | 1.21892618 | 12.69439653 | 3.84E-09 |
| ITGA5 | 2.34E-05 | 1.217844117 | 5.640187444 | 0.000610342 |
| IQCJ-SCHIP1 | 5.81E-05 | 1.211676667 | 5.132402731 | 0.001264729 |
| PCNT | 2.15E-11 | 1.197752204 | 12.77803038 | 2.26E-09 |
| PTGIR | 4.61E-08 | 1.193379662 | 8.754848422 | 2.49E-06 |
| CFB | 2.97E-09 | 1.192254438 | 10.16748928 | 2.06E-07 |
| ABCB1 | 3.49E-06 | 1.189911205 | 6.493074142 | 0.000117521 |
| CP | 1.16E-11 | 1.187229916 | 12.98507763 | 1.25E-09 |
| AP000781.1 | 1.87E-05 | 1.184358554 | 5.598766594 | 0.000512567 |
| AL645922.1 | 8.19E-10 | 1.17423932 | 10.66971142 | 6.40E-08 |
| AL035078.4 | 0.000278241 | 1.166764059 | 4.14852124 | 0.004671817 |
| IL4I1 | 0.000525893 | 1.149037457 | 3.767811793 | 0.007757813 |
| AC016876.2 | 1.69E-05 | 1.146293829 | 5.469225562 | 0.00047019 |
| RDH10 | 9.31E-08 | 1.140950025 | 8.022102675 | 4.74E-06 |
| PSTPIP2 | 0.000182306 | 1.138203535 | 4.255969967 | 0.003308062 |
| UBE2L6 | 1.10E-08 | 1.137121608 | 9.048482016 | 6.87E-07 |
| HP | 6.61E-10 | 1.136771054 | 10.43500656 | 5.23E-08 |
| TEX26-AS1 | 5.71E-12 | 1.13658184 | 12.77905487 | 6.46E-10 |
| C1R | 2.41E-14 | 1.126116623 | 15.33466492 | 3.90E-12 |
| IFI16 | 4.73E-07 | 1.125824928 | 7.120883253 | 2.06E-05 |
| GLUL | 8.83E-08 | 1.124318269 | 7.930896221 | 4.52E-06 |
| ITPRIP | 5.79E-06 | 1.122120743 | 5.876976338 | 0.000180897 |
| LDHA | 4.72E-08 | 1.120998707 | 8.21199735 | 2.54E-06 |
| SCARA5 | 3.51E-05 | 1.118115171 | 4.981063937 | 0.000853482 |
| C1QTNF1 | 7.14E-21 | 1.109870014 | 22.35961036 | 3.38E-18 |
| TAL1 | 7.96E-05 | 1.108814126 | 4.54521994 | 0.001650414 |
| SLA | 3.53E-08 | 1.104799956 | 8.233279099 | 1.97E-06 |
| HPR | 1.91E-10 | 1.103762471 | 10.72632714 | 1.76E-08 |
| APOD | 1.26E-18 | 1.091052717 | 19.5293706 | 4.30E-16 |
| PELI1 | 2.53E-07 | 1.088909604 | 7.183034913 | 1.19E-05 |

| POR | 1.23E-06 | 1.073265535 | 6.344255764 | 4.66E-05 |
| --- | --- | --- | --- | --- |
| FOLR2 | 5.00E-05 | 1.072971509 | 4.614978028 | 0.001128499 |
| PTGER3 | 4.31E-05 | 1.071623395 | 4.678528495 | 0.001007299 |
| LINC01554 | 0.000467355 | 1.071216521 | 3.567529263 | 0.007114911 |
| CES1 | 0.000523835 | 1.069423171 | 3.508569335 | 0.007744526 |
| JMJD4 | 0.000444092 | 1.066138974 | 3.574259556 | 0.006845555 |
| DNAJA1 | 9.92E-10 | 1.065350004 | 9.59206238 | 7.62E-08 |
| CPNE8 | 2.90E-05 | 1.063591618 | 4.82610613 | 0.00072551 |
| CYP1B1 | 1.78E-13 | 1.059626383 | 13.5087682 | 2.55E-11 |
| F13A1 | 4.49E-13 | 1.057042075 | 13.05159615 | 6.05E-11 |
| PLEKHG1 | 3.45E-07 | 1.051284216 | 6.794118501 | 1.56E-05 |
| PLIN2 | 2.45E-16 | 1.046980873 | 16.34420795 | 5.19E-14 |
| DCUN1D3 | 0.000111846 | 1.038389461 | 4.103069951 | 0.002182007 |
| GIMAP8 | 2.16E-14 | 1.033864514 | 14.12881153 | 3.55E-12 |
| SGK1 | 3.59E-07 | 1.028139564 | 6.626533732 | 1.61E-05 |
| ABHD3 | 0.000270296 | 1.026858018 | 3.663993701 | 0.004575924 |
| SLC16A3 | 4.21E-05 | 1.016649719 | 4.44902413 | 0.000988982 |
| PROS1 | 4.65E-13 | 1.014545021 | 12.51150168 | 6.22E-11 |
| MTND6P3 | 1.72E-18 | -1.001843063 | -17.79816219 | 5.75E-16 |
| MT-TE | 3.40E-17 | -1.001893771 | -16.4996601 | 8.83E-15 |
| MT-ND4 | 7.54E-12 | -1.005426782 | -11.18303454 | 8.37E-10 |
| RBM6 | 5.61E-06 | -1.009825894 | -5.302356591 | 0.000176305 |
| CHST6 | 4.99E-05 | -1.010290541 | -4.346556425 | 0.001127783 |
| MTND6P22 | 2.52E-12 | -1.021333591 | -11.84661002 | 2.99E-10 |
| LINC01031 | 3.48E-05 | -1.021944043 | -4.556521019 | 0.000848136 |
| AC016355.1 | 4.33E-07 | -1.022197707 | -6.504367689 | 1.90E-05 |
| ARHGAP22 | 0.00061804 | -1.022670749 | -3.281733234 | 0.008868346 |
| AC090616.6 | 7.98E-05 | -1.023761839 | -4.195435333 | 0.001652794 |
| TNFRSF13C | 0.000182001 | -1.025424794 | -3.835012811 | 0.003305786 |
| MTCO2P22 | 2.16E-16 | -1.026824028 | -16.08503182 | 4.70E-14 |
| AC093525.10 | 0.000108891 | -1.032823607 | -4.093088919 | 0.002128856 |
| CAVIN1 | 0.000210356 | -1.034177937 | -3.802718043 | 0.00372539 |
| SH3RF2 | 0.000440938 | -1.035687426 | -3.475375802 | 0.006814045 |
| MAPRE3-AS1 | 8.71E-06 | -1.041263986 | -5.268887431 | 0.000263178 |
| AC103988.1 | 1.19E-07 | -1.04326052 | -7.224488924 | 5.92E-06 |
| INS-IGF2 | 2.81E-09 | -1.047065131 | -8.953336878 | 1.96E-07 |
| AC051619.3 | 0.000282136 | -1.048271625 | -3.720883311 | 0.004730705 |
| CADPS | 1.78E-05 | -1.051845565 | -4.996138365 | 0.000490188 |
| SYCP2 | 4.44E-05 | -1.051852825 | -4.578734648 | 0.001027797 |
| MTCO1P12 | 1.81E-10 | -1.060117477 | -10.32769739 | 1.68E-08 |
| AC093752.1 | 0.000231883 | -1.060815921 | -3.855781577 | 0.004048282 |
| IGSF3 | 0.000152132 | -1.061632786 | -4.053079978 | 0.002835899 |
| KLF2 | 3.89E-07 | -1.063429123 | -6.816500435 | 1.72E-05 |
| PKP2 | 0.000287695 | -1.064375194 | -3.769023855 | 0.004788931 |
| KCNJ3 | 0.000440991 | -1.065347762 | -3.574849055 | 0.006814045 |
| FYB2 | 7.23E-05 | -1.065655787 | -4.412983426 | 0.001522517 |
| AC132825.2 | 1.20E-17 | -1.070022171 | -18.10650554 | 3.25E-15 |
| ABCB4 | 0.000465108 | -1.074464401 | -3.580594677 | 0.007092418 |
| TGFB2 | 1.80E-06 | -1.079974176 | -6.203553667 | 6.53E-05 |
| MT-ND3 | 1.59E-16 | -1.080823865 | -17.07480724 | 3.62E-14 |
| TAGLN | 4.33E-08 | -1.081755463 | -7.965406964 | 2.37E-06 |
| ARHGEF1 | 6.36E-05 | -1.086075833 | -4.557574633 | 0.001365541 |

| AC016907.2 | 3.89E-05 | -1.087013596 | -4.793943552 | 0.000926252 |
| --- | --- | --- | --- | --- |
| MTRNR2L12 | 1.55E-05 | -1.087392305 | -5.230048001 | 0.000434896 |
| MTRNR2L10 | 2.75E-11 | -1.087832667 | -11.48898121 | 2.84E-09 |
| AC135050.1 | 9.94E-06 | -1.092006565 | -5.463069343 | 0.000296282 |
| PTDSS1 | 0.000627641 | -1.093835759 | -3.502778198 | 0.008997809 |
| ANKRD36C | 5.41E-06 | -1.094544652 | -5.765167722 | 0.000171517 |
| ANKRD20A17P | 9.63E-06 | -1.095214523 | -5.493948611 | 0.000288247 |
| CRY2 | 8.35E-05 | -1.095595959 | -4.468068186 | 0.001718505 |
| UPF3AP2 | 6.96E-05 | -1.097473218 | -4.562688171 | 0.001476382 |
| PPARGC1B | 0.000100339 | -1.09790144 | -4.389993706 | 0.001993185 |
| MT-CYB | 1.06E-16 | -1.103201475 | -17.62270467 | 2.45E-14 |
| MKI67 | 2.14E-05 | -1.103418867 | -5.153194484 | 0.000572626 |
| EGR3 | 0.000312333 | -1.108039334 | -3.884101156 | 0.005086725 |
| AL353729.1 | 4.41E-05 | -1.109827339 | -4.833476038 | 0.00102491 |
| AC073840.1 | 9.57E-05 | -1.111899925 | -4.468910924 | 0.001917398 |
| MT-TF | 5.38E-18 | -1.116551613 | -19.28180128 | 1.55E-15 |
| ANKRD36 | 2.68E-05 | -1.11690349 | -5.105781644 | 0.000682046 |
| PCSK7 | 2.21E-08 | -1.119238331 | -8.568016258 | 1.29E-06 |
| AC245297.1 | 6.39E-07 | -1.124634017 | -6.966850374 | 2.67E-05 |
| MT-TW | 3.89E-10 | -1.127391567 | -10.60910119 | 3.27E-08 |
| PTMS | 1.36E-07 | -1.129519735 | -7.754468032 | 6.69E-06 |
| ORAI2 | 0.000264437 | -1.132211295 | -4.050686396 | 0.004497388 |
| SNORD13 | 0.000572305 | -1.144099475 | -3.709596932 | 0.00833413 |
| HEYL | 2.10E-05 | -1.145869704 | -5.359506075 | 0.000565072 |
| RMND5A | 5.52E-06 | -1.146342285 | -6.02728683 | 0.000174048 |
| FAM133CP | 6.18E-09 | -1.149962616 | -9.439925461 | 4.00E-07 |
| GOLGA8O | 0.000532658 | -1.151262864 | -3.768718635 | 0.007837252 |
| MTCO3P12 | 3.28E-15 | -1.15453501 | -16.72175996 | 6.18E-13 |
| DYRK2 | 1.92E-05 | -1.155355049 | -5.449871938 | 0.000522425 |
| LRRC37A4P | 0.00019633 | -1.158469196 | -4.294460827 | 0.003527816 |
| PCDH17 | 9.49E-06 | -1.162724518 | -5.839913051 | 0.000284564 |
| ANKRD49P2 | 0.000671357 | -1.165512352 | -3.698224884 | 0.009557635 |
| AC114498.2 | 3.92E-11 | -1.166483825 | -12.1397726 | 3.88E-09 |
| AC138776.1 | 9.90E-06 | -1.170949722 | -5.859647738 | 0.000295936 |
| SPARC | 0.000525996 | -1.172250563 | -3.843829991 | 0.007757813 |
| AL049835.1 | 2.68E-06 | -1.173674831 | -6.538849961 | 9.27E-05 |
| PYGM | 2.94E-06 | -1.173933129 | -6.493371211 | 0.000100643 |
| RNU6-758P | 5.38E-06 | -1.177405468 | -6.203794049 | 0.000171081 |
| TET1 | 0.000609679 | -1.178560914 | -3.788953734 | 0.008777047 |
| MT-ATP6 | 3.38E-15 | -1.184229076 | -17.13634396 | 6.30E-13 |
| ANKRD36B | 2.98E-07 | -1.18621506 | -7.741498103 | 1.38E-05 |
| CHD3 | 2.06E-06 | -1.187024887 | -6.749001267 | 7.37E-05 |
| TIAM1 | 0.000122172 | -1.187270319 | -4.645823874 | 0.00235052 |
| ASPN | 5.29E-08 | -1.19003254 | -8.659389918 | 2.79E-06 |
| AC007952.2 | 9.68E-13 | -1.192623901 | -14.32821298 | 1.27E-10 |
| FAM153B | 3.24E-08 | -1.194504442 | -8.946521283 | 1.82E-06 |
| CSF2RA | 0.000603234 | -1.195918872 | -3.850277627 | 0.00869525 |
| PPP1R3C | 4.42E-07 | -1.199936781 | -7.624744003 | 1.94E-05 |
| SLFN12 | 1.07E-05 | -1.208873379 | -6.007397942 | 0.000315486 |
| EGR1 | 2.32E-10 | -1.209068293 | -11.64959269 | 2.10E-08 |
| MIR133A1HG | 4.09E-09 | -1.209350558 | -10.14383859 | 2.70E-07 |
| ADAMTSL3 | 4.14E-06 | -1.214795804 | -6.539332882 | 0.000136272 |

| GOLGA8Q | 0.000139307 | -1.218795305 | -4.699708349 | 0.002620674 |
| --- | --- | --- | --- | --- |
| Z97200.1 | 1.84E-07 | -1.222351453 | -8.233036139 | 8.85E-06 |
| MASP1 | 1.29E-07 | -1.223860083 | -8.430361145 | 6.39E-06 |
| NR4A1 | 5.26E-14 | -1.224815031 | -16.26435795 | 8.08E-12 |
| AL137143.1 | 0.000600614 | -1.225862668 | -3.948999919 | 0.008664251 |
| CR381670.1 | 2.96E-06 | -1.226905979 | -6.783260548 | 0.000101046 |
| CYP4V2 | 0.000156992 | -1.228186059 | -4.672169373 | 0.002914712 |
| LRMDA | 0.000271198 | -1.233271215 | -4.398724928 | 0.004585892 |
| BRF2 | 8.91E-05 | -1.233533655 | -4.995697127 | 0.001813974 |
| PLCB2 | 2.03E-07 | -1.235126784 | -8.267203113 | 9.70E-06 |
| NR2F2-AS1 | 6.12E-06 | -1.239947155 | -6.463849619 | 0.000190374 |
| AL390783.1 | 0.000283085 | -1.24058486 | -4.401697422 | 0.004730705 |
| AC023818.1 | 1.61E-07 | -1.240843984 | -8.430775647 | 7.79E-06 |
| MT-TG | 1.31E-19 | -1.241323638 | -23.4414904 | 5.12E-17 |
| CNTNAP3 | 1.81E-08 | -1.24174534 | -9.613222612 | 1.08E-06 |
| MLF1 | 6.92E-05 | -1.243974995 | -5.175176721 | 0.001468769 |
| IGFBP5 | 3.39E-07 | -1.247465148 | -8.07015578 | 1.54E-05 |
| FUT1 | 1.01E-06 | -1.247943009 | -7.480047223 | 3.96E-05 |
| HCG11 | 1.72E-08 | -1.248314974 | -9.692919485 | 1.03E-06 |
| SNHG5 | 2.43E-10 | -1.264743989 | -12.1601723 | 2.16E-08 |
| AC006254.1 | 3.31E-05 | -1.267728531 | -5.680407126 | 0.000810351 |
| ATP1A3 | 0.00031933 | -1.273892737 | -4.453223086 | 0.005177813 |
| BTN3A2 | 2.49E-07 | -1.275930533 | -8.425051773 | 1.17E-05 |
| COL3A1 | 3.26E-09 | -1.278794991 | -10.85261847 | 2.24E-07 |
| HSPB8 | 9.95E-06 | -1.279340332 | -6.399606475 | 0.000296282 |
| AQP1 | 1.91E-05 | -1.283932058 | -6.060199249 | 0.000519624 |
| LINC02843 | 0.000353343 | -1.288081946 | -4.446205064 | 0.005620569 |
| AL356585.4 | 2.13E-05 | -1.294016627 | -6.045493328 | 0.000571252 |
| MYO3B | 3.71E-06 | -1.297666643 | -7.046571434 | 0.000124034 |
| FAM118A | 0.000127376 | -1.297849473 | -5.055011537 | 0.002425927 |
| TBX2 | 0.000451732 | -1.298284786 | -4.34291681 | 0.006928568 |
| NPIPB2 | 3.56E-09 | -1.308186315 | -11.05264724 | 2.42E-07 |
| ANKRD49P1 | 1.36E-08 | -1.309211522 | -10.2981173 | 8.31E-07 |
| AMOTL2 | 2.15E-06 | -1.310080446 | -7.425748309 | 7.64E-05 |
| MT-TL2 | 2.65E-09 | -1.311662055 | -11.24961567 | 1.87E-07 |
| LRRC39 | 0.000127619 | -1.313509899 | -5.114916546 | 0.002428063 |
| AC011446.2 | 1.06E-09 | -1.318523402 | -11.83537383 | 8.05E-08 |
| COL1A2 | 5.18E-06 | -1.321176567 | -6.983197692 | 0.00016562 |
| SYNPO2L | 2.33E-12 | -1.322251924 | -15.38201484 | 2.82E-10 |
| GCOM1 | 0.000143034 | -1.32613147 | -5.098393407 | 0.00267985 |
| LARP4P | 1.69E-05 | -1.336286032 | -6.376749997 | 0.000470065 |
| MMP16 | 0.000471776 | -1.336714957 | -4.44626765 | 0.007158562 |
| SULF1 | 0.000106865 | -1.338731908 | -5.316325486 | 0.002100384 |
| AC114801.3 | 6.98E-19 | -1.342854405 | -24.38120649 | 2.57E-16 |
| ATF3 | 3.70E-10 | -1.345855979 | -12.69314141 | 3.15E-08 |
| FSD2 | 5.31E-08 | -1.34637763 | -9.79470233 | 2.80E-06 |
| BRSK1 | 1.53E-12 | -1.349577048 | -15.94562203 | 1.92E-10 |
| FOS | 3.94E-18 | -1.350900077 | -23.51218025 | 1.19E-15 |
| PLEKHH2 | 0.000114727 | -1.354233353 | -5.33613446 | 0.002233473 |
| B3GALT2 | 1.94E-05 | -1.357219398 | -6.396208861 | 0.000526098 |
| DIO2 | 5.74E-06 | -1.35789575 | -7.116470309 | 0.000180037 |
| AF001548.2 | 0.000507645 | -1.362363367 | -4.488224441 | 0.007558579 |

| TNFAIP8L1 | 6.17E-06 | -1.370213125 | -7.138593369 | 0.000191443 |
| --- | --- | --- | --- | --- |
| MT-TM | 9.73E-10 | -1.373912095 | -12.38142125 | 7.51E-08 |
| AC010998.2 | 0.000266916 | -1.376836158 | -4.920296362 | 0.004531184 |
| MUSTN1 | 0.000108209 | -1.378150644 | -5.465381124 | 0.002122283 |
| PDE4C | 0.000323377 | -1.384171816 | -4.831161436 | 0.005234229 |
| ADGRA2 | 5.68E-05 | -1.38443995 | -5.877954896 | 0.001247892 |
| MECOM-AS1 | 1.39E-06 | -1.384955398 | -8.111958849 | 5.15E-05 |
| GREB1L | 2.78E-05 | -1.393280679 | -6.347903729 | 0.000699958 |
| GJA1 | 5.37E-06 | -1.398720203 | -7.371015578 | 0.000171064 |
| TUSC8 | 3.35E-06 | -1.402392083 | -7.678801502 | 0.000112766 |
| AC005154.2 | 5.77E-05 | -1.406663084 | -5.962843734 | 0.001259868 |
| FAM215B | 4.77E-06 | -1.406824517 | -7.486478328 | 0.000153719 |
| AC136424.2 | 9.32E-05 | -1.411089487 | -5.687649571 | 0.001879494 |
| RN7SL608P | 0.000253826 | -1.415316455 | -5.088718932 | 0.004340945 |
| MT-CO3 | 4.22E-14 | -1.415437425 | -18.93074608 | 6.60E-12 |
| OGN | 1.74E-09 | -1.428594397 | -12.51224595 | 1.29E-07 |
| MRC2 | 0.000499363 | -1.432829126 | -4.73060489 | 0.007472614 |
| CASC15 | 3.62E-07 | -1.447832818 | -9.326547571 | 1.61E-05 |
| MEG3 | 4.28E-09 | -1.458912227 | -12.20833341 | 2.81E-07 |
| GOLGA8DP | 0.000149297 | -1.459733798 | -5.584865516 | 0.002788699 |
| MT-TV | 1.20E-25 | -1.464450908 | -36.49487177 | 7.91E-23 |
| AC245297.2 | 0.000250428 | -1.466774634 | -5.282321453 | 0.004294776 |
| PROM1 | 0.000411073 | -1.469889386 | -4.977163919 | 0.006417062 |
| LINC02388 | 7.00E-07 | -1.477846718 | -9.095705526 | 2.88E-05 |
| AC011466.4 | 1.08E-06 | -1.478858547 | -8.823071162 | 4.19E-05 |
| AC108215.1 | 2.25E-05 | -1.484497886 | -6.900062397 | 0.000594779 |
| AP000224.1 | 0.000310938 | -1.484868293 | -5.207917053 | 0.005073776 |
| LINC00632 | 1.83E-05 | -1.492973568 | -7.071581237 | 0.000503179 |
| PTN | 1.36E-05 | -1.494913694 | -7.274598108 | 0.00038833 |
| CCN2 | 1.33E-05 | -1.496640401 | -7.300170209 | 0.000379971 |
| CARMN | 5.51E-07 | -1.496942804 | -9.369196745 | 2.35E-05 |
| BEX1 | 4.40E-06 | -1.497505083 | -8.021618454 | 0.00014303 |
| MTRNR2L6 | 3.21E-08 | -1.497795737 | -11.22361987 | 1.81E-06 |
| SEPTIN11 | 8.89E-06 | -1.504378625 | -7.598468107 | 0.000268263 |
| MT-TC | 5.72E-19 | -1.510690275 | -27.55922252 | 2.15E-16 |
| SYT2 | 8.64E-07 | -1.517612833 | -9.201859682 | 3.45E-05 |
| ANK1 | 2.86E-11 | -1.53488903 | -16.18440524 | 2.89E-09 |
| AC025627.3 | 6.75E-06 | -1.537261533 | -7.948466337 | 0.000207481 |
| AJ239322.1 | 9.33E-06 | -1.537431883 | -7.733323015 | 0.000280202 |
| TOGARAM2 | 0.000209208 | -1.541020622 | -5.670064631 | 0.003712184 |
| C5orf63 | 2.86E-11 | -1.548545455 | -16.32843558 | 2.89E-09 |
| OLFML2A | 4.05E-05 | -1.552748037 | -6.819710657 | 0.000958397 |
| MYZAP | 1.77E-06 | -1.557369623 | -8.957472681 | 6.44E-05 |
| MT-TY | 9.63E-23 | -1.563925264 | -34.43174026 | 5.22E-20 |
| AL022323.3 | 0.000120812 | -1.56847911 | -6.145128089 | 0.002329803 |
| SPN | 1.28E-06 | -1.571454235 | -9.258754717 | 4.83E-05 |
| FNDC5 | 3.69E-09 | -1.572813919 | -13.26386477 | 2.50E-07 |
| HOPX | 2.93E-07 | -1.587096083 | -10.36830032 | 1.36E-05 |
| SERTAD4 | 1.29E-06 | -1.589827482 | -9.364281195 | 4.83E-05 |
| DRAXIN | 1.13E-06 | -1.590104132 | -9.455898218 | 4.34E-05 |
| KCNN3 | 4.72E-07 | -1.591037518 | -10.06510911 | 2.06E-05 |
| NANOGP8 | 0.000115135 | -1.594861943 | -6.281827838 | 0.002238807 |

| CHGB | 6.81E-05 | -1.60234441 | -6.676777289 | 0.001449668 |
| --- | --- | --- | --- | --- |
| MUC20 | 2.53E-07 | -1.605348805 | -10.58910898 | 1.19E-05 |
| PTGDS | 3.51E-07 | -1.621850707 | -10.46874601 | 1.58E-05 |
| NFXL1 | 4.86E-06 | -1.629455955 | -8.657514969 | 0.000156452 |
| AC105339.2 | 4.39E-05 | -1.638631347 | -7.139731403 | 0.001021557 |
| FN3K | 4.31E-05 | -1.645509078 | -7.183138072 | 0.001007299 |
| MYH7B | 1.02E-09 | -1.645813015 | -14.79729246 | 7.81E-08 |
| DZIP1L | 2.21E-09 | -1.648481302 | -14.2693399 | 1.60E-07 |
| MTND3P25 | 1.63E-10 | -1.648708602 | -16.1364793 | 1.52E-08 |
| HLF | 1.52E-05 | -1.651413143 | -7.959129156 | 0.000426421 |
| DUSP1 | 4.50E-10 | -1.658939742 | -15.50650919 | 3.72E-08 |
| RN7SL275P | 9.91E-05 | -1.664775261 | -6.665989806 | 0.001972049 |
| LFNG | 0.000499187 | -1.669094772 | -5.510911355 | 0.007472614 |
| CHST1 | 5.15E-08 | -1.669346757 | -12.16582527 | 2.73E-06 |
| PRMT9 | 0.000115365 | -1.672552225 | -6.586387518 | 0.002238807 |
| REEP1 | 7.76E-05 | -1.674030742 | -6.880284532 | 0.001613363 |
| AL024508.1 | 2.36E-12 | -1.677839083 | -19.50799127 | 2.85E-10 |
| AC008397.2 | 3.67E-05 | -1.678258367 | -7.444222374 | 0.000886052 |
| MT-TS1 | 1.03E-19 | -1.679764689 | -31.89461981 | 4.31E-17 |
| UNC5B | 5.09E-10 | -1.684897545 | -15.65758149 | 4.14E-08 |
| DNAJB1P1 | 4.65E-10 | -1.690266503 | -15.77459121 | 3.81E-08 |
| AC092683.1 | 7.25E-15 | -1.69584515 | -23.97824091 | 1.26E-12 |
| ZSWIM4 | 2.66E-06 | -1.697488831 | -9.464559392 | 9.19E-05 |
| CCDC102B | 1.17E-08 | -1.697785432 | -13.46348641 | 7.27E-07 |
| GPR22 | 9.29E-10 | -1.703293714 | -15.38440185 | 7.22E-08 |
| MTCO1P40 | 7.48E-17 | -1.705076634 | -27.49633087 | 1.77E-14 |
| MT-TP | 5.06E-19 | -1.705358423 | -31.20106583 | 1.94E-16 |
| SLC35F1 | 1.19E-05 | -1.715347336 | -8.448864953 | 0.000344698 |
| FP671120.4 | 8.95E-07 | -1.720889916 | -10.40854458 | 3.53E-05 |
| RNA5-8SN1 | 8.95E-07 | -1.720889916 | -10.40854458 | 3.53E-05 |
| RNA5-8SN2 | 8.95E-07 | -1.720889916 | -10.40854458 | 3.53E-05 |
| RNA5-8SN3 | 8.95E-07 | -1.720889916 | -10.40854458 | 3.53E-05 |
| GPR20 | 0.000324983 | -1.721678909 | -6.005456744 | 0.005255597 |
| GOLGA8CP | 1.89E-06 | -1.721838735 | -9.855383893 | 6.79E-05 |
| AC092979.1 | 0.000213776 | -1.742771982 | -6.396043939 | 0.003778695 |
| ELP5 | 0.000305471 | -1.77865505 | -6.25202635 | 0.00500592 |
| FGF12 | 6.46E-10 | -1.779353451 | -16.35187902 | 5.16E-08 |
| AC117453.1 | 0.000129814 | -1.779489685 | -6.916305544 | 0.002464724 |
| RNF165 | 0.000159375 | -1.783814344 | -6.774177256 | 0.002953003 |
| C17orf97 | 1.28E-06 | -1.792804041 | -10.56387185 | 4.83E-05 |
| ACE | 0.000122786 | -1.80223491 | -7.048269906 | 0.002355557 |
| P2RY1 | 0.000177859 | -1.802319353 | -6.758561285 | 0.00324333 |
| NRXN3 | 0.000133533 | -1.804769431 | -6.992416577 | 0.002524946 |
| AC004943.2 | 1.54E-15 | -1.886098182 | -27.93982281 | 2.95E-13 |
| RPS14P4 | 1.70E-17 | -1.908746954 | -32.0087536 | 4.54E-15 |
| NREP | 7.11E-07 | -1.909323826 | -11.73854381 | 2.91E-05 |
| KCNJ2 | 3.36E-10 | -1.926318114 | -18.25021647 | 2.86E-08 |
| NEB | 3.77E-09 | -1.934557493 | -16.29706979 | 2.53E-07 |
| AL390719.3 | 0.000264733 | -1.937418552 | -6.93051704 | 0.00449827 |
| SLIT3 | 8.16E-06 | -1.938634475 | -9.864161403 | 0.00024872 |
| AC009690.3 | 0.00026795 | -1.941219383 | -6.933929775 | 0.004543137 |
| PLXDC1 | 1.15E-09 | -1.960265103 | -17.52546911 | 8.63E-08 |

| COL5A3 | 2.74E-05 | -1.969719217 | -8.985638205 | 0.000693508 |
| --- | --- | --- | --- | --- |
| GOLGA8K | 1.83E-06 | -1.983260588 | -11.38000683 | 6.60E-05 |
| LRRN3 | 1.95E-06 | -2.024786338 | -11.56081984 | 7.00E-05 |
| AC104794.3 | 1.50E-05 | -2.035515389 | -9.819432044 | 0.000423352 |
| TMEM178B | 1.14E-10 | -2.052402348 | -20.40563839 | 1.07E-08 |
| AC159540.2 | 2.28E-16 | -2.057525708 | -32.18300664 | 4.89E-14 |
| LRRC17 | 5.66E-10 | -2.06571918 | -19.10256953 | 4.55E-08 |
| GRIN2A | 2.27E-08 | -2.074814007 | -15.86105481 | 1.32E-06 |
| NTM | 2.83E-11 | -2.093818202 | -22.08620582 | 2.89E-09 |
| DMBX1 | 6.05E-07 | -2.108284139 | -13.10987532 | 2.55E-05 |
| MTND2P9 | 1.76E-22 | -2.13041245 | -46.34784583 | 9.00E-20 |
| AL031283.1 | 2.82E-14 | -2.136148521 | -28.94478572 | 4.52E-12 |
| PRAG1 | 0.000515874 | -2.138251534 | -7.029408041 | 0.007645224 |
| AC087457.1 | 2.35E-24 | -2.181924724 | -51.55746688 | 1.40E-21 |
| INHBA | 4.41E-10 | -2.18429647 | -20.4358214 | 3.66E-08 |
| CXCL14 | 5.36E-07 | -2.188627896 | -13.72396417 | 2.30E-05 |
| MTCO1P2 | 7.40E-07 | -2.205201394 | -13.51933623 | 3.01E-05 |
| SNORA61 | 2.00E-10 | -2.209775182 | -21.43178072 | 1.83E-08 |
| SOX11 | 3.40E-11 | -2.224706662 | -23.28972683 | 3.42E-09 |
| FSCN1 | 8.22E-11 | -2.23671637 | -22.5574828 | 7.98E-09 |
| MXRA5 | 8.18E-09 | -2.26522144 | -18.31948467 | 5.18E-07 |
| SOX4 | 4.61E-16 | -2.316950886 | -35.53346171 | 9.24E-14 |
| STARD4-AS1 | 6.58E-07 | -2.332767421 | -14.42052655 | 2.74E-05 |
| F5 | 2.31E-12 | -2.351983138 | -27.36840341 | 2.82E-10 |
| EPHB1 | 0.000426692 | -2.358309488 | -7.947232284 | 0.00662721 |
| LINC01151 | 1.14E-05 | -2.361704384 | -11.67793006 | 0.000331843 |
| PMEPA1 | 1.18E-13 | -2.378069268 | -30.74323151 | 1.74E-11 |
| AC135983.2 | 9.56E-10 | -2.386000238 | -21.52017415 | 7.41E-08 |
| AEBP1 | 3.80E-10 | -2.461653542 | -23.18876312 | 3.22E-08 |
| ENC1 | 3.25E-10 | -2.487703904 | -23.60394482 | 2.79E-08 |
| POSTN | 9.73E-28 | -2.509531615 | -67.78761787 | 8.54E-25 |
| ELN | 1.95E-13 | -2.520735103 | -32.04121775 | 2.74E-11 |
| ZNF618 | 1.08E-08 | -2.600533062 | -20.71990103 | 6.76E-07 |
| AL591543.1 | 2.39E-10 | -2.767195039 | -26.62704389 | 2.14E-08 |
| PIEZO2 | 6.90E-07 | -3.020632028 | -18.60984246 | 2.84E-05 |
| FOSB | 1.69E-25 | -3.261796457 | -80.80167653 | 1.07E-22 |
| MTCO1P53 | 8.13E-25 | -3.523127763 | -84.87244338 | 4.99E-22 |
| NR4A2 | 3.83E-22 | -3.714204294 | -79.54526975 | 1.86E-19 |
| MTND4P24 | 1.05E-40 | -3.75412614 | -150.0874556 | 2.76E-37 |
| COL1A1 | 5.83E-43 | -3.823467812 | -161.4817752 | 2.15E-39 |
| AP003396.5 | 1.29E-28 | -3.83080679 | -106.8421809 | 1.25E-25 |
| THY1 | 4.41E-31 | -4.002446482 | -121.4970225 | 5.81E-28 |
| RGS4 | 2.13E-31 | -4.452618292 | -136.5722968 | 3.02E-28 |
| AC008670.1 | 7.86E-41 | -4.5314861 | -181.7333548 | 2.42E-37 |
| AC008434.1 | 1.39E-50 | -4.594339487 | -229.0657925 | 6.39E-47 |

DEGs HIPPOCAMPUS

| gene_ID | metap | metafc | idx | fdr |
| --- | --- | --- | --- | --- |
| ANKRD22 | 6.03E-12 | 3.734330136 | 41.8977582 | 1.33E-09 |
| S100A9 | 5.61E-53 | 3.35690755 | 175.400921 | 5.18E-49 |
| SOCS3 | 4.67E-17 | 3.123152265 | 51.00237079 | 1.92E-14 |
| TOP2A | 4.65E-21 | 3.05634793 | 62.1420001 | 3.18E-18 |
| MKI67 | 4.28E-10 | 2.837363456 | 26.58298057 | 7.62E-08 |
| STC1 | 1.31E-22 | 2.777211092 | 60.77521556 | 1.15E-19 |
| S100A8 | 4.46E-39 | 2.634901881 | 101.0510314 | 1.37E-35 |
| AL139280.2 | 0.00016248 | 2.592127585 | 9.822089422 | 0.007634911 |
| ZFP36 | 4.53E-12 | 2.576642815 | 29.23034046 | 1.04E-09 |
| PCAT1 | 2.64E-07 | 2.479569057 | 16.30993436 | 2.77E-05 |
| IL1B | 2.84E-12 | 2.473621102 | 28.56259974 | 6.64E-10 |
| BCL2A1 | 1.52E-10 | 2.452057579 | 24.07207104 | 2.90E-08 |
| AC215522.2 | 5.65E-13 | 2.4133708 | 29.55954772 | 1.43E-10 |
| SCIN | 8.81E-33 | 2.396373809 | 76.81630399 | 1.63E-29 |
| TDRD12 | 2.76E-05 | 2.327544712 | 10.61237064 | 0.001621797 |
| IL1RL1 | 3.51E-09 | 2.312942996 | 19.5550315 | 5.31E-07 |
| SECTM1 | 2.12E-06 | 2.286673185 | 12.97553253 | 0.000169017 |
| HAVCR2 | 4.10E-15 | 2.181943855 | 31.39140321 | 1.33E-12 |
| AL390860.1 | 6.99E-07 | 2.092392542 | 12.87965501 | 6.49E-05 |
| IL4R | 2.45E-16 | 2.076194399 | 32.41117817 | 9.05E-14 |
| MPZL2 | 0.000141328 | 2.045549335 | 7.874896232 | 0.00679664 |
| CCDC114 | 1.99E-05 | 2.03222796 | 9.553533913 | 0.001221279 |
| NFKB2 | 2.37E-13 | 1.970534152 | 24.8775351 | 6.35E-11 |
| FCGBP | 3.40E-16 | 1.958711809 | 30.29954941 | 1.23E-13 |
| IL18 | 6.31E-09 | 1.949454622 | 15.98514226 | 9.18E-07 |
| C1QB | 8.10E-21 | 1.915192637 | 38.47894974 | 5.16E-18 |
| LERFS | 1.13E-06 | 1.864701812 | 11.08635835 | 0.000100201 |
| PLAC4 | 3.77E-06 | 1.854127882 | 10.05565079 | 0.000276446 |
| CASP4 | 1.42E-08 | 1.839277876 | 14.43431285 | 1.85E-06 |
| AC022144.1 | 3.22E-06 | 1.786636661 | 9.812661312 | 0.000241662 |
| ERAP2 | 2.33E-06 | 1.766396021 | 9.949935644 | 0.000183772 |
| CHI3L2 | 2.35E-58 | 1.75422415 | 101.0954695 | 4.33E-54 |
| CYTH4 | 6.28E-07 | 1.75056162 | 10.85712056 | 5.86E-05 |
| CD93 | 5.17E-12 | 1.739889913 | 19.63673404 | 1.17E-09 |
| HCLS1 | 2.68E-07 | 1.716526789 | 11.27960503 | 2.78E-05 |
| DNAJB1P1 | 2.26E-14 | 1.698256398 | 23.17583788 | 6.83E-12 |
| HILPDA | 7.86E-17 | 1.694407628 | 27.28789565 | 3.02E-14 |
| PIK3AP1 | 2.22E-08 | 1.688179529 | 12.91972876 | 2.74E-06 |
| LINC01554 | 1.45E-07 | 1.67885956 | 11.48190814 | 1.57E-05 |
| LAIR1 | 1.00E-15 | 1.650957095 | 24.76267182 | 3.49E-13 |
| C5AR1 | 4.29E-10 | 1.637658855 | 15.34069208 | 7.62E-08 |
| ADAMTS9 | 1.04E-14 | 1.593267751 | 22.27876844 | 3.20E-12 |
| NLRC5 | 4.35E-08 | 1.583069601 | 11.65364357 | 5.15E-06 |
| C1QA | 4.33E-19 | 1.582966782 | 29.06839091 | 2.05E-16 |
| JAK3 | 3.92E-06 | 1.582559674 | 8.556671249 | 0.000283792 |
| ANGPT2 | 3.98E-14 | 1.562970656 | 20.94422105 | 1.15E-11 |
| OLR1 | 6.72E-09 | 1.560539154 | 12.75399042 | 9.62E-07 |
| CD163 | 1.96E-28 | 1.556032169 | 43.11252887 | 2.59E-25 |

| AL133492.1 | 0.000104275 | 1.547633017 | 6.162395871 | 0.005275743 |
| --- | --- | --- | --- | --- |
| SBNO2 | 7.94E-06 | 1.531909406 | 7.812729115 | 0.000537402 |
| TLR2 | 9.67E-06 | 1.5146065 | 7.595001971 | 0.000638687 |
| SLC11A1 | 1.10E-20 | 1.500241161 | 29.94497546 | 6.75E-18 |
| DDIT4-AS1 | 0.000165579 | 1.489861549 | 5.633159173 | 0.007760774 |
| AL353759.1 | 2.18E-05 | 1.459141474 | 6.801123322 | 0.001317135 |
| AC007952.2 | 8.09E-07 | 1.439719831 | 8.770972024 | 7.43E-05 |
| TLR7 | 8.14E-06 | 1.424032978 | 7.247554946 | 0.000548515 |
| HLA-DQB1-AS1 | 4.66E-06 | 1.407497868 | 7.503701893 | 0.000331274 |
| FAM157B | 2.85E-06 | 1.404790338 | 7.788967997 | 0.000217773 |
| TM4SF1 | 1.45E-13 | 1.356979068 | 17.42002679 | 4.07E-11 |
| CDKN1A | 7.59E-32 | 1.355092739 | 42.17018 | 1.27E-28 |
| EMP1 | 5.59E-17 | 1.339316948 | 21.76722339 | 2.24E-14 |
| PLEK | 3.03E-20 | 1.333669212 | 26.03176659 | 1.75E-17 |
| AC025647.2 | 0.000108081 | 1.308576458 | 5.190141345 | 0.005407585 |
| TNFRSF1B | 1.02E-10 | 1.303936781 | 13.02800605 | 1.96E-08 |
| WAS | 3.55E-08 | 1.294015757 | 9.639756001 | 4.29E-06 |
| PLSCR1 | 4.96E-09 | 1.289002884 | 10.70469175 | 7.27E-07 |
| FAM157A | 4.31E-06 | 1.279800994 | 6.866212274 | 0.000308826 |
| RGS1 | 1.11E-15 | 1.259585614 | 18.83682038 | 3.80E-13 |
| SERPINE1 | 5.21E-12 | 1.259180295 | 14.20788571 | 1.17E-09 |
| ALOX5AP | 6.56E-08 | 1.258422794 | 9.039519976 | 7.62E-06 |
| FPR1 | 1.87E-11 | 1.248978439 | 13.39789505 | 3.93E-09 |
| AC011511.5 | 2.28E-09 | 1.248411621 | 10.79003042 | 3.50E-07 |
| AC130448.1 | 3.83E-05 | 1.247632634 | 5.510442964 | 0.002163363 |
| IKZF1 | 9.63E-12 | 1.24164976 | 13.67870708 | 2.07E-09 |
| MYO1F | 1.87E-05 | 1.239088618 | 5.857443575 | 0.001165254 |
| ARHGAP9 | 3.09E-05 | 1.202303767 | 5.422975122 | 0.001792597 |
| LAPTM5 | 5.38E-10 | 1.190628804 | 11.03593296 | 9.47E-08 |
| DEPP1 | 1.76E-05 | 1.185572469 | 5.637831898 | 0.001095817 |
| C3 | 8.56E-08 | 1.164539298 | 8.230381162 | 9.64E-06 |
| TFEC | 8.28E-08 | 1.160563932 | 8.218957803 | 9.38E-06 |
| DNAH11 | 5.89E-10 | 1.156173243 | 10.67127877 | 1.03E-07 |
| AC020913.3 | 2.11E-08 | 1.150660683 | 8.831238307 | 2.62E-06 |
| NCKAP1L | 2.41E-05 | 1.148111524 | 5.302328993 | 0.001443912 |
| MS4A6A | 6.04E-12 | 1.147436642 | 12.87325226 | 1.33E-09 |
| C1R | 3.46E-06 | 1.145763715 | 6.257406017 | 0.000255338 |
| TLL1 | 1.92E-05 | 1.14304987 | 5.392707586 | 0.001182774 |
| CD14 | 6.00E-19 | 1.133372226 | 20.65173635 | 2.70E-16 |
| MIR1248 | 2.22E-13 | 1.127280959 | 14.26509904 | 6.05E-11 |
| SNORA81 | 2.77E-13 | 1.127280959 | 14.15575508 | 7.21E-11 |
| ICAM1 | 4.79E-08 | 1.118767324 | 8.189343851 | 5.63E-06 |
| HAP1 | 2.28E-07 | 1.11575004 | 7.410843718 | 2.45E-05 |
| GADD45G | 9.17E-20 | 1.111305299 | 21.15664514 | 4.46E-17 |
| VEGFA | 8.63E-07 | 1.108804774 | 6.723864099 | 7.85E-05 |
| YBX3 | 6.09E-10 | 1.102375792 | 10.15851591 | 1.04E-07 |
| CD68 | 2.96E-05 | 1.101279764 | 4.987431377 | 0.001729616 |
| FYB1 | 2.43E-11 | 1.09301016 | 11.60255451 | 4.92E-09 |
| MAML3 | 6.96E-05 | 1.085372531 | 4.512344433 | 0.003736135 |
| MYOF | 5.29E-06 | 1.073706094 | 5.665676832 | 0.000371272 |
| AC105101.1 | 0.000130975 | 1.072879116 | 4.16578633 | 0.00638185 |
| FCGR1B | 2.58E-14 | 1.061439725 | 14.42378729 | 7.68E-12 |

| IL7R | 6.25E-11 | 1.055864822 | 10.77442666 | 1.24E-08 |
| --- | --- | --- | --- | --- |
| HLA-DQB1 | 1.84E-06 | 1.053919767 | 6.043204922 | 0.000151197 |
| MS4A7 | 1.82E-06 | 1.052655898 | 6.0431007 | 0.00015022 |
| FCGR1CP | 4.90E-14 | 1.033879417 | 13.76091001 | 1.39E-11 |
| PLK3 | 8.80E-09 | 1.024717232 | 8.254536803 | 1.22E-06 |
| ITGB2 | 4.94E-07 | 1.016606813 | 6.411137212 | 4.70E-05 |
| FCGR1A | 6.61E-15 | 1.00280879 | 14.21986347 | 2.10E-12 |
| CEBPD | 1.27E-08 | 1.000578359 | 7.900708584 | 1.68E-06 |
| AC024051.2 | 2.52E-07 | -1.002966997 | -6.618625588 | 2.67E-05 |
| AC024051.1 | 2.94E-06 | -1.015757974 | -5.619240877 | 0.000223215 |
| CPLX1 | 0.000116597 | -1.023943342 | -4.027489925 | 0.005757202 |
| LINC00499 | 0.000118395 | -1.081154022 | -4.245332455 | 0.005830388 |
| MET | 2.51E-05 | -1.093015184 | -5.028450064 | 0.001489722 |
| ALYREF | 1.18E-06 | -1.095273694 | -6.491396925 | 0.000104098 |
| RSPO2 | 6.65E-05 | -1.135972402 | -4.744906564 | 0.003592646 |
| RAB3B | 0.000150126 | -1.137777175 | -4.350339572 | 0.007163788 |
| SLC1A2 | 1.26E-05 | -1.155604194 | -5.662077203 | 0.000810674 |
| COL24A1 | 4.93E-07 | -1.184906435 | -7.473203392 | 4.70E-05 |
| PCDH11Y | 3.56E-07 | -1.214711176 | -7.833568691 | 3.50E-05 |
| CTXND1 | 2.62E-06 | -1.268110288 | -7.078421597 | 0.000203214 |
| GSTM5 | 2.01E-05 | -1.336081138 | -6.274350465 | 0.001231132 |
| MFSD3 | 1.23E-05 | -1.340682936 | -6.583710526 | 0.000793079 |
| RCAN2 | 4.28E-07 | -1.350900578 | -8.602802785 | 4.16E-05 |
| PTPRT | 9.68E-06 | -1.382130158 | -6.929931823 | 0.000638687 |
| HECW1 | 3.75E-06 | -1.400910509 | -7.601973463 | 0.000275595 |
| SYT2 | 0.000197798 | -1.447909265 | -5.362734711 | 0.009041424 |
| AC132217.2 | 2.81E-07 | -1.450389653 | -9.502059349 | 2.88E-05 |
| IGF2 | 2.74E-07 | -1.458146578 | -9.567622509 | 2.83E-05 |
| RORB | 2.09E-06 | -1.523491032 | -8.652624334 | 0.000167956 |
| SNORD13 | 1.38E-07 | -1.541036711 | -10.571984 | 1.51E-05 |
| AC024051.7 | 6.94E-08 | -1.557485779 | -11.14907572 | 7.92E-06 |
| IPCEF1 | 1.55E-06 | -1.559818631 | -9.060006537 | 0.000131695 |
| NDUFA4 | 1.55E-06 | -1.566075326 | -9.098408747 | 0.000131695 |
| CRYM | 2.98E-07 | -1.602331826 | -10.45646037 | 3.04E-05 |
| RGS4 | 1.13E-15 | -1.623364754 | -24.26351129 | 3.80E-13 |
| TOX | 1.20E-05 | -1.637376715 | -8.05925605 | 0.00077535 |
| KMO | 4.14E-05 | -1.677794695 | -7.354292678 | 0.002322156 |
| INS-IGF2 | 1.33E-06 | -1.683629207 | -9.895280794 | 0.000114454 |
| TMEM233 | 1.48E-05 | -1.692243505 | -8.173588611 | 0.000941819 |
| GRB14 | 7.83E-05 | -1.711503739 | -7.027670199 | 0.004108808 |
| ETV1 | 6.80E-09 | -1.716903434 | -14.02230697 | 9.67E-07 |
| PART1 | 3.39E-05 | -1.82562094 | -8.158994028 | 0.00194706 |
| SORCS1 | 5.39E-05 | -1.869924483 | -7.981171199 | 0.002972851 |
| KCNH5 | 0.000196377 | -1.918583231 | -7.112015347 | 0.008998732 |
| AC119673.3 | 6.49E-10 | -2.139742683 | -19.65969966 | 1.09E-07 |
| MTND4P24 | 6.92E-12 | -2.148139579 | -23.9728722 | 1.50E-09 |
| AC008434.1 | 3.44E-06 | -2.367322852 | -12.93371259 | 0.000255131 |
| MTCO1P53 | 1.83E-12 | -2.439527947 | -28.6363371 | 4.38E-10 |
| SLC13A4 | 1.92E-12 | -2.442697586 | -28.61953915 | 4.55E-10 |
| MT-TV | 2.35E-21 | -2.464250221 | -50.83498107 | 1.74E-18 |
| PTGER3 | 1.82E-06 | -2.662087335 | -15.27883683 | 0.00015022 |
| U52111.1 | 9.34E-24 | -3.55780942 | -81.93507711 | 9.58E-21 |

AC008670.1 6.91E-24 -3.929080805 -91.00024159 7.97E-21

DEGs CORTEX

| gene_ID | metap | metafc | idx | fdr |
| --- | --- | --- | --- | --- |
| SLC16A12 | 1.09E-07 | 4.885237101 | 34.00785607 | 7.48E-06 |
| NDC80 | 1.97E-06 | 4.885237101 | 27.86915405 | 9.62E-05 |
| FAM9C | 1.66E-05 | 4.759576083 | 22.74982643 | 0.000614495 |
| CYTIP | 1.28E-05 | 4.710945754 | 23.04443757 | 0.00049789 |
| MKI67 | 6.86E-19 | 4.455848512 | 80.93447067 | 3.33E-16 |
| IL1RL1 | 1.19E-24 | 4.197771797 | 100.4332267 | 9.54E-22 |
| TOP2A | 1.34E-23 | 4.070345681 | 93.10493324 | 9.11E-21 |
| S100A8 | 8.19E-52 | 4.047003623 | 206.7484193 | 3.78E-48 |
| SOCS3 | 9.09E-24 | 3.949778925 | 91.009521 | 6.45E-21 |
| AL355075.4 | 5.69E-12 | 3.285089305 | 36.93948851 | 1.04E-09 |
| PLA1A | 4.84E-10 | 3.202288395 | 29.82860888 | 5.70E-08 |
| AC006057.1 | 0.00015463 | 3.175109903 | 12.09940902 | 0.003828031 |
| AL365475.1 | 3.42E-05 | 3.155369055 | 14.09229829 | 0.001105734 |
| FCGBP | 3.51E-25 | 3.037603972 | 74.28353469 | 2.95E-22 |
| SERPINA3 | 1.04E-57 | 3.023628842 | 172.2960345 | 9.60E-54 |
| AL049839.2 | 6.48E-57 | 2.910098111 | 163.5129047 | 3.99E-53 |
| KNL1 | 2.88E-07 | 2.893565482 | 18.92682357 | 1.74E-05 |
| NLRC5 | 1.88E-17 | 2.835462332 | 47.42230625 | 7.25E-15 |
| S100A9 | 1.03E-44 | 2.721970882 | 119.7342258 | 3.16E-41 |
| SCIN | 1.39E-28 | 2.653967315 | 73.93269718 | 1.51E-25 |
| CD163 | 2.13E-35 | 2.650602348 | 91.90232501 | 4.36E-32 |
| FPR1 | 9.89E-22 | 2.600756525 | 54.6281535 | 5.89E-19 |
| ZFP36 | 3.65E-18 | 2.583635182 | 45.05205913 | 1.65E-15 |
| AL139317.1 | 0.000173176 | 2.573574284 | 9.680530303 | 0.004213725 |
| BCL2A1 | 1.24E-07 | 2.571881849 | 17.76504205 | 8.31E-06 |
| AC114730.3 | 4.47E-11 | 2.563483265 | 26.53143643 | 6.82E-09 |
| MS4A6A | 9.81E-31 | 2.545185964 | 76.37732272 | 1.29E-27 |
| SECTM1 | 2.46E-22 | 2.419013311 | 52.27229463 | 1.57E-19 |
| ADAMTS9 | 1.08E-18 | 2.396060617 | 43.05297211 | 5.09E-16 |
| ANKRD22 | 5.69E-05 | 2.386034777 | 10.12925409 | 0.001696307 |
| OR7E55P | 6.96E-05 | 2.37293418 | 9.86548364 | 0.002001616 |
| AL158154.3 | 6.40E-05 | 2.372413992 | 9.950142578 | 0.001866017 |
| CD93 | 3.06E-16 | 2.297959901 | 35.65057069 | 1.01E-13 |
| ADM | 1.99E-10 | 2.294560852 | 22.26003182 | 2.61E-08 |
| AC004263.2 | 3.61E-06 | 2.273845779 | 12.37673637 | 0.000162784 |
| C1QB | 1.62E-39 | 2.268210901 | 87.98491945 | 3.74E-36 |
| SERPINE1 | 7.31E-10 | 2.266802679 | 20.70918878 | 8.09E-08 |
| TLR2 | 6.51E-11 | 2.176588179 | 22.17212473 | 9.69E-09 |
| IGKC | 0.000144317 | 2.172262882 | 8.342971995 | 0.003616345 |
| LINC02271 | 1.39E-05 | 2.147222464 | 10.42753347 | 0.000537901 |
| THBD | 8.66E-05 | 2.136866798 | 8.681465144 | 0.002397212 |
| TYMP | 2.03E-40 | 2.135835782 | 84.77722647 | 5.35E-37 |
| AZGP1 | 3.27E-07 | 2.116377595 | 13.72642853 | 1.93E-05 |
| AC009879.1 | 0.000121793 | 2.101168727 | 8.224766387 | 0.003154666 |
| DEFA1 | 4.77E-17 | 2.098425532 | 34.24934216 | 1.66E-14 |
| AC104564.3 | 0.000476764 | 2.091886388 | 6.948611625 | 0.009580935 |
| KCNG4 | 9.20E-06 | 2.082896228 | 10.48957925 | 0.000375204 |
| LERFS | 6.85E-07 | 2.079319404 | 12.81694199 | 3.69E-05 |

| LDLRAD2 | 6.43E-05 | 2.069352412 | 8.674436783 | 0.001872708 |
| --- | --- | --- | --- | --- |
| HLA-DMB | 3.14E-13 | 2.059274988 | 25.74758278 | 6.90E-11 |
| NFKB2 | 2.65E-05 | 1.980511434 | 9.065322333 | 0.000902492 |
| LAIR1 | 1.07E-15 | 1.966154405 | 29.43747078 | 3.34E-13 |
| CD86 | 1.56E-08 | 1.957838671 | 15.28258246 | 1.32E-06 |
| YBX3 | 4.76E-18 | 1.95782333 | 33.91384934 | 2.05E-15 |
| HCK | 0.000223083 | 1.897363315 | 6.928285163 | 0.005221673 |
| AL731559.1 | 0.000292218 | 1.892577756 | 6.688923662 | 0.006470847 |
| AC092653.2 | 3.22E-05 | 1.880074761 | 8.445380626 | 0.001050893 |
| TNFRSF10A | 2.90E-07 | 1.877729959 | 12.27478357 | 1.75E-05 |
| OSMR | 6.68E-05 | 1.876039541 | 7.832688842 | 0.00192806 |
| ALOX5AP | 6.38E-14 | 1.871807386 | 24.69820077 | 1.53E-11 |
| F13A1 | 1.19E-12 | 1.870739374 | 22.30733406 | 2.39E-10 |
| AL662890.1 | 0.000121346 | 1.854539309 | 7.262328866 | 0.003147573 |
| PIK3AP1 | 2.83E-10 | 1.848677029 | 17.6502024 | 3.59E-08 |
| RHOXF1-AS1 | 1.00E-10 | 1.822452452 | 18.22226133 | 1.42E-08 |
| DEFA3 | 1.01E-14 | 1.817048844 | 25.43133932 | 2.82E-12 |
| RGS1 | 4.43E-20 | 1.800609258 | 34.84855674 | 2.34E-17 |
| C1QA | 8.94E-31 | 1.796297906 | 53.9765322 | 1.27E-27 |
| TFCP2L1 | 4.74E-05 | 1.786658189 | 7.72637449 | 0.001465419 |
| SLC7A2 | 3.98E-08 | 1.784303075 | 13.2032662 | 3.07E-06 |
| CCDC200 | 3.67E-08 | 1.775179958 | 13.20010495 | 2.86E-06 |
| AL031595.1 | 0.000385821 | 1.771514111 | 6.047265977 | 0.008078616 |
| GTF3C2-AS1 | 7.99E-05 | 1.754451115 | 7.188634379 | 0.002246653 |
| DEFA1B | 1.00E-15 | 1.742327549 | 26.13166587 | 3.20E-13 |
| MICB | 0.000142717 | 1.731698316 | 6.659287548 | 0.00359087 |
| LILRB3 | 0.000100116 | 1.725767367 | 6.902204219 | 0.002703119 |
| IL1B | 3.09E-08 | 1.715344046 | 12.88167491 | 2.45E-06 |
| ADAMTS9-AS1 | 1.49E-05 | 1.711496963 | 8.263136846 | 0.000563474 |
| PDLIM1 | 1.65E-08 | 1.706109305 | 13.27733646 | 1.39E-06 |
| SYK | 2.32E-06 | 1.677658508 | 9.45371002 | 0.000111302 |
| GPR4 | 8.44E-06 | 1.677162018 | 8.509677669 | 0.00034622 |
| LINC02485 | 8.72E-05 | 1.645596536 | 6.680253714 | 0.002409199 |
| AC009831.4 | 6.22E-05 | 1.640802788 | 6.901268081 | 0.001829906 |
| NSA2P4 | 3.46E-05 | 1.635913046 | 7.29851864 | 0.00111192 |
| AL034550.2 | 0.000276442 | 1.627188251 | 5.790181356 | 0.006241225 |
| AC022144.1 | 6.46E-09 | 1.61977336 | 13.26595004 | 6.02E-07 |
| LGALS9B | 0.000459909 | 1.592201952 | 5.313700799 | 0.009282616 |
| ANGPTL4 | 2.29E-10 | 1.583642642 | 15.26727319 | 2.98E-08 |
| STC1 | 2.34E-06 | 1.581019453 | 8.902175225 | 0.0001117 |
| LGALS9C | 0.000248975 | 1.57895069 | 5.690293575 | 0.005733245 |
| PARVG | 3.93E-08 | 1.578071866 | 11.68645763 | 3.04E-06 |
| CDC20B | 0.000176834 | 1.566439162 | 5.877959942 | 0.004285788 |
| ARHGDIB | 1.90E-08 | 1.555577443 | 12.00998535 | 1.56E-06 |
| CDK2 | 7.73E-05 | 1.554829265 | 6.393346965 | 0.002185637 |
| MS4A7 | 3.23E-10 | 1.541003799 | 14.62597116 | 4.00E-08 |
| LAPTM5 | 9.53E-11 | 1.535602073 | 15.38833123 | 1.37E-08 |
| AC016355.1 | 8.10E-06 | 1.52860562 | 7.782737329 | 0.000334746 |
| CD14 | 4.84E-20 | 1.512323182 | 29.21127191 | 2.48E-17 |
| PARP10 | 2.13E-05 | 1.499009249 | 7.003978669 | 0.00075078 |
| FCGR2C | 1.97E-09 | 1.498436551 | 13.0453504 | 1.99E-07 |
| CNTNAP3C | 0.000235079 | 1.486393372 | 5.393803222 | 0.005460934 |

| AL513523.3 | 0.000406334 | 1.484638568 | 5.034582617 | 0.008393936 |
| --- | --- | --- | --- | --- |
| USP18 | 1.68E-07 | 1.480341542 | 10.0305124 | 1.09E-05 |
| EMP1 | 1.66E-21 | 1.479955362 | 30.75438481 | 9.27E-19 |
| VSIR | 1.03E-05 | 1.477000298 | 7.36711104 | 0.000409274 |
| HLA-DRA | 6.36E-18 | 1.47299257 | 25.33004077 | 2.67E-15 |
| IL4R | 1.25E-06 | 1.453447083 | 8.579213829 | 6.37E-05 |
| AC116366.2 | 0.000108391 | 1.448717554 | 5.744174759 | 0.0028761 |
| AIF1 | 7.25E-14 | 1.448692327 | 19.03547392 | 1.69E-11 |
| AL606970.3 | 8.94E-05 | 1.448642592 | 5.865016746 | 0.002453441 |
| FCGR1CP | 1.29E-15 | 1.447043435 | 21.54752684 | 3.89E-13 |
| AL139287.1 | 2.12E-05 | 1.431583449 | 6.690289338 | 0.000750586 |
| HLA-DRB1 | 2.81E-35 | 1.426616875 | 49.29219193 | 5.18E-32 |
| LCP1 | 3.23E-08 | 1.425676317 | 10.67921984 | 2.55E-06 |
| FGL2 | 6.37E-06 | 1.425569548 | 7.407205459 | 0.000269756 |
| FYB1 | 2.02E-19 | 1.424767515 | 26.6342632 | 1.01E-16 |
| HLA-DQA1 | 5.05E-06 | 1.418838112 | 7.514805751 | 0.000219058 |
| RGS16 | 7.69E-15 | 1.410622302 | 19.90952585 | 2.19E-12 |
| FCGR1A | 2.61E-14 | 1.409853266 | 19.15055236 | 6.79E-12 |
| CASP4 | 8.95E-08 | 1.402628946 | 9.885830899 | 6.38E-06 |
| PTPRC | 2.32E-06 | 1.40099084 | 7.893606199 | 0.000111302 |
| CD44 | 8.72E-13 | 1.39771059 | 16.85534559 | 1.81E-10 |
| RUNX1 | 0.000410208 | 1.384249975 | 4.688448974 | 0.008455046 |
| IFI16 | 6.64E-15 | 1.380936587 | 19.57861602 | 1.95E-12 |
| AL590652.1 | 6.04E-07 | 1.380771696 | 8.587295893 | 3.31E-05 |
| MNDA | 9.41E-12 | 1.380124048 | 15.21805258 | 1.61E-09 |
| CP | 9.25E-08 | 1.379024223 | 9.699872011 | 6.55E-06 |
| SELL | 7.61E-06 | 1.374139778 | 7.033695376 | 0.000315822 |
| TBXAS1 | 0.000444592 | 1.373789834 | 4.6049961 | 0.009032703 |
| AC240565.3 | 1.58E-05 | 1.352130256 | 6.491721029 | 0.000591859 |
| DEPP1 | 1.44E-09 | 1.35200567 | 11.95364809 | 1.52E-07 |
| SLC11A1 | 2.09E-14 | 1.351118976 | 18.48178189 | 5.72E-12 |
| FAAP100 | 5.20E-05 | 1.345745895 | 5.765331858 | 0.001589522 |
| SPOCD1 | 6.00E-29 | 1.344033547 | 37.93116716 | 6.92E-26 |
| OLR1 | 6.15E-10 | 1.342906035 | 12.36922425 | 6.97E-08 |
| ANKRD49P2 | 1.18E-05 | 1.339067464 | 6.597236717 | 0.000465139 |
| ATE1-AS1 | 0.000142593 | 1.337276418 | 5.143032751 | 0.00359087 |
| S100A4 | 8.06E-07 | 1.337261849 | 8.148887102 | 4.29E-05 |
| CPVL | 4.24E-07 | 1.331357253 | 8.484715328 | 2.44E-05 |
| HCLS1 | 0.000285868 | 1.319828146 | 4.677252903 | 0.006407046 |
| SIGLEC10 | 1.13E-07 | 1.312552687 | 9.118695307 | 7.64E-06 |
| SPP1 | 3.46E-25 | 1.292488386 | 31.61471992 | 2.95E-22 |
| SUMO4 | 0.00038253 | 1.272049249 | 4.347017972 | 0.008034914 |
| RCC2P6 | 0.000161862 | 1.269969553 | 4.814270997 | 0.003991008 |
| C5AR1 | 1.64E-09 | 1.266439321 | 11.12633251 | 1.71E-07 |
| NIBAN1 | 3.57E-14 | 1.261236206 | 16.96045871 | 8.91E-12 |
| POM121B | 6.27E-05 | 1.25267466 | 5.264594457 | 0.001835302 |
| LTBR | 2.71E-05 | 1.249073902 | 5.70360397 | 0.000921626 |
| CD74 | 1.12E-07 | 1.242267715 | 8.636368412 | 7.61E-06 |
| KIAA0040 | 1.28E-07 | 1.236009851 | 8.517845398 | 8.59E-06 |
| EVI2B | 2.80E-05 | 1.227725219 | 5.588891978 | 0.000948405 |
| GBP5 | 3.21E-14 | 1.219029361 | 16.44920893 | 8.12E-12 |
| PECAM1 | 6.71E-09 | 1.21601223 | 9.938985495 | 6.23E-07 |

| FKBP5 | 3.41E-13 | 1.210967572 | 15.09779786 | 7.40E-11 |
| --- | --- | --- | --- | --- |
| TNFRSF1A | 8.16E-05 | 1.209688345 | 4.945870268 | 0.002282058 |
| C1R | 6.38E-12 | 1.206573565 | 13.50774875 | 1.13E-09 |
| UNC93B1 | 0.000264567 | 1.203182397 | 4.304341735 | 0.006009873 |
| APBB1IP | 1.15E-08 | 1.193895592 | 9.479592406 | 9.95E-07 |
| PLEK | 1.58E-09 | 1.191464462 | 10.48588683 | 1.66E-07 |
| RELL1 | 0.000345653 | 1.190489741 | 4.120713584 | 0.007379786 |
| AC010624.5 | 3.94E-26 | 1.187204254 | 30.15979606 | 3.83E-23 |
| HLA-B | 2.56E-11 | 1.186209246 | 12.5640539 | 3.94E-09 |
| ABCD4 | 0.000354168 | 1.180259821 | 4.07282971 | 0.007526781 |
| ELOVL7 | 1.03E-05 | 1.179875493 | 5.885316584 | 0.000409274 |
| CTSC | 0.000208149 | 1.173756093 | 4.321330472 | 0.004940998 |
| TAGLN2 | 6.21E-08 | 1.170610663 | 8.436742123 | 4.55E-06 |
| PLAUR | 3.33E-06 | 1.167121289 | 6.393115998 | 0.000151805 |
| HMGN1P36 | 0.000143341 | 1.16653597 | 4.483731732 | 0.003601665 |
| AC016876.2 | 6.14E-09 | 1.163224369 | 9.55236291 | 5.75E-07 |
| SLA | 1.49E-07 | 1.161537629 | 7.931069537 | 9.80E-06 |
| AL450345.2 | 0.00027841 | 1.159461936 | 4.122251768 | 0.006278003 |
| CEBPD | 1.41E-09 | 1.158692856 | 10.25560674 | 1.50E-07 |
| BTK | 0.000245085 | 1.148622577 | 4.147312086 | 0.005665765 |
| VAMP8 | 2.87E-07 | 1.147045562 | 7.503758322 | 1.74E-05 |
| SYTL4 | 7.86E-06 | 1.140335341 | 5.821189796 | 0.000325297 |
| LPCAT2 | 0.000164804 | 1.138012895 | 4.305139014 | 0.004046204 |
| ANGPT2 | 2.20E-12 | 1.137061471 | 13.25490284 | 4.33E-10 |
| HAP1 | 3.77E-09 | 1.135194836 | 9.562558797 | 3.68E-07 |
| ANXA2 | 1.89E-05 | 1.134172617 | 5.356296637 | 0.000685809 |
| CYBB | 1.93E-05 | 1.124176741 | 5.299894758 | 0.00069476 |
| NCF1 | 0.000121996 | 1.118330141 | 4.376756528 | 0.003155501 |
| LYN | 1.66E-07 | 1.112348437 | 7.542615856 | 1.08E-05 |
| HLA-E | 0.000117319 | 1.111302048 | 4.368117363 | 0.003064575 |
| H2AC19 | 2.98E-05 | 1.106138264 | 5.006468153 | 0.000989161 |
| PLAC8 | 2.22E-05 | 1.10556957 | 5.144697872 | 0.000779826 |
| ITPKC | 0.000109032 | 1.102798525 | 4.369779727 | 0.002888956 |
| SMAD1-AS1 | 0.00038505 | 1.097176886 | 3.746291495 | 0.00807163 |
| WAS | 3.27E-07 | 1.095586671 | 7.105576273 | 1.93E-05 |
| TYROBP | 6.04E-09 | 1.089133148 | 8.951328934 | 5.69E-07 |
| GBP2 | 6.95E-33 | 1.080486145 | 34.74608732 | 1.17E-29 |
| VWF | 0.000378369 | 1.078167017 | 3.689578716 | 0.007960436 |
| NMI | 7.88E-11 | 1.076371596 | 10.87538691 | 1.15E-08 |
| C1orf162 | 0.000289313 | 1.076322015 | 3.808707897 | 0.006437922 |
| DRAXIN | 8.53E-06 | 1.073929109 | 5.443549386 | 0.000349484 |
| S100A11 | 5.27E-07 | 1.072836936 | 6.735186323 | 2.93E-05 |
| HMGB1P20 | 4.95E-06 | 1.062209267 | 5.635078385 | 0.000215774 |
| IRF1 | 4.60E-17 | 1.061131336 | 17.33645938 | 1.63E-14 |
| H2AC18 | 1.59E-05 | 1.060957152 | 5.089850831 | 0.000594841 |
| TNFAIP3 | 1.73E-05 | 1.058722856 | 5.04100962 | 0.000633464 |
| IL10RB | 0.000486584 | 1.052237806 | 3.485897382 | 0.009725369 |
| RASL12 | 0.000404707 | 1.051307809 | 3.566939067 | 0.008369692 |
| PLAC4 | 5.60E-05 | 1.048758748 | 4.459412312 | 0.001675131 |
| TAGLN | 1.76E-08 | 1.04812217 | 8.127863285 | 1.46E-06 |
| PSMB8-AS1 | 7.64E-13 | 1.045380639 | 12.66702251 | 1.60E-10 |
| FCGR1B | 1.33E-10 | 1.039519019 | 10.26502429 | 1.81E-08 |

| CD68 | 1.82E-08 | 1.032350514 | 7.989674144 | 1.50E-06 |
| --- | --- | --- | --- | --- |
| PIM1 | 3.63E-05 | 1.028951887 | 4.568066759 | 0.001159331 |
| ADAMTS1 | 2.37E-05 | 1.028330046 | 4.755533692 | 0.000822568 |
| NEAT1 | 8.18E-08 | 1.025341093 | 7.266804219 | 5.90E-06 |
| SRGN | 3.02E-20 | 1.025106114 | 20.00954074 | 1.64E-17 |
| TMBIM1 | 3.07E-05 | 1.022824062 | 4.616382977 | 0.001011259 |
| AC010260.1 | 5.45E-08 | 1.012092237 | 7.351573214 | 4.04E-06 |
| PARP9 | 2.10E-14 | 1.007054469 | 13.7733196 | 5.72E-12 |
| ATF3 | 4.88E-24 | 1.001919448 | 23.35637329 | 3.60E-21 |
| RNASE1 | 1.23E-09 | -1.00774272 | -8.979556645 | 1.32E-07 |
| TMEM125 | 9.90E-11 | -1.011889 | -10.12333281 | 1.42E-08 |
| PLP1 | 8.23E-11 | -1.013225735 | -10.21794715 | 1.20E-08 |
| AC073283.3 | 2.46E-06 | -1.033345618 | -5.795512491 | 0.000116944 |
| TYMSOS | 7.84E-12 | -1.036790221 | -11.51421626 | 1.37E-09 |
| AC024051.8 | 0.000184107 | -1.042397509 | -3.893280193 | 0.004432982 |
| PTGDS | 7.58E-07 | -1.042642774 | -6.381310294 | 4.06E-05 |
| RNU2-2P | 0.000254564 | -1.048989702 | -3.770282685 | 0.00582109 |
| FSCN3 | 0.000151481 | -1.059029978 | -4.045116119 | 0.003760142 |
| ARF5 | 0.000136355 | -1.062871416 | -4.108347701 | 0.003467333 |
| AC004540.1 | 0.000428862 | -1.073688133 | -3.615840663 | 0.008771012 |
| MDH1 | 4.91E-05 | -1.080651447 | -4.656617554 | 0.001513245 |
| DBNDD1 | 9.04E-05 | -1.10052308 | -4.450156844 | 0.00247424 |
| ARHGEF17 | 1.05E-07 | -1.122059646 | -7.830425358 | 7.27E-06 |
| AC006441.3 | 0.000325647 | -1.125200455 | -3.923859073 | 0.00705046 |
| CDC42EP4 | 0.000165196 | -1.146753599 | -4.337022958 | 0.004046204 |
| MAL2 | 6.20E-08 | -1.150670925 | -8.293628608 | 4.55E-06 |
| KCNJ3 | 0.000175793 | -1.155257818 | -4.337990911 | 0.00426616 |
| RAB11FIP5 | 2.82E-07 | -1.171296661 | -7.671006037 | 1.73E-05 |
| PPP3R1 | 2.18E-05 | -1.18399996 | -5.520176496 | 0.000765507 |
| AC253536.7 | 0.000123061 | -1.200533903 | -4.693943991 | 0.003169716 |
| DDTL | 6.65E-05 | -1.20618657 | -5.038579317 | 0.001921493 |
| DDT | 6.27E-05 | -1.20750665 | -5.074931919 | 0.001835302 |
| PINLYP | 9.79E-05 | -1.215637658 | -4.873700384 | 0.002651329 |
| CENPS-CORT | 0.000193902 | -1.230653357 | -4.568698201 | 0.004623441 |
| ZNF428 | 0.000117211 | -1.237037272 | -4.862832782 | 0.003064575 |
| CYP2E1 | 0.00018943 | -1.246014412 | -4.638353915 | 0.004549264 |
| NDUFA4 | 6.81E-15 | -1.259592002 | -17.84436974 | 1.97E-12 |
| ETV5 | 9.54E-06 | -1.280961098 | -6.431033551 | 0.000385288 |
| RRAGA | 4.36E-05 | -1.30684471 | -5.698694137 | 0.001373634 |
| GPRASP2 | 0.000313815 | -1.307261852 | -4.579764394 | 0.006866753 |
| ARHGEF25 | 0.000111138 | -1.32655033 | -5.245362047 | 0.002928511 |
| TRAPPC4 | 8.32E-05 | -1.329660416 | -5.425127423 | 0.002313162 |
| TUBBP1 | 0.000452724 | -1.340180657 | -4.481787407 | 0.009167658 |
| AC138811.2 | 7.05E-06 | -1.365103716 | -7.032942984 | 0.000295815 |
| NDRG4 | 3.98E-09 | -1.381238926 | -11.60255848 | 3.87E-07 |
| AC024051.9 | 2.93E-05 | -1.390179714 | -6.302363982 | 0.000975936 |
| AC024051.4 | 0.000164915 | -1.398557099 | -5.2903777 | 0.004046204 |
| HMGB1P29 | 9.50E-06 | -1.399331527 | -7.02762762 | 0.000384878 |
| PIP4K2C | 7.01E-06 | -1.440161178 | -7.423318613 | 0.000294747 |
| SVOP | 4.58E-07 | -1.444730471 | -9.158431347 | 2.59E-05 |
| AC024051.10 | 4.53E-05 | -1.481494905 | -6.436150916 | 0.001416463 |
| RNU2-1 | 4.70E-05 | -1.483413628 | -6.420419662 | 0.001458023 |

| NDUFB4 | 4.17E-05 | -1.499231894 | -6.566907101 | 0.001315476 |
| --- | --- | --- | --- | --- |
| HINT1 | 9.86E-08 | -1.547180814 | -10.83978523 | 6.90E-06 |
| AL133352.1 | 9.46E-06 | -1.564103883 | -7.858172124 | 0.000384004 |
| PPIAP22 | 8.39E-06 | -1.576056415 | -8.000580278 | 0.000345021 |
| AC024051.3 | 5.42E-05 | -1.578422111 | -6.733509263 | 0.001635662 |
| AC024051.1 | 3.49E-06 | -1.609310281 | -8.782683821 | 0.000158272 |
| MIR1248 | 2.56E-11 | -1.618350418 | -17.14114993 | 3.94E-09 |
| SNORA81 | 2.41E-11 | -1.622494489 | -17.22871835 | 3.77E-09 |
| ATP6V0E2-AS1 | 1.47E-05 | -1.65307233 | -7.988433465 | 0.000563474 |
| GPD1L | 0.000331043 | -1.682303117 | -5.854609318 | 0.007158902 |
| AC139256.2 | 3.16E-10 | -1.740134263 | -16.53173045 | 3.94E-08 |
| AC011603.3 | 1.31E-07 | -1.742208091 | -11.99369413 | 8.70E-06 |
| TUBA1B | 3.21E-09 | -1.796180665 | -15.25695393 | 3.15E-07 |
| AC024051.7 | 3.83E-06 | -1.796629865 | -9.73106668 | 0.000171467 |
| AC012146.1 | 2.92E-05 | -1.851645933 | -8.395871239 | 0.000975933 |
| PGAM4 | 6.79E-08 | -2.12396506 | -15.22487529 | 4.94E-06 |
| PPIAP29 | 1.55E-08 | -2.372425149 | -18.52464954 | 1.32E-06 |
| MTCO1P53 | 8.25E-18 | -2.433045418 | -41.56513263 | 3.39E-15 |
| AC119673.3 | 1.86E-11 | -2.537097315 | -27.2216127 | 2.99E-09 |
| PPIAP46 | 5.36E-10 | -2.967142856 | -27.50878832 | 6.18E-08 |
| MTND4P24 | 1.63E-16 | -4.058440671 | -64.07916191 | 5.46E-14 |
| AC008670.1 | 4.58E-22 | -4.899575184 | -104.5515117 | 2.82E-19 |
