# Supplementary material for: Transcriptome analysis of six tissues obtained post‐mortem from sepsis patients
Source: J Cell Mol Med. 2023 Sep 20;27(20):3157–67. doi: 10.1111/jcmm.17938 (PMC10568675; doi:10.1111/jcmm.17938)
Supplement: Supplementary file 3 — Table S3. [file JCMM-27-3157-s001.docx]

| **PATHWAY** | **colon** | **cortex** | **hippocampus** | **heart** | **kidney** | **lung** |
| --- | --- | --- | --- | --- | --- | --- |
| antimicrobial peptides | 1.972259 | 2.213256 | 1.984387 | 0.000000 | 2.172684 | 0.000000 |
| olfactory signaling pathway | −1.941446 | 0.000000 | 1.937934 | 0.000000 | 1.814010 | 1.710141 |
| interleukin 2 signaling | 0.000000 | 0.000000 | 1.770624 | 0.000000 | 0.000000 | 1.880928 |
| blood group systems biosynthesis | 0.000000 | 0.000000 | 0.000000 | 0.000000 | 0.000000 | 2.074480 |
| organic cation transport | 0.000000 | 0.000000 | 0.000000 | 0.000000 | 0.000000 | 1.816151 |
| innate immune system | 1.429210 | 1.728225 | 1.577395 | 1.709353 | 0.000000 | 0.000000 |
| neutrophil degranulation | 1.473047 | 1.841384 | 1.600909 | 1.823743 | 0.000000 | 0.000000 |
| fcgr activation | 1.694849 | 1.846681 | 1.912718 | 1.870295 | 0.000000 | 0.000000 |
| cytokine signaling | 1.389613 | 2.001155 | 1.983752 | 1.897861 | 0.000000 | 0.000000 |
| cytokine signaling in immune system | 0.000000 | 1.800727 | 1.690810 | 1.721998 | −1.309343 | 0.000000 |
| interleukin 10 signaling | 0.000000 | 2.288941 | 2.266978 | 2.096343 | 0.000000 | 0.000000 |
| interferon gamma signaling | 0.000000 | 2.323714 | 2.118746 | 1.799155 | 0.000000 | 0.000000 |
| interleukin 4 and interleukin 13 signaling | 0.000000 | 2.332689 | 2.310439 | 1.693768 | 0.000000 | 0.000000 |
| interleukin 6 signaling | 0.000000 | 1.728016 | 1.757767 | 1.790579 | 0.000000 | 0.000000 |
| leishmania infection | 0.000000 | 1.854831 | 1.851250 | 1.560150 | 0.000000 | 0.000000 |
| interferon alpha beta signaling | 0.000000 | 2.190238 | 1.874244 | 1.739690 | 0.000000 | 0.000000 |
| interferon signaling | 0.000000 | 2.007208 | 1.657973 | 1.777592 | 0.000000 | 0.000000 |
| gpcr ligand binding | 0.000000 | 0.000000 | 1.525205 | 0.000000 | 0.000000 | 0.000000 |
| purinergic signaling in leishmaniasis infection | 0.000000 | 0.000000 | 1.832988 | 0.000000 | 0.000000 | 0.000000 |
| traf3 dependent irf activation pathway | 0.000000 | 0.000000 | 1.845521 | 0.000000 | 0.000000 | 0.000000 |
| class a 1 rhodopsin like receptors | 0.000000 | 0.000000 | 1.655810 | 0.000000 | 0.000000 | 0.000000 |
| fcgr3a mediated il10 synthesis | 0.000000 | 0.000000 | 1.636612 | 0.000000 | 0.000000 | 0.000000 |
| interleukin 9 signaling | 0.000000 | 0.000000 | 1.761117 | 0.000000 | 0.000000 | 0.000000 |
| fceri mediated ca 2 mobilization | 0.000000 | 0.000000 | 1.706647 | 0.000000 | 0.000000 | 0.000000 |
| interleukin 7 signaling | 0.000000 | 0.000000 | 1.720151 | 0.000000 | 0.000000 | 0.000000 |
| cell surface interactions at the vascular wall | 0.000000 | 1.926991 | 1.537814 | 0.000000 | −1.786232 | 0.000000 |
| immunoregulatory interactions between a lymphoid and a non lymphoid cell | 0.000000 | 1.999470 | 2.102778 | 0.000000 | 0.000000 | 1.767259 |
| complement cascade | 0.000000 | 2.241075 | 1.983185 | 0.000000 | 0.000000 | 0.000000 |
| initial triggering of complement | 0.000000 | 2.076500 | 1.987870 | 0.000000 | 0.000000 | 0.000000 |
| alternative complement activation | 0.000000 | 1.605074 | 1.645232 | 0.000000 | 0.000000 | 0.000000 |
| pd 1 signaling | 0.000000 | 1.824504 | 1.699019 | 0.000000 | 0.000000 | 0.000000 |
| regulation of ifna signaling | 0.000000 | 1.833478 | 1.673520 | 0.000000 | 0.000000 | 0.000000 |
| signaling by leptin | 0.000000 | 1.704599 | 1.737416 | 0.000000 | 0.000000 | 0.000000 |
| toll like receptor cascades | 0.000000 | 1.738670 | 1.703387 | 0.000000 | 0.000000 | 0.000000 |
| interleukin 2 family signaling | 0.000000 | 1.836288 | 1.761804 | 0.000000 | 0.000000 | 0.000000 |

| **PATHWAY** | **colon** | **cortex** | **hippocampus** | **heart** | **kidney** | **lung** |
| --- | --- | --- | --- | --- | --- | --- |
| interleukin 6 family signaling | 0 | 1.789290 | 1.819751 | 0.000000 | 0.000000 | 0.000000 |
| regulation of tlr by endogenous ligand | 0 | 1.961051 | 1.822433 | 0.000000 | 0.000000 | 0.000000 |
| generation of second messenger molecules | 0 | 1.892535 | 1.970797 | 0.000000 | 0.000000 | 0.000000 |
| interleukin 15 signaling | 0 | 1.745590 | 1.958065 | 0.000000 | 0.000000 | 0.000000 |
| rho gtpases activate nadph oxidases | 0 | 1.797994 | 2.088242 | 0.000000 | 0.000000 | 0.000000 |
| slc mediated transmembrane transport | 0 | 0.000000 | −1.372039 | 1.523387 | 0.000000 | 0.000000 |
| organic anion transporters | 0 | 0.000000 | 0.000000 | 1.505067 | 0.000000 | 0.000000 |
| interleukin 12 family signaling | 0 | 0.000000 | 0.000000 | 1.623993 | 0.000000 | 0.000000 |
| listeria monocytogenes entry into host cells | 0 | 0.000000 | 0.000000 | 1.681469 | 0.000000 | 0.000000 |
| tnfr2 non canonical nf kb pathway | 0 | 0.000000 | 0.000000 | 1.682807 | 0.000000 | 0.000000 |
| assembly of active lpl and lipc lipase complexes | 0 | 0.000000 | 0.000000 | 1.962983 | 0.000000 | 0.000000 |
| nucleotide salvage | 0 | 0.000000 | 0.000000 | 1.918167 | 0.000000 | 0.000000 |
| ros and rns production in phagocytes | 0 | 0.000000 | 0.000000 | 1.881988 | 0.000000 | 0.000000 |
| eicosanoid ligand binding receptors | 0 | 0.000000 | 0.000000 | 1.808418 | 0.000000 | 0.000000 |
| interleukin 35 signalling | 0 | 0.000000 | 0.000000 | 1.807805 | 0.000000 | 0.000000 |
| digestion | 0 | 0.000000 | 0.000000 | 1.851189 | 0.000000 | 0.000000 |
| pyrimidine salvage | 0 | 0.000000 | 0.000000 | 1.842664 | 0.000000 | 0.000000 |
| phenylalanine and tyrosine metabolism | 0 | 0.000000 | 0.000000 | 1.726746 | 0.000000 | 0.000000 |
| phenylalanine metabolism | 0 | 0.000000 | 0.000000 | 1.719656 | 0.000000 | 0.000000 |
| activated ntrk2 signals through fyn | 0 | 0.000000 | 0.000000 | 1.743274 | 0.000000 | 0.000000 |
| ctla4 inhibitory signaling | 0 | 0.000000 | 0.000000 | 1.764414 | 0.000000 | 0.000000 |
| synthesis of dolichyl phosphate | 0 | 0.000000 | 0.000000 | 1.755405 | 0.000000 | 0.000000 |
| adaptive immune system | 0 | 1.284468 | 0.000000 | 1.275000 | −1.266481 | 0.000000 |
| interleukin 1 family signaling | 0 | 1.543411 | 0.000000 | 2.029687 | 0.000000 | 0.000000 |
| interleukin 18 signaling | 0 | 1.536270 | 0.000000 | 1.632023 | 0.000000 | 0.000000 |
| dissolution of fibrin clot | 0 | 1.738376 | 0.000000 | 1.764543 | 0.000000 | 0.000000 |
| interleukin 37 signaling | 0 | 1.785533 | 0.000000 | 1.732711 | 0.000000 | 0.000000 |
| interleukin 1 processing | 0 | 1.780470 | 0.000000 | 1.850922 | 0.000000 | 0.000000 |
| plasma lipoprotein remodeling | 0 | 1.720342 | 0.000000 | 1.861317 | 0.000000 | 0.000000 |
| response to elevated platelet cytosolic ca2 | 0 | 1.598448 | −1.490992 | 0.000000 | −1.743225 | 0.000000 |
| hemostasis | 0 | 1.471687 | 0.000000 | 0.000000 | −1.782869 | 0.000000 |
| regulation of insulin like growth factor igf transport and uptake by insulin like growth factor binding proteins igfbps | 0 | 1.557671 | 0.000000 | 0.000000 | −1.551272 | 0.000000 |
| integrin cell surface interactions | 0 | 1.892637 | 0.000000 | 0.000000 | −1.782209 | 0.000000 |
| degradation of the extracellular matrix | 0 | 1.586265 | 0.000000 | 0.000000 | −2.011482 | 0.000000 |
| extracellular matrix organization | 0 | 1.614479 | 0.000000 | 0.000000 | −2.078979 | −1.261483 |

| **PATHWAY** | **colon** | **cortex** | **hippocampus** | **heart** | **kidney** | **lung** |
| --- | --- | --- | --- | --- | --- | --- |
| anti inflammatory response favouring leishmania parasite infection | 0.000000 | 1.699136 | 0.000000 | 0.000000 | 0.000000 | 1.482566 |
| irak4 deficiency tlr2 4 | 0.000000 | 1.845325 | 0.000000 | 0.000000 | 0.000000 | 1.861509 |
| growth hormone receptor signaling | 0.000000 | 1.957707 | 0.000000 | 0.000000 | 0.000000 | 0.000000 |
| antigen activates b cell receptor bcr leading to generation of second messengers | 0.000000 | 1.809314 | 0.000000 | 0.000000 | 0.000000 | 0.000000 |
| pecam1 interactions | 0.000000 | 1.816600 | 0.000000 | 0.000000 | 0.000000 | 0.000000 |
| formation of the cornified envelope | 0.000000 | 1.880264 | 0.000000 | 0.000000 | 0.000000 | 0.000000 |
| interleukin 20 family signaling | 0.000000 | 1.873210 | 0.000000 | 0.000000 | 0.000000 | 0.000000 |
| diseases of immune system | 0.000000 | 1.845264 | 0.000000 | 0.000000 | 0.000000 | 0.000000 |
| keratinization | 0.000000 | 1.860113 | 0.000000 | 0.000000 | 0.000000 | 0.000000 |
| beta defensins | 0.000000 | 1.523215 | 0.000000 | 0.000000 | 0.000000 | 0.000000 |
| pre notch expression and processing | 0.000000 | 1.521004 | 0.000000 | 0.000000 | 0.000000 | 0.000000 |
| diseases associated with glycosaminoglycan metabolism | 0.000000 | 1.758832 | 0.000000 | 0.000000 | 0.000000 | 0.000000 |
| tnfr1 induced proapoptotic signaling | 0.000000 | 1.748004 | 0.000000 | 0.000000 | 0.000000 | 0.000000 |
| endosomal vacuolar pathway | 0.000000 | 1.716991 | 0.000000 | 0.000000 | 0.000000 | 0.000000 |
| meiotic recombination | 0.000000 | 1.733157 | 0.000000 | 0.000000 | 0.000000 | 0.000000 |
| interleukin 3 interleukin 5 and gm csf signaling | 0.000000 | 1.618386 | 0.000000 | 0.000000 | 0.000000 | 0.000000 |
| creation of c4 and c2 activators | 0.000000 | 1.688890 | 0.000000 | 0.000000 | 0.000000 | 0.000000 |
| g0 and early g1 | 0.000000 | 1.675418 | 0.000000 | 0.000000 | 0.000000 | 0.000000 |
| tp53 regulates transcription of cell death genes | 0.000000 | 1.672374 | 0.000000 | 0.000000 | 0.000000 | 0.000000 |
| transcriptional regulation of granulopoiesis | 0.000000 | 2.009258 | 1.705272 | 0.000000 | 0.000000 | −1.937580 |
| estrogen dependent gene expression | 0.000000 | 1.462864 | 0.000000 | 0.000000 | 0.000000 | −1.681861 |
| dna damage telomere stress induced senescence | 0.000000 | 1.611942 | 0.000000 | 0.000000 | 0.000000 | −1.804545 |
| runx1 regulates genes involved in megakaryocyte differentiation and platelet function | 0.000000 | 1.657110 | 0.000000 | 0.000000 | 0.000000 | −1.706082 |
| activated pkn1 stimulates transcription of ar androgen receptor regulated genes klk2 and klk3 | 0.000000 | 1.754949 | 0.000000 | 0.000000 | 0.000000 | −1.923275 |
| deposition of new cenpa containing nucleosomes at the centromere | 0.000000 | 1.761253 | 0.000000 | 0.000000 | 0.000000 | −1.847297 |
| prc2 methylates histones and dna | 0.000000 | 1.844864 | 0.000000 | 0.000000 | 0.000000 | −2.087938 |
| sirt1 negatively regulates rrna expression | 0.000000 | 1.708092 | 0.000000 | 0.000000 | 0.000000 | −2.059043 |
| scavenging of heme from plasma | 0.000000 | 0.000000 | 0.000000 | 1.904994 | 0.000000 | 0.000000 |
| lipid particle organization | 0.000000 | 0.000000 | 0.000000 | 0.000000 | 1.519616 | 0.000000 |
| metal ion slc transporters | 0.000000 | 0.000000 | 0.000000 | 0.000000 | 1.877266 | 0.000000 |
| cargo concentration in the er | 0.000000 | 0.000000 | 0.000000 | 0.000000 | 1.780596 | 0.000000 |
| zinc transporters | 0.000000 | 0.000000 | 0.000000 | 0.000000 | 1.800304 | 0.000000 |
| alpha defensins | 1.640599 | 1.600139 | 0.000000 | 0.000000 | 1.845171 | 0.000000 |
| defensins | 1.887890 | 1.857948 | 0.000000 | 0.000000 | 1.889455 | 0.000000 |
| metal sequestration by antimicrobial proteins | 0.000000 | 1.523375 | 1.487208 | 0.000000 | 1.551419 | 0.000000 |

| **PATHWAY** | **colon** | **cortex** | **hippocampus** | **heart** | **kidney** | **lung** |
| --- | --- | --- | --- | --- | --- | --- |
| activation of c3 and c5 | 0.000000 | 0.000000 | 0 | 0.000000 | 1.804257 | 0 |
| transport of bile salts and organic acids metal ions and amine compounds | 0.000000 | 1.714358 | 0 | 0.000000 | 1.686542 | 0 |
| degradation of dvl | 1.855611 | 0.000000 | 0 | 1.762461 | −1.668420 | 0 |
| class i mhc mediated antigen processing presentation | 1.423963 | 0.000000 | 0 | 1.304007 | 0.000000 | 0 |
| response to metal ions | 1.936188 | 0.000000 | 0 | 2.093814 | 0.000000 | 0 |
| dectin 1 mediated noncanonical nf kb signaling | 1.868053 | 0.000000 | 0 | 1.799490 | 0.000000 | 0 |
| metallothioneins bind metals | 1.876295 | 0.000000 | 0 | 1.930447 | 0.000000 | 0 |
| interleukin 1 signaling | 1.708766 | 0.000000 | 0 | 1.821329 | 0.000000 | 0 |
| antigen processing cross presentation | 1.590403 | 0.000000 | 0 | 1.783531 | 0.000000 | 0 |
| regulation of mrna stability by proteins that bind au rich elements | 1.554447 | 0.000000 | 0 | 1.805528 | 0.000000 | 0 |
| metabolism of polyamines | 1.892836 | 0.000000 | 0 | 1.643457 | 0.000000 | 0 |
| auf1 hnrnp d0 binds and destabilizes mrna | 1.752455 | 0.000000 | 0 | 1.652836 | 0.000000 | 0 |
| downstream signaling events of b cell receptor bcr | 1.671700 | 0.000000 | 0 | 1.568439 | 0.000000 | 0 |
| metabolism of nucleotides | 1.666349 | 0.000000 | 0 | 1.609748 | 0.000000 | 0 |
| asymmetric localization of pcp proteins | 1.796125 | 0.000000 | 0 | 0.000000 | −1.622160 | 0 |
| degradation of cysteine and homocysteine | 1.756030 | 0.000000 | 0 | 0.000000 | −1.783458 | 0 |
| hiv infection | 1.516452 | 0.000000 | 0 | 0.000000 | −1.410416 | 0 |
| post translational protein modification | 1.281259 | 0.000000 | 0 | 0.000000 | −1.225102 | 0 |
| copii mediated vesicle transport | 1.745933 | −1.500387 | 0 | 0.000000 | 0.000000 | 0 |
| negative regulation of notch4 signaling | 1.770700 | 0.000000 | 0 | 0.000000 | 0.000000 | 0 |
| degradation of gli1 by the proteasome | 1.735505 | 0.000000 | 0 | 0.000000 | 0.000000 | 0 |
| hedgehog ligand biogenesis | 1.749088 | 0.000000 | 0 | 0.000000 | 0.000000 | 0 |
| defective cftr causes cystic fibrosis | 1.889317 | 0.000000 | 0 | 0.000000 | 0.000000 | 0 |
| ire1alpha activates chaperones | 1.848187 | 0.000000 | 0 | 0.000000 | 0.000000 | 0 |
| degradation of axin | 1.810276 | 0.000000 | 0 | 0.000000 | 0.000000 | 0 |
| unfolded protein response upr | 1.814814 | 0.000000 | 0 | 0.000000 | 0.000000 | 0 |
| clec7a dectin 1 signaling | 1.509853 | 0.000000 | 0 | 0.000000 | 0.000000 | 0 |
| cross presentation of soluble exogenous antigens endosomes | 1.696887 | 0.000000 | 0 | 0.000000 | 0.000000 | 0 |
| cellular response to hypoxia | 1.675545 | 0.000000 | 0 | 0.000000 | 0.000000 | 0 |
| regulation of runx3 expression and activity | 1.672609 | 0.000000 | 0 | 0.000000 | 0.000000 | 0 |
| degradation of beta catenin by the destruction complex | 1.655561 | 0.000000 | 0 | 0.000000 | 0.000000 | 0 |
| n glycan trimming in the er and calnexin calreticulin cycle | 1.654042 | 0.000000 | 0 | 0.000000 | 0.000000 | 0 |
| interleukin 27 signaling | 1.639863 | 0.000000 | 0 | 0.000000 | 0.000000 | 0 |
| regulation of ras by gaps | 1.643579 | 0.000000 | 0 | 0.000000 | 0.000000 | 0 |
| abc transporter disorders | 1.627644 | 0.000000 | 0 | 0.000000 | 0.000000 | 0 |

| **PATHWAY** | **colon** | **cortex** | **hippocampus** | **heart** | **kidney** | **lung** |
| --- | --- | --- | --- | --- | --- | --- |
| fceri mediated nf kb activation | 1.627294 | 0.000000 | 0.000000 | 0.000000 | 0.000000 | 0.000000 |
| apc c cdh1 mediated degradation of cdc20 and other apc c cdh1 targeted proteins in late mitosis early g1 | 1.612195 | 0.000000 | 0.000000 | 0.000000 | 0.000000 | 0.000000 |
| regulation of pten stability and activity | 1.605726 | 0.000000 | 0.000000 | 0.000000 | 0.000000 | 0.000000 |
| the role of gtse1 in g2 m progression after g2 checkpoint | 1.600253 | 0.000000 | 0.000000 | 0.000000 | 0.000000 | 0.000000 |
| mitochondrial protein import | 1.822930 | −2.002037 | −1.906535 | 0.000000 | −1.859199 | 0.000000 |
| protein localization | 1.849551 | −1.972137 | −2.067835 | 0.000000 | −1.789942 | 0.000000 |
| diseases of metabolism | 0.000000 | 0.000000 | −1.422944 | 0.000000 | −1.459217 | 0.000000 |
| metabolism of lipids | 0.000000 | 0.000000 | −1.284075 | 0.000000 | −1.353002 | 0.000000 |
| glutathione conjugation | 0.000000 | 0.000000 | −2.078847 | 0.000000 | −1.606826 | 0.000000 |
| mitochondrial fatty acid beta oxidation | 0.000000 | 0.000000 | −1.690011 | 0.000000 | −1.808306 | 0.000000 |
| peroxisomal protein import | 0.000000 | 0.000000 | −1.727068 | 0.000000 | −1.639636 | 0.000000 |
| metabolism of amino acids and derivatives | 1.612339 | 0.000000 | −1.616403 | 1.415922 | −1.619828 | 0.000000 |
| sulfur amino acid metabolism | 2.019812 | 0.000000 | −1.923759 | 0.000000 | −1.936331 | 0.000000 |
| infectious disease | 1.325519 | 0.000000 | 0.000000 | 1.364947 | 0.000000 | −1.401026 |
| cellular responses to external stimuli | 1.415178 | 0.000000 | 0.000000 | 0.000000 | 0.000000 | −1.717170 |
| runx1 regulates transcription of genes involved in differentiation of hscs | 1.616061 | 0.000000 | 0.000000 | 0.000000 | 0.000000 | −1.770813 |
| selenoamino acid metabolism | 1.630002 | 0.000000 | 0.000000 | 1.566442 | −1.529003 | −1.953737 |
| nonsense mediated decay nmd | 1.757693 | 0.000000 | 0.000000 | 0.000000 | −1.655115 | −2.253848 |
| signaling by robo receptors | 1.564215 | 0.000000 | 0.000000 | 0.000000 | −1.773884 | −2.101780 |
| metabolism of rna | 1.345920 | 0.000000 | −1.160843 | 0.000000 | −1.381398 | −1.616788 |
| pcp ce pathway | 1.738261 | 0.000000 | 0.000000 | 0.000000 | −1.772312 | −1.592112 |
| srp dependent cotranslational protein targeting to membrane | 1.766923 | 0.000000 | −1.618922 | 1.674855 | 0.000000 | −2.240524 |
| eukaryotic translation elongation | 1.835165 | 0.000000 | −1.616606 | 1.618549 | −1.746574 | −2.351813 |
| eukaryotic translation initiation | 1.619063 | 0.000000 | −1.442266 | 1.497475 | −1.556385 | −2.277138 |
| translation | 1.774910 | 0.000000 | −2.034711 | 0.000000 | −1.342425 | −2.034331 |
| regulation of expression of slits and robos | 1.945173 | 0.000000 | −1.660903 | 0.000000 | −1.749951 | −2.190439 |
| response of eif2ak4 gcn2 to amino acid deficiency | 1.849677 | 0.000000 | −1.498703 | 0.000000 | −1.699947 | −2.218469 |
| asparagine n linked glycosylation | 1.440317 | 0.000000 | −1.663714 | 0.000000 | 0.000000 | 0.000000 |
| transport of small molecules | 0.000000 | 0.000000 | −1.432927 | 0.000000 | 0.000000 | 0.000000 |
| vesicle mediated transport | 0.000000 | 0.000000 | −1.242318 | 0.000000 | 0.000000 | 0.000000 |
| membrane trafficking | 0.000000 | 0.000000 | −1.286055 | 0.000000 | 0.000000 | 0.000000 |
| opsins | 0.000000 | 0.000000 | −1.329714 | 0.000000 | 0.000000 | 0.000000 |
| biological oxidations | 0.000000 | 0.000000 | −1.871785 | 0.000000 | 0.000000 | 0.000000 |
| tp53 regulates metabolic genes | 0.000000 | 0.000000 | −1.946204 | 0.000000 | 0.000000 | 0.000000 |
| neurotransmitter receptors and postsynaptic signal transmission | 0.000000 | 0.000000 | −1.562858 | 0.000000 | 0.000000 | 0.000000 |

| **PATHWAY** | **colon** | **cortex** | **hippocampus** | **heart** | **kidney** | **lung** |
| --- | --- | --- | --- | --- | --- | --- |
| hiv transcription initiation | 0.00000 | 0.000000 | −1.636935 | 0.000000 | 0.000000 | 0.000000 |
| phase ii conjugation of compounds | 0.00000 | 0.000000 | −1.643788 | 0.000000 | 0.000000 | 0.000000 |
| calnexin calreticulin cycle | 0.00000 | 0.000000 | −1.786011 | 0.000000 | 0.000000 | 0.000000 |
| neuronal system | 0.00000 | 0.000000 | −1.739847 | 0.000000 | 0.000000 | 0.000000 |
| notch2 activation and transmission of signal to the nucleus | 0.00000 | 0.000000 | −1.723644 | 0.000000 | 0.000000 | 0.000000 |
| phase i functionalization of compounds | 0.00000 | 0.000000 | −1.731066 | 0.000000 | 0.000000 | 0.000000 |
| pyruvate metabolism | 0.00000 | 0.000000 | −1.707095 | 0.000000 | 0.000000 | 0.000000 |
| transmission across chemical synapses | 0.00000 | 0.000000 | −1.685324 | 0.000000 | 0.000000 | 0.000000 |
| cooperation of prefoldin and tric cct in actin and tubulin folding | 0.00000 | −2.104430 | −2.078086 | 0.000000 | 0.000000 | −1.830029 |
| formation of tubulin folding intermediates by cct tric | 0.00000 | −2.057046 | −1.930155 | 0.000000 | 0.000000 | −1.812391 |
| er to golgi anterograde transport | 0.00000 | −1.660771 | −1.641173 | 0.000000 | 0.000000 | −1.517257 |
| transport of connexons to the plasma membrane | 0.00000 | −1.716431 | −1.877021 | 0.000000 | 0.000000 | −1.715934 |
| citric acid cycle tca cycle | 0.00000 | −1.770346 | −1.848563 | −1.792356 | 0.000000 | 0.000000 |
| formation of atp by chemiosmotic coupling | 0.00000 | −2.043299 | −2.083723 | −1.697148 | 0.000000 | 0.000000 |
| mitochondrial translation | 1.68783 | −1.835346 | −2.273042 | 0.000000 | 0.000000 | 0.000000 |
| cristae formation | 0.00000 | −2.180573 | −2.065570 | 0.000000 | 0.000000 | 0.000000 |
| copi mediated anterograde transport | 0.00000 | −1.708927 | −1.839001 | 0.000000 | 0.000000 | 0.000000 |
| pyruvate metabolism and citric acid tca cycle | 0.00000 | −1.630244 | −1.740782 | 0.000000 | 0.000000 | 0.000000 |
| mitochondrial biogenesis | 0.00000 | −1.724129 | −1.630531 | 0.000000 | 0.000000 | 0.000000 |
| autophagy | 0.00000 | −1.586806 | −1.571281 | 0.000000 | 0.000000 | −1.182287 |
| transport to the golgi and subsequent modification | 0.00000 | −1.596263 | −1.593251 | 0.000000 | 0.000000 | 0.000000 |
| influenza infection | 0.00000 | 0.000000 | −1.474243 | 0.000000 | −1.534374 | −2.026208 |
| mrna splicing minor pathway | 0.00000 | 0.000000 | 0.000000 | 0.000000 | −1.653789 | −1.626740 |
| cell cell junction organization | 0.00000 | 0.000000 | 0.000000 | 0.000000 | −1.555348 | −1.654818 |
| mrna splicing | 0.00000 | 0.000000 | 0.000000 | 0.000000 | −1.532567 | −1.724387 |
| nervous system development | 0.00000 | 0.000000 | 0.000000 | 0.000000 | −1.499876 | −1.700361 |
| diseases of signal transduction by growth factor receptors and second messengers | 0.00000 | 0.000000 | 0.000000 | 0.000000 | −1.476492 | −1.453817 |
| cell junction organization | 0.00000 | 0.000000 | 0.000000 | 0.000000 | −1.342407 | −1.554582 |
| developmental biology | 0.00000 | 0.000000 | 0.000000 | 0.000000 | −1.383968 | −1.663843 |
| processing of capped intron containing pre mrna | 0.00000 | 0.000000 | 0.000000 | 0.000000 | −1.446985 | −1.601311 |
| hsp90 chaperone cycle for steroid hormone receptors shr | 0.00000 | 0.000000 | −1.699167 | 0.000000 | 0.000000 | −1.622856 |
| selective autophagy | 0.00000 | 0.000000 | −1.629737 | 0.000000 | 0.000000 | −1.546426 |
| activation of ampk downstream of nmdars | 0.00000 | 0.000000 | −1.754857 | 0.000000 | 0.000000 | −1.864489 |
| hedgehog off state | 0.00000 | 0.000000 | −1.522559 | 0.000000 | 0.000000 | −1.855955 |
| signaling by hedgehog | 0.00000 | 0.000000 | −1.503509 | 0.000000 | 0.000000 | −1.812936 |

| **PATHWAY** | **colon** | **cortex** | **hippocampus** | **heart** | **kidney** | **lung** |
| --- | --- | --- | --- | --- | --- | --- |
| cilium assembly | 0 | 0 | 0 | 0 | 0 | −2.298640 |
| intraflagellar transport | 0 | 0 | 0 | 0 | 0 | −2.534686 |
| cell cycle | 0 | 0 | 0 | 0 | 0 | −1.458243 |
| s phase | 0 | 0 | 0 | 0 | 0 | −1.470035 |
| cell cycle mitotic | 0 | 0 | 0 | 0 | 0 | −1.536582 |
| chromatin modifying enzymes | 0 | 0 | 0 | 0 | 0 | −1.530395 |
| mitotic g2 g2 m phases | 0 | 0 | 0 | 0 | 0 | −1.513137 |
| signaling by nuclear receptors | 0 | 0 | 0 | 0 | 0 | −1.493872 |
| epigenetic regulation of gene expression | 0 | 0 | 0 | 0 | 0 | −1.500867 |
| ub specific processing proteases | 0 | 0 | 0 | 0 | 0 | −1.505181 |
| m phase | 0 | 0 | 0 | 0 | 0 | −1.563352 |
| transcriptional regulation by runx1 | 0 | 0 | 0 | 0 | 0 | −1.564997 |
| esr mediated signaling | 0 | 0 | 0 | 0 | 0 | −1.592946 |
| sumoylation of transcription cofactors | 0 | 0 | 0 | 0 | 0 | −1.577577 |
| hats acetylate histones | 0 | 0 | 0 | 0 | 0 | −1.578843 |
| signaling by notch1 | 0 | 0 | 0 | 0 | 0 | −1.579325 |
| gene silencing by rna | 0 | 0 | 0 | 0 | 0 | −1.644317 |
| signaling by notch4 | 0 | 0 | 0 | 0 | 0 | −1.644160 |
| negative regulation of mapk pathway | 0 | 0 | 0 | 0 | 0 | −1.649062 |
| rna polymerase i promoter escape | 0 | 0 | 0 | 0 | 0 | −1.650557 |
| signaling by notch | 0 | 0 | 0 | 0 | 0 | −1.663921 |
| signaling by fgfr2 | 0 | 0 | 0 | 0 | 0 | −1.666928 |
| foxo mediated transcription | 0 | 0 | 0 | 0 | 0 | −1.666713 |
| regulation of hsf1 mediated heat shock response | 0 | 0 | 0 | 0 | 0 | −1.666688 |
| notch1 intracellular domain regulates transcription | 0 | 0 | 0 | 0 | 0 | −1.659410 |
| mitotic prophase | 0 | 0 | 0 | 0 | 0 | −1.655949 |
| negative epigenetic regulation of rrna expression | 0 | 0 | 0 | 0 | 0 | −1.653299 |
| dna damage recognition in gg ner | 0 | 0 | 0 | 0 | 0 | −1.609237 |
| cellular response to heat stress | 0 | 0 | 0 | 0 | 0 | −1.612765 |
| programmed cell death | 0 | 0 | 0 | 0 | 0 | −1.615624 |
| e3 ubiquitin ligases ubiquitinate target proteins | 0 | 0 | 0 | 0 | 0 | −1.637130 |
| activation of bh3 only proteins | 0 | 0 | 0 | 0 | 0 | −1.634449 |
| signaling by notch1 pest domain mutants in cancer | 0 | 0 | 0 | 0 | 0 | −1.635264 |
| recruitment of mitotic centrosome proteins and complexes | 0 | 0 | 0 | 0 | 0 | −1.629540 |
| activation of anterior hox genes in hindbrain development during early embryogenesis | 0 | 0 | 0 | 0 | 0 | −1.625760 |

| **PATHWAY** | **colon** | **cortex** | **hippocampus** | **heart** | **kidney** | **lung** |
| --- | --- | --- | --- | --- | --- | --- |
| protein folding | 0 | −1.298164 | −1.297289 | 0 | 0.000000 | −1.626484 |
| carboxyterminal post translational modifications of tubulin | 0 | 0.000000 | 0.000000 | 0 | 0.000000 | −2.104265 |
| rho gtpases activate pkns | 0 | 0.000000 | 0.000000 | 0 | 0.000000 | −2.094049 |
| formation of the beta catenin tcf transactivating complex | 0 | 0.000000 | 0.000000 | 0 | 0.000000 | −1.996136 |
| ercc6 csb and ehmt2 g9a positively regulate rrna expression | 0 | 0.000000 | 0.000000 | 0 | 0.000000 | −2.044318 |
| organelle biogenesis and maintenance | 0 | 0.000000 | 0.000000 | 0 | 0.000000 | −2.031327 |
| apoptosis induced dna fragmentation | 0 | 0.000000 | 0.000000 | 0 | 0.000000 | −1.769790 |
| translocation of slc2a4 glut4 to the plasma membrane | 0 | 0.000000 | 0.000000 | 0 | 0.000000 | −1.770304 |
| reproduction | 0 | 0.000000 | 0.000000 | 0 | 0.000000 | −1.775646 |
| smooth muscle contraction | 0 | 0.000000 | 0.000000 | 0 | 0.000000 | −1.780729 |
| protein ubiquitination | 0 | 0.000000 | 0.000000 | 0 | 0.000000 | −1.748546 |
| rna polymerase i transcription | 0 | 0.000000 | 0.000000 | 0 | 0.000000 | −1.763562 |
| notch4 intracellular domain regulates transcription | 0 | 0.000000 | 0.000000 | 0 | 0.000000 | −1.761089 |
| rrna processing | 0 | 0.000000 | 0.000000 | 0 | 0.000000 | −1.761972 |
| fgfr2 alternative splicing | 0 | 0.000000 | 0.000000 | 0 | 0.000000 | −1.759055 |
| oxidative stress induced senescence | 0 | 0.000000 | 0.000000 | 0 | 0.000000 | −1.756735 |
| signaling by wnt | 0 | 0.000000 | 0.000000 | 0 | −1.293817 | −1.755067 |
| base excision repair | 0 | 0.000000 | 0.000000 | 0 | 0.000000 | −1.731155 |
| condensation of prophase chromosomes | 0 | 0.000000 | 0.000000 | 0 | 0.000000 | −1.733687 |
| hsf1 activation | 0 | 0.000000 | 0.000000 | 0 | 0.000000 | −1.734824 |
| amyloid fiber formation | 0 | 0.000000 | 0.000000 | 0 | 0.000000 | −1.713893 |
| deactivation of the beta catenin transactivating complex | 0 | 0.000000 | 0.000000 | 0 | 0.000000 | −1.714078 |
| recognition and association of dna glycosylase with site containing an affected purine | 0 | 0.000000 | 0.000000 | 0 | 0.000000 | −1.719686 |
| rho gtpases activate iqgaps | 0 | 0.000000 | 0.000000 | 0 | 0.000000 | −1.721872 |
| rmts methylate histone arginines | 0 | 0.000000 | 0.000000 | 0 | 0.000000 | −1.677558 |
| signaling by notch2 | 0 | 0.000000 | 0.000000 | 0 | 0.000000 | −1.676457 |
| aggrephagy | 0 | 0.000000 | 0.000000 | 0 | 0.000000 | −1.691109 |
| rho gtpases activate paks | 0 | 0.000000 | 0.000000 | 0 | 0.000000 | −1.692526 |
| transcriptional regulation by small rnas | 0 | 0.000000 | 0.000000 | 0 | 0.000000 | −1.693908 |
| constitutive signaling by aberrant pi3k in cancer | 0 | 0.000000 | 0.000000 | 0 | 0.000000 | −1.701024 |
| uptake and actions of bacterial toxins | 0 | 0.000000 | 0.000000 | 0 | 0.000000 | −1.700399 |
| hcmv infection | 0 | 0.000000 | 0.000000 | 0 | 0.000000 | −1.707051 |
| hsf1 dependent transactivation | 0 | 0.000000 | 0.000000 | 0 | 0.000000 | −1.706210 |
| tcf dependent signaling in response to wnt | 0 | 0.000000 | 0.000000 | 0 | 0.000000 | −1.907166 |
| inhibition of dna recombination at telomere | 0 | 0.000000 | 0.000000 | 0 | 0.000000 | −1.913344 |

| **PATHWAY** | **colon** | **cortex** | **hippocampus** | **heart** | **kidney** | **lung** |
| --- | --- | --- | --- | --- | --- | --- |
| telomere maintenance | 0 | 0.000000 | 0.000000 | 0.000000 | 0.000000 | −1.916630 |
| non integrin membrane ecm interactions | 0 | 0.000000 | 0.000000 | 0.000000 | 0.000000 | −1.962766 |
| activation of the mrna upon binding of the cap binding complex and eifs and subsequent binding to 43s | 0 | 0.000000 | 0.000000 | 0.000000 | 0.000000 | −1.941541 |
| egr2 and sox10 mediated initiation of schwann cell myelination | 0 | 0.000000 | 0.000000 | 0.000000 | 0.000000 | −1.948813 |
| attenuation phase | 0 | 0.000000 | 0.000000 | 0.000000 | 0.000000 | −1.866640 |
| meiotic synapsis | 0 | 0.000000 | 0.000000 | 0.000000 | 0.000000 | −1.859464 |
| positive epigenetic regulation of rrna expression | 0 | 0.000000 | 0.000000 | 0.000000 | 0.000000 | −1.860615 |
| negative regulation of the pi3k akt network | 0 | 0.000000 | 0.000000 | 0.000000 | 0.000000 | −1.856890 |
| signaling by hippo | 0 | 0.000000 | 0.000000 | 0.000000 | 0.000000 | −1.855189 |
| anchoring of the basal body to the plasma membrane | 0 | 0.000000 | 0.000000 | 0.000000 | 0.000000 | −1.792974 |
| laminin interactions | 0 | 0.000000 | 0.000000 | 0.000000 | 0.000000 | −1.791586 |
| base excision repair ap site formation | 0 | 0.000000 | 0.000000 | 0.000000 | 0.000000 | −1.805095 |
| hcmv early events | 0 | 0.000000 | 0.000000 | 0.000000 | 0.000000 | −1.798696 |
| epha mediated growth cone collapse | 0 | 0.000000 | 0.000000 | 0.000000 | 0.000000 | −1.821461 |
| b wich complex positively regulates rrna expression | 0 | 0.000000 | 0.000000 | 0.000000 | 0.000000 | −1.836649 |
| chromosome maintenance | 0 | 0.000000 | 0.000000 | 0.000000 | 0.000000 | −1.835746 |
| gap junction trafficking and regulation | 0 | 0.000000 | 0.000000 | 0.000000 | 0.000000 | −1.835320 |
| senescence associated secretory phenotype sasp | 0 | 0.000000 | 0.000000 | 0.000000 | 0.000000 | −1.828183 |
| signaling by fgfr | 0 | 0.000000 | 0.000000 | 0.000000 | 0.000000 | −1.830528 |
| assembly and cell surface presentation of nmda receptors | 0 | −1.997519 | 0.000000 | 0.000000 | −1.759173 | −1.835658 |
| recruitment of numa to mitotic centrosomes | 0 | −1.564606 | 0.000000 | 0.000000 | 0.000000 | −1.689501 |
| biosynthesis of maresins | 0 | −1.480480 | 0.000000 | 0.000000 | 0.000000 | 0.000000 |
| copi dependent golgi to er retrograde traffic | 0 | −1.503556 | 0.000000 | 0.000000 | 0.000000 | 0.000000 |
| protein protein interactions at synapses | 0 | −1.547111 | 0.000000 | 0.000000 | 0.000000 | 0.000000 |
| golgi to er retrograde transport | 0 | −1.582437 | 0.000000 | 0.000000 | 0.000000 | 0.000000 |
| synthesis of active ubiquitin roles of e1 and e2 enzymes | 0 | −1.607505 | 0.000000 | 0.000000 | 0.000000 | 0.000000 |
| neurotransmitter release cycle | 0 | −1.659708 | 0.000000 | 0.000000 | 0.000000 | 0.000000 |
| neurexins and neuroligins | 0 | −1.704242 | 0.000000 | 0.000000 | 0.000000 | 0.000000 |
| copi independent golgi to er retrograde traffic | 0 | −1.739349 | 0.000000 | 0.000000 | 0.000000 | 0.000000 |
| folding of actin by cct tric | 0 | −1.744028 | 0.000000 | 0.000000 | 0.000000 | 0.000000 |
| mitochondrial iron sulfur cluster biogenesis | 0 | −1.808733 | 0.000000 | 0.000000 | 0.000000 | 0.000000 |
| golgi cisternae pericentriolar stack reorganization | 0 | −1.912602 | 0.000000 | 0.000000 | 0.000000 | 0.000000 |
| post chaperonin tubulin folding pathway | 0 | −1.872761 | 0.000000 | 0.000000 | 0.000000 | 0.000000 |
| complex i biogenesis | 0 | −2.562063 | −2.365290 | −1.783144 | −2.052063 | 0.000000 |
| the citric acid tca cycle and respiratory electron transport | 0 | −2.666418 | −2.678674 | −1.941198 | −2.036375 | 0.000000 |

| **PATHWAY** | **colon** | **cortex** | **hippocampus** | **heart** | **kidney** | **lung** |
| --- | --- | --- | --- | --- | --- | --- |
| respiratory electron transport | 0.000000 | −2.782151 | −2.745683 | −1.951907 | −2.141375 | 0.000000 |
| respiratory electron transport atp synthesis by chemiosmotic coupling and heat production by uncoupling proteins | 0.000000 | −2.819193 | −2.721798 | −2.028755 | −2.195784 | 0.000000 |
| muscle contraction | 0.000000 | 0.000000 | 0.000000 | −1.617837 | 0.000000 | −1.473672 |
| release of hh np from the secreting cell | 0.000000 | 0.000000 | 0.000000 | −1.551947 | 0.000000 | 0.000000 |
| passive transport by aquaporins | 0.000000 | 0.000000 | 0.000000 | −1.465966 | 0.000000 | 0.000000 |
| activation of trka receptors | 0.000000 | 0.000000 | 0.000000 | −1.499718 | 0.000000 | 0.000000 |
| ngf independant trka activation | 0.000000 | 0.000000 | 0.000000 | −1.499718 | 0.000000 | 0.000000 |
| coenzyme a biosynthesis | 0.000000 | 0.000000 | 0.000000 | −1.670186 | 0.000000 | 0.000000 |
| met promotes cell motility | 0.000000 | 0.000000 | 0.000000 | −1.670810 | 0.000000 | 0.000000 |
| defects in biotin btn metabolism | 0.000000 | 0.000000 | 0.000000 | −1.660192 | 0.000000 | 0.000000 |
| nucleotide like purinergic receptors | 0.000000 | 0.000000 | 0.000000 | −1.652188 | 0.000000 | 0.000000 |
| syndecan interactions | 0.000000 | 0.000000 | 0.000000 | −1.654236 | 0.000000 | 0.000000 |
| a tetrasaccharide linker sequence is required for gag synthesis | 0.000000 | 0.000000 | 0.000000 | −1.850201 | 0.000000 | 0.000000 |
| defective b4galt7 causes eds progeroid type | 0.000000 | 0.000000 | 0.000000 | −1.848182 | 0.000000 | 0.000000 |
| hs gag degradation | 0.000000 | 0.000000 | 0.000000 | −1.766912 | 0.000000 | 0.000000 |
| met activates ptk2 signaling | 0.000000 | 0.000000 | 0.000000 | −1.758589 | 0.000000 | 0.000000 |
| cardiac conduction | −1.683592 | 0.000000 | 0.000000 | −1.850176 | 0.000000 | 0.000000 |
| striated muscle contraction | −1.793022 | 0.000000 | 0.000000 | −1.670354 | 0.000000 | 0.000000 |
| g alpha s signalling events | −1.521841 | 0.000000 | 0.000000 | 0.000000 | 0.000000 | 0.000000 |
| long term potentiation | −1.460153 | 0.000000 | 0.000000 | 0.000000 | 0.000000 | 0.000000 |
| interaction between l1 and ankyrins | −1.931240 | 0.000000 | 0.000000 | 0.000000 | 0.000000 | 0.000000 |
| transcriptional regulation by mecp2 | −1.849972 | 0.000000 | 0.000000 | 0.000000 | 0.000000 | 0.000000 |
| erythrocytes take up carbon dioxide and release oxygen | −1.887587 | 0.000000 | 0.000000 | 0.000000 | 0.000000 | 0.000000 |
| erythrocytes take up oxygen and release carbon dioxide | −1.889636 | 0.000000 | 0.000000 | 0.000000 | 0.000000 | 0.000000 |
| collagen chain trimerization | −1.697145 | 0.000000 | 0.000000 | −2.028254 | −2.239604 | 0.000000 |
| assembly of collagen fibrils and other multimeric structures | 0.000000 | 0.000000 | 0.000000 | −1.795334 | −1.861085 | 0.000000 |
| collagen formation | 0.000000 | 0.000000 | 0.000000 | −1.567907 | −1.743295 | 0.000000 |
| negative regulation of nmda receptor mediated neuronal transmission | −2.007119 | 0.000000 | 0.000000 | 0.000000 | −1.758479 | 0.000000 |
| phase 0 rapid depolarisation | −2.142854 | 0.000000 | 0.000000 | 0.000000 | −1.863845 | 0.000000 |
| activation of nmda receptors and postsynaptic events | 0.000000 | −1.553360 | 0.000000 | 0.000000 | −1.756183 | 0.000000 |
| synthesis of pa | 0.000000 | 0.000000 | 0.000000 | 0.000000 | −2.097031 | 0.000000 |
| elastic fibre formation | 0.000000 | 0.000000 | 0.000000 | 0.000000 | −2.152587 | 0.000000 |
| molecules associated with elastic fibres | 0.000000 | 0.000000 | 0.000000 | 0.000000 | −2.145204 | 0.000000 |
| unblocking of nmda receptors glutamate binding and activation | 0.000000 | 0.000000 | 0.000000 | 0.000000 | −1.830585 | 0.000000 |
| defects of contact activation system cas and kallikrein kinin system kks | 0.000000 | 0.000000 | 0.000000 | 0.000000 | −1.821781 | 0.000000 |

| **PATHWAY** | **colon** | **cortex** | **hippocampus** | **heart** | **kidney** | **lung** |
| --- | --- | --- | --- | --- | --- | --- |
| diseases associated with o glycosylation of proteins | 0 | 0 | 0 | 0 | −1.823702 | 0 |
| gaba synthesis release reuptake and degradation | 0 | 0 | 0 | 0 | −1.846215 | 0 |
| rna polymerase ii transcription termination | 0 | 0 | 0 | 0 | −1.836834 | 0 |
| trafficking of ampa receptors | 0 | 0 | 0 | 0 | −1.841371 | 0 |
| collagen degradation | 0 | 0 | 0 | 0 | −1.977985 | 0 |
| collagen biosynthesis and modifying enzymes | 0 | 0 | 0 | 0 | −1.917576 | 0 |
| ecm proteoglycans | 0 | 0 | 0 | 0 | −1.883107 | 0 |
| mineralocorticoid biosynthesis | 0 | 0 | 0 | 0 | −1.324665 | 0 |
| disease | 0 | 0 | 0 | 0 | −1.347186 | 0 |
| signaling by receptor tyrosine kinases | 0 | 0 | 0 | 0 | −1.340355 | 0 |
| fc epsilon receptor fceri signaling | 0 | 0 | 0 | 0 | −1.530316 | 0 |
| intracellular signaling by second messengers | 0 | 0 | 0 | 0 | −1.548103 | 0 |
| phospholipid metabolism | 0 | 0 | 0 | 0 | −1.552465 | 0 |
| flt3 signaling | 0 | 0 | 0 | 0 | −1.621203 | 0 |
| mapk family signaling cascades | 0 | 0 | 0 | 0 | −1.612789 | 0 |
| regulation of runx2 expression and activity | 0 | 0 | 0 | 0 | −1.614667 | 0 |
| c type lectin receptors clrs | 0 | 0 | 0 | 0 | −1.586596 | 0 |
| pi3k akt signaling in cancer | 0 | 0 | 0 | 0 | −1.582465 | 0 |
| hedgehog on state | 0 | 0 | 0 | 0 | −1.600256 | 0 |
| signaling by vegf | 0 | 0 | 0 | 0 | −1.593845 | 0 |
| transport of nucleosides and free purine and pyrimidine bases across the plasma membrane | 0 | 0 | 0 | 0 | −1.761568 | 0 |
| tnfr1 induced nfkappab signaling pathway | 0 | 0 | 0 | 0 | −1.749345 | 0 |
| beta catenin independent wnt signaling | 0 | 0 | 0 | 0 | −1.750996 | 0 |
| regulation of tnfr1 signaling | 0 | 0 | 0 | 0 | −1.750472 | 0 |
| acetylcholine neurotransmitter release cycle | 0 | 0 | 0 | 0 | −1.733268 | 0 |
| ncam1 interactions | 0 | 0 | 0 | 0 | −1.720367 | 0 |
| intrinsic pathway of fibrin clot formation | 0 | 0 | 0 | 0 | −1.717447 | 0 |
| platelet activation signaling and aggregation | 0 | 0 | 0 | 0 | −1.717176 | 0 |
| energy dependent regulation of mtor by lkb1 ampk | 0 | 0 | 0 | 0 | −1.639081 | 0 |
| creatine metabolism | 0 | 0 | 0 | 0 | −1.651578 | 0 |
| sulfide oxidation to sulfate | 0 | 0 | 0 | 0 | −1.659258 | 0 |
| nuclear signaling by erbb4 | 0 | 0 | 0 | 0 | −1.701081 | 0 |
| resolution of abasic sites ap sites | 0 | 0 | 0 | 0 | −1.698137 | 0 |
| cation coupled chloride cotransporters | 0 | 0 | 0 | 0 | −1.686621 | 0 |
| tcr signaling | 0 | 0 | 0 | 0 | −1.680302 | 0 |
